# Eight millennia of continuity of a previously unknown lineage in Argentina

**DOI:** 10.1038/s41586-025-09731-3

**Published:** 2025-11-05

**Authors:** Javier Maravall-López, Josefina M. B. Motti, Nicolás Pastor, M. Pía Tavella, Mariana Fabra, M. Pilar Babot, Mariano Bonomo, Silvia E. Cornero, Guillermo N. Lamenza, Diego Catriel Leon, Paula C. Miranda de Zela, Gustavo G. Politis, Sofía C. Angeletti, G. Roxana Cattáneo, Mariana Dantas, Hilton Drube, Lucia G. Gonzalez Baroni, Salomón Hocsman, Andrés D. Izeta, Reinaldo A. Moralejo, Verónica Aldazabal, Diego M. Basso, Cristina Bayón, María Guillermina Couso, Ulises D’Andrea, Paula Del Río, Germán G. Figueroa, Romina Frontini, Mariela Edith Gonzalez, Andrés G. Laguens, Jorge G. Martínez, Pablo G. Messineo, Beatriz Nores, Daniel E. Olivera, Gisela M. Sario, Analía Sbattella, Clara Scabuzzo, Aldana M. Tavarone, Rodrigo Vecchi, Kim Callan, Ella Caughran, Oscar Estrada, Trudi Frost, Lora Iliev, Aisling Kearns, Jack Kellogg, Kim-Louise Krettek, Ann Marie Lawson, Matthew Mah, Nihal Manjila, Adam Micco, Iris Patterson, Lijun Qiu, Xavier Roca-Rada, Gregory Soos, Peter A. Webb, J. Noah Workman, Nadin Rohland, Nick Patterson, Iosif Lazaridis, Lars Fehren-Schmitz, Cosimo Posth, Bastien Llamas, Swapan Mallick, Darío A. Demarchi, Graciela S. Cabana, David Reich, Rodrigo Nores

**Affiliations:** 1 Department of Human Evolutionary Biology, Harvard University, Cambridge, Massachusetts, USA; 2 Department of Epidemiology, Harvard School of Public Health, Boston, Massachusetts, USA; 3 Broad Institute of MIT and Harvard, Cambridge, Massachusetts, USA; 4 Laboratorio de Ecología Evolutiva Humana, Facultad de Ciencias Sociales, Universidad Nacional del Centro de la Provincia de Buenos Aires, Consejo Nacional de Investigaciones Científicas y Técnicas, Quequén, Argentina; 5 Instituto de Antropología de Córdoba, Consejo Nacional de Investigaciones Científicas y Técnicas, Córdoba, Argentina; 6 Departamento de Diversidad Biológica y Ecología, Facultad de Ciencias Exactas, Físicas y Naturales, Universidad Nacional de Córdoba, Córdoba, Argentina; 7 Museo de Antropologías, Facultad de Filosofía y Humanidades, Universidad Nacional de Córdoba, Córdoba, Argentina; 8 Departamento de Antropología, Facultad de Filosofía y Humanidades, Universidad Nacional de Córdoba, Córdoba, Argentina; 9 Centro Científico Tecnológico CONICET NOA Sur, Consejo Nacional de Investigaciones Científicas y Técnicas, San Miguel de Tucumán, Argentina; 10 Grupo de Investigación en Arqueología Andina & Instituto de Arqueología y Museo, Facultad de Ciencias Naturales e Instituto Miguel Lillo, Universidad Nacional de Tucumán, San Miguel de Tucumán, Argentina; 11 División Arqueología, Facultad de Ciencias Naturales y Museo, Universidad Nacional de La Plata, Consejo Nacional de Investigaciones Científicas y Técnicas, La Plata, Argentina; 12 Museo Universitario Florentino y Carlos Ameghino, Facultad de Ciencias Exactas, Ingeniería y Agrimensura, Universidad Nacional de Rosario, Rosario, Argentina; 13 División Antropología, Facultad de Ciencias Naturales y Museo, Universidad Nacional de La Plata, Consejo Nacional de Investigaciones Científicas y Técnicas, La Plata, Argentina; 14 Instituto de Estudios para el Desarrollo Social, Universidad Nacional de Santiago del Estero, Consejo Nacional de Investigaciones Científicas y Técnicas, Santiago del Estero, Argentina; 15 Instituto de Linguística, Folklore y Arqueología, Facultad de Humanidades, Ciencias Sociales y de la Salud, Universidad Nacional de Santiago del Estero, Santiago del Estero, Argentina; 16 Banco Nacional de Datos Genéticos, Buenos Aires, Argentina; 17 Instituto de Investigaciones Arqueológicas y Paleontológicas del Cuaternario Pampeano, Facultad de Ciencias Sociales, Universidad Nacional del Centro de la Provincia de Buenos Aires, Consejo Nacional de Investigaciones Científicas y Técnicas, Olavarría, Buenos Aires, Argentina; 18 Centro de Genética Forense, Poder Judicial de la Provincia de Córdoba, Córdoba, Argentina; 19 Centro Científico Tecnológico Córdoba, Consejo Nacional de Investigaciones Científicas y Técnicas, Córdoba, Argentina; 20 Facultad de Ciencias Médicas, Universidad Nacional de Santiago del Estero, Santiago del Estero, Argentina; 21 Facultad de Ciencias Exactas y Naturales, Universidad Nacional de Catamarca, San Fernando del Valle de Catamarca, Argentina; 22 Instituto Multidisciplinario de Historia y Ciencias Humanas, Consejo Nacional de Investigaciones Científicas y Técnicas, Buenos Aires, Argentina; 23 Centro Regional de Estudios Arqueológicos, Facultad de Humanidades y Ciencias Sociales, Universidad Nacional de Jujuy, San Salvador de Jujuy, Argentina; 24 Departamento de Humanidades, Universidad Nacional del Sur, Bahía Blanca, Buenos Aires, Argentina; 25 Fundación de Historia Natural Félix de Azara, Ciudad Autónoma de Buenos Aires, Argentina; 26 Junta Municipal de Historia de Río Cuarto, Departamento de Antropología y Prehistoria, Río Cuarto, Córdoba, Argentina; 27 Museo Universitario Florentino y Carlos Ameghino, Facultad de Ciencias Exactas, Ingeniería y Agrimensura, Universidad Nacional de Rosario, Rosario, Argentina; 28 Instituto Nacional de Antropología y Pensamiento Latinoamericano, Consejo Nacional de Investigaciones Científicas y Técnicas, Buenos Aires, Argentina; 29 Facultad de Filosofía y Letras, Universidad de Buenos Aires, Buenos Aires, Argentina; 30 Dirección General de Patrimonio Cultural, Provincia de Santiago del Estero, Santiago del Estero, Argentina; 31 Centro de Investigación Científica y de Transferencia Tecnológica a la Producción, Consejo Nacional de Investigaciones Científicas y Técnicas, Universidad Autónoma de Entre Ríos, Diamante, Entre Ríos, Argentina; 32 Department of Genetics, Harvard Medical School, Boston, Massachusetts, USA; 33 Howard Hughes Medical Institute, Harvard Medical School, Boston, Massachusetts, USA; 34 Centre for Anthropobiology and Genomics of Toulouse (CAGT), CNRS UMR 5288, Université Toulouse III - Paul Sabatier, Toulouse, France; 35 Senckenberg Centre for Human Evolution and Palaeoenvironment at the University of Tübingen, Tübingen, Germany; 36 Australian Centre for Ancient DNA, School of Biological Sciences, University of Adelaide, Adelaide, SA, Australia; 37 UCSC Paleogenomics, Department of Anthropology, University of California, Santa Cruz, California, USA; 38 UCSC Genomics Institute, University of California, Santa Cruz, California, USA; 39 Archaeo- and Palaeogenetics, Institute for Archaeological Sciences, Department of Geosciences, University of Tübingen, Tübingen, Germany; 40 ARC Centre of Excellence for Australian Biodiversity and Heritage, University of Adelaide, Adelaide, SA, Australia; 41 National Centre for Indigenous Genomics, John Curtin School of Medical Research, Australian National University, Canberra, ACT, Australia Indigenous Genomics, The Kids Research Institute Australia, Adelaide, SA, Australia; 42 Molecular Anthropology Laboratories, Department of Anthropology, University of Tennessee, Knoxville, Tennessee, USA

## Abstract

The central Southern Cone of South America was one of the last regions of the globe to be peopled^1^, yet remains underrepresented in ancient DNA. We generated new genome-wide data from 238 ancient individuals spanning ten millennia. The oldest, from the Pampas region and dating to 10000 years before present (BP), had distinct genetic affinity with Middle Holocene Southern Cone individuals, showing that differentiation from the Central Andes and Central East Brazil had begun by this time. Individuals dating to 4600-150BP primarily descended from a hitherto-unsampled deep lineage whose earliest representative is an individual dating to around 8500BP. This Central Argentina lineage co-existed with two other lineages during the Mid-Holocene, and within Central Argentina, this ancestry persisted for thousands of years with no evidence of interregional migration. Central Argentina ancestry was involved in three distinct gene flows: it mixed into the Pampas by 3300BP and became the main component there after 800BP; with Central Andes ancestry in Northwest Argentina; and with Tropical and Subtropical Forest ancestry in the Gran Chaco. In Northwest Argentina, there was an increased rate of close kin unions by 1000BP, paralleling the pattern in the Central Andes. In the Paraná River region, a 400BP individual with a Guaraní archaeological association clusters with Brazilian groups, consistent with Guaraní presence by this time.

## Introduction

**Figure 1.**
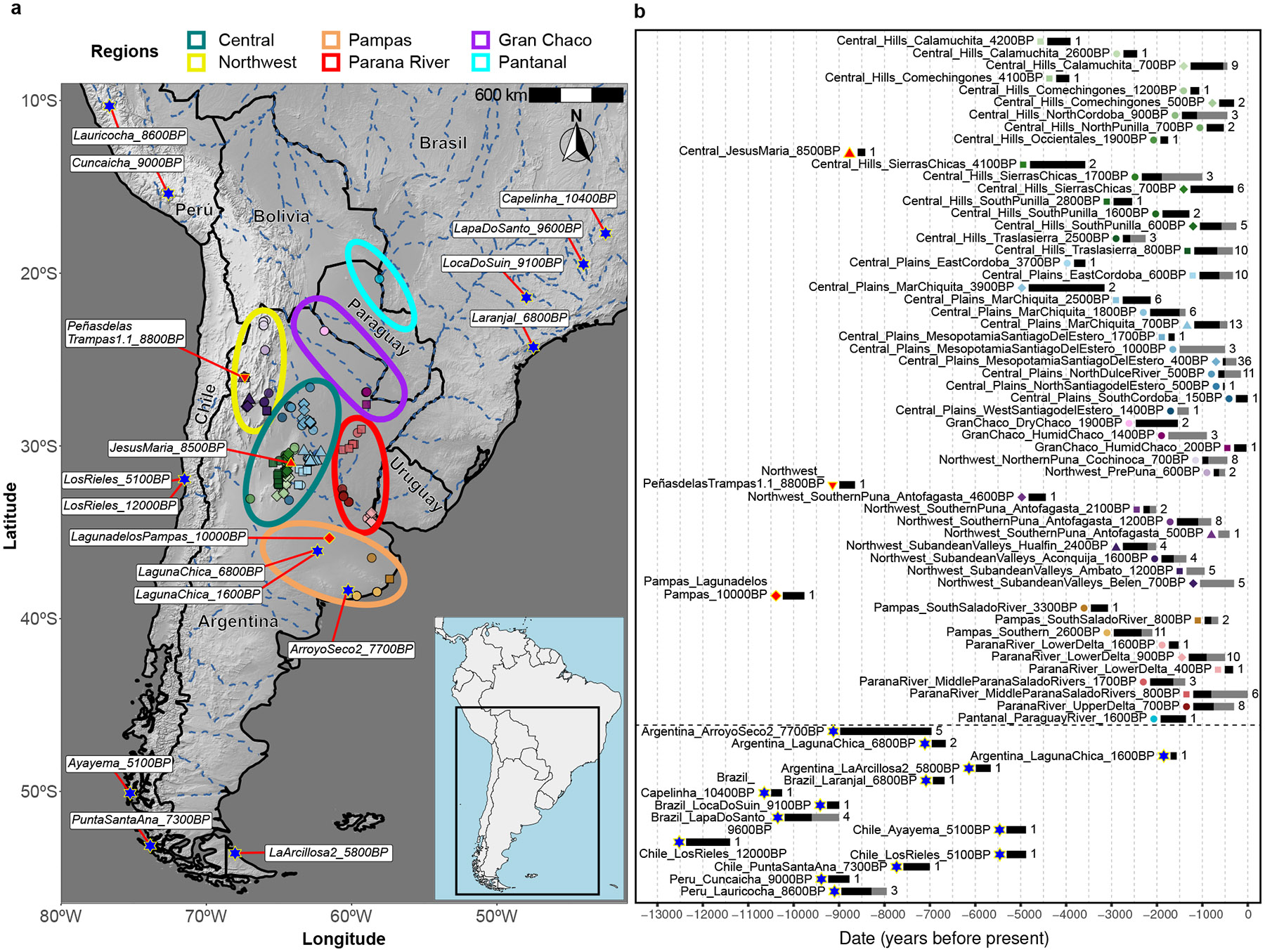
Overview of geographic and temporal sampling. **(a)** Geographic distribution of newly-reported and selected previously-published early South American ancient individuals. **(b)** Temporal distribution of newly-reported ancient individuals. For each grouping, the number to the right of the bar indicates the sample size, and the dark fill of the bar indicates the proportion with a direct radiocarbon date.

The peopling of South America likely followed both the Pacific and Atlantic coasts^1,2^. Genetic differentiation is detectable in ancient genomic data after 9000BP in at least three main clusters: Central Andes, Tropical/Subtropical Forest or Lowlands (including Amazonia), and Central Chile/Patagonia/Pampas^3,4^. However, current sampling has major gaps. We focus on the poorly sampled Central Southern Cone (CSC), the territory of Central and Northern Argentina comprising the Andean mountains in the West to the Eastern fluvial plains and Southern grassland plains. The CSC has diverse biogeographical regions that we divide for analysis into Northwest Argentina (Northern and Southern Puna, Pre-Puna, and Sub-Andean Valleys including Belén, Aconquija, Hualfín, and Ambato); Central Argentina (Hills including the Southern Pampean Hills of Córdoba and San Luis provinces (hereafter Córdoba Hills), and Plains which include the Laguna Mar Chiquita region, East Córdoba, and the Santiagueña Plains); Gran Chaco (Dry and Humid); Paraná River and the adjacent alluvial plains (Middle Paraná-Salado Rivers, Upper Delta, and Lower Delta); and Pampas (Central Pampean Dunefields, Southern Pampas (including Interserrana and Pampas South), and South Salado River). We also studied an ancient individual from Pantanal in present-day Paraguay (Figure 1a). Our sampling is influenced by the intensity of archaeological research and available samples, providing more resolution in some regions than in others.

The CSC has been inhabited since the late Pleistocene, and archaeological research documents multiple influences from the Central Andes and the Lowlands^5-8^. The earliest widely-accepted site is Arroyo Seco 2 (14000 BP; all dates calibrated in what follows), in the Pampas. From the late Pleistocene and Early Holocene (13000-8200BP), human presence is well documented in the Pampas, the Puna in Northwest Argentina, and the Córdoba Hills in the Central region^9^. From 13300-11200 BP, several sites from the Southern Cone are characterized by fishtail projectile points, whose wide distribution has been interpreted as a signal of a rapid migration across South America, paralleling inferences from ancient genomes^2,10,11^.

Humans expanded into a wider range of CSC environments in the Middle Holocene (8200-4200BP). Nevertheless, some areas such as the Gran Chaco, the Central Plains, and the Paraná River show less evidence of settlement in this period (Supplementary Information Sections 1-6). These changes occurred at a time of increased temperature known as the Mid-Holocene Hypsithermal^12^; however, the consequences of those environmental fluctuations varied across regions, which may help explain the uneven distribution of archaeological sites^13,14^. Around 4500BP, there was a transition away from hunting and gathering as the sole means of subsistence in the Puna and valleys of Northwest Argentina^15^.

In the Late Holocene (after 4200BP), the CSC harbored communities that ranged from sedentary agro-pastoralists in the Northwest who hunted, foraged, and exchanged goods from several ecoregions over long distances via llama caravans^16^; semi-sedentary horticulturists in the Córdoba Hills^17,18^, and in the Central Plains and Paraná River adapted to fluvial environments^19-21^; and nomadic hunter-gatherers in the Pampas and Gran Chaco^22-24^. Ethnographic records document wide cultural variation in the CSC at the time of European contact^25,26^: Comechingones (Hênîa and Kâmîare) in the Córdoba Hills; Sanavirones in the Laguna Mar Chiquita area; Diaguitas speaking Cacan in the Sub-Andean Valleys; Atacamas speaking Kunza in the Puna; Tonocotés in the Santiagueña Plains; Lules in northwest Santiago del Estero; Chaná-Timbú in the Middle Paraná-Salado shores and Paraná Delta; Guaraní groups speaking Tupí-Guaraní languages who likely arrived by around 700 BP in the Paraná Lower Delta^27^; Wichí speaking a Mataco-Mataguaya language in the southern Gran Chaco; and in the same area Mocovíes and Qom (Toba) speaking a Guaycurú language. The introduction of horses and cattle brought about profound changes in the economy and mobility of the Indigenous peoples of the Pampas and Patagonia^24^. Some scholars postulate that the Southern Pampas was previously inhabited by groups related to Chon-speaking Patagonian Tehuelches^28^. In the Northern Pampas, Querandí groups were mobile hunter-gatherers whose linguistic affiliation is unclear.

To characterize the genetic structure of the CSC in the Early Holocene, and to test for gene flow and demographic differences across subregions, we screened 344 bone or tooth samples from 310 individuals up to 10000BP. The Supplementary Information (SI) contains descriptions of Supplementary Data 1-14 providing details of these samples and the analyses performed. A single SI document includes Supplementary Figures 1-84 and a text presenting archaeological context (SI Sections 1-7) and genetic analyses (SI Sections 8-13).

**Extended Data Figure 1.**
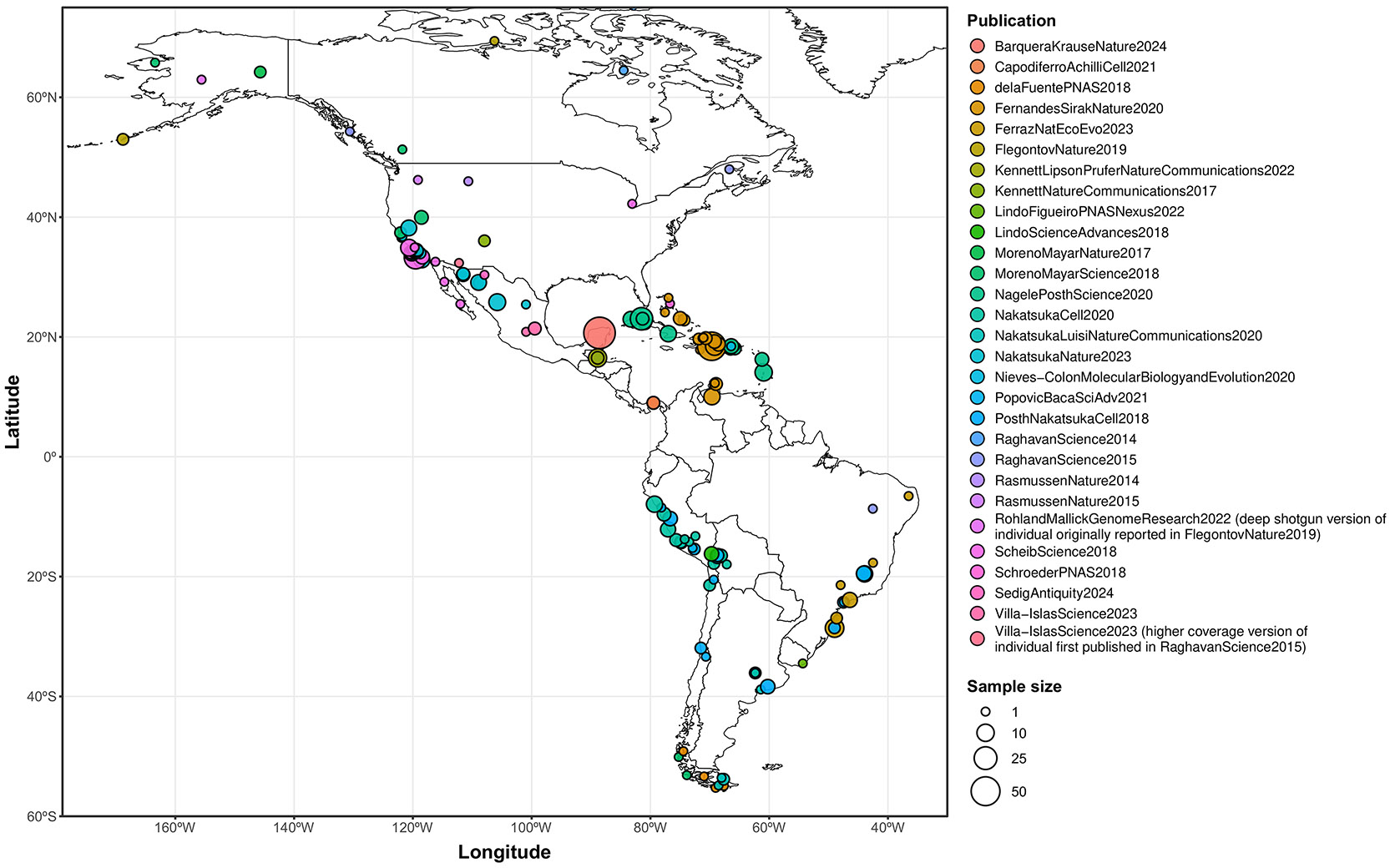
Geographical origin of previously-published individuals we analyze.

We enriched ancient DNA libraries for more than 1.2 million targeted single nucleotide polymorphisms (SNPs), and added to this off-target sites (not originally targeted by the enrichment protocol but commonly captured because of proximity to targeted SNPs) to arrive at a set of roughly 2 million analyzed SNPs (Methods). We obtained new genome-wide data passing quality control from 238 ancient individuals (Figures 1a, 1b), with a median of 659,011 SNPs covered at least once (207 individuals with at least 50,000 SNPs covered; Supplementary Data 1). We co-analyzed the newly-reported individuals with previously reported data for 588 pre-European contact Native/Indigenous Americans (Extended Data Figure 1, Supplementary Data 1), using the curation provided by the Allen Ancient DNA Resource (Methods). We defined “pre-European contact Native/Indigenous American individuals” as those with a mean date (a direct radiocarbon date or a contextual date) before 600BP. We also included SNP array data from present-day Native Americans^2^, restricting to sites intersecting the “1240k” set.

## Distinctive genetic drift by 10000BP

**Figure 2.**
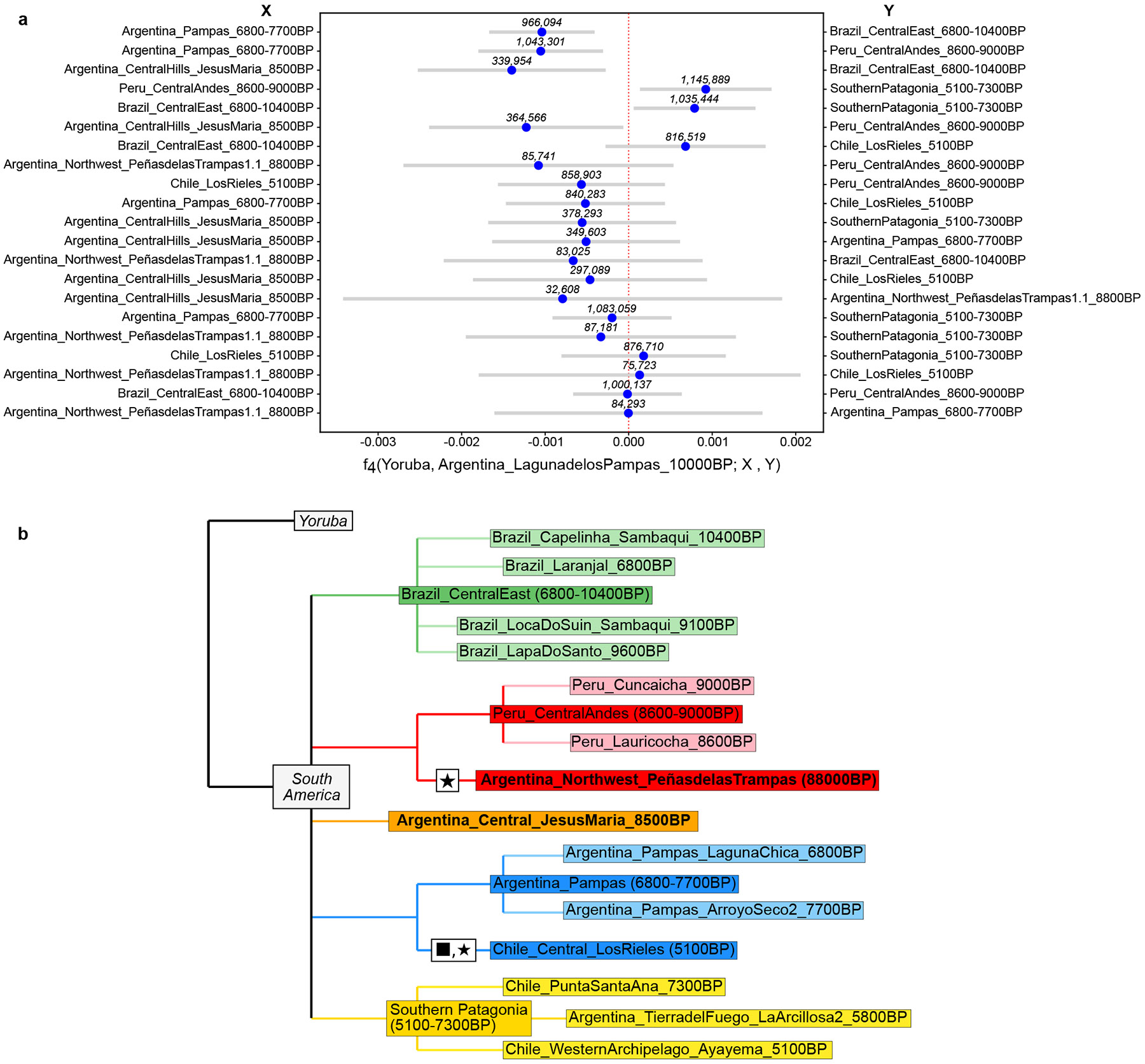
Relationships among deep South American lineages. **(a)** Affinities of *LagunadelosPampas_10000BP* to Early/Middle Holocene South Americans quantified by $f_{4}$ statistics. Bars denote 95% CIs (1.96× SE) around the mean across genomic-block jackknife pseudoreplicates ($f_{4}$ point estimates). The only significantly non-zero statistics (top 6) indicate excess allele-sharing with Middle Holocene Southern Cone individuals, with respect to both early individuals from the Central East of Brazil (10400-6800BP) and the Central Andes (9000-8600BP). At the same time, *LagunadelosPampas_10000BP* appears symmetrically related to all three of Southern Cone groupings up to the limits of our resolution. The number of SNPs used for each test is shown above each point estimate in the figure. **(b)** Distinct lineages in South America by the Middle Holocene. Clades were established using a combination of cladality tests and automatic exploration of population history models. We represent lineages for which we could not robustly favor a particular splitting order as a politomy. Newly-reported individuals are in bold, and thin evidence for some clades is indicated by ★. ■ indicates detected affinity for Mesoamerican-related populations. We found no evidence of mixture events fitting the data significantly better, although this could be a reflection of low statistical power. *LagunadelosPampas_10000BP* is absent from the tree because of its ambiguous positions across well-fitting models.

To understand how the oldest individual, *Argentina_Pampas_LagunadelosPampas_10000BP* (hence, *LagunadelosPampas_10000BP*) relates to other Early/Middle Holocene South Americans, we computed $f_{4}$-statistics of the form:

(1) $$f_{4}(\text{Outgroup},\text{Pop}1,\text{Pop}2,\text{Pop}3)$$

(Supplementary Data 2) which should not deviate significantly from zero if *Pop*2 and *Pop*3 are a true clade (descended without mixture from a common ancestral population) with respect to *Pop*1. A violation of this test—whose deviation from zero can be expressed as an approximately normally distributed *Z-*score computed using a genomic block jackknife—indicates a wrong phylogeny or a history that involves gene flow among the tested lineages. These statistics reveal shared drift among *LagunadelosPampas_10000BP* and *Argentina_Central_JesusMaria_8500BP* (henceforth, *JesusMaria_8500BP*), the individuals from Southern Patagonia (5100-7300BP) and those from the Argentinian Pampas (7700-6800BP), with respect to both early individuals from the Central-East of Brazil (10400-6800BP) and the Central Andes (9000-8600BP) (Figure 2a).

All pairs of *JesusMaria_8500BP*, Southern Patagonia (5100-7300BP), and Argentinian Pampas (7700-6800BP) are symmetrically related to *LagunadelosPampas_10000BP*, up to the limits of our resolution for statistics unaffected by biases due to using different sequencing technologies (Figure 2a) (SI Section 9, Supplementary Data 2). The most plausible explanation is that *LagunadelosPampas_10000BP* belonged to an ancestral Southern Cone population that split from Central East Brazil and Central Andes groups by 10000BP and was geographically in the CSC by that time before differentiating into distinct components. Neither *PeñasdelasTrampas1.1_8800BP*, from Southern Puna in Northwest Argentina, nor *LosRieles_5100BP* from Central Chile, showed affinity to *LagunadelosPampas_10000BP*, so we could not make a definitive statement about their relationship to this individual.

**Extended Data Figure 2.**
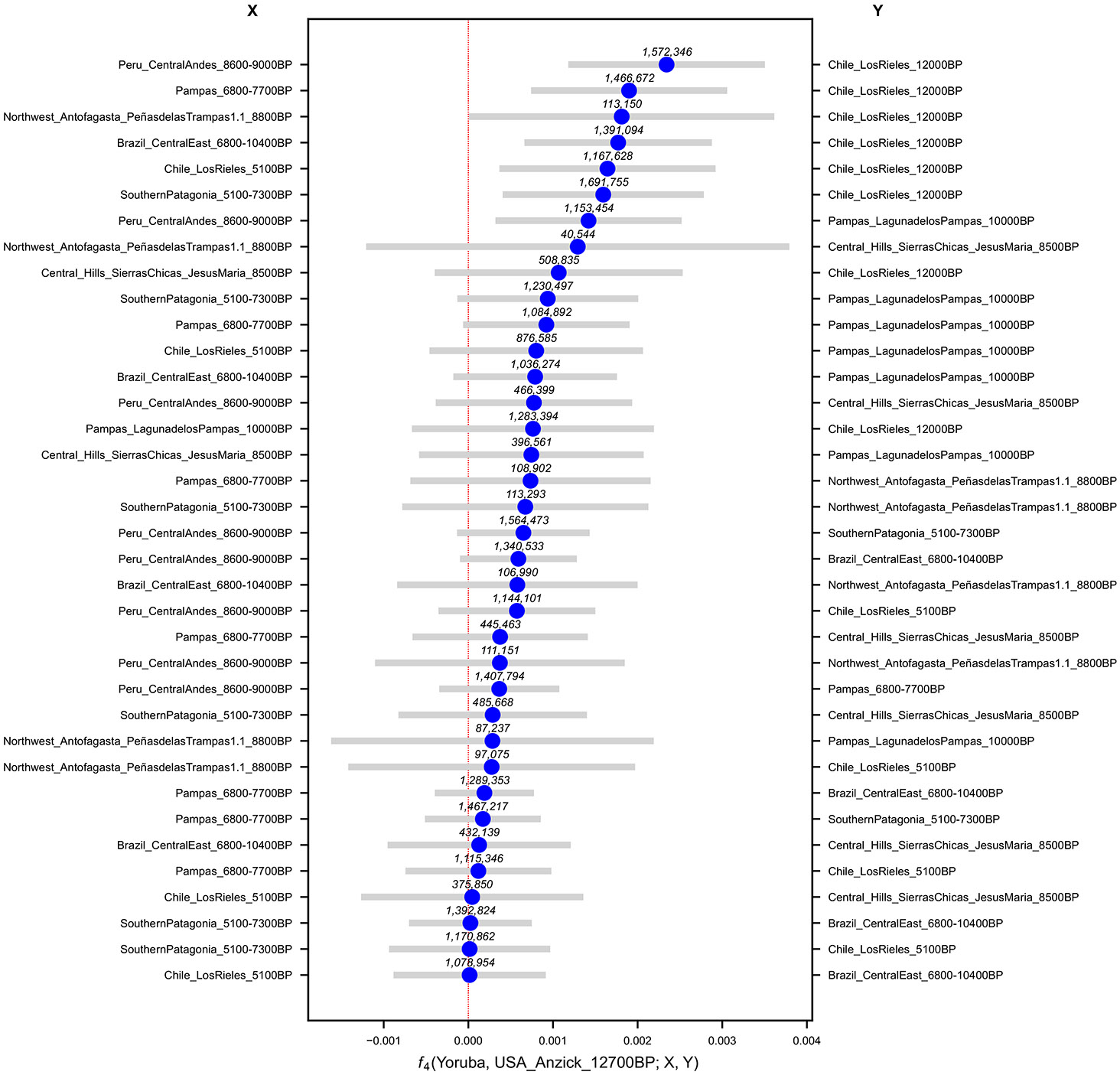
Affinities of Anzick to Early/Middle Holocene South Americans quantified by $f_{4}$ statistics. Bars denote 95% confidence intervals (1.96 × SE) around the mean across genomic-block jackknife pseudoreplicates (point estimate). The number of SNPs used for each test is shown above each point estimate in the figure.

We evaluated the affinities of *LagunadelosPampas_10000BP* to Anzick, a 12500BP individual from present-day Montana, USA, with distinctive genetic affinities to early South Americans relative to later ones^11^. *Chile_LosRieles_12000BP* showed the strongest affinity (*∣Z∣ <* 4.1), followed by weaker affinity with *LagunadelosPampas_10000BP* (*∣Z∣ <* 2.6) (Extended Data Figure 2, Supplementary Data 2). However, since these three individuals were positioned together as a clade in an outgroup*-f*_3_ neighbor-joining tree (Supplementary Figure 1), both probably harbored a distinct Anzick-related genetic component. Affinity with Anzick in early South America, and absence thereof, has been associated with at least two independent migration waves and population replacement^11^. However, the fact that *LagunadelosPampas_10000BP* also exhibits excess allele-sharing with later Southern Cone individuals without a significant genetic affinity towards Anzick, suggests that this individual may have been admixed between a basal Southern Cone lineage and a basal Anzick-associated lineage, and thus these Anzick-related lineages may not have been completely replaced^29^.

We re-examined several other claims of complex relationships between Central and South Americans, studying evidence of asymmetrical relatedness to Mesoamerican-related populations among late Middle Holocene individuals from Central Chile and the Central Andes (Supplementary Data 2)^11,30^. Using *qpAdm* (Methods), we modeled *Chile_LosRieles_5100BP* as a mixture of 16.2 ± 3.3% Mesoamerican-related and the rest *Brazil_LapaDoSanto_9600BP*-related (SI Section 9, Supplementary Data 3). But while asymmetrical relationships to Mesoamerican populations have been interpreted as evidence of a third ancestry movement into the subcontinent—in addition to the differential affinity to Anzick^11^—we cannot reject a simple two-source model of diverse early South American populations using *qpWave* (Methods) (*p >* 0.12) (Supplementary Data 2). This supports the theory that asymmetrical relatedness to Anzick may be better explained by a model of structure on a gradient than two independent pulses^29^, with the structured populations differentially related not only to Anzick but also to Mesoamericans.

Affinity between late Central Andes individuals and ancient Californians has been interpreted as evidence of a fourth migration pulse into South America^11^. However, late Central Andes individuals show stronger genetic affinity to ancient Caribbean individuals than to ancient Californians (Supplementary Data 2) when compared to early Central Andes individuals (*Z* = 6). Recent work has documented south-to-north migration in Central America^31^, and that California attraction is only detectable when considering Californian populations with Mexican-related gene flow^32^. Thus, the late Central Andes signal is plausibly driven by interactions within South America and back-migration spreading up to California.

## Three deep lineages in the Mid-Holocene

We combined published data with three individuals dated to before 8500BP: *LagunadelosPampas_10000BP* (Pampas), *PeñasdelasTrampas1.1_8800BP* (Northwest Argentina), and *JesusMaria_8500BP* (Central Argentina) (Figures 1a, 1b). Using $f_{4}$-statistics, we identified four possible clades of South American Early/Middle Holocene individuals: Brazil, Central Andes, Pampas, and Southern Patagonia (Figure 2b, SI Section 9) ^3,11,29,30,33,34^.

We merged these putative clades into common labels and combined them with remaining individuals that were not identified as part of any clade for automatic population history model exploration. We used the *find graphs* function of ADMIXTOOLS2, which evaluates randomly-perturbed admixture graphs until the resulting graph cannot be made to better fit the data. Because this search gets trapped in local optima, we performed 100 independent iterations, each starting from a randomly-initialized graph, to explore the diversity of equally well-fitting models. We found no evidence that models involving admixture events fit the data significantly better than ones without mixture (SI Section 9, Supplementary Data 2), and hence we examined only the nine unique best-fitting models with no admixture (Supplementary Data 2, Supplementary Figures 2-10 (range of scores: 34.1-43.3, worst residuals: 2.9-4.8)). For all these models, many internal branches had a drift value of either 0, indicating an inability to discern the order of splits, or 1-2, indicating weak support for a branch.

All nine models include a clade with *PeñasdelasTrampas1.1_8800BP* and Central Andes (9000-8600BP). Eight of the nine support a clade of *Chile_LosRieles_5100BP* and Middle Holocene Argentinian Pampas (7700-6800BP), with the exception of the worst-fitting one (Supplementary Figure 8). These clades were not rejected by $f_{4}$-statistics (Supplementary Data 2). These two clades are not confident due to the low inferred drift ancestral to them (1), although *PeñasdelasTrampas1.1_8800BP* grouping with the Central Andes cluster agrees with an outgroup*-f*_3_ tree (Supplementary Figure 1). The placement of *LagunadelosPampas_10000BP* was more ambiguous, appearing as an isolated lineage (3 models) or grouped with the Central Argentina *JesusMaria_8500BP* (5 models), or the Middle Holocene Argentinian Pampas (7700-6800BP) (1 model), consistent with its basal position in CSC diversity.

Our results indicate that the CSC harbored at least three deep lineages: a lineage represented by *PeñasdelasTrampas1.1_8800BP* that appears cladal with the main ancestry component present in the Central Andes since 9000BP^11,33^; a lineage occupying the Pampas in the Middle Holocene^11^, whose earliest representatives are *ArroyoSeco2 7700BP*; and a Central Argentina lineage, whose earliest sampled individual is *JesusMaria_8500BP* (Figure 2b; see also SI Section 9).

## Ancestry landscape of the Late Holocene

**Figure 3.**
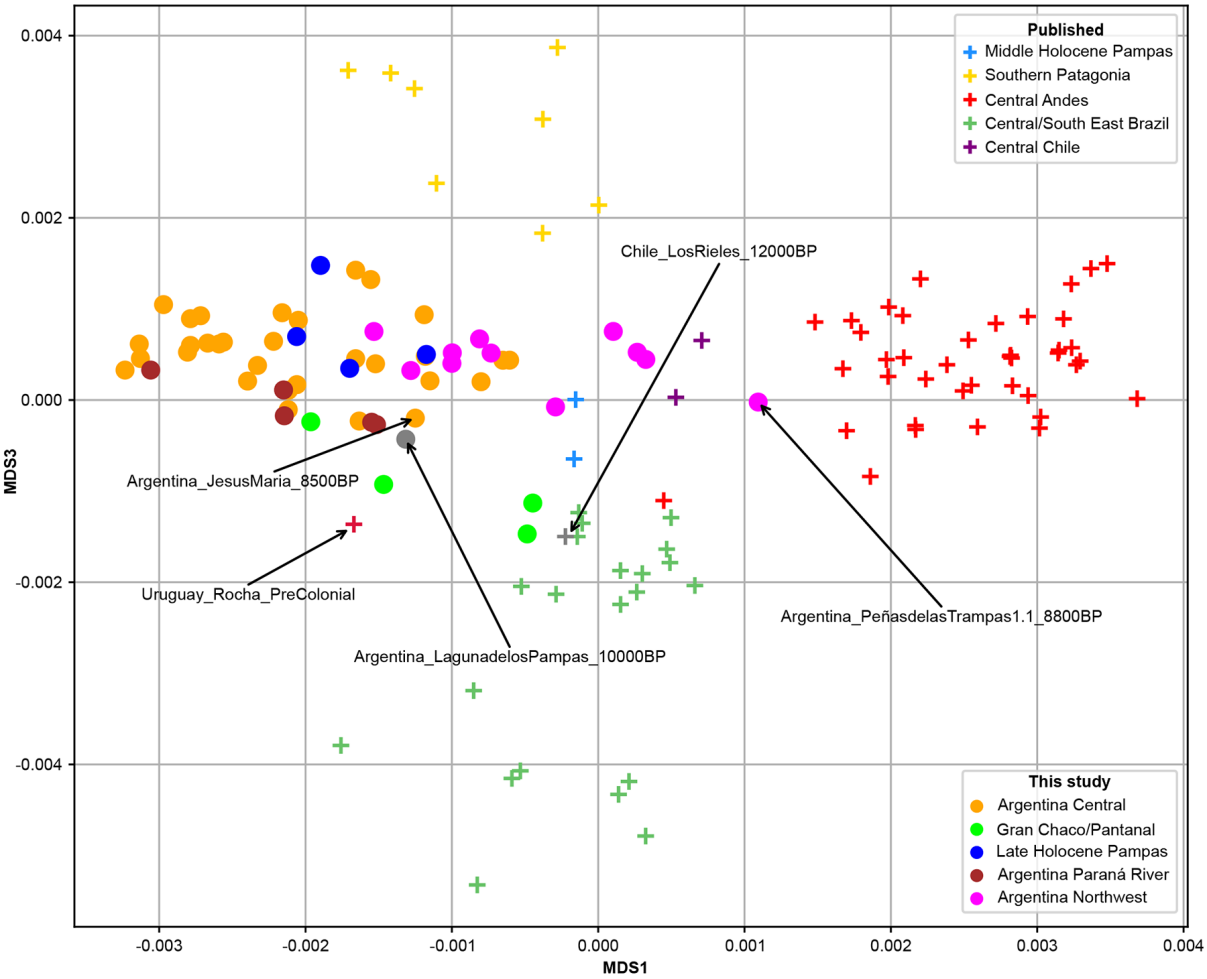
MDS1× MDS3 plot of an outgroup-*f*_*3*_ distance matrix of the form 1/*f*_*3*_(Pop1, Pop2; Yoruba), where Pop_i_, i ∈ {1, 2}, is a newly-reported or previously-published ancient American context label from present-day Argentina, Chile, Brazil, Uruguay, Peru, Bolivia or Paraguay. We found this more informative than plotting the first against the second component, because in that case Patagonian populations appeared interspersed with Brazilian populations. Populations sampled in present-day USA, Mexico, Belize, Venezuela and the Caribbean were removed from the plot, as they appeared very distant to the newly-reported populations (see also Supplementary Figure 11). We caution against over-interpreting the position of the oldest individuals, like *LosRieles_12000BP*, who may simply lack much shared drift with the rest.

We computed outgroup-*f*_3_ statistics, measuring shared drift between pairs of populations up to the split from a common ancestor; we use the inverse as a measure of genetic distance. Dimensionality reduction techniques like multidimensional scaling (MDS), developed for distance-based settings, are useful for visualizing affinities. Figure 3 shows the first and the third component of this MDS analysis (also Supplementary Figure 11), in which most new samples form a cluster including the oldest Central Argentinian, *JesusMaria_8500BP*. The horizontal axis differentiates Central Andes (right) from Central Argentina (left); vertical Southern Patagonia (top) from Central East Brazil (bottom). A neighbor-joining tree produces similar patterns (Supplementary Figure 1).

**Extended Data Figure 3.**
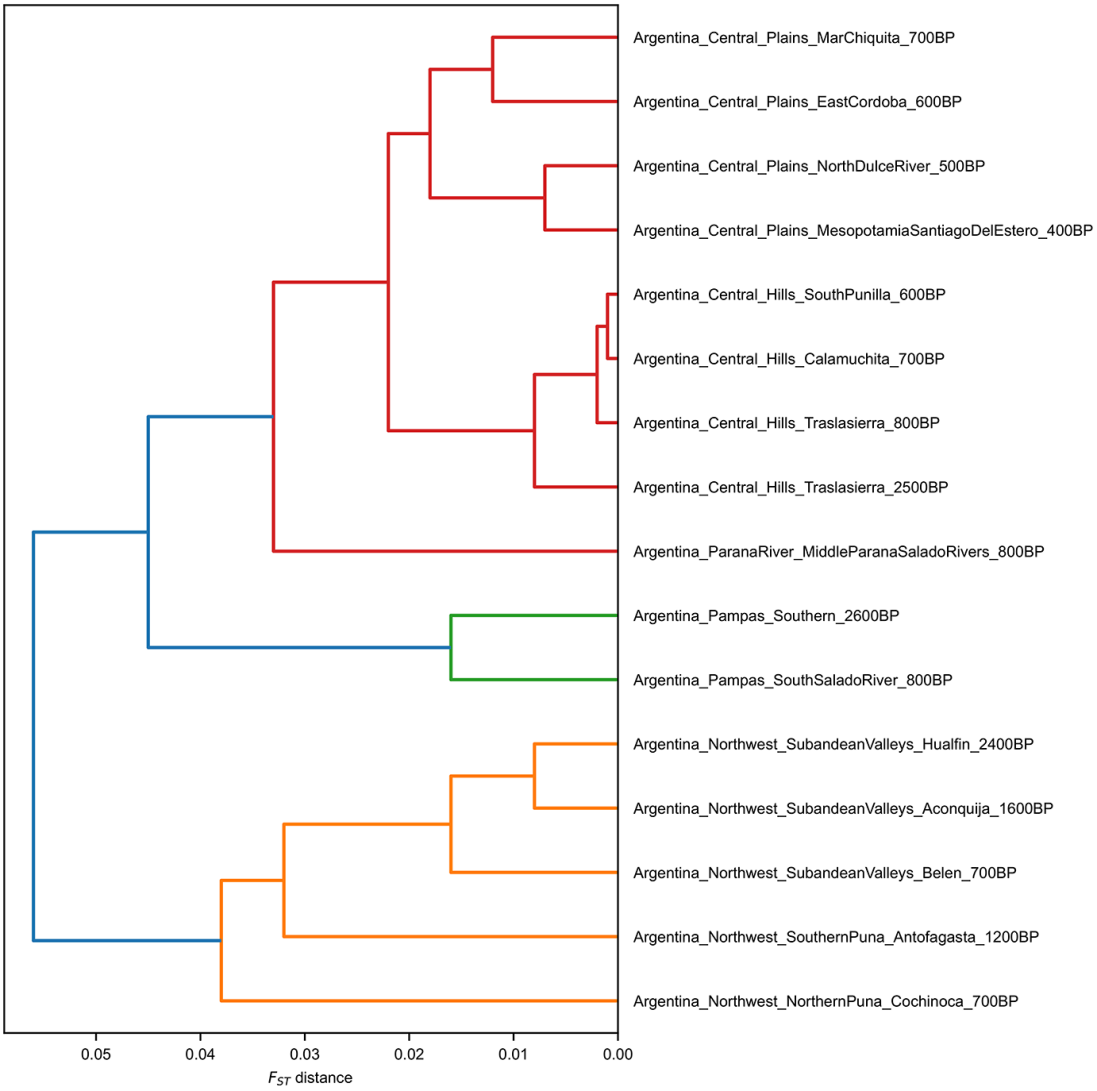
Complete hierarchical-clustering tree from *F*_*st*_ distances, restricted to populations for which at least 5000 SNPs were used for all pairwise computations. Colors represent automatically-inferred clusters.

Late Holocene populations from the Northwest are shifted toward Central Andes groups, hinting at admixture. In the neighbor joining tree, the 700-600BP individuals from Northern Puna and Pre-Puna fall in the Central Andes cluster, closest to Bolivian populations. Individuals from the Gran Chaco and Paraguay Pantanal regions shift towards or fall within the cluster of Central East Brazilian populations, but not so the 200BP Gran Chaco individual, who clusters with Central Argentina. A 400BP individual with a Guaraní archaeological association also appears in this cluster, likely reflecting the Guaraní expansion^27^, but data are too sparse for ancestry component modeling (Supplementary Data 1). All remaining samples clustered, with imperfect but consistent separation between Pampas, Northwest, Paraná River, and Central Argentina individuals, mirroring *F*_*st*_ hierarchical clustering (Extended Data Figure 3 and Supplementary Figure 12).

To test for genetic affinities, we computed $f_{4}$(Outgroup*, P*1; *P*2*, P*3), where *P*2 are early Middle Holocene South Americans, *P*3 groups from the study subregion, and *P*1 all other ancient groups (Supplementary Data 4). The great majority of CSC individuals show affinity to Southern Patagonia, Central Andes, and Middle Holocene Pampas compared to Central-East Brazil, implying that Brazil is likely the deepest split (Supplementary Data 4). Applying a false-discovery rate (FDR) for clade rejection at FDR*<* 0.05 using the Benjamini-Yekutiel procedure (Methods) *Z*_*BY*_), we highlight six observations (Supplementary Data 4, SI Section 11). First, Northern Puna and pre-Puna individuals shared alleles at an excess rate with people of the Central Andes (2.9 *< Z*_*BY*_ *<* 5), and other Northwest Argentina groups have evidence of admixture between Central Argentina and Central Andes sources (Table 1). Second, Late Holocene individuals from Central Argentina attract others from the same region (3 *< Z*_*BY*_ *<* 27.1) and are a clade with *JesusMaria_8500BP*, except for excess sharing with Mexicans and ancient Californians (3 *< Z*_*BY*_ *<* 3.6) in *Argentina_Central_Hills_Calamuchita_4200BP* and later, but with no evidence for an increasing trend with time (Supplementary Data 5). This points to a demographic process connecting lower North America all the way to the CSC; while we do not have sufficient sampling from 8500-4200BP to identify the likely sources, it is plausibly the same process induced Mesoamerican affinity in *Chile_LosRieles_5100BP*. Third, the Late Holocene individuals from the Paraná River region shared drift with Central Argentina (3 *< Z*_*BY*_ *<* 16.3). Fourth, individuals from the Gran Chaco including the 1400BP individual from the El Cachapé complex share alleles with modern groups from the same region like Chané, Wichí, Guaraní or Toba (3 *< Z*_*BY*_ *<* 6.9); the Paraguay Pantanal individual at 1600BP shows a similar signal despite separation by >800 km, supporting a “Chaco-Pantanal” archaeological connection^35^. Fifth, modern Gran Chaco populations are admixed between a Central Argentina and a Tropical/Subtropical Forest source (Table 1). Sixth, individuals from the Pampas share drift both with other from the same region (3 *< Z*_*BY*_ *<* 15.4), and with Central Argentina compared to the Middle Holocene Pampas (3 *< Z*_*BY*_ *<* 9.8), with direct evidence of admixture in Late Holocene Pampas (*Southern_2600BP* and *LagunaChica_1600BP*) (Table 1).

**Figure 4.**
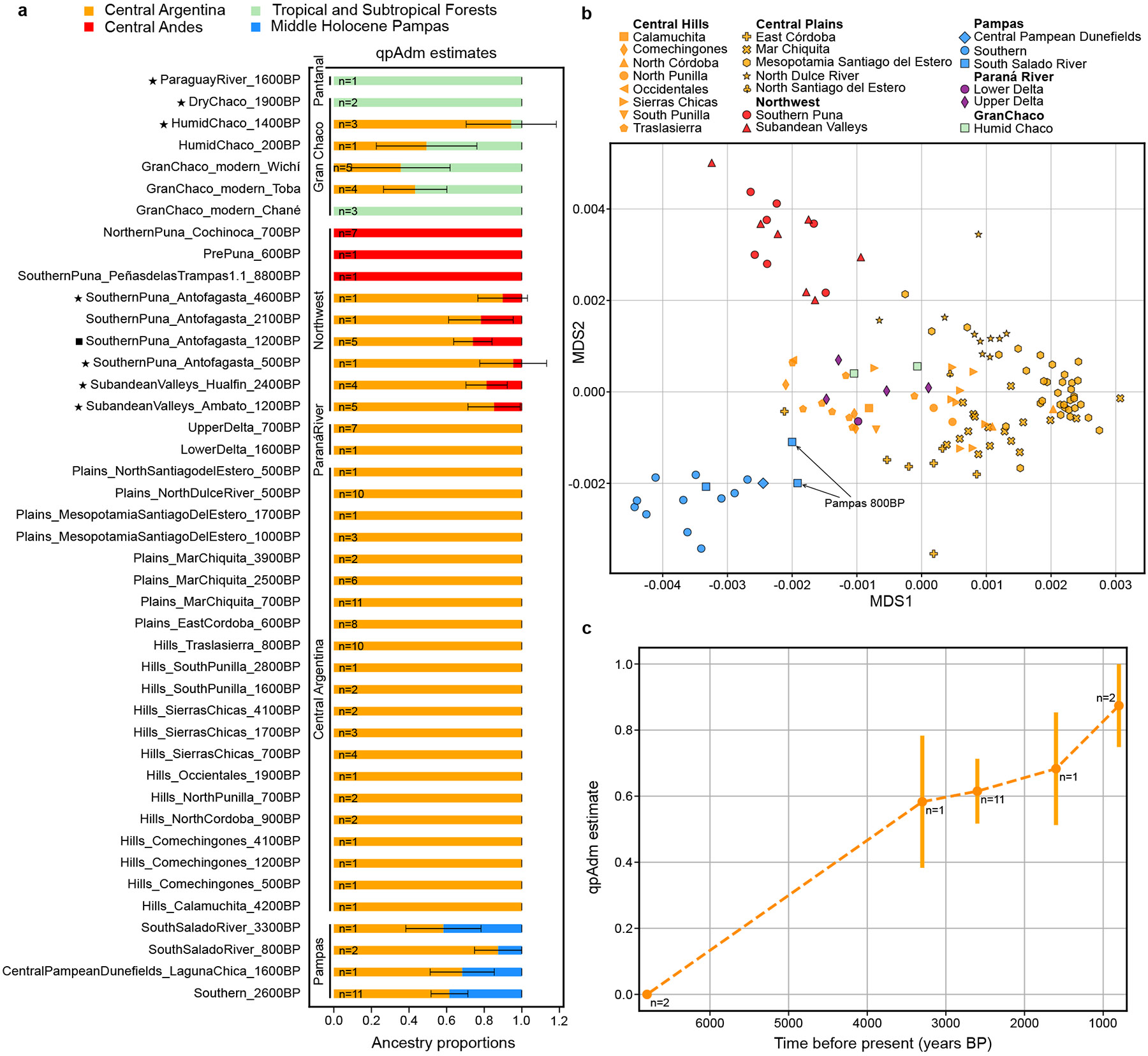
Ancestry modeling and fine-scale structure within the Central Southern Cone reveal three distinct admixture processes. **(a)** *qpAdm* ancestry component estimates for selected groupings. Bars denote 95% confidence intervals (1.96 × standard error) around the mean across genomic-block jackknife pseudoreplicates (point estimates). ★ indicates instances in which a Central Argentina-only model was also found to fit the flagged grouping label. ■ indicates instances in which a Central Andes-only model was also found to fit the flagged label (see SI Section 12 for details). Inferences for Gran Chaco and Pantanal were more ambiguous due to low sample sizes and coverages. The number of individuals within each grouping is shown within each horizontal bar in the figure. **(b)** MDS1×MDS2 plot of a distance matrix of the form 1/*f*_*3*_(I_1_, I_2_; Yoruba), where I_i_, i ∈ {1, 2} is an individual from a context label estimated to carry primarily Central-Argentina ancestry. This low-dimensional decomposition revealed two axes of variation, which can be interpreted, in light of the *qpAdm* results (Figure 4a), as resulting from admixture between three poles of ancestry: Central Argentina, Central Andes, and Middle Holocene Pampas. Overall, we observe geographically-driven clustering maintained over thousands of years. **(c)** *qpAdm* estimates of Central-Argentina ancestry in the Pampas region over time. Bars denote 95% confidence intervals (1.96 × standard error) around the mean across genomic-block jackknife pseudoreplicates (point estimates). The 6800BP data point corresponds to individuals from the *LagunaChica* site, who appear to be a clade with the 7700BP *ArroyoSeco* individuals (Middle Holocene Pampas). Central Argentina-ancestry in the Pampas increased (two-sided *p* = 0.0014 from a Z-test for a significant difference in Central-Argentina ancestry proportions in *SouthSaladoRiver_800BP* with respect to *Southern_2600BP*). This suggests multiple waves of admixture or continuous gene flow from Central Argentina into the Pampas. The number of individuals within each grouping is shown next to each point estimate in the figure.

To quantify admixture, we used *qpAdm* (Methods) (Figure 4a, Supplementary Data 6-12). We asked what groups were consistent with being simple clades or two-way mixtures of the relevant deep South American lineages (Central Argentina, Central Andes, Middle Holocene Pampas and Tropical/Sub-tropical Forest), cyclically assessing models with respect to the other sources and more distant outgroups, and adding complexity to failing single-source models if needed (SI Section 12). ADMIXTURE results were less informative, but shared some broad patterns with the *qpAdm* conclusions (see SI Section 13, Supp. Figures 13-17 for details).

## Fine-structure in Central Argentina

**Extended Data Figure 4.**
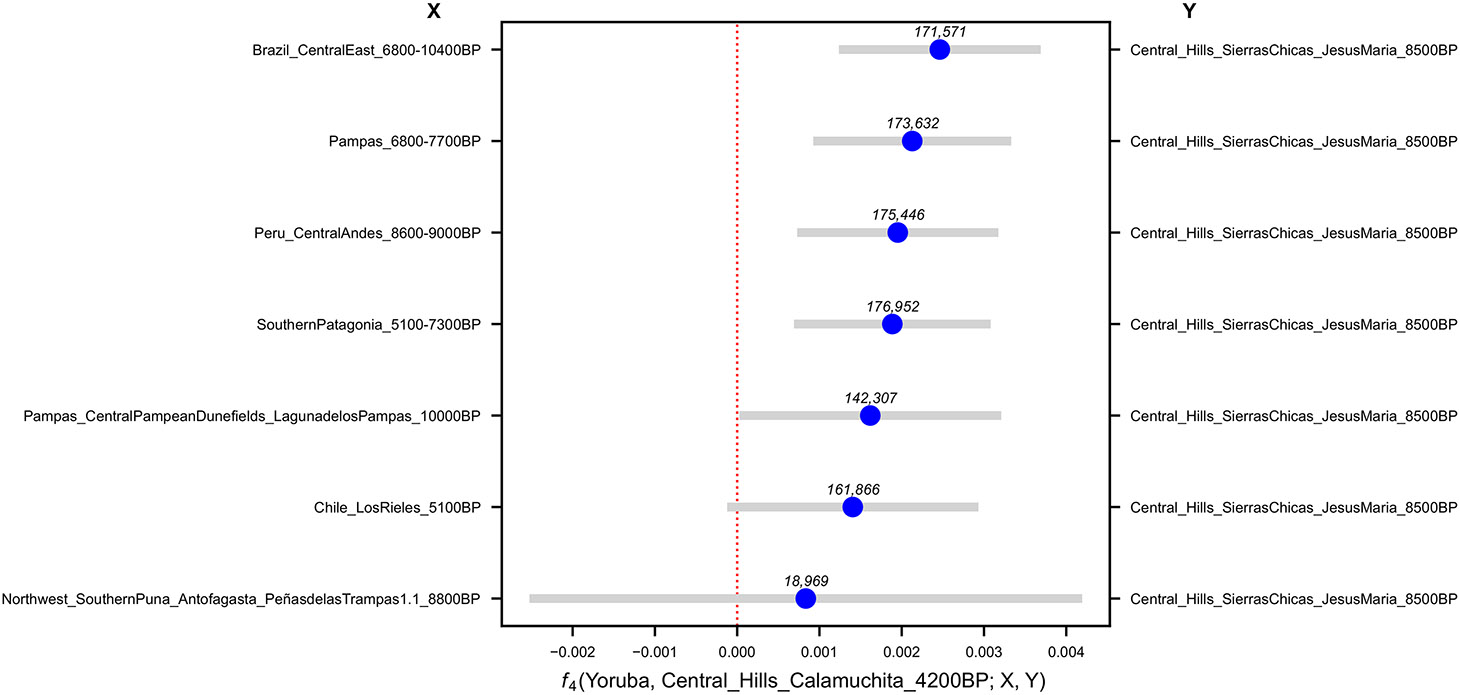
Affinities of a representative 4200BP Central Argentina population to Early/Middle Holocene South American samples quantified by $f_{4}$ statistics. Bars are 95% confidence intervals (1.96 × SE) around the mean across genomic-block jackknife pseudoreplicates. (point estimate). The number of SNPs used for each test is shown above each point estimate in the figure.

**Extended Data Figure 5.**
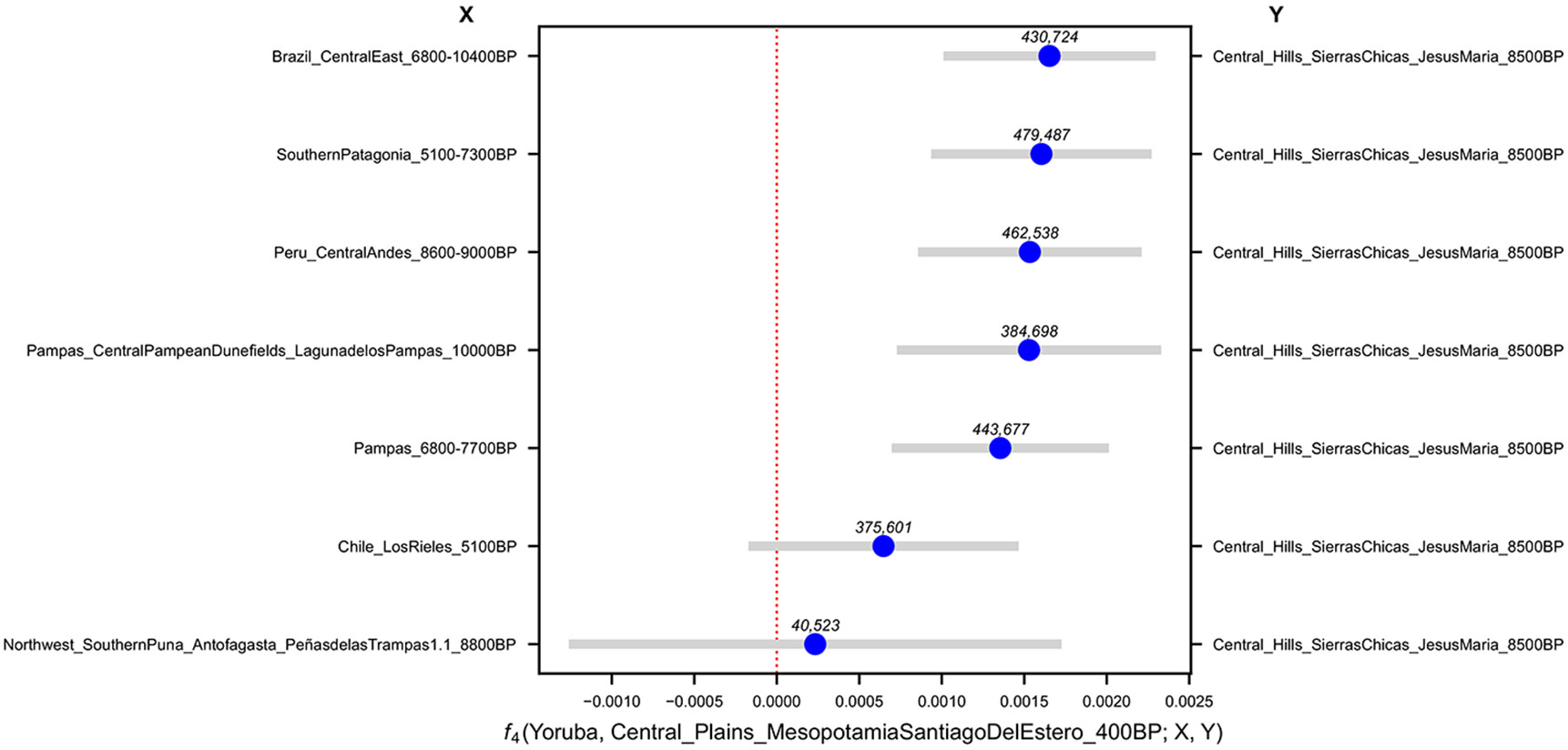
Affinities of a representative 400BP Central Argentina population to Early/Middle Holocene South Americans quantified by $f_{4}$ statistics. Bars are 95% confidence intervals (1.96 × SE) around the mean across genomic-block jackknife pseudoreplicates (point estimate). The number of SNPs used for each test is shown above each point estimate in the figure.

**Extended Data Figure 6.**
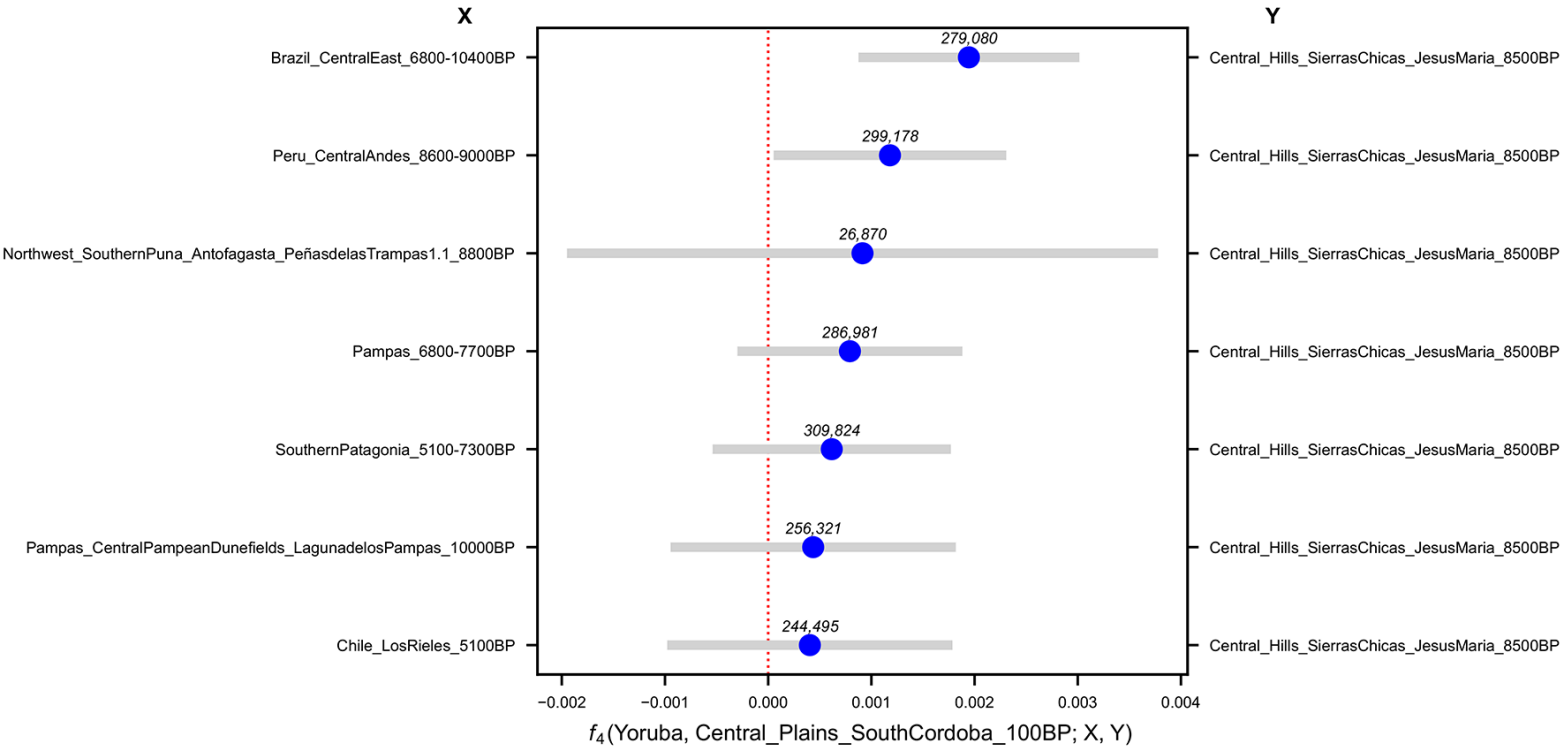
Affinities of a representative 150BP Central Argentina population to Early/Middle Holocene South Americans quantified by $f_{4}$ statistics. Bars are 95% confidence intervals (1.96 × SE) around the mean across genomic-block jackknife pseudoreplicates (point estimate). The number of SNPs used for each test is shown above each point estimate in the figure.

**Extended Data Figure 7.**
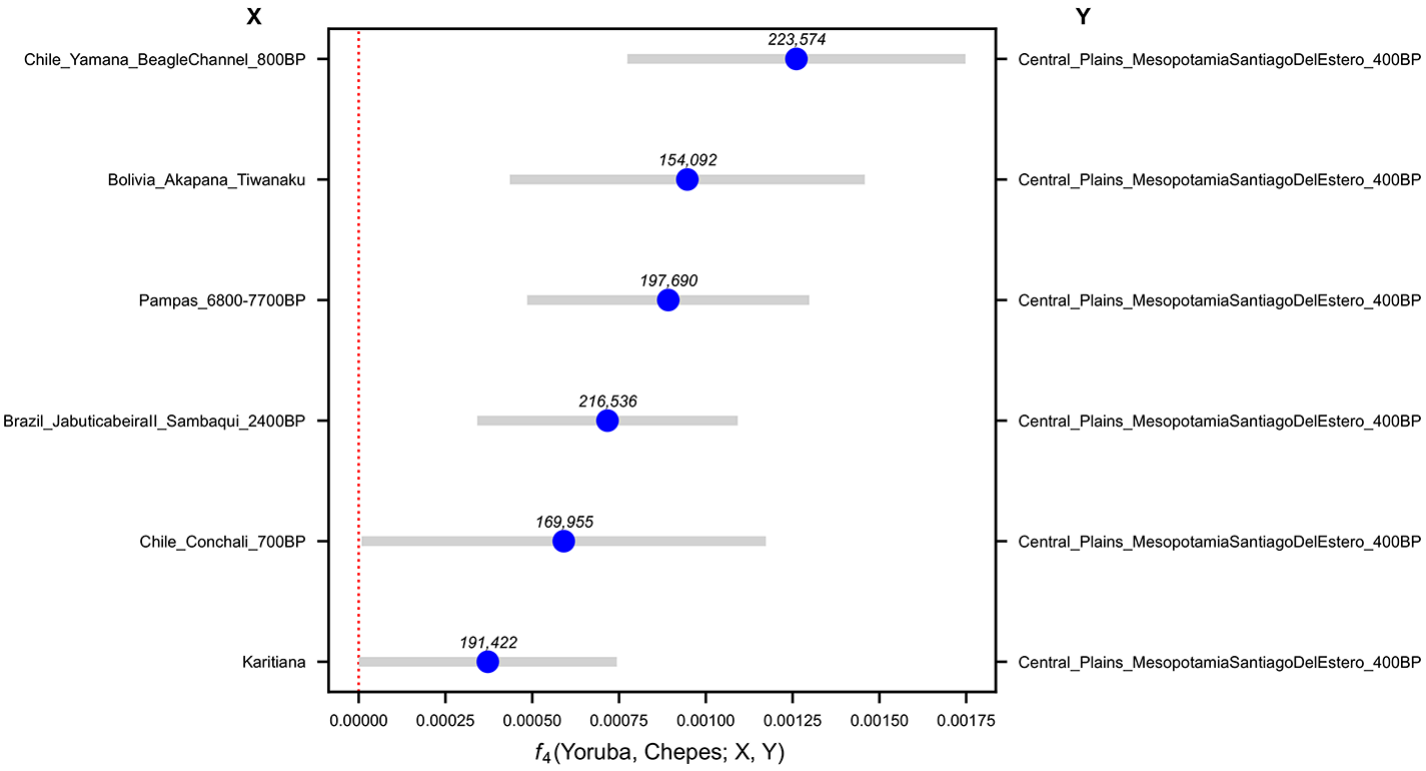
Affinities of a modern Central Argentina admixed population^4^ to Late Holocene South Americans quantified by $f_{4}$ statistics. Bars are 95% confidence intervals (1.96 × SE) around the mean across genomic-block jackknife pseudoreplicates (point estimate). The number of SNPs used for each test is shown above each point estimate in the figure.

We compared genetic affinity of selected Late Holocene Central Argentina populations from 4200-150BP, to the earliest Central Argentina individual *JesusMaria_8500BP*, with respect to other Early and Middle Holocene South Americans. $f_{4}$-statistics are positively skewed, showing excess allele sharing with *JesusMaria_8500BP* (Extended Data Figures 4 to 6, Supplementary Figures 18 to 24) (*Z <* 5.54). Most individuals from Central Argentina were consistent with being genetically homogeneous (Supplementary Figure 81), suggesting continuity in Central Argentina going back more than eight millennia, and persisting until at least 150BP. This extends previous findings based on ancient mitochondrial DNA which detected deep, locally-specific mtDNA clades in Central Argentina^36^. When we analyzed modern admixed Central Argentinian individuals^4^, we found the same pattern of $f_{4}$-statistic skew towards Late Holocene Central Argentina individuals (Extended Data Figure 7) (although Early/Middle Holocene comparisons were under-powered due to the small overlap between the SNP sets; Supplementary Figures 25 to 70), suggesting that the ancestry component represented by *JesusMaria_8500BP* is the main Native American lineage in the region up to the present day. However, modern individuals that previous work^4^ labeled as belonging to the “Central Western Argentina” lineage (Calingasta and Río Grande) actually appear genetically closest to ancient individuals from Central Chile, Middle Holocene Pampas and Southern Patagonia (Supplementary Figures 34 and 55), and thus are not reflecting the deep lineage represented by *JesusMaria_8500BP* that we characterize here.

To obtain a fine-grained picture of the evolution of the Central Argentina lineage, we computed an outgroup-*f*_3_ distance matrix between all pairs of individuals from groupings that were inferred to carry majority Central Argentina-type ancestry (Figure 4a). We find two axes of variation in Figure 4b resulting from admixture of the three ancestry poles Central Argentina, Central Andes, and Middle Holocene Pampas. The persistence of these clines for thousands of years with no individuals clustering outside their region suggests isolation by distance, undisrupted by further pulses of cross-regional migration.

We also observed a separation between the Córdoba Hills and the Central Plains where we have particularly dense sampling, indicating geographic substructure even at this fine level as also seen in mitochondrial DNA^37^. This is consistent with distinct material culture, diet, physical activity, and mortuary practices over the last two millennia between groups that inhabited the Córdoba Hills and the Laguna Mar Chiquita region^21^.

## Interactions with Central Argentina

**Extended Data Figure 8.**
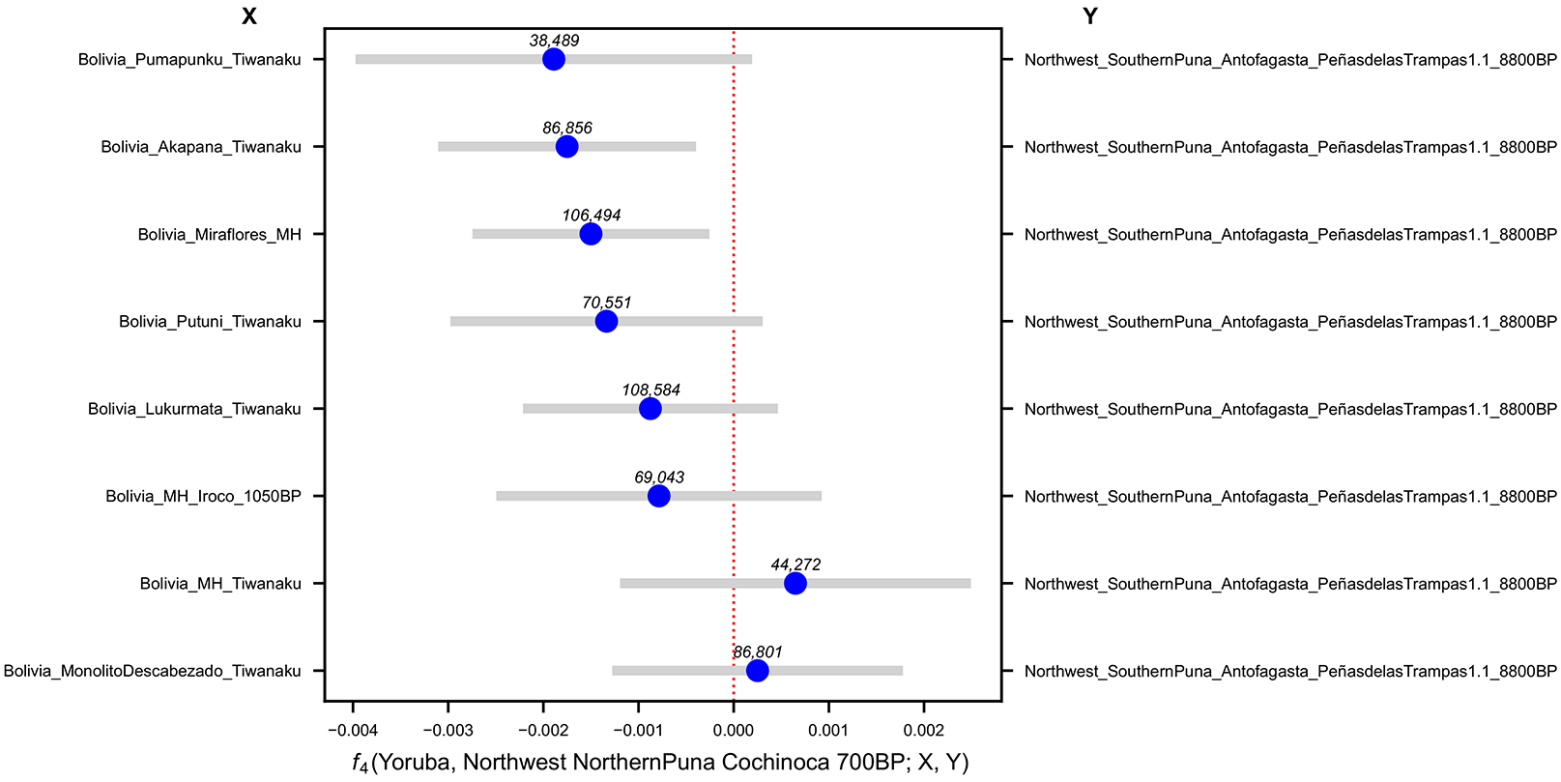
Affinities of *Northwest_NorthernPuna_Cochinoca_700BP* to Late Holocene Bolivians quantified by $f_{4}$ statistics. Bars are 95% confidence intervals (1.96 × SE) around the mean across genomic-block jackknife pseudoreplicates (point estimate). The number of SNPs used for each test is shown above each point estimate in the figure.

Northwest Argentina people (Northern Puna and Pre-Puna) in the last millennium were genetically indistinguishable from Central Andes individuals. But other Northwest groups showed a mostly Central Argentina background (Figure 4a). Northern Puna individuals shared more alleles with Late Holocene groups from Bolivia than with *PeñasdelasTrampas1.1_8800BP* (Extended Data Figure 8). Thus, while Central Andes ancestry in Northwestern Argentina has a deep history, interactions with the southern Central Andes continued. The evidence of Central Argentina ancestry in the Northwest is paralleled by archaeological evidence linking peoples in Puna, Sub-Andean Valleys, and Santiagueña Plains^38^ (SI Sections 2,6). The male *Northwest_SubandeanValleys_Belen ElShincaldeQuimivil_500BP*, buried within an Inca site, had artifacts indicating a potential Central Andean origin, which were interpreted as evidence of relocation in his lifetime under the *mitmaqkuna* Inca practice^39^. However, his genetic background is not significantly different from that of other Sub-Andean Valley individuals from the same grouping (Supplementary Data 13), so there is in fact no genetic evidence that this individual was a migrant.

Gran Chaco and Pantanal history could only be explored roughly with our data due to low sample sizes and poor data quality. However, *f*_3_-based analyses cluster them with Brazilian groups, so they are unlikely to have had Central Argentina-type ancestry alone. For Gran Chaco individuals dating to 200BP (*HumidChaco_ElChancho_200BP*, clustering with Central Argentina in an outgroup *f*_3_-tree) or later (including present-day Toba and Wichí^2^), the only robust model supports a mixture of Central Argentina and Amazonian-related sources (Table 1, Figure 4a). Indeed, most ancient individuals from the Gran Chaco showed significant affinity to modern counterparts, indicating some continuity over two millennnia (Supplementary Data 4). The major exception was the Chané, who belong to the Arawak linguistic family and are thought to have migrated more recently to the Gran Chaco and mixed with Chiriguanos (Guaraní ethnolinguistic group), and had no evidence of Central Argentina-type ancestry.

Paraná River region individuals showed affinity with Central Argentina in $f_{4}$-statistics. In fact, most analyses were consistent with their being simple clades with Central Argentina, and failures of this clade test were plausibly due to data artifacts (SI Section 12, Supplementary Data 4, Supplementary Figure 82). This finding aligns with archaeological links between the Paraná River region, the Córdoba Hills and the Laguna Mar Chiquita (Central Plains) populations in the Late Holocene^7,20,21^; other archaeological evidence links the Middle Paraná-Salado Rivers and Santiagueña plains^40,41^. Some Paraná River individuals were associated with the Goya-Malabrigo archaeological complex, characterized by zoomorphic appendages in pottery, earth mound construction, and a riverine horticulture subsistence strategy^42^. It has been hypothesized that these traits are a signal of Arawak ethnolinguistic groups spreading along eastern South American rivers^43^. We explored this possibility by comparing the newly reported data with the limited Arawak-related data currently available, that is, both ancient Arawak-associated people from the Ceramic-period Caribbean and modern representatives (Piapoco from northern South America and the geographically closer Chané from Gran Chaco). Since we did not find any genetic signal of a specific affinity (Supplementary Data 13), our results do not provide evidence of a large-scale Arawak migration. Arawak influence in the Paraná River region could have been mediated by a small number of individuals, or by cultural transmission^42^. Alternatively, a large-scale migration could have occurred, and the absence of the Arawak signal in the Paraná groups could reflect incomplete representation of genomic diversity of Arawak-speaking groups among available samples.

Pampas region individuals from around 6800BP do not show affinity with the Central Argentina lineage when compared to 7700BP Pampas individuals from Arroyo Seco 2 (Supplementary Data 4). Thus, the Arroyo Seco 2 lineage persisted in the region for at least a thousand years without detected interaction with the neighboring Central Argentina lineage. However, Late Holocene Pampas individuals cannot be modelled as a simple clade with the Middle Holocene Pampas or Middle Holocene Central Argentina lineages (Table 1, Supplementary Figure 80). By 3300BP, Pampas individuals fit as a mixture of the Middle Holocene Central Argentina (58 ± 10%, Figure 4c, Supplementary Data 12) and Middle Holocene Pampas lineages. Due to limited sampling, we can only place a lower bound on the beginning of this southward spread of Central Argentinian ancestry at 3300BP; we attempted to estimate a date for this mixture (Methods) but it was too noisy. Central Argentina ancestry in the Pampas also continued to increase after 3300BP (Figure 4c, P=0.0014 from a *Z-*test in *SouthSaladoRiver_800BP* vs. *Southern_2600BP*), likely reflecting further gene flow from Central Argentina into the Pampas. A previous analysis of a 1600BP sample from the Laguna Chica site^33^ found excess allele-sharing between this individual and Central Andes populations relative to 6800BP Pampas individuals from the same site, which was interpreted as evidence of Central Andes-related gene flow^33^ (SI Section 12). However, this was a misinterpretation, and instead these findings are driven by the then-unsampled Central-Argentina lineage. The migrations into the Pampas that we detect are consistent with the observed differentiation between mitochondrial clades from Early/Middle^44^ and Late Holocene^45^ individuals. Archaeological evidence indicates an increase in population density in the Pampas around 3500BP^46^, along with the introduction of ceramics and the bow and arrow^24^. Nevertheless, other archaeological connections between these regions are sparse, including evidence of lithic raw material from Southern Pampas found in the south of Córdoba province^47^, as well as copper necklace beads found in the Pampas^23^, potentially sourced from Central Argentina.

## Kinship and community sizes

We analysed the distribution of runs of homozygosity (ROH) in individuals with sufficient data using hapROH (Methods) and used these distributions to estimate effective community sizes (*Ne*) (Supplementary Data 14). Communities in the Central Argentina likely had sizes comparable to those of the Central Andes, and both larger than those in the Argentinian Northwest and the Paraná River region. Individuals from the Pampas showed the highest estimated effective population size, plausibly inflated by the inferred history of admixture in that region (Table 2).

**Extended Data Figure 9.**
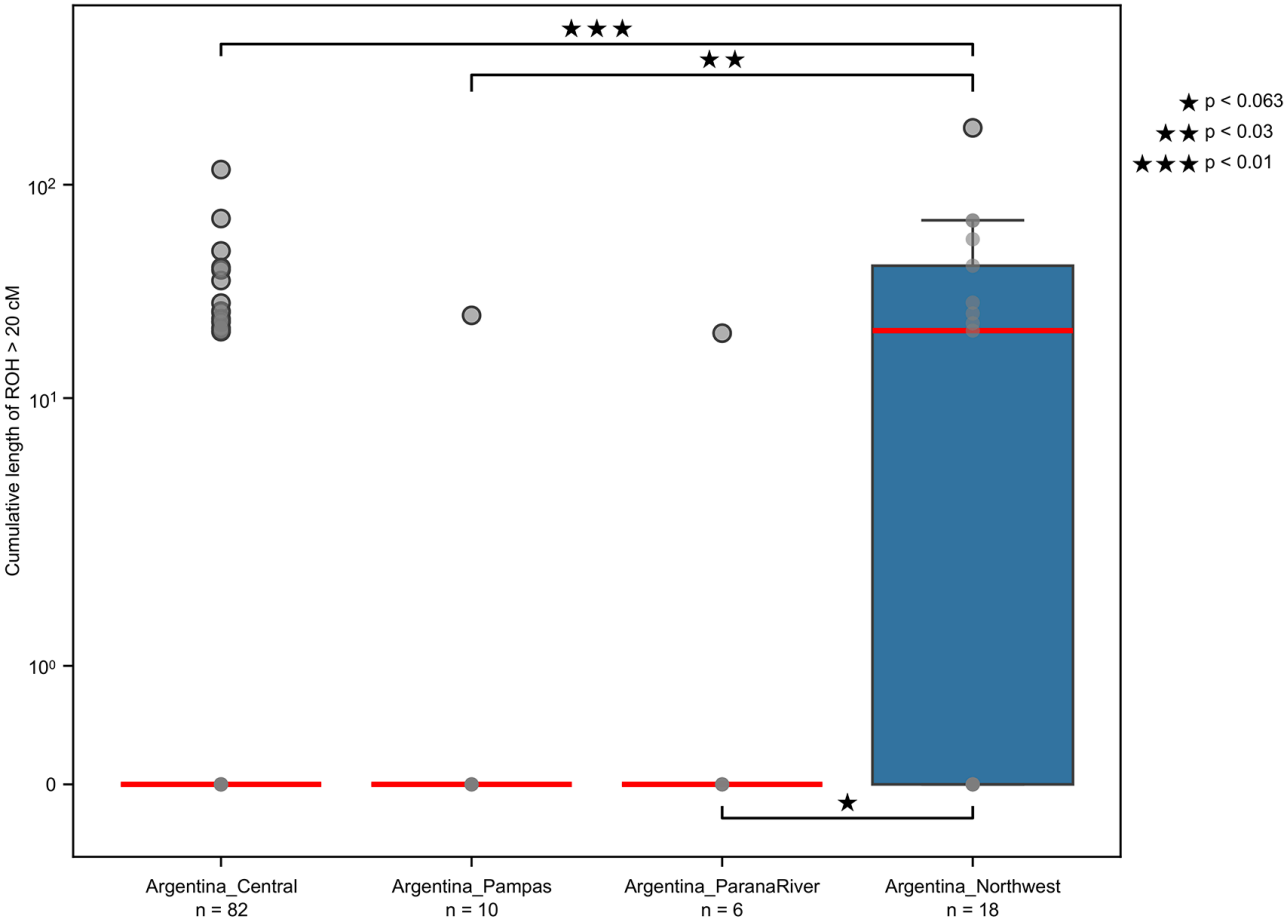
Differences in the distribution of cumulative length of ROH segments greater than 20cM for groups ≤3000BP. Horizontal red lines denote median values (log scale), with boxes showing the interquartile range (IQR) and bars showing 1.5 x IQR Pairwise group comparisons were performed using a Conover’s test (two-sided), with correction for multiple comparisons (Benjamini–Hochberg) at FDR=0.05. Corrected *p*-values for a difference between Northwest Argentina and Central Argentina (*p*=0.00739), and between Northwest Argentina and Argentina Pampas (*p*=0.0274), were significant at α=0.05 (see Supplementary Figure 76 for details). The number of individuals within each grouping is shown below each X axis label in the figure.

The cumulative length of ROH segments longer than 20cM primarily reflects increased parental relatedness, and allowed us to detect significant differences among study regions (Kruskal-Wallis, *p* = 0.009). To identify which region pairs were driving this result, we performed a Conover test—a non-parametric method that compares rank differences between groups—applying an FDR correction at 0.05 to adjust resulting *p-*values (Supplementary Figure 76). A higher rate of close-kin unions occurred in the Argentinian Northwest compared to the Central (*p <* 0.01) and Pampas (*p <* 0.03), suggesting differences in mating practices despite close proximity (Extended Data Figure 9). Given the genetic and cultural connections with the Central Andes (SI Section 2), this may reflect a similar phenomenon to what has been reported in that region after the decline of Wari and Tiwanaku societies (1000BP)^48^. This was interpreted as the origin or widespread adoption of the *ayllu* system, a social and political unit bound together by rules of kinship affiliation and reciprocity, with preference of within-group marriage to facilitate cooperation and keep resources within the community. Although the *ayllu* is not documented in Northwest Argentina archaeologically or ethnographically, our findings pointing to a common pattern of close-kin marriage reinforces the evidence of a related process.

**Extended Data Figure 10.**
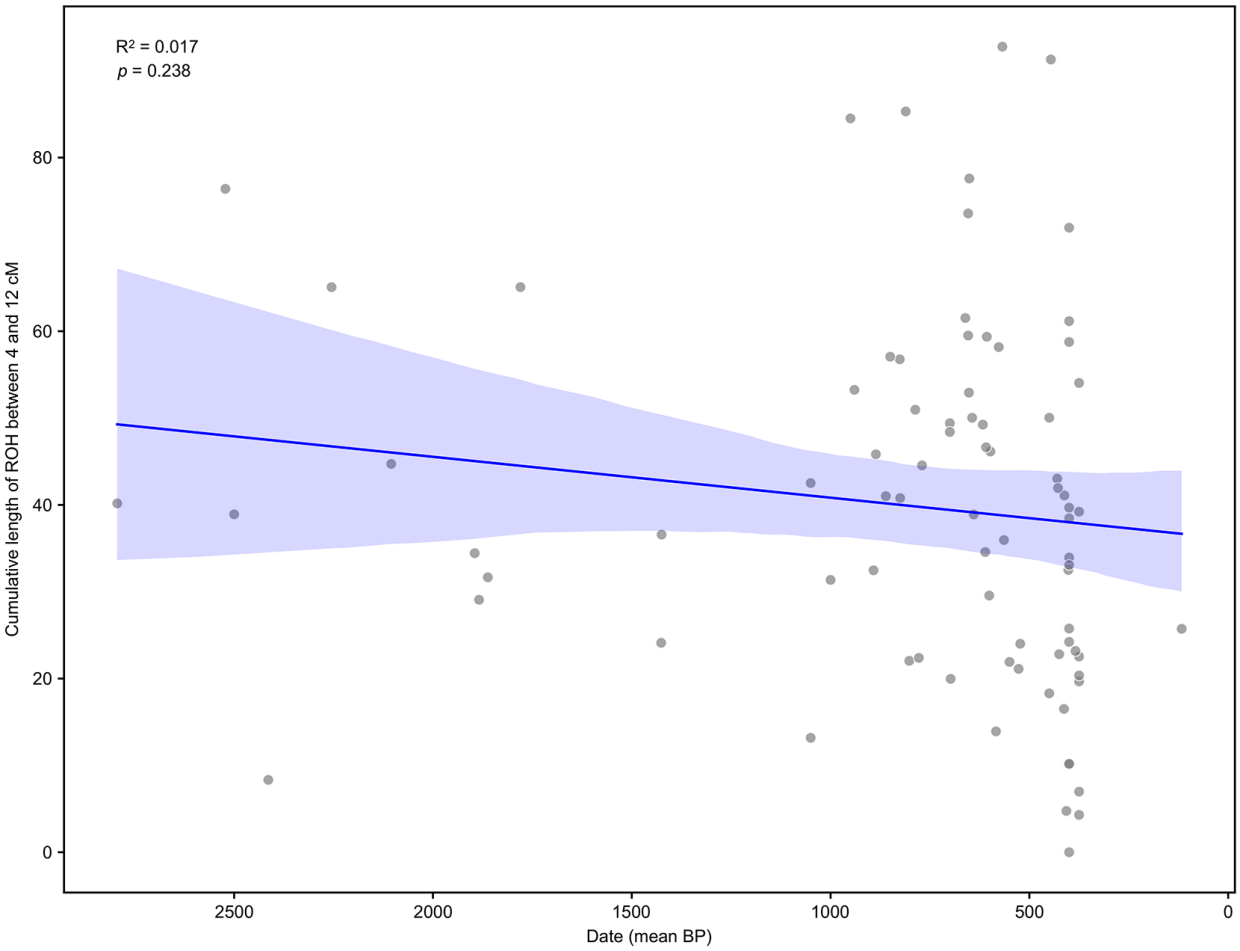
Linear regression of cumulative length of ROH between 4 and 12 cM on date (mean BP), for individuals from Argentina Central at high enough coverage to call ROH (mean BP below 2500). Error bands show 95% confidence intervals around the mean linear regression fit. There is no evidence of a significant association (*p*=0.238 from a two-sided *t*-test on the slope coefficient being zero).

In the Central region, where we had a large sample size, we tested for an association between time and the cumulative length of ROH between 4 and 12 cM, which reflects background relatedness and thus is informative of community sizes. We found no evidence of population size growth in the last two and a half millennia (Extended Data Figure 10).

## Discussion

Our finding that a 10000BP Southern Cone individual shared more alleles with Middle Holocene individuals from the same region than with individuals from Central Andes or Central Eastern Brazil places a lower bound on genetic divergence of Southern Cone people.

We also identify a previously unsampled deep lineage in Central Argentina that possessed distinctive genetic drift by 8500BP and persisted as the main ancestry component throughout our time transect. This overall genetic homogeneity co-existed with the language diversity observed in the region by the 16^th^ century, suggesting that these languages likely developed largely *in situ* and are not associated with deep genetic structure. This cautions against simplistic extrapolations regarding the mechanisms underlying linguistic and genetic differentiation^49^. We found that the Central Argentina lineage is geographically structured along two clines, one reflecting admixture with Central Andes-like ancestry and the other with Middle Holocene Pampas-like ancestry. Individuals clustered with geographically proximate groups, regardless of date, suggesting limited gene flow among communities.

In the Pampas, this deep Central Argentina lineage expanded southwards, where it admixed, beginning by at least 3300BP, with the distinct Middle Holocene genetic component in that region^11^, eventually becoming the dominant ancestry in the Pampas during the last millennium. There is a gap in available data from the Pampas between 6800BP and 3300BP, and more densely sampled time series would enable a richer characterization of this process.

In Northwest Argentina, we document a long-standing presence of Central Andes-type ancestry, at least by around 9000BP; and evidence of genetic connectivity between the Central Argentina and Central Andes lineages potentially as early as 4600BP.

We infer an admixture event in the Gran Chaco region involving a Tropical/Subtropical Forests-like source and the Central Argentina lineage. This is consistent with archaeological evidence of increased population movements into the Gran Chaco since about 800BP^50^. In the Paraná River Lower Delta, a 400BP individual with a Guaraní-associated archaeological context clustered with populations from Brazil, a region with the largest density of Guaraní sites^27^. We found no evidence of a specific affinity between modern and ancient published Arawak-associated individuals from the Caribbean, north of South America, and the Gran Chaco, and the Paraná Delta groups, and thus, while there is archaeological support for a local adoption of Arawak cultural traits, we were not able to detect a significant migration with our data.

We find a higher rate of close-kin unions in Northwest than Central Argentina, potentially reflecting adoption of what in the Central Andes was the *ayllu* social system, a kinship-based organizational structure.

A limitation in our study is sparse sampling of the Mid-to-Early-Holocene, and of the Pampas, Gran Chaco and Pantanal regions. However, the genetic structure revealed here provides a basis for correlation to archaeology, and enriches our understanding of an important world region.

## Online Methods

### Genetic data

We produced 504 ancient DNA libraries from 344 distinct skeletal samples (Supplementary Data 1). We used in-solution enrichment for over 1.2 million targeted single nucleotide polymorphisms (SNPs), a standard set of genetic markers widely used in ancient DNA studies^51-54^ to gather genome-wide data that met standard criteria for ancient DNA authenticity from 238 unique individuals (Supplementary Data 1). To maximize usable information for genetic analysis, we expanded this targeted SNP set with off-target sites (sites not originally targeted by the enrichment protocol but commonly captured because of close physical proximity) to arrive at approximately 2 million SNPs described in^54^. Individuals were assigned to groups using archaeological, geographical and chronological criteria. The 238 individuals from the Central Southern Cone were grouped into six biogeographical regions of Argentina and one from the Pantanal region of Paraguay as described in the main text, which we further subdivided for analysis as described in the Supplementary Information (Sections 1-7). Each individual was assigned to one of the main regions and subregions based on their geographic origin. Individuals from the same subregion were further grouped according to chronological criteria. In a few cases, individuals from the same region and time period were separated into different groups based on distinct cultural or archaeological characteristics (e.g., Inca and Guaraní).

The newly-reported individuals were co-analyzed with genetic data from 588 ancient pre-European contact American individuals (Extended Data Figure 1, Supplementary Data 1)^2,11,29-34,55-74^, with the data curated as described in the Allen Ancient DNA Resource^75^, a publicly-available collection of ancient human genome-wide data. For co-analysis purposes, we defined “pre-European contact American individuals” as those having a mean date (either a direct radiocarbon date or a contextual date from archaeological evidence) before 600BP. We also included in the analysis previously-generated SNP array data from present-day Native American groups^2^, restricted to the sites intersecting the “1240k SNP set”^52^.

### Direct accelerator mass spectrometry14*C* bone dates and calibration

We report 35 new direct accelerator mass spectrometry 14*C* dates obtained from specialized laboratories at Pennsylvania State University [PSUAMS] (*n* = 13) and the University of Georgia [UGAMS] (*n* = 22), which we combined with 98 previously-reported 14*C* dates from studies of the newly individuals (Supplementary Data 1). We also integrated archaeological context information to provide information on chronology (SI Sections 1-7). Additionally, we made use of 398 previously-reported 14*C* dates for the previously-published ancient American individuals whose genetic data we used for co-analysis (Supplementary Data 1). All calibrated 14*C* ages were calculated using OxCal (v.4.4)^76^ with the Southern Hemisphere terrestrial (IntCal20)^77^ calibration curves. The marine reservoir effect was not considered, as all individuals analyzed in this study had a terrestrial-based subsistence. Calibrated dates are reported in Supplementary Data 1 and in the Supplementary Information (Sections 1-6) as 95.4% CI calibrated radiocarbon ages in BCE-CE format. We also report the date mean in BP, in years before 1950 CE (calculated as the OxCal mu for a direct radiocarbon date, or as the average of the range for a contextual date), as well as the date standard deviation in BP (OxCal sigma for a direct radiocarbon date, or the standard deviation of the uniform distribution between the two bounds for a contextual date). Individual dates listed under Individual IDs correspond to the date mean in BP (years before 1950 CE), rounded to the nearest hundred, except for the individual *Argentina Central Plains SouthCordoba BarrioAlberdiRioCuarto 150BP*. Grouping dates listed under Group ID are expressed as the average of the individual date means in BP (years before 1950 CE) of the group members, also rounded to the nearest hundred.

### Ancient DNA laboratory work

Tooth or bone powder was prepared in dedicated clean rooms at Harvard Medical School (HMS) by processing 228 samples corresponding to 201 individuals, and at the University of Tennessee, Knoxville (UTK), using a freezer mill for 108 samples from individual remains. Further wet laboratory processing for all these samples was conducted at HMS. Eight samples from six individuals (including two independent duplicates of individuals powdered at UTK) were analyzed at the Australian Centre for Ancient DNA (ACAD). Additionally, for one sample, bone powder was prepared in dedicated clean rooms at University of Tübingen (UT) by abrasing the outer layer of the temporal bone surface before sampling the cochlea from the internal acoustic meatus. Around 50 mg of bone powder was generated using an electric dentist drill. DNA was extracted from powdered samples using a method optimized for retaining small DNA fragments^78-80^. The DNA was converted into sequenceable form using double-stranded or single-stranded library preparation protocols, typically pretreated with uracil-DNA glycosylase (UDG) to minimize cytosine-to-thymine errors common in ancient DNA^81-83^, expect for DNA processed at UT, which was converted into sequenceable form using single-stranded, double indexed library preparation protocols with no UDG treatment^82^, generating multiple libraries from the same extract. For all double-stranded libraries (except for four prepared at the University of California Santa Cruz), we replaced MinElute columns for reaction cleanups with magnetic silica beads and Qiagen Buffer PB. We then used SPRI beads instead of MinElute columns for PCR cleanup at the end of library preparation^84,85^, except for libraries prepared at the University of California Santa Cruz. For libraries prepared at UT, nuclear in-solution capture was performed directly, foregoing shotgun sequencing.

We enriched the libraries for sequences overlapping mtDNA^86^ and approximately 1.24 million nuclear targets together (1240k+) through two rounds of enrichment^51-53^, except for the 4 libraries from the University of California Santa Cruz, for which the mtDNA (1 round) and 1240k (2 rounds) enrichments were performed independently. For a number of libraries, including the 8 from ACAD, we used the Twist 1.4M capture kit^54,87^ instead of the 1240k enrichment, which gives more uniform coverage and targets a somewhat larger set of SNPs. For some samples we prepared 2 libraries simultaneously, and multiplexed them into one capture reaction; double-stranded libraries were captured for a single round, while single-stranded libraries were captured for 2 consecutive rounds. The unenriched (shotgun) and enriched products (mtDNA, 1240k, 1240k+, Twist1.4M) of double-stranded libraries were indexed and sequenced on an Illumina NextSeq500 instrument for 2 × 76 cycles and 2 × 7 cycles, or on an Illumina HiSeq X10 or NovaSeq instrument using 2×101 cycles and 2×7 cycles, expect for the data prepared at UT, which were sequenced on a NovaSeq platform for 2 × 121 cycles and 2 × 8 cycles generating. Single-stranded libraries and double-stranded libraries prepared at ACAD were already indexed at the end of library preparation and were sequenced on either Illumina HiSeq X10 or Novaseq instruments for 2 × 101 and 2 × 8 cycles. For the single-stranded libraries, we used a custom sequencing read 1 primer CL72. We sequenced the nuclear capture products for about 20M reads per library (on average 30-40 Million reads per captured library in the case of data prepared at UT), and also for typically hundreds of thousands of reads for the unenriched/shotgun library.

### Computational processing of sequence data

We merged paired reads overlapping by at least 15 nucleotides (allowing one mismatch) using custom code that concurrently trims adapters (https://github.com/DReichLab/ADNA-Tools), selecting the highest quality base for each nucleotide in the overlap. Non-merging read pairs were discarded. The resulting merged sequences were then mapped to the human genome reference sequence (GRCh37 from the 1000 Genomes Project^88^ using the *samse* command of the Burrows-Wheeler aligner (BWA)^89^ (v.0.7.15). Duplicate sequences were marked with Picard (command MarkDuplicates) (v.2.17.10; http: //broadinstitute.github.io/picard/). For variant calling, we used a pseudo-haploid approach, representing each SNP with a single allele representative. We first estimated error rates empirically (assuming that sites monomorphic in 1000 Genomes data^88^ are in fact monomorphic). We stratified these error rate estimates by library type, SNP bases (variant and reference), read position, strand, mapping quality, and base quality, with the base positions more than 10 bases from the 5’ or 3’ end being considered central and merged. These error rates are determined from the sample BAM, which makes our procedure adaptive. If we simply thresholded on the estimated error, this would introduce bias. For example, at a (*C, T*) SNP, the estimated error *E*(*C, T*) for *C →T* may be very different from *E*(*T, C*) for *T →C*. Instead we use a symmetric function *S* and, for instance, at a *(C, T)* SNP, we calculate *S* = max*{E*(*C, T*)*, E*(*T, C*)*}*. We threshold *S* with a parametric value (0.02) and bases with *S* below threshold go into a pileup of reliable bases. Finally, a random base in the pileup is selected. The actual error achieved is smaller than the threshold which is an upper bound on the error of each potential base that contributes to the pileup. For analysis, we used the SNP set that includes off-target sites apart from the standard “1240k” sites and is described in^54^.

### Contamination estimation

We evaluated the authenticity of ancient DNA by measuring the damage rate in the first nucleotide, and we flagged individuals as potentially contaminated if the cytosine-to-thymine substitution rate was less than 3% in UDG-treated libraries and less than 10% in non-UDG-treated libraries. Contamination evidence based on mtDNA polymorphism was determined using contamMix^90^, while hapConX^91^ and ANGSD^92^ were used to assess contamination evidence based on X-chromosome polymorphism in males (Extended Data Tables 0.1. and 0.2). These individuals were excluded from analysis, but we report their data. Additionally, we excluded, but still reported, individuals from analysis who were not genetically homogeneous with ancient pre-European contact Native Americans as assessed by either $f_{4}$-statistics or *qpAdm* (SI Section 8, Supplementary Data 1).

### Kinship analyses

We analyzed all pairs of individuals to test for evidence of close biological relatedness. In particular, we examined all non-CpG autosomal sites and calculated the mean mismatch rate at all SNPs covered by at least one sequence in both individuals. We compared this to the rate of difference between the two chromosomes within each individual, assuming that they were not closely related^60^. Individuals inferred to have a 2nd degree or closer relationship with someone else in the dataset (Supplementary Data 1) were excluded from analyses, usually keeping the individual with higher-coverage data (see Supplementary Section 1.2 for details).

### $f_{4}$-statistics and outgroup *f*_3_-distance matrices

To compute *f*_3_-and $f_{4}$-statistics, we used the *qp3pop* and *qpDstat* packages in ADMIXTOOLS^93^ (v.7.0.2). When indicated because of an extremely large number of tests, we corrected $f_{4}$-statistic *Z-*scores at FDR *<* 0.05 using the Benjamini-Yekutieli procedure^94^ (*Z*_*BY*_) using a custom script available at https://github.com/javiermaravall/aDNA_CSC/. Using the outgroupmode: YES parameter, we computed outgroup*-f*_3_ statistics of the form *f*_3_(Pop 1, Pop 2; Yoruba) or *f*_3_(Ind 1, Ind 2; Yoruba). Because these quantities measure shared drift with respect to the outgroup up to the split of Pop 1 and Pop 2^95^, or of Ind 1 and Ind 2, their inverses can be appropriately used to construct a pairwise genetic-distance matrix. We used these matrices to compute neighbor-joining trees using the ape R package (v5.8)^96^, rooting them at *USA_Ancient_Beringian.SG*. To obtain a low-dimensional representation of these objects, we applied MDS to the matrices using the function *cmdscale* from the *R stats* package [58] (v3.6.2) ^97,98^.

### Automatic exploration of population history models

To automatically explore the space of population history models (admixture graphs), we used the R library ADMIXTOOLS2^99,100^ (version 2.0.0). To extract data, we used function *extract f2* setting *maxmiss=0.15*. This kept 329279 SNPs, 293834 of which were polymorphic among the studied groups. Although the recommended value of this parameter is 0 for automatic population-history model exploration, lower values of allowed missingness resulted in too small numbers of SNPs retained (<30000). We launched 100 independent iterations of the function *find graphs*, for each of *n* = 0, 1 admixture events, which starts from a given set of populations and explores admixture graphs until the resulting graph cannot be made to better fit the data. Because this search can get trapped in local optima, the execution of a large number of independent iterations, each starting from a randomly-initialized admixture graph, enables better characterization of the set of optimally-fitting graphs. For each *n* and each iteration, we recorded the hash (unique topology identifier), score (a measure of fit) and worst residual (*Z-*score for the largest deviation between observed $f_{4}$-statistics and the value predicted by the model). For each *n*, we gathered all final models with a unique hash, and aggregated these across values of *n*. This resulted in a set of 52 unique models (Supplementary Data 2). To understand if some elements of this set better fit the data than others, we tested, for each pair of models, whether the scores were significantly different. To this end, we used the functions *qpgraph resample multi* and *compare fits*, which perform this test using a combination of holding out data and SNP block bootstrap resampling, to account for both differences in model complexity and potential differences in scores due to chance alone. Because these tests indicated no evidence for invoking a higher number of admixture events (Supplementary Data 2), we chose not to explore models with a number of admixture events greater than 1.

### Computation of *F*_*st*_ values

To compute *F*_*st*_ between pairs of groupings, we used smartpca^101^ (v. 18711), with the flags inbreed: YES, fstonly: YES, fstverbose: YES. We restricted to groupings for which at least 5000 SNPs were used for all pairwise computations. We computed a complete hierarchical clustering tree with the package *linkage* from the *scipy* library^102,103^ (v.1.16.0).

### Testing cladality and sources of ancestry using qpWave and qpAdm

Determining whether pairs of populations (A, B) and (C, D) form clades can be reframed as evaluating whether a single gene flow event separated the pairs ($f_{4}(A,B,C,D)=0$) or multiple events occurred ($f_{4}(A,B,C,D)\neq 0$). The *qpWave* method estimates the minimum number of gene flow events between two groups, L and R (sizes $n_{L}$ and $n_{R}$). It uses $f_{4}$-statistics $f_{4}(L_{i},L_{f};R_{m},R_{n})$ to quantify shared genetic drift within L and R. If L and R form distinct clades, all $f_{4}$-statistics should be zero. It uses $f_{4}$-statistics of the form $\frac{n_{L}(n_{L}-1)}{2}\cdot \frac{n_{R}(n_{R}-1)}{2}$, forming a matrix X. The rank of X indicates the minimum number of gene flow events; a higher rank suggests more events. Practically, X is an $\left(n_{L}-1)\times (n_{R}-1\right)$ matrix of $f_{4}(L_{1},L_{i};R_{1},R_{j})$ statistics. If $n_{R}>n_{L}$, the maximum rank is $n_{L}-1$, implying at least $n_{L}-1$ gene flow events. *p*-values are derived from a $\chi^{2}$-distribution based on log-likelihood differences between models. Full details are in the original publication^2^ . *qpAdm* extends this concept to assess the genetic make-up of an additional population T, by comparing gene flow events in L and R with those in L∪T and R. If L∪T and R show more events than L and R, T has gene flow with R and cannot be modeled solely from L. If both models yield the same rank, T can be modeled from L, allowing estimation of contributions from L to T^53^. For *qpWave* computations, we used ADMIXTOOLS^93^ (v.7.0.2), setting the allsnps: YES. For *qpAdm* computations, we used ADMIXTOOLS2^99,100^ (version 2.0.0), setting allsnps=TRUE. To quantify a Mesoamerican contribution into *Chile_LosRieles_5100BP*, we performed an inverse variance-weighted meta-analysis across passing models with a Mesoamerican-related source (Supplementary Data 3). Dates of admixture events were estimated using *DATES*^104^ (v210), but were too noisy.

### ADMIXTURE clustering analysis

We used the ADMIXTURE^105,106^ (v1.23) software package to perform an unsupervised assessment of genetic structure among the newly-reported individuals, including ancient (Extended Data Tables 0.2. and 0.3) and modern^2^ Native Americans for reference. The Karitiana and Surui groups were excluded, to avoid biases that can arise through the presence of highly-drifted populations^107^. Input data was prepared using PLINK (v 1.9)^108^. We used the maf 0.01 parameter to remove SNPs with minor allele frequency below 0.01. To prune out genetic markers in strong linkage disequilibrium (LD), we applied the indep-pairwise parameter with the following options: a pairwise $r^{2}$ threshold of 0.4, a window size of 200 variants and a step size of 25 variants. For each value K=1,…,12 of the number of source populations, we ran 4 random-seed replicates.

### Analyses of runs of homozygosity

To call runs of homozygosity (ROH) longer than 4cM in ancient individuals, we used *hapROH*^109^ (v0.64). We used the 1000 Genomes Project haplotype panel^88^, which includes 5,008 global haplotypes, as our reference panel. We restricted analysis to individuals for whom at least 400,000 SNPs were covered with respect to the 1240k SNP set. Because this methodology was calibrated for the 1240k SNP set, not including off-target sites, we downsampled to the 1240k SNP set for this analysis. All analyses were conducted using the default settings of *hapROH*. To estimate effective population sizes for study subregions (*Ne*) from ROH distributions, we restricted to individuals with a mean date up to 3000 BP and with a cumulative sum of ROH segments longer than 20cM below 50 (to avoid biases due to inbreeding) and used function MLE ROH Ne() from *hapROH* (Supplementary Data 14). To test for significant differences among study subregions in the ROH distributions of segments above 20 cM (informative of recent instances of close parental relatedness) we used the python library SciPy v.1.13.1^102,103^ to perform a Kruskal-Wallis test (function *kruskal()*) using the cumulative length of segments in that length range for each individual, which we followed up on with a Conover test for each pair of subregions, performed using the python library *scikit-posthocs*^110^ *v.0.9.0*, and correcting *p–*values at FDR *<* 0.05 (function *posthoc conover()* with parameter p adjust= ‘fdr bh’). To test for a significant association between ROH segments in the range 4-12 cM (which are informative of the levels of background relatedness and thus of effective population sizes) and time in the Central Argentina region, we regressed the cumulative sum of segments in that length range on mean date, for Central Argentina individuals with a mean date below 2500 BP, using the SciPy library^102^ v.1.13.1 (function *linregress(*)).

### Map plotting

Figure 1a was generated in R^111^ v.4.3.2 with open-source packages *dplyr*^112^ *v1.1.4*, *ggforce*^113^ *v0.4.2*, *ggnewscale*^114^ *v0.4.10*, *ggplot2*^115^ *v3.4.4*, *ggspatial*^116^ *v1.1.9*, *ggstar*^117^ *v1.0.4*, *ggrepel*^118^ *v0.9.5*, *paletteer*^119^ *v1.3*, *raster*^120^ *v3.6-26*, *rnaturalearth*^121^ *v1.0.1*, *sf*^122,123^ *v1.0-15*, *tidyterra v0.5.2*^124^ and *terra*^125^ *v1.7-*71, using Natural Earth (https://www.naturalearthdata.com), GADM (https://gadm.org) and Portal de Información Hídrica de Córdoba-APRHI (https://portal-aprhi.opendata.arcgis.com/) data.

### Ethics Statement

This study adhered to ethical guidelines for working with human remains drafted both by a diverse and international group of anthropological and paleogenetic scholars^126^ and the Argentine Association of Biological Anthropology^127^, treating these deceased individuals with respect and using minimally-destructive analyses techniques. Our research program involving ancient human remains received approval from the Ethics Committee of the CEMIC (Comité de Etica en Investigación, Centro de Educación Médica e Inves-tigaciones Clínicas ‘Norberto Quirno’). Skeletal samples were exported with authorization from the institutions safeguarding them (provincial and national museums, universities, etc.), obtaining proper permits from each province (e.g., Agencia Córdoba Cultura), and the Argentina government (Instituto Nacional de Antropología y Pensamiento Latinoamericano and Customs). In instances where Indigenous communities were associated with these individuals, analyses were conducted in engagement with these communities (i.e., ^128^, primarily facilitated through interactions between archaeologists and the communities). In the particular case of samples from the Córdoba province, we secured endorsement and support for this research from the Consejo de Comunidades de Pueblos Indígenas de la Provincia de Córdoba, Argentina (Council of Communities of Indigenous Peoples of the Province of Córdoba).

As part of our ongoing commitment to responsible and ethical research practices, we summarized the main results of our analyses in a simplified, bulleted text in Spanish describing regional-level population history inferences (SI Section 7), and shared it with Indigenous communities (when present or identified), rural localities, regional Indigenous councils (such as the mentioned Consejo de Comunidades de Pueblos Indígenas de la Provincia de Córdoba), and other stakeholders, including museum directors and curators, landowners, and local authorities. We received positive and constructive feedback from them, including comments regarding how the genetic insights could be integrated with their traditional knowledge about their history.

## Extended Data

**Extended Data Table 1.** Selected $f_{4}$-statistics. *Plains_MiddleSaladoRiver_SantiagodelEstero_400B* is a late Central Argentina population that is a clade with *Central_JesusMaria_8500BP* and contains tens of well-covered individuals, increasing power for $f_{4}$-statistic computations. Toba and Wichí are modern populations from the Gran Chaco. Karitiana and Piapoco are modern populations from the Northwest Brazilian Amazon and Eastern Colombia, whose ancestry is characteristic of Tropical and Subtropical Forests Native American peoples [3, 10]. Blue statistics show that Late Holocene Pampas populations cannot be related with Central Argentina and Middle Holocene Pampas via a simple tree, indicating gene flow between these two lineages. Red statistics show similar patterns for Northwest Argentina context labels in the case of the Central Argentina and Central Andes lineages. Green statistics show similar patterns for modern Gran Chaco populations for Central Argentina and the Forest and Subtropical Forests ancestry components.

■	Late Holocene Pampas populations cannot be related with Central Argentina and Middle Holocene Pampas populations via a simple tree.				
■	Similar evidence of admixture between the Central Argentina and the Central Andes lineages in Northwest Argentina.				
■	Similar evidence of admixture between the Central Argentina lineage and a Tropical and Subtropical Forests source in the Gran Chaco region.				

Outgroup	Pop_1	Pop_2	Pop_3	Z	n_SNPs
Yoruba	Argentina_Pampas_6800-7700BP	Argentina_Central_Plains_MesopotamiaSantiagoDelEstero_400BP	Argentina_Pampas_CentralPampeanDunefields_1600BP	4.65	898597
Yoruba	Argentina_Central_Plains_MesopotamiaSantiagoDelEstero_400BP	Argentina_Pampas_6800-7700BP	Argentina_Pampas_CentralPampeanDunefields_1600BP	7.053	898597
Yoruba	Argentina_Central_JesusMaria_8500BP	Argentina_Pampas_6800-7700BP	Argentina_Pampas_Southern_2600BP	2.613	446224
Yoruba	Argentina_Pampas_6800-7700BP	Argentina_Central_JesusMaria_8500BP	Argentina_Pampas_Southern_2600BP	3.6	446224
Yoruba	Argentina_Central_Plains_MesopotamiaSantiagoDelEstero_400BP	Bolivia_Akapana_Tiwanaku	Argentina_Northwest_SubandeanValleys_Hualfin_2400BP	4.129	922246
Yoruba	Bolivia_Akapana_Tiwanaku	Argentina_Central_Plains_MesopotamiaSantiagoDelEstero_400BP	Argentina_Northwest_SubandeanValleys_Hualfin_2400BP	2.337	922246
Yoruba	Argentina_Central_Plains_MesopotamiaSantiagoDelEstero_400BP	Bolivia_Akapana_Tiwanaku	Argentina_Northwest_SouthernPuna_Antofagasta_1200BP	3.166	1084211
Yoruba	Bolivia_Akapana_Tiwanaku	Argentina_Central_Plains_MesopotamiaSantiagoDelEstero_400BP	Argentina_Northwest_SouthernPuna_Antofagasta_1200BP	2.607	1084211
Yoruba	Argentina_Central_Plains_MesopotamiaSantiagoDelEstero_400BP	Bolivia_Miraflores_MH	Argentina_Northwest_SouthernPuna_Antofagasta_2100BP	3.332	549747
Yoruba	Bolivia_Miraflores_MH	Argentina_Central_Plains_MesopotamiaSantiagoDelEstero_400BP	Argentina_Northwest_SouthernPuna_Antofagasta_2100BP	4.625	549747
Yoruba	Karitiana	Argentina_Central_Plains_MesopotamiaSantiagoDelEstero_400BP	Wichi	2.315	315637
Yoruba	Argentina_Central_Plains_MesopotamiaSantiagoDelEstero_400BP	Karitiana	Wichi	2.728	315637
Yoruba	Piapoco	Argentina_Central_JesusMaria_8500BP	Toba	3.273	131781
Yoruba	Argentina_Central_JesusMaria_8500BP	Piapoco	Toba	3.428	131781

**Extended Data Table 2.** *hapROH* estimates of effective population size (*Ne*) by region, rounded to the nearest integer. Estimates are obtained by fitting the distribution of runs of homozygosity of individuals from each region with a mean date not older than 3000BP. The estimates indicate that the communities in the Central region of Argentina likely had similar sizes as in the Central Andes, and likely higher than those in the Argentinian Northwest or the Paraná River region. The individuals from the Pampas had the largest effective population size, likely reflecting admixture.

Group	*N_e_* point estimate	Lower bound of 95% CI	Upper bound of 95% CI	*n*
Argentina_Central	707	650	762	40.0
Argentina_Northwest	438	374	514	6.5
Argentina_Pampas	1100	828	1501	4.5
Argentina_ParanaRiver	518	406	678	3.0
Brazil_Coastal	245	217	278	6.5
CentralAndes	789	683	919	13.0
SouthernPatagonia	174	147	208	2.5

## Supplementary Material

Extended Data 1

Extended Data 2

Extended Data 3

Extended Data4

Extended Data5

Extended Data6

Extended Data7

Extended Data8

Extended Data9

Extended Data10

Extended Data11

Extended Data12

Extended Data13

Extended Data14

Supplementary Information

**Supplementary information** The online version contains supplementary material available at https:[to be made available upon publication]

## Data Availability

Genotype data for newly-reported individuals included in main analyses from this study can be obtained from the Harvard Dataverse repository at doi.org/10.7910/DVN/UQVPJQ. The aligned sequences for all individuals are available through the European Nucleotide Archive, accession PRJEB97713. Previously published data used in our analyses are available as follows: genetic data for modern individuals from Native American groups^2^ are available for non-profit research on population history under an inter-institutional data access agreement with the Universidad de Antioquia, Colombia (queries regarding data access should be sent to a.ruizlin@ucl.ac.uk); genetic data for previously-published ancient individuals is available at the Allen Ancient DNA Resource (doi: 10.7910/DVN/FFIDCW); 1000 Genomes haplotype reference panel (http://ftp.1000genomes.ebi.ac.uk/vol1/ftp/release/20130502/), human reference genome hg19 (https://www.ncbi.nlm.nih.gov/datasets/genome/GCF_000001405.13/); data used for map plotting is available at Natural Earth (https://www.naturalearthdata.com), GADM (https://gadm.org) and Portal de Información Hídrica de Córdoba-APRHI (https://portal-aprhi.opendata.arcgis.com/). Other newly reported data, such as radiocarbon dates and archaeological context information, are included in this manuscript, the Supplementary Information, and Supplementary Data files.

## Code availability

Custom scripts and accompanying materials for the appropriate results sections are available at https://github.com/javiermaravall/aDNA_CSC/.
