# Supplementary material for: Eight millennia of continuity of a previously unknown lineage in Argentina
Source: Nature. Author manuscript; Available in PMC 2025 Dec 30. (PMC12747222; doi:10.1038/s41586-025-09731-3)
Supplement: Supplementary Information [file NIHMS2125634-supplement-Supplementary_Information.pdf]

Supplementary Information

For *Eight millennia of continuity of a previously unknown lineage in Argentina*

Contents

Supplementary Data 1-14 (online Excel tables) 3
Supplementary Figures 1-84 6
SI Section 1 Summary of the sampling strategy, geographic origins of the individuals, and methods for chronological assignment 63
SI Section 2 Northwest Region (Argentina) 64
SI Section 3 Gran Chaco Region (Argentina) and Pantanal (Paraguay) 81
SI Section 4 Pampas Region (Argentina) 86
SI Section 5 Paraná River Region (Argentina) 93
SI Section 6 Central Region (Argentina) 104
SI Section 7 Bulleted summary of the main results shared with stakeholders 167
SI Section 8 Preprocessing of genotype data 170
8.1 Assessment of contamination or post-colonial admixture 170
8.2 Exclusion of relatives up to 2nd degree 170
SI Section 9 Interrogation of deep lineages in the Early and Middle Holocene Central Southern Cone 171
9.1 Differential attraction to Mesoamerican and Northern South American populations 175
SI Section 10 Relationships to previously-reported relevant lineages in the Americas 179
10.1 NNA and SNA ancestries 179
10.2 Population *Y* 179
SI Section 11 Genetic affinities of newly-reported Late Holocene individuals 180
SI Section 12 Formal modelling of ancestry components 182
12.1 Northwest Argentina 183
12.2 Pantanal 185
12.3 Gran Chaco 185
12.4 Paraná River 187
12.5 Central Argentina 188
12.6 Pampas 193
SI Section 13 Relationship to ADMIXTURE results 195

Description of Supplementary Data 1-14 (online Excel tables)

I. Supplementary Data 1:

Sheet 1. Summary statistics of ancient DNA libraries generated for this study.

Sheet 2. Description of newly reported ancient individuals included in genetic analyses.

Sheet 3. Description of previously published ancient individuals included in genetic analyses.

Sheet 4. Results of `qpAdm` modeling to assess contamination.

Sheet 5. f_4 tests for presence of NNA ancestry.

Sheet 6. f_4 tests for presence of PopY ancestry.

II. Supplementary Data 2:

Sheet 1. Outgroup f_3 results for published and newly-reported Early-early Middle Holocene South Americans.

Sheet 2. f_4 statistics for Early-early Middle Holocene South Americans.

Sheet 3. Summary statistics for unique models resulting from automatic population history model exploration.

Sheet 4. $p-$values for significantly better fit for each pair of `find_graphs` final models with a distinct hash, up to 1 admixture event.

Sheet 5. f_4 statistics for Early-early Middle Holocene South American lineages, with merges identified by cladality tests.

Sheet 6. *Lauricocha_5800* appears to be genetically homogenenous with *Cuncaicha_9000*.

Sheet 7. f_4 tests for differential affinity to Mesoamerican-related populations among early ancient South Americans.

Sheet 8. `qpWave` tests for early ancient South American individuals.

Sheet 9. Affinities of Late Holocene Central Andes populations with respect to Middle Holocene Central Andes individuals.

III. Supplementary Data 3: `qpAdm` models for Mesoamerican-related contribution into *Chile_LosRieles_5100BP*.

IV. Supplementary Data 4: f_4 statistic results by study subregion.

V. Supplementary Data 5:

Sheet 1. Outgroup f_3 results for published and newly-reported ancient groups, with *YRI* as outgroup.

Sheet 2. `smartpca` F_{st} estimates for pairs of newly-reported ancient context labels.

Sheet 3. Newly-reported groups do not show evidence of excess affinity to representative Mesoamerican-related populations w.r.t. *Calamuchita_4200BP*.

Sheet 4. f_4s used as a guide in `qpAdm` modelling.

VI. Supplementary Data 6: Single-source `qpAdm` $p-$values for Central Argentina.

VII. Supplementary Data 7: Single-source `qpAdm` $p-$values for Gran Chaco.

VIII. Supplementary Data 8: Single-source `qpAdm` $p-$values for Northwest Argentina.

IX. Supplementary Data 9: Single-source `qpAdm` $p-$values for Pampas.

X. Supplementary Data 10: Single-source `qpAdm` $p-$values for Pantanal.

XI. Supplementary Data 11: Single-source `qpAdm` $p-$values for Paraná River.

XII. Supplementary Data 12:

Sheet 1. `qpAdm` $p-$values for modelling Pampas groupings as a mixture of Central Argentina and Middle Holocene Pampas.

Sheet 2. `qpAdm` $p-$values for modelling Northwest groupings as a mixture of Central Argentina and Central Andes.

Sheet 3. `qpAdm` $p-$values for modelling Northwest groupings as a mixture of Central Argentina and Central Andes (with an additional Central-Andes proxy).

Sheet 4. `qpAdm` $p-$values for modelling Gran Chaco groupings as a mixture of Central Argentina and Kartiana (proxy for Tropical and Subtropical Forests-type ancestry).

Sheet 5. `qpAdm` $p-$values for modelling Central Argentina groupings as a mixture of Central Argentina and Southern Patagonia.

Sheet 6. `qpAdm` $p-$values for modelling Central Argentina groupings as a mixture of Central Argentina and Middle Holocene Pampas.

XIII. Supplementary Data 13:

Sheet 1. Individual I12744 with an Inka archaeological association appears genetically homogeneous with other individuals in its context label.

Sheet 2. Substantial genetic affinity with Middle Holocene Central Argentina among later Central Argentina populations.

Sheet 3. Genetic affinities of modern admixed groupings from (Luisi et al. 2020, PLOS Genetics) for Early/Middle Holocene South Americans.

Sheet 4. Genetic affinities of modern admixed groupings from (Luisi et al. 2020, PLOS Genetics) for Late Holocene South Americans.

Sheet 5. Outgroup f_3 results for pairs of individuals whose grouping was inferred to carry predominantly Central Argentina-type ancestry by `qpAdm`.

Sheet 6. Absence of affinity with Arawak-related populations among Paraná river groupings.

Sheet 7. Absence of affinity with Arawak-related populations among Paraná river individuals.

XIV. Supplementary Data 14:

Sheet 1. `hapROH` output output for sufficiently-covered newly-reported and previously-published comparison individuals.

Sheet 2. `hapROH` maximum-likelihood N_e estimates.

Supplementary Figures

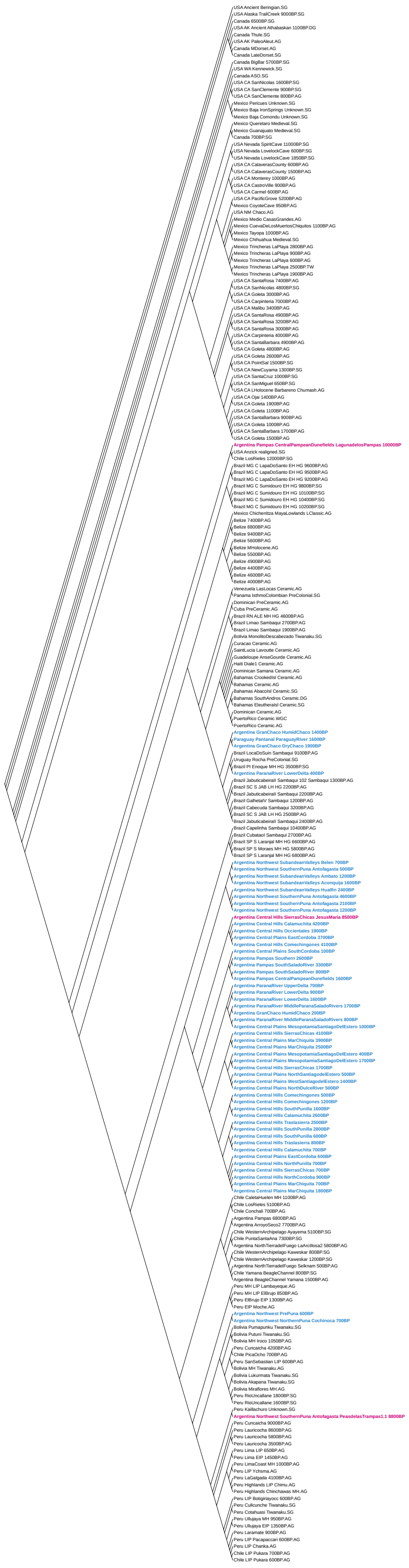

Supplementary Figure 1: Outgroup$-f_3$ neighbor-joining tree from a distance matrix of the form $1/f_3(\mathrm{Pop}_1, \mathrm{Pop}_2; \mathrm{Yoruba})$, where Pop_i, $i \in \{1, 2\}$, is a newly-reported (in blue, with Early/Middle Holocene individuals in magenta) or previously-published (in black) ancient American context label.

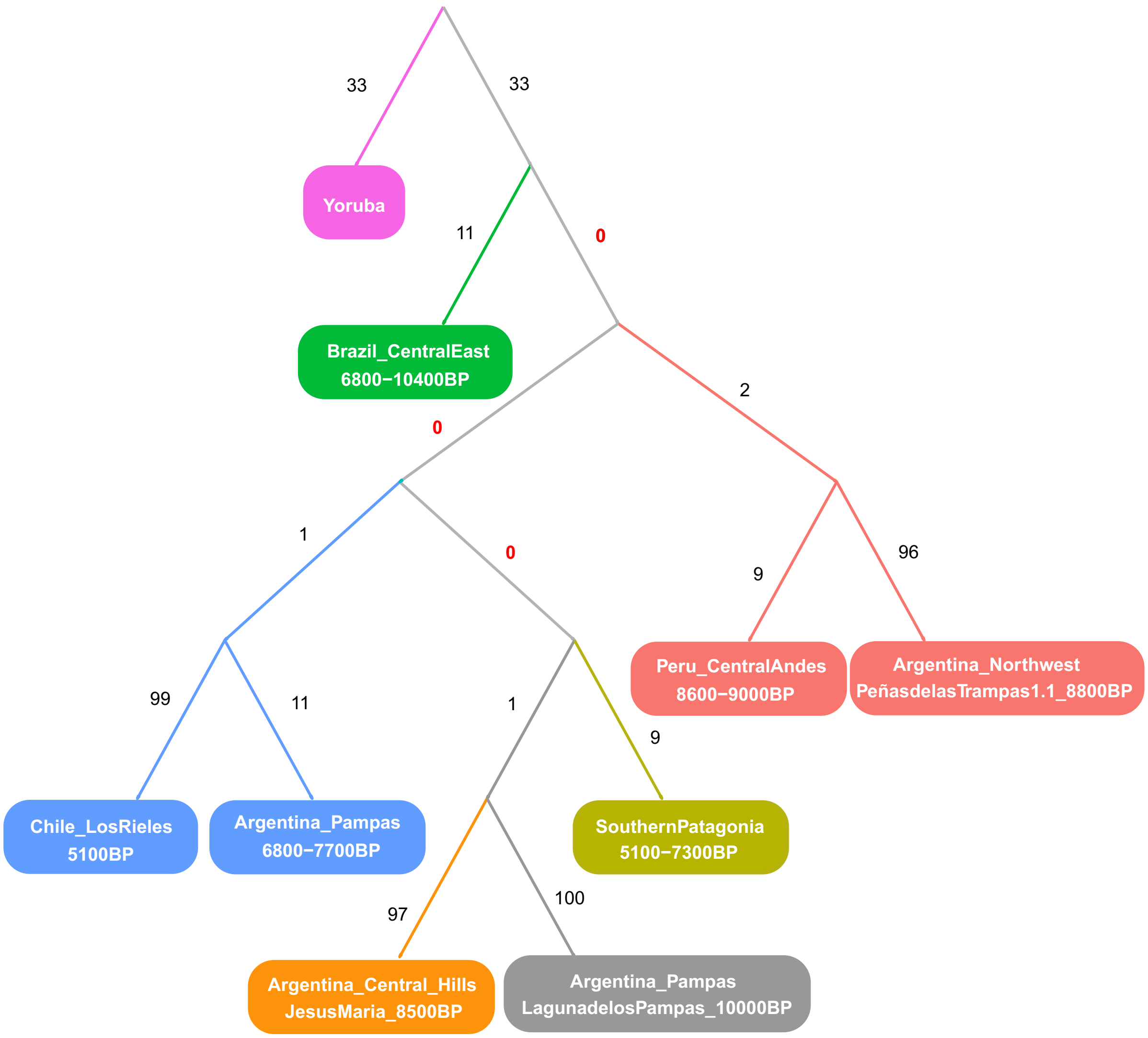

Supplementary Figure 2: First of 9 unique trees identified by `find_graphs` involving Early/Middle Holocene South American individuals. Score ≈ 34.067, worst residual ≈ 2.998.

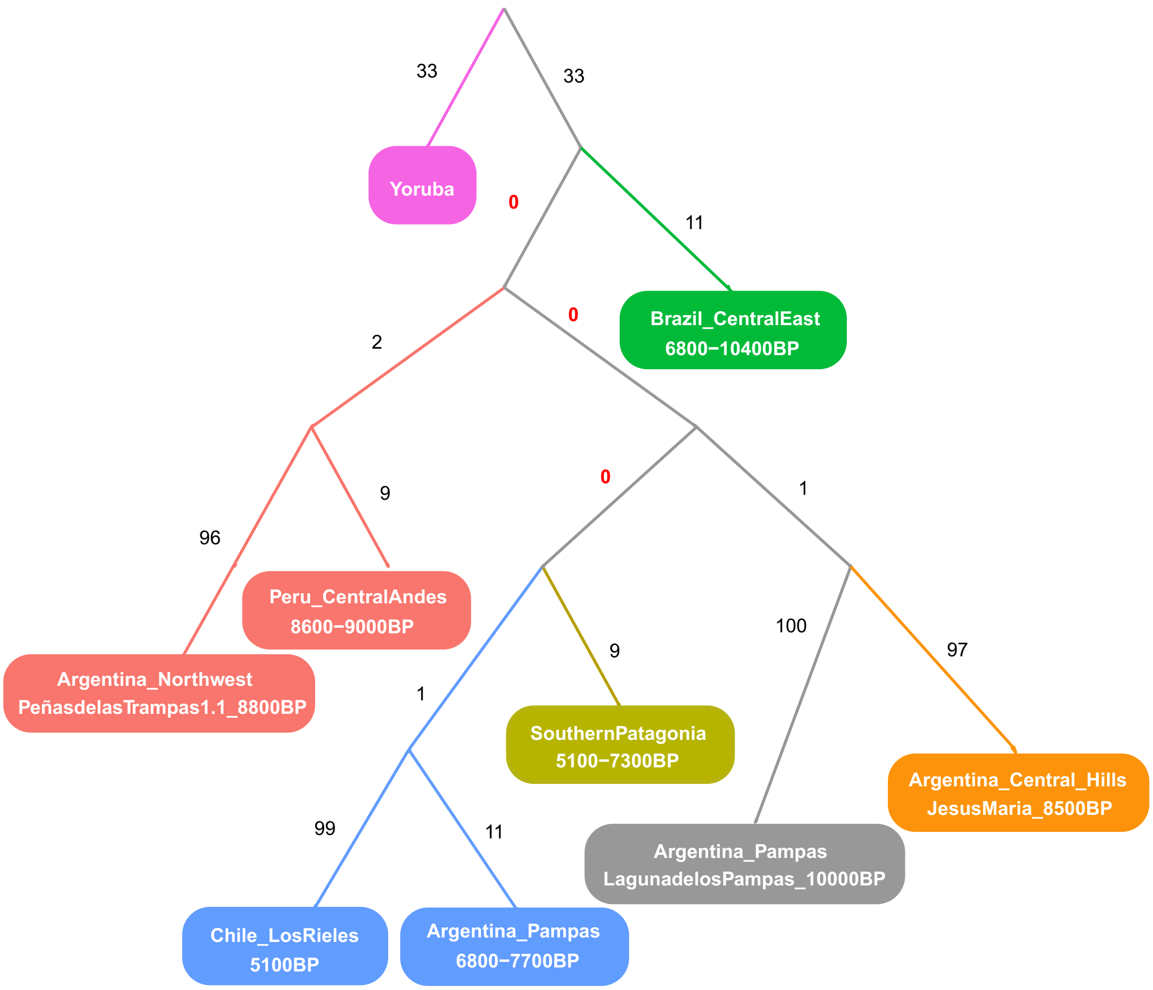

Supplementary Figure 3: Second of 9 unique trees identified by `find_graphs` involving Early/Middle Holocene South American individuals. Score ≈ 34.976, worst residual ≈ 2.864.

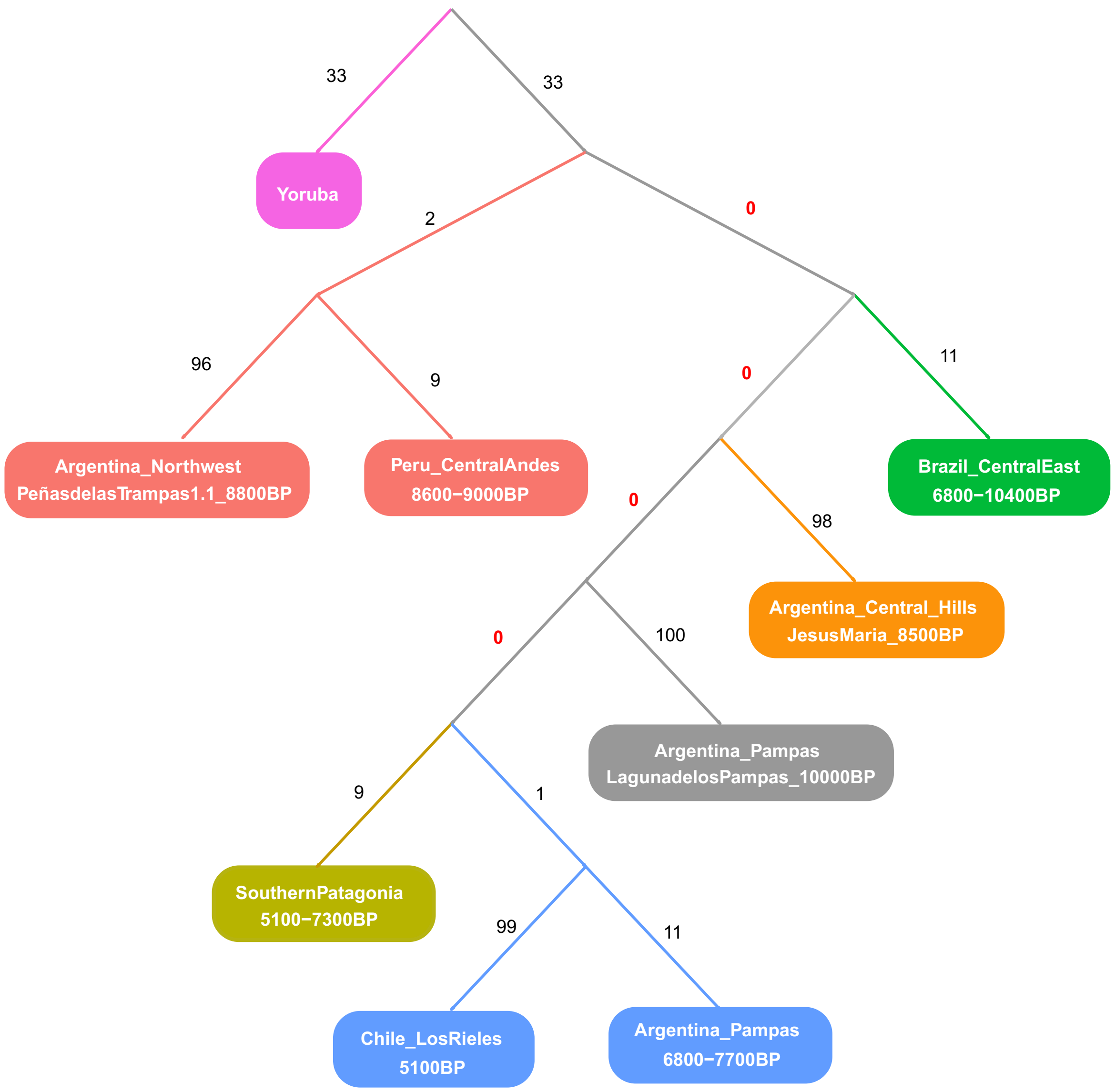

Supplementary Figure 4: Third of 9 unique trees identified by `find_graphs` involving Early/Middle Holocene South American individuals. Score ≈ 38.455, worst residual ≈ 3.545.

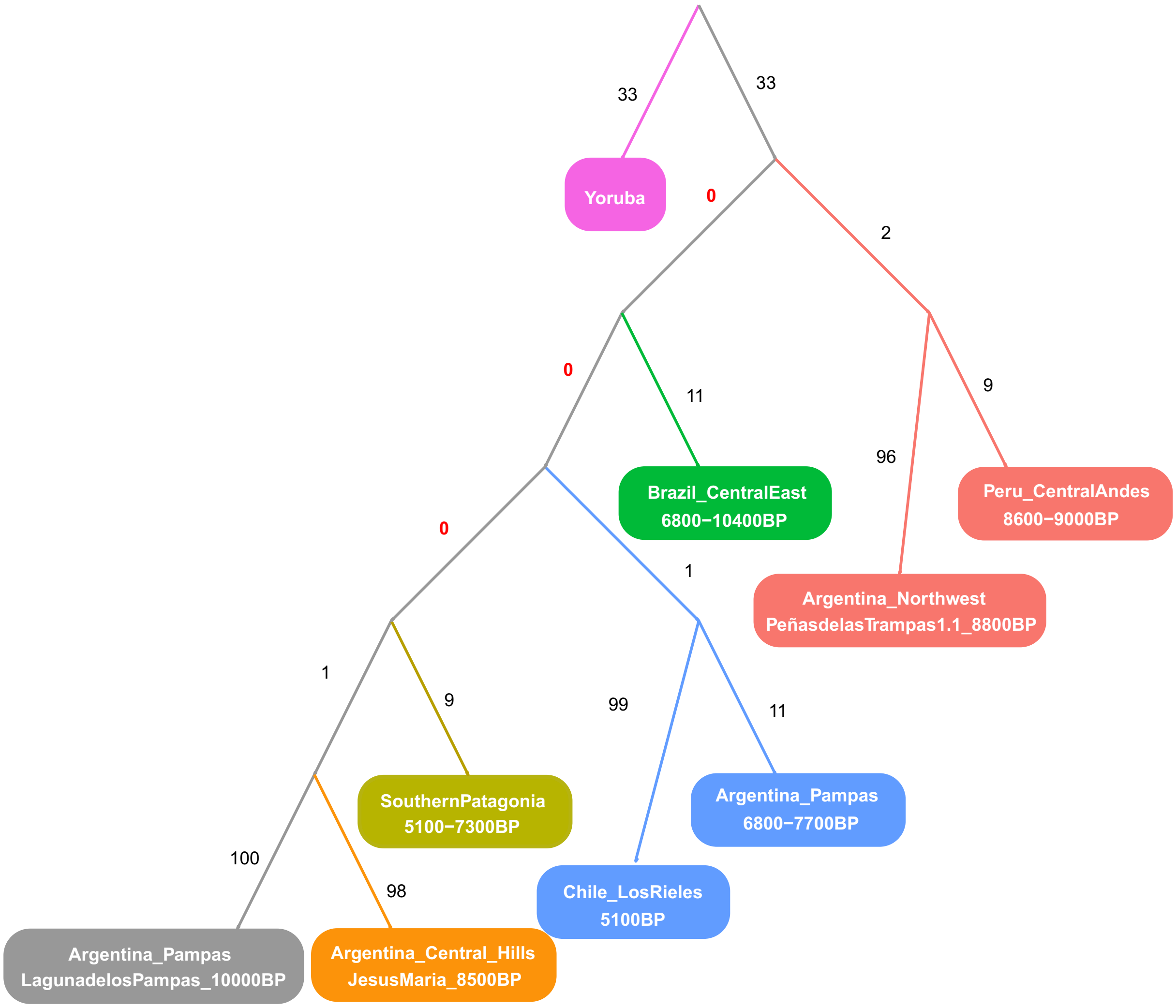

Supplementary Figure 5: Fourth of 9 unique trees identified by `find_graphs` involving Early/Middle Holocene South American individuals. Score ≈ 36.782, worst residual ≈ 3.545.

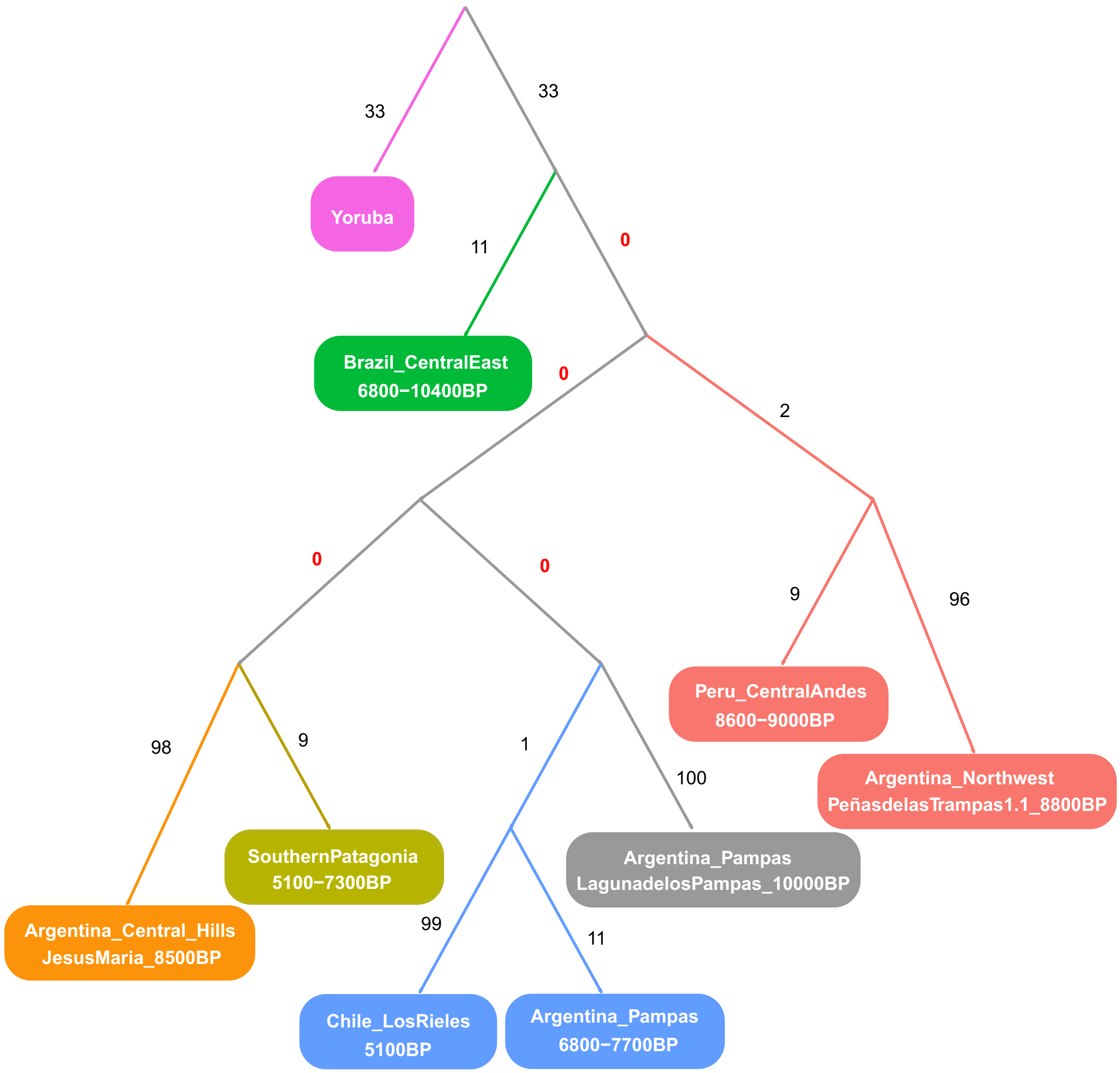

Supplementary Figure 6: Fifth of 9 unique trees identified by `find_graphs` involving Early/Middle Holocene South American individuals. Score ≈ 36.200, worst residual ≈ 2.921.

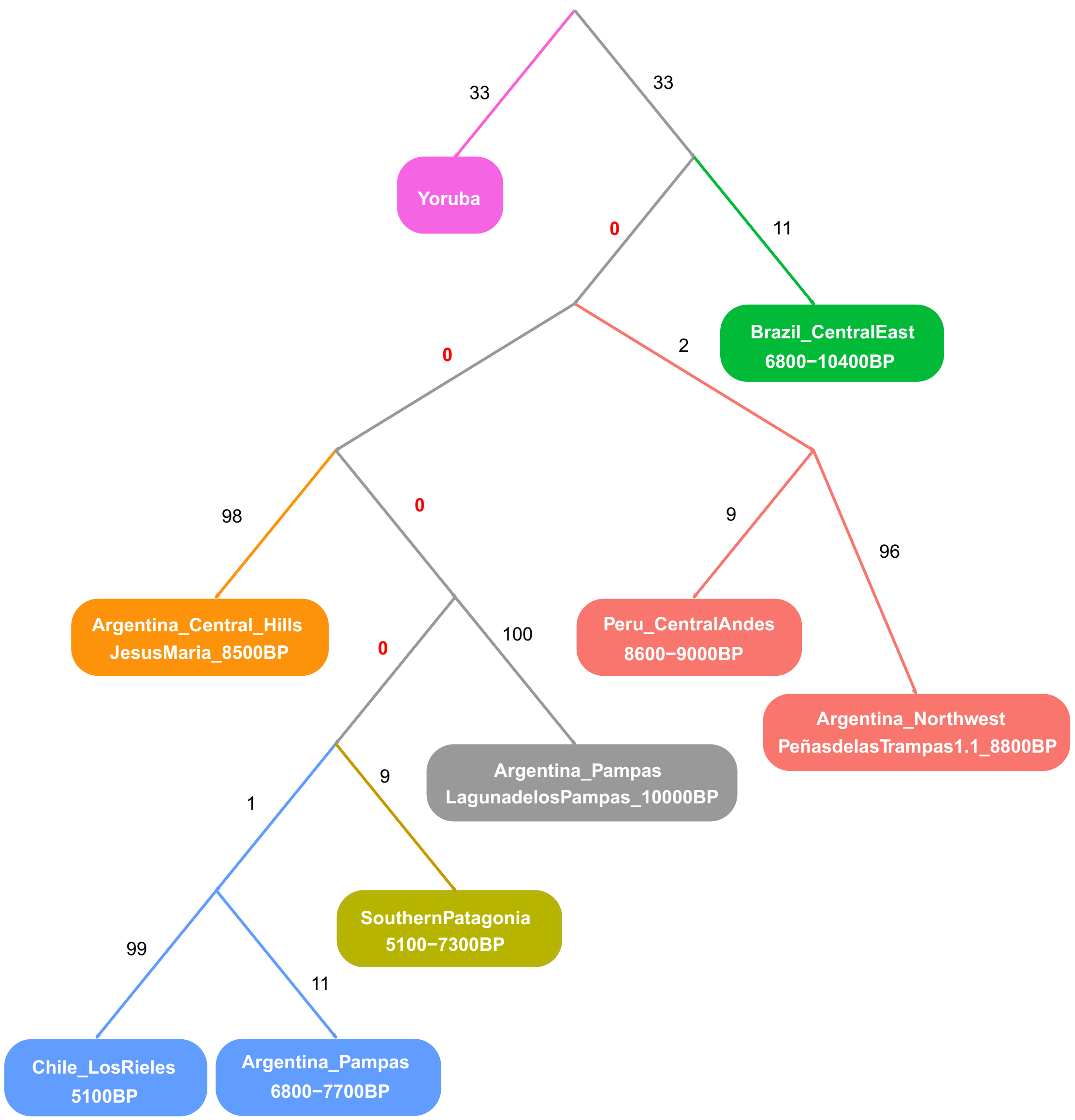

Supplementary Figure 7: Sixth of 9 unique trees identified by `find_graphs` involving Early/Middle Holocene South American individuals. Score ≈ 35.660, worst residual ≈ 2.864.

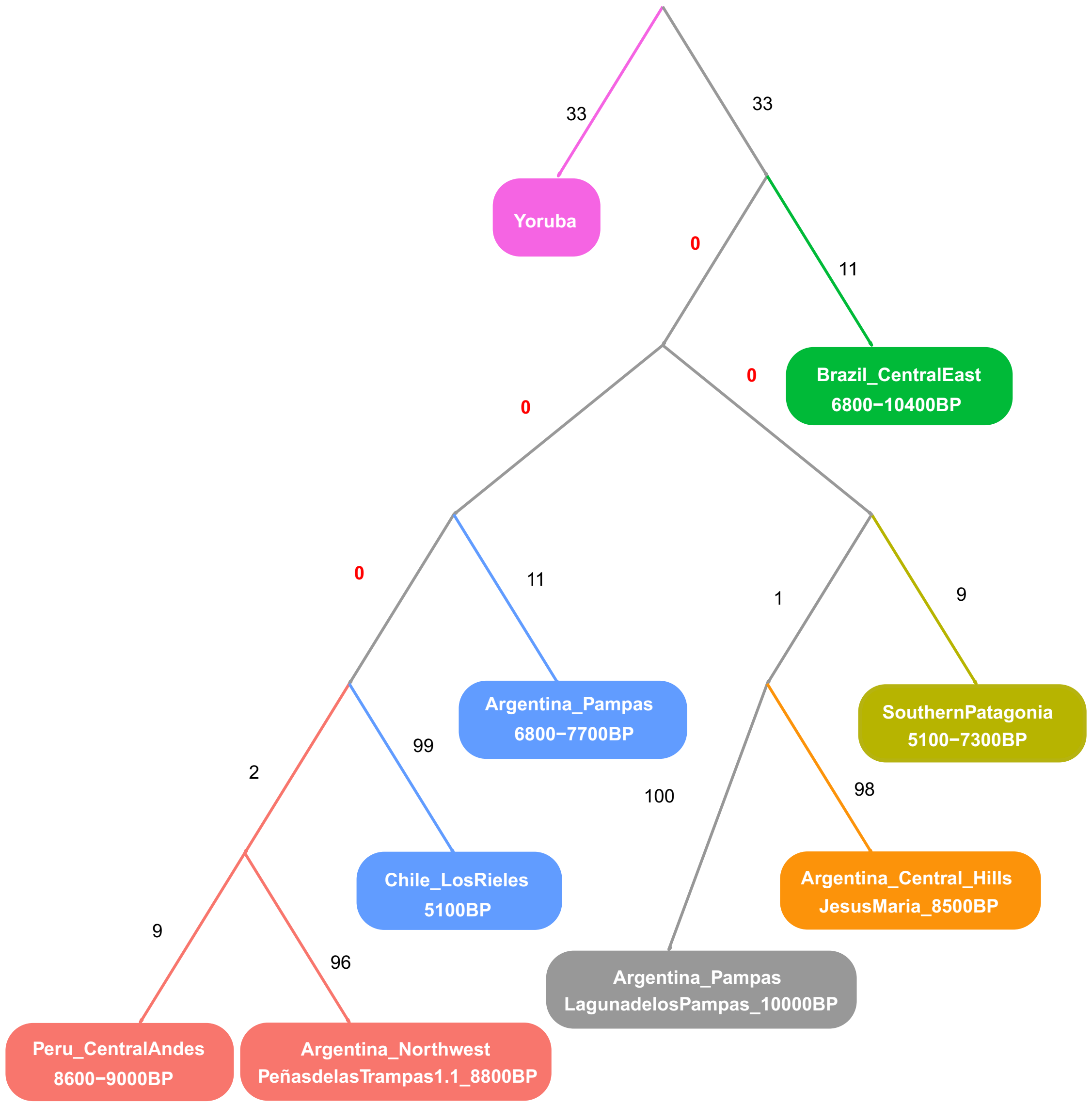

Supplementary Figure 8: Seventh of 9 unique trees identified by `find_graphs` involving Early/Middle Holocene South American individuals. Score ≈ 43.379, worst residual ≈ 4.750.

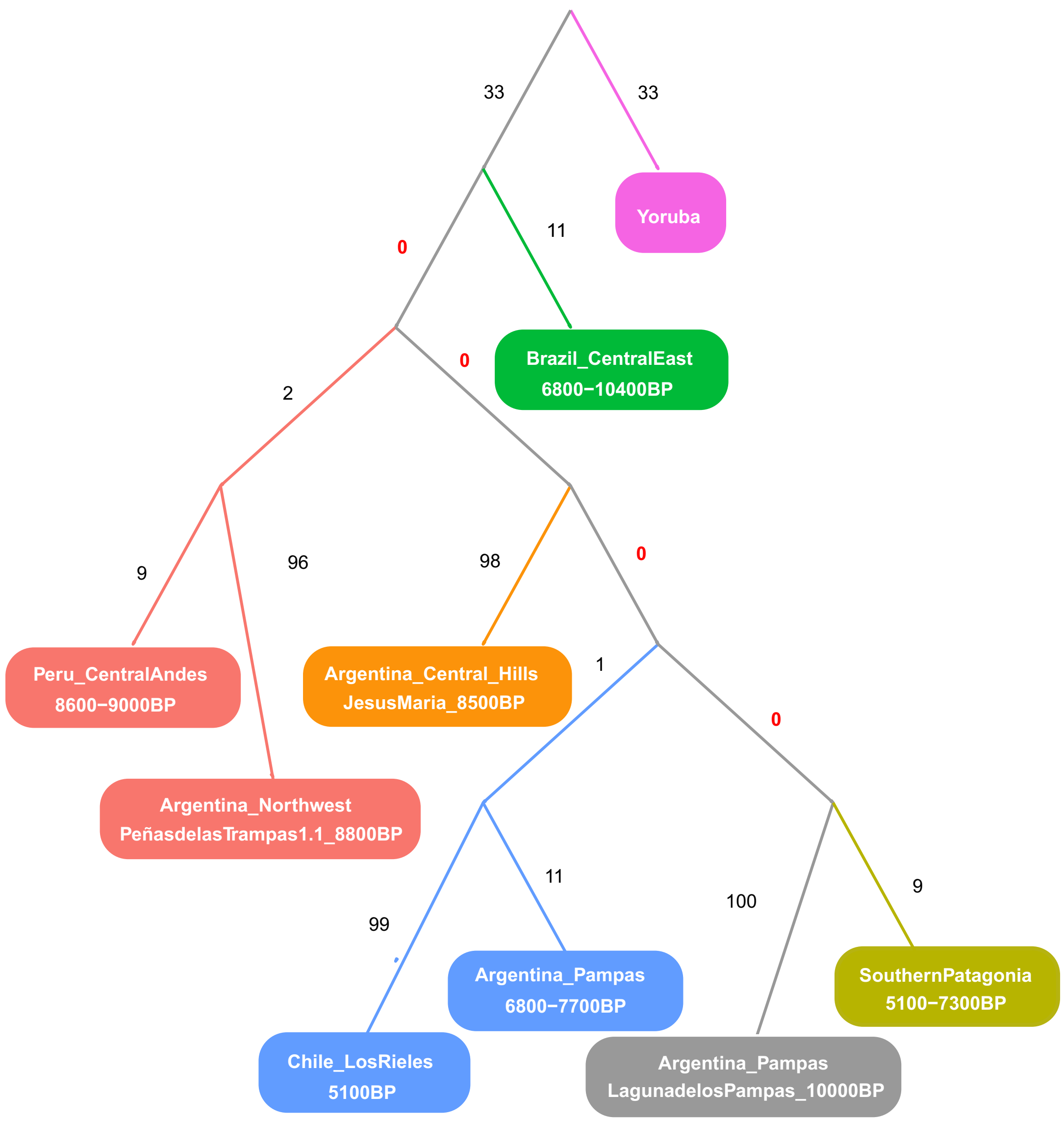

Supplementary Figure 9: Eighth of 9 unique trees identified by `find_graphs` involving Early/Middle Holocene South American individuals. Score ≈ 35.642, worst residual ≈ 2.864.

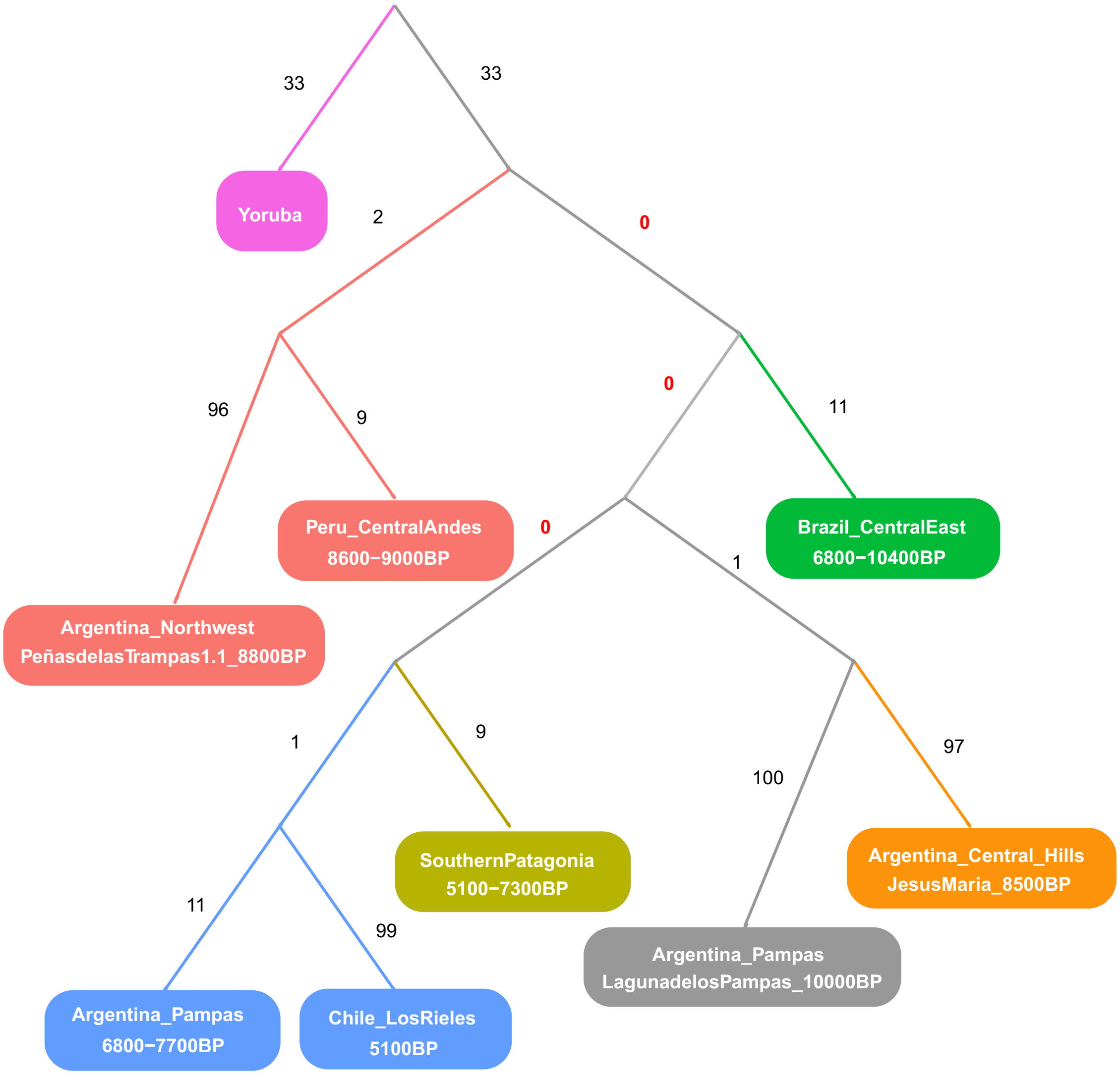

Supplementary Figure 10: Ninth of 9 unique trees identified by `find_graphs` involving Early/Middle Holocene South American individuals. Score ≈ 37.612, worst residual ≈ 3.545.

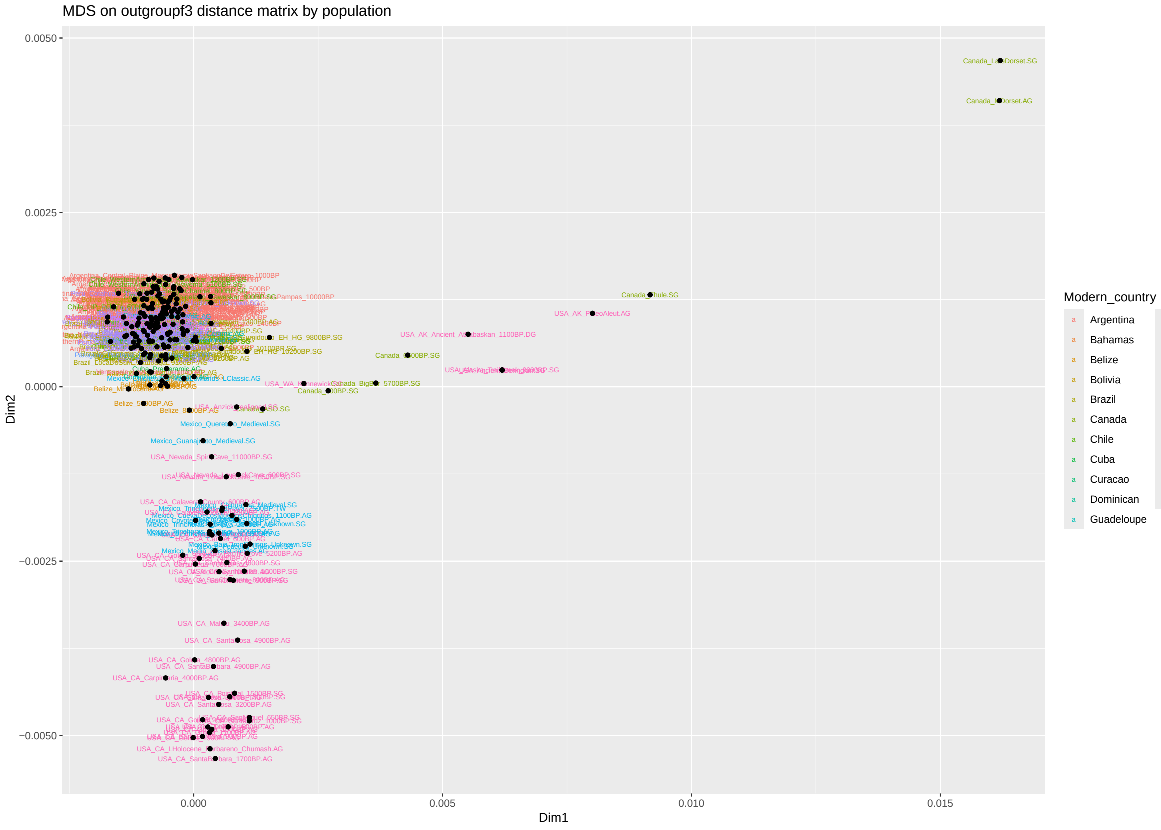

Supplementary Figure 11: $\text{MDS}_1\times$ MDS_3 plot on an outgroup$-f_3$ distance matrix of the form $1/f_3(\text{Pop}_1, \text{Pop}_2; \text{Yoruba})$, where Pop_i, $i \in \{1, 2\}$, is a newly-reported or previously-published ancient American context label.

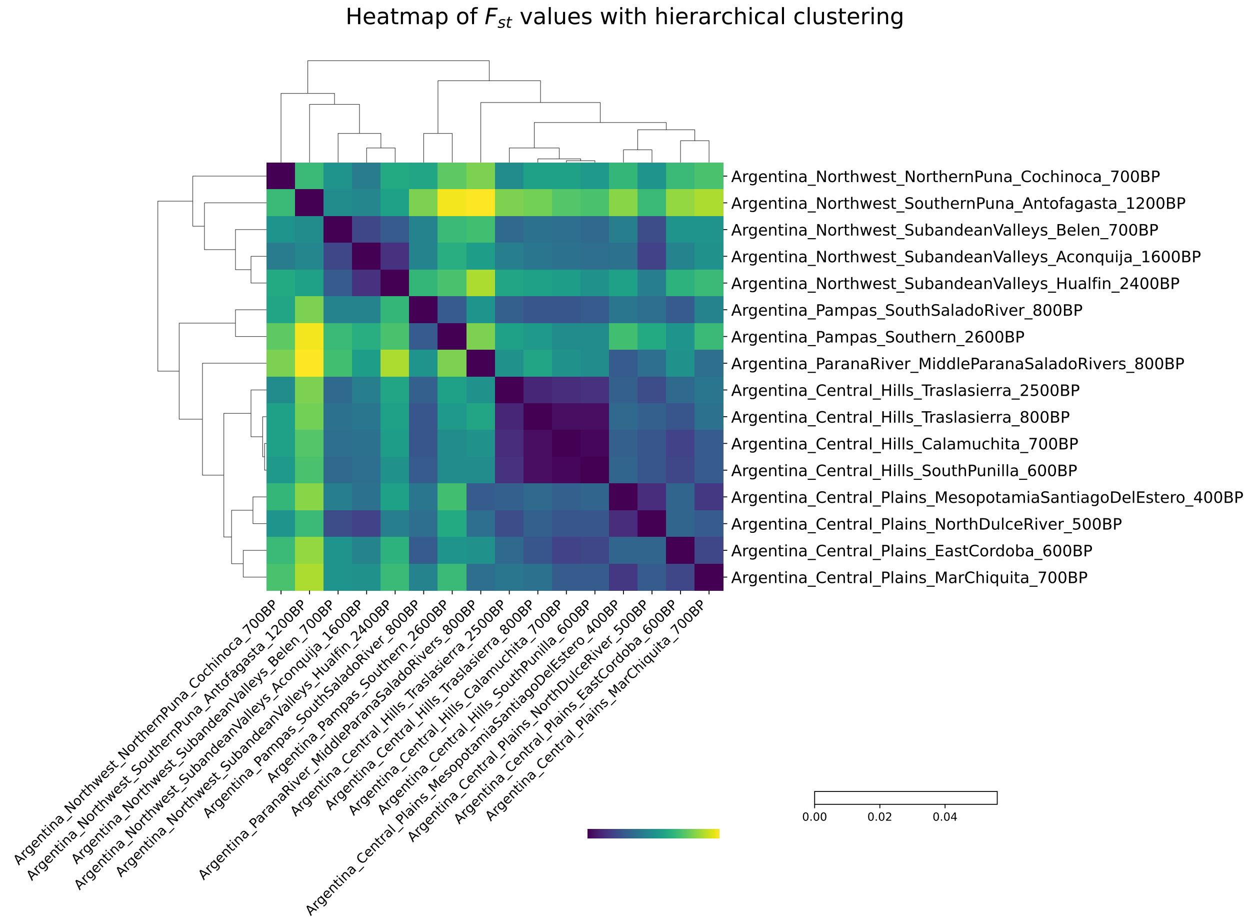

Supplementary Figure 12: Heatmap of F_{st} values, restricted to populations for which at least 5000 SNPs were used for all pairwise computations.

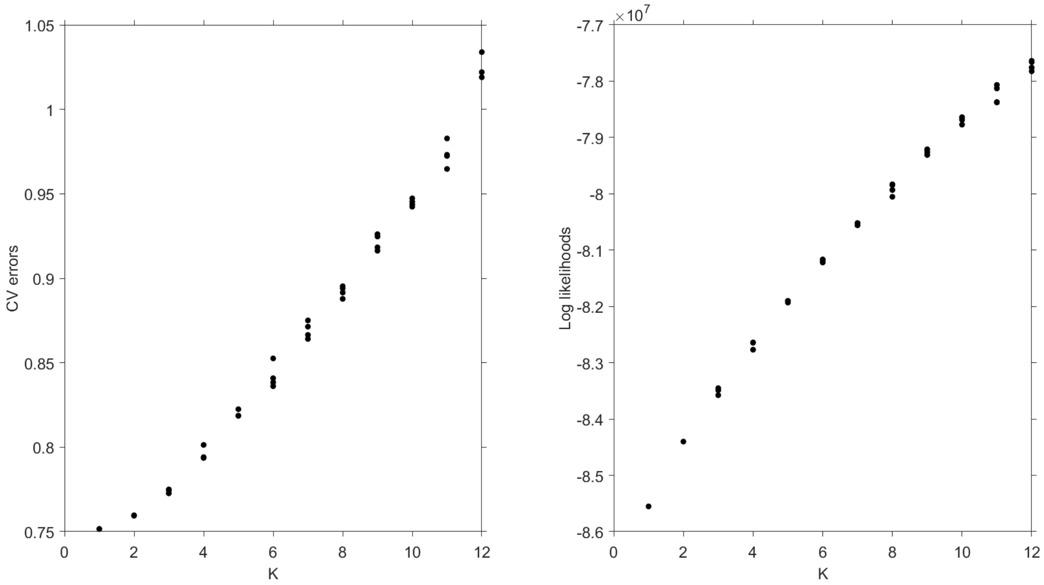

Supplementary Figure 13: ADMIXTURE cross-validation errors and log-likelihoods for $K = 1, \ldots, 12$, considering only ancient and modern [2] South American individuals. The monotonous increase in cross-validation error values with K indicates overall bad fit to the data. Full results for each K and each random iteration $i = 1, \ldots, 4$ for each K are available at https://github.com/javiermaravall/aDNA_CSC/.

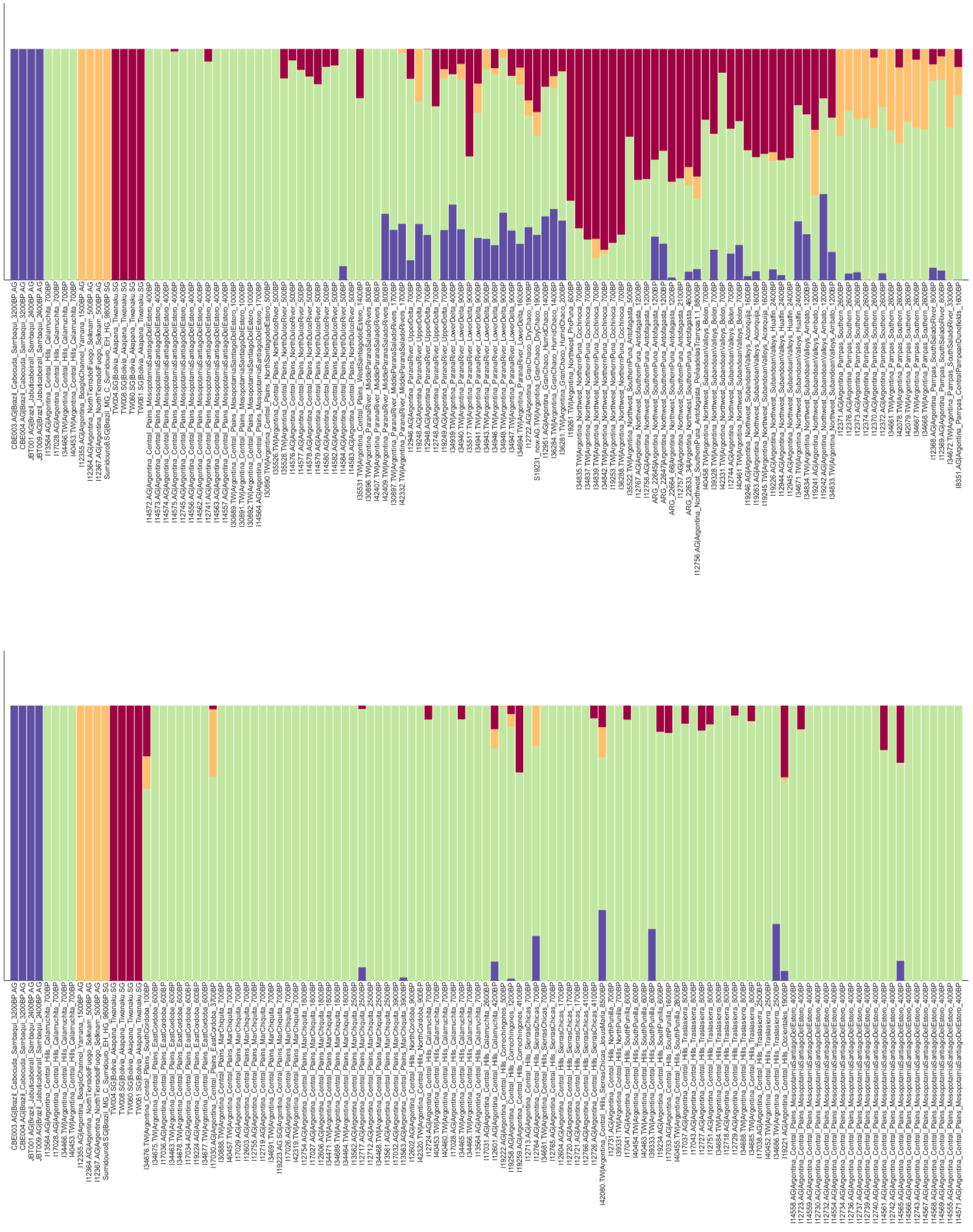

Supplementary Figure 14: ADMIXTURE component estimates for newly-reported individuals at $K = 4$ (random iteration $i = 1$).

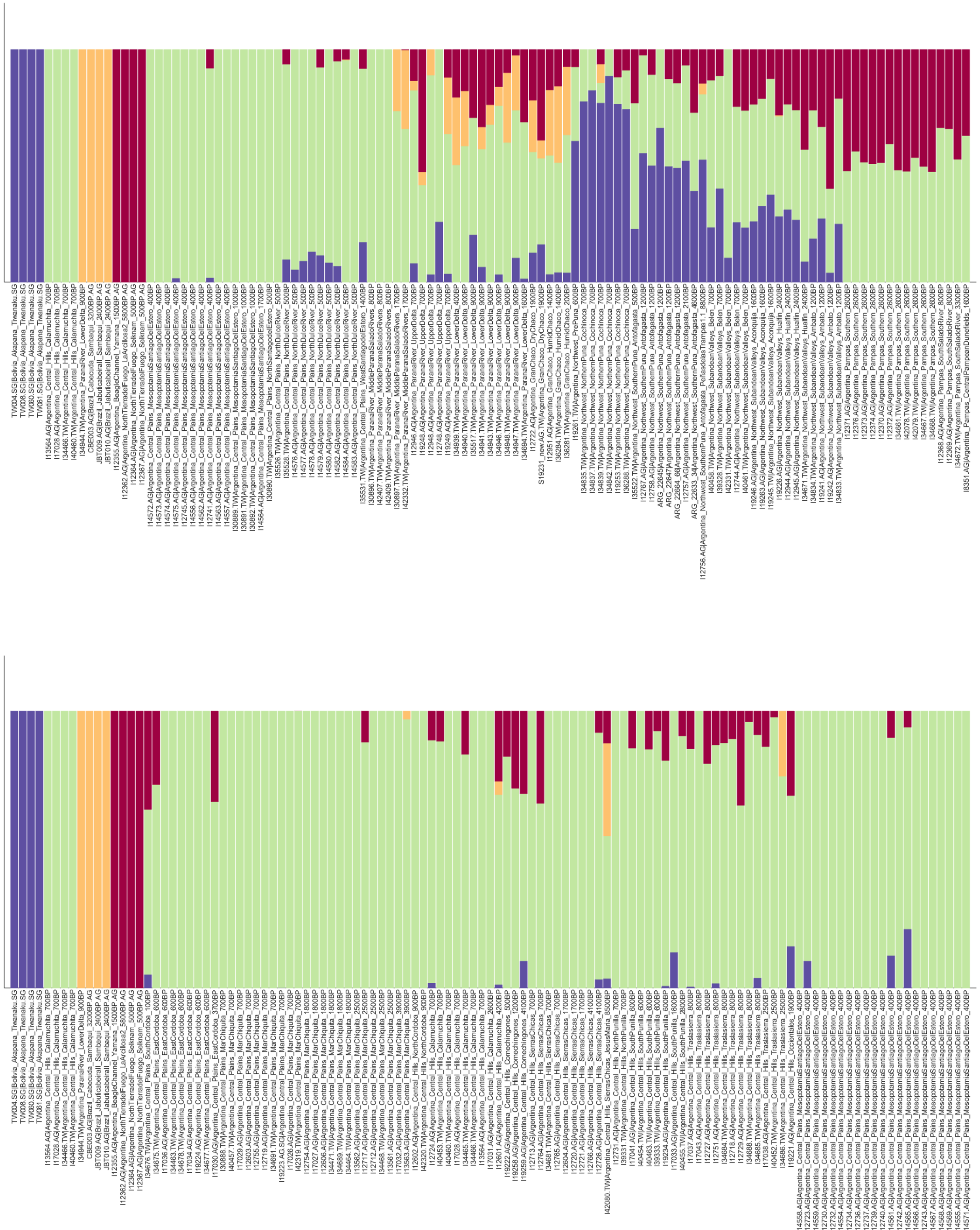

Supplementary Figure 15: ADMIXTURE component estimates for newly-reported individuals at $K = 4$ (random iteration $i = 2$).

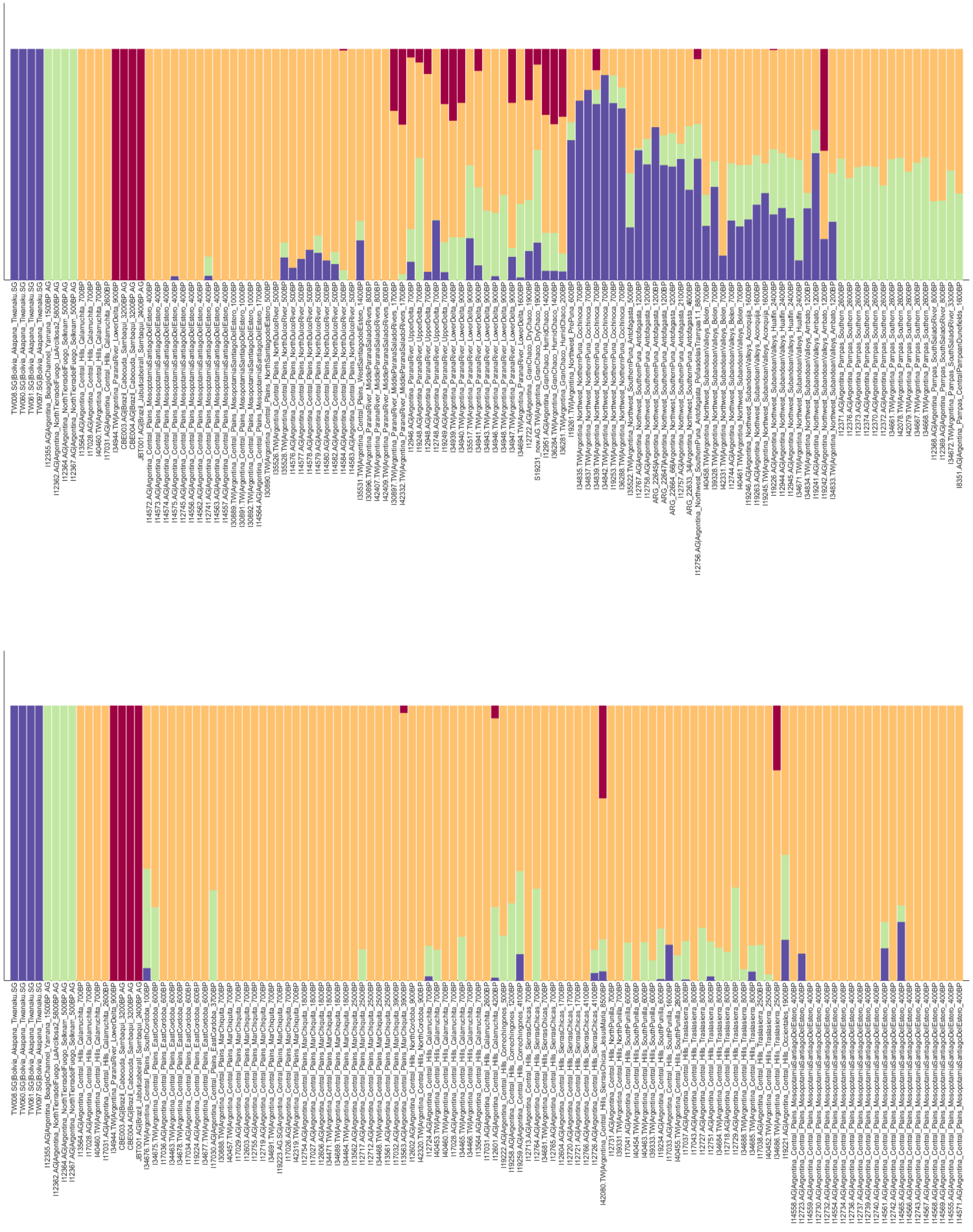

Supplementary Figure 16: ADMIXTURE component estimates for newly-reported individuals at $K = 4$ (random iteration $i = 3$).

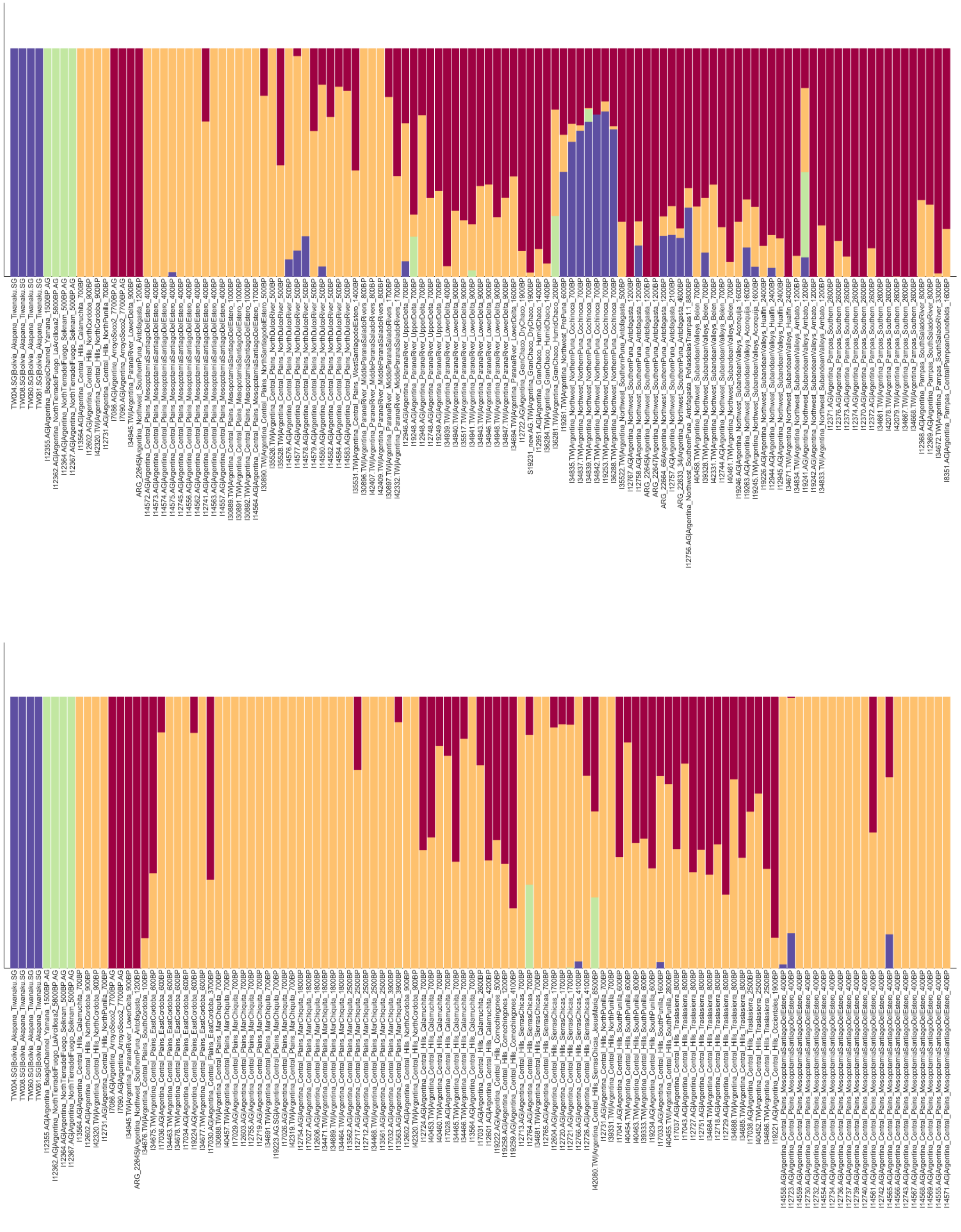

Supplementary Figure 17: ADMIXTURE component estimates for newly-reported individuals at $K = 4$ (random iteration $i = 4$).

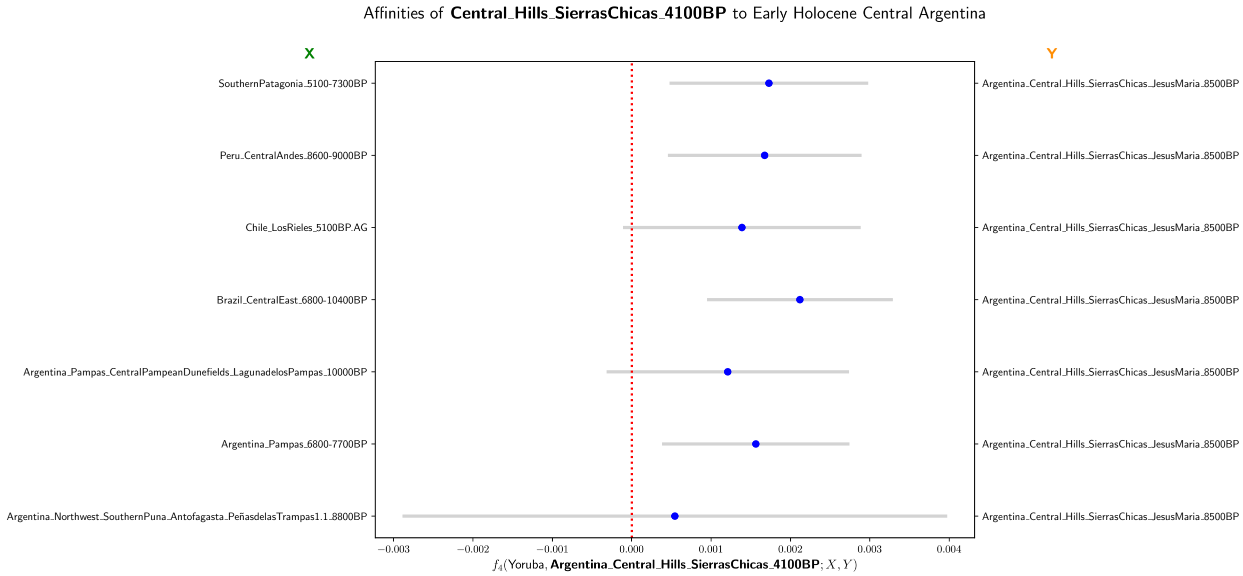

Supplementary Figure 18: Affinities of a representative 4100BP Central Argentina population to Early/Middle Holocene South American samples. Bars denote 95% confidence intervals (1.96× standard errors).

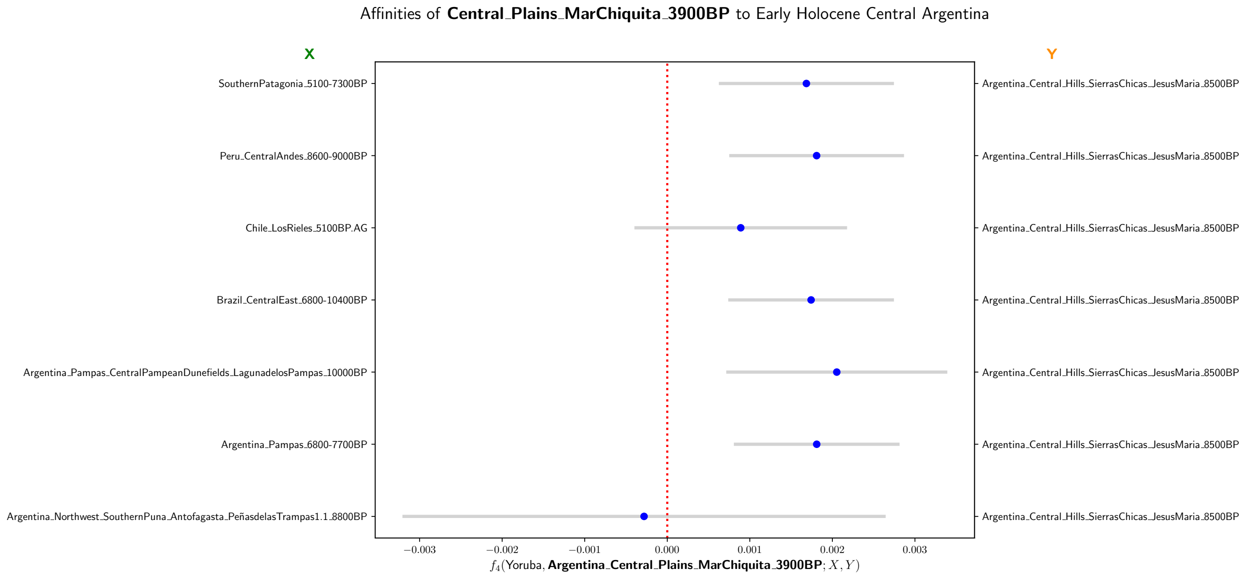

Supplementary Figure 19: Affinities of a representative 3900BP Central Argentina population to Early/Middle Holocene South American samples. Bars denote 95% confidence intervals (1.96× standard errors).

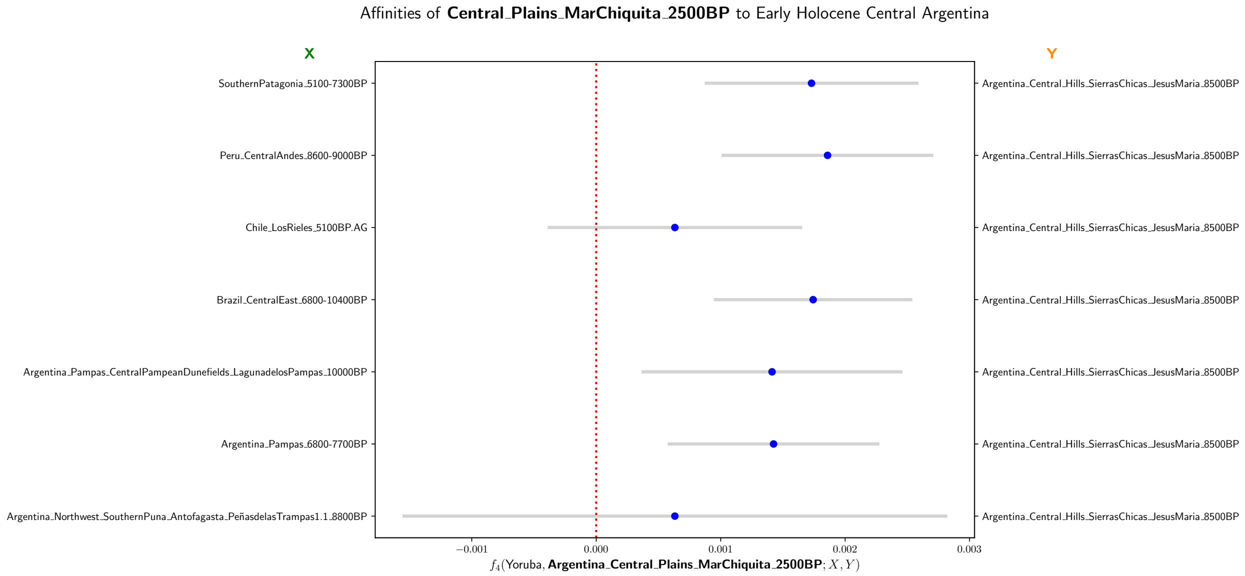

Supplementary Figure 20: Affinities of a representative 2500BP Central Argentina population to Early/Middle Holocene South American samples. Bars denote 95% confidence intervals (1.96× standard errors).

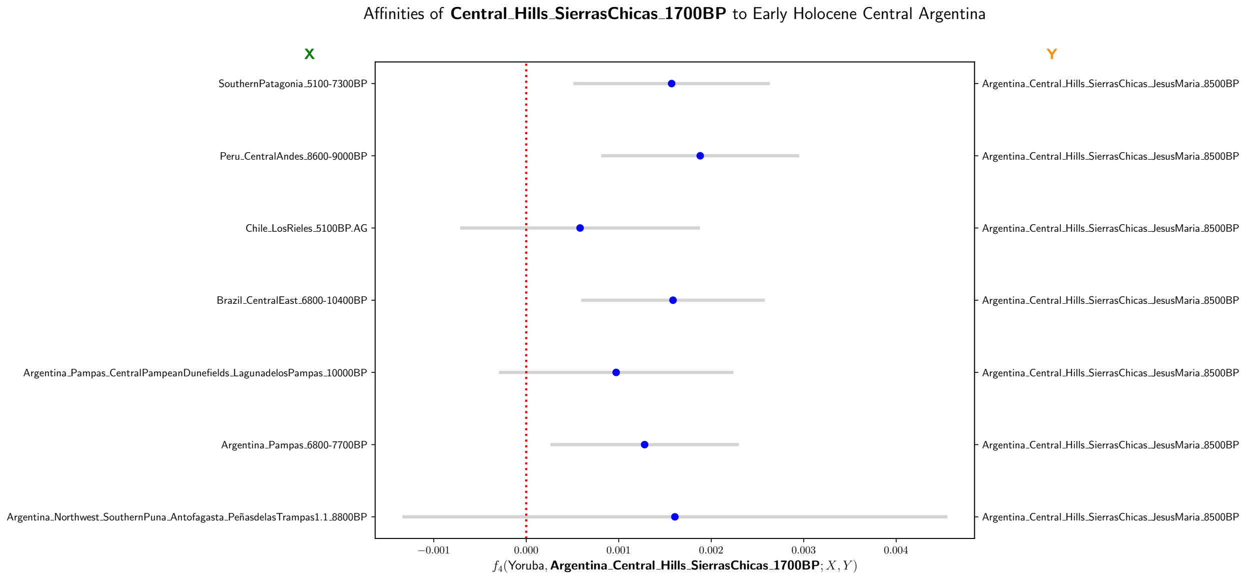

Supplementary Figure 21: Affinities of a representative 1700BP Central Argentina population to Early/Middle Holocene South American samples. Bars denote 95% confidence intervals (1.96× standard errors).

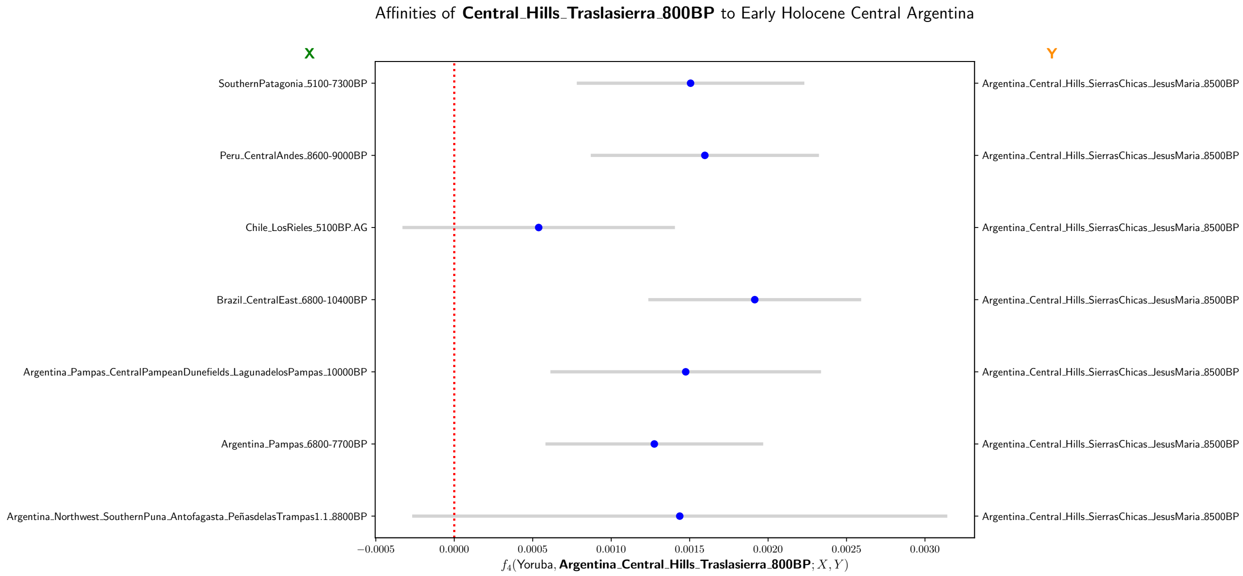

Supplementary Figure 22: Affinities of a representative 800BP Central Argentina population to Early/Middle Holocene South American samples. Bars denote 95% confidence intervals (1.96× standard errors).

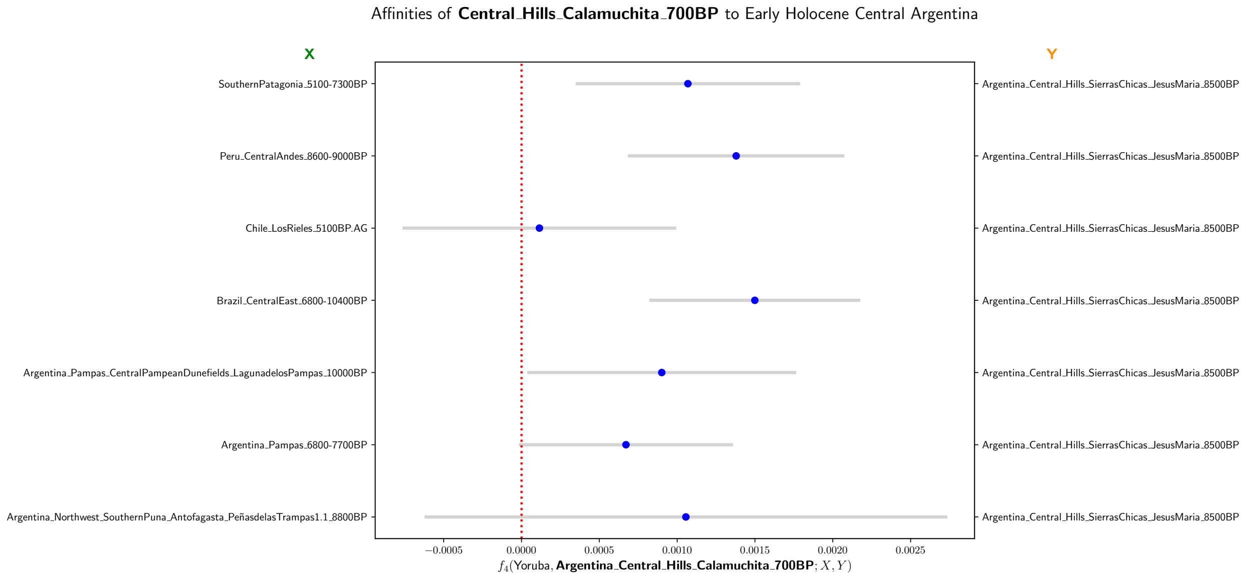

Supplementary Figure 23: Affinities of a representative 700BP Central Argentina population to Early/Middle Holocene South American samples. Bars denote 95% confidence intervals (1.96× standard errors).

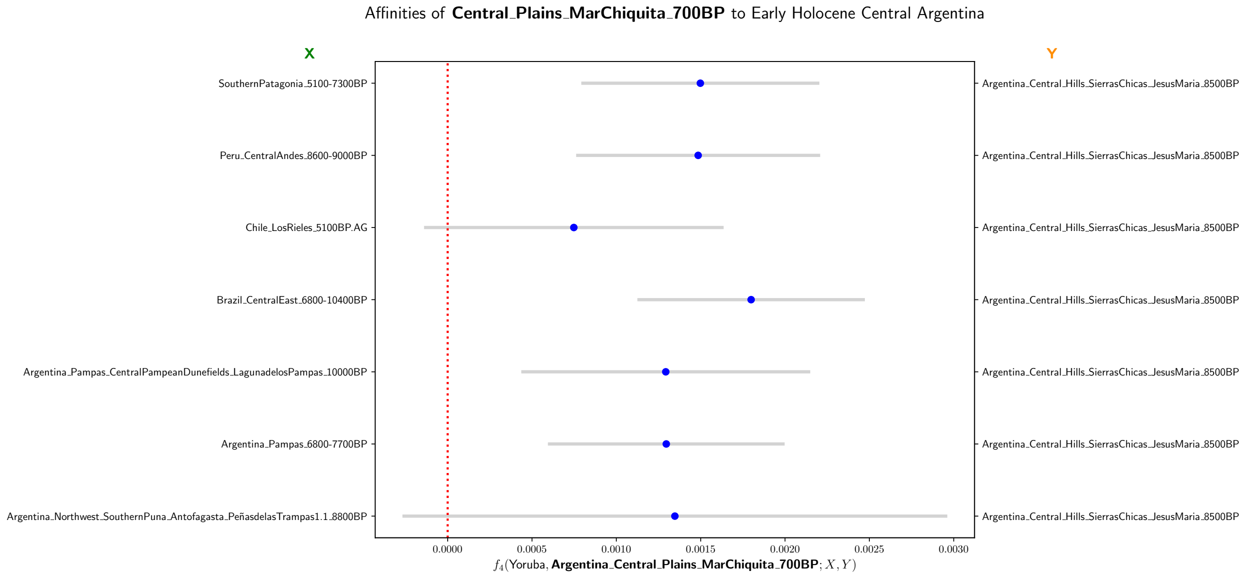

Supplementary Figure 24: Affinities of a representative 700BP Central Argentina population to Early/Middle Holocene South American samples. Bars denote 95% confidence intervals (1.96× standard errors).

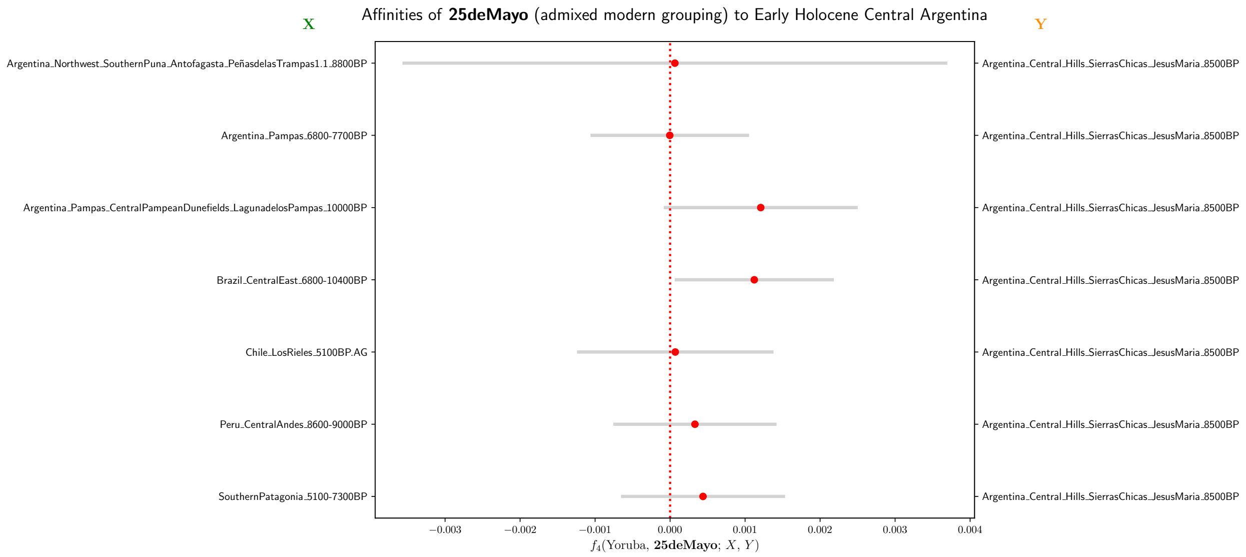

Supplementary Figure 25: Affinities of a modern Central Argentina admixed grouping from [1] to Early/Middle Holocene South American samples. Red dots denote tests with fewer than 75000 SNPs available. Bars denote 95% confidence intervals (1.96× standard errors).

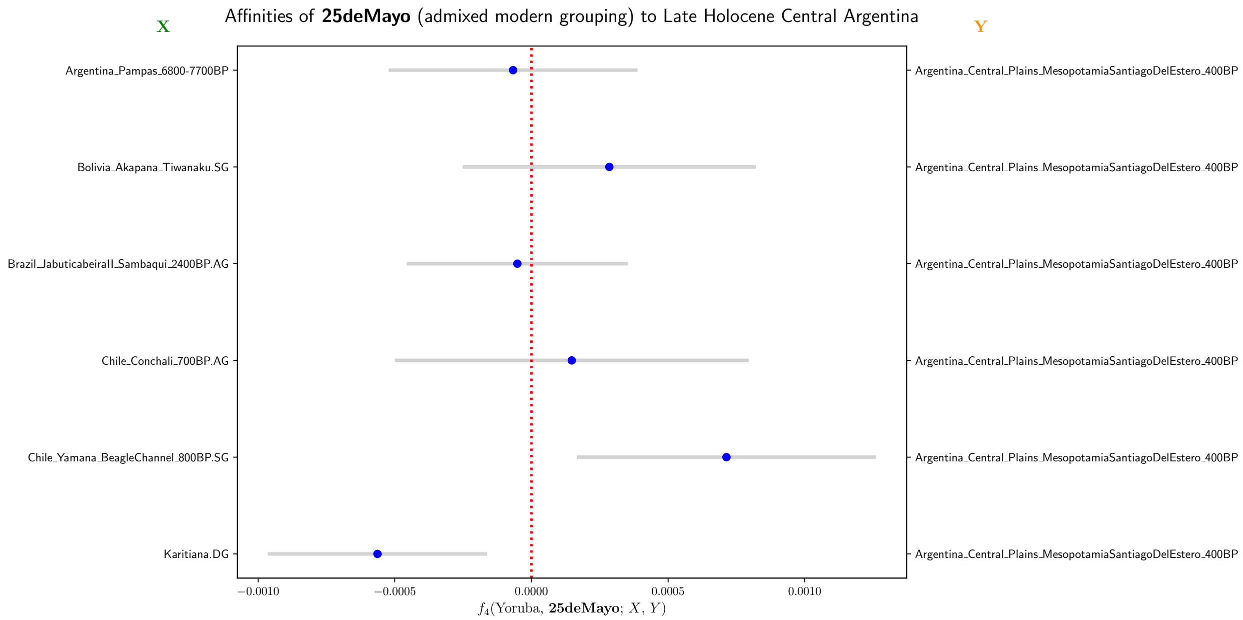

Supplementary Figure 26: Affinities of a modern Central Argentina admixed grouping from [1] to Late Holocene South American samples. Bars denote 95% confidence intervals (1.96× standard errors).

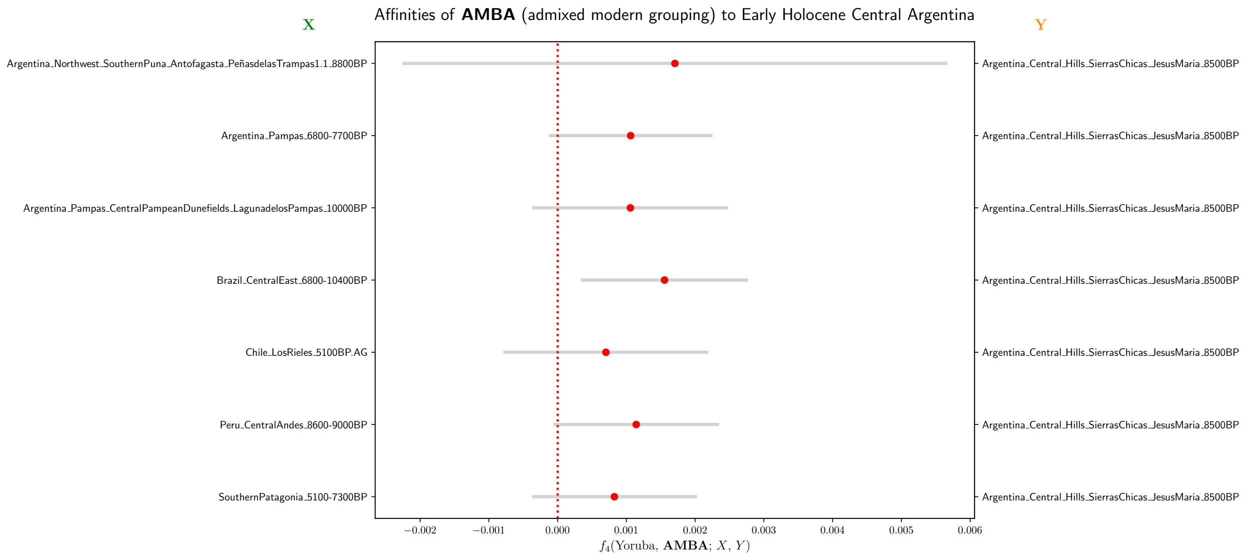

Supplementary Figure 27: Affinities of a modern Central Argentina admixed grouping from [1] to Early/Middle Holocene South American samples. Red dots denote tests with fewer than 75000 SNPs available. Bars denote 95% confidence intervals (1.96× standard errors).

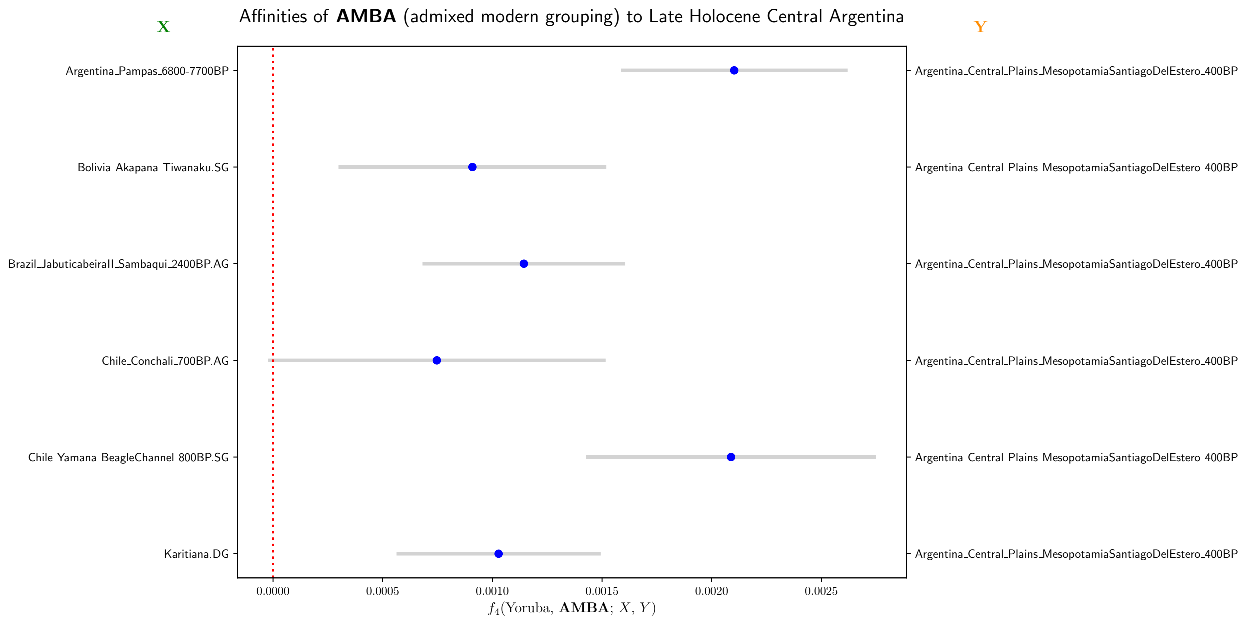

Supplementary Figure 28: Affinities of a modern Central Argentina admixed grouping from [1] to Late Holocene South American samples. Bars denote 95% confidence intervals (1.96× standard errors).

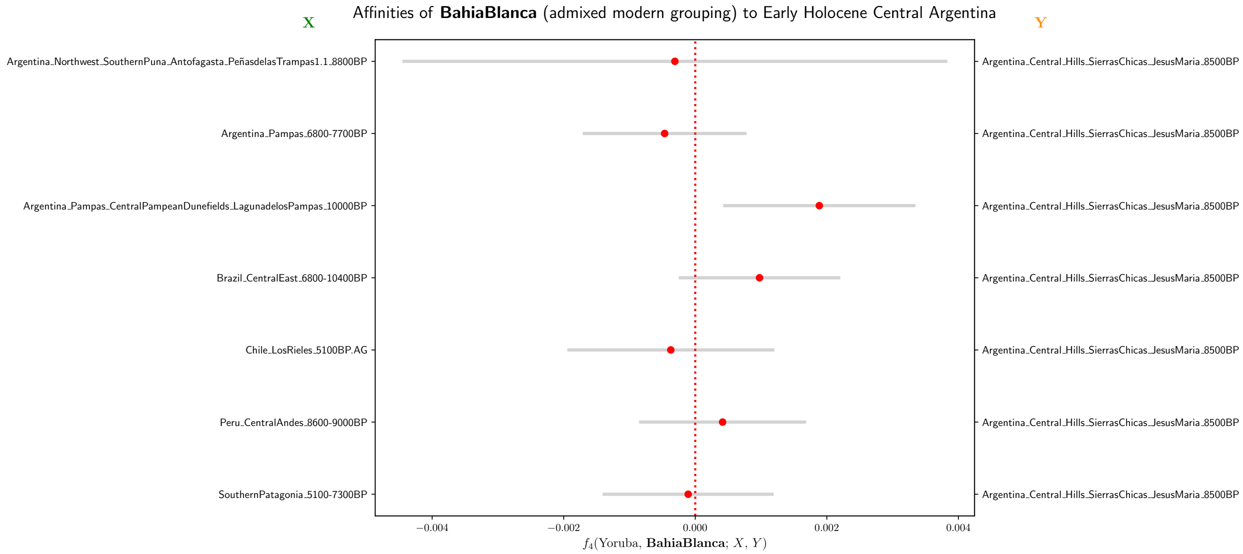

Supplementary Figure 29: Affinities of a modern Central Argentina admixed grouping from [1] to Early/Middle Holocene South American samples. Red dots denote tests with fewer than 75000 SNPs available. Bars denote 95% confidence intervals (1.96× standard errors).

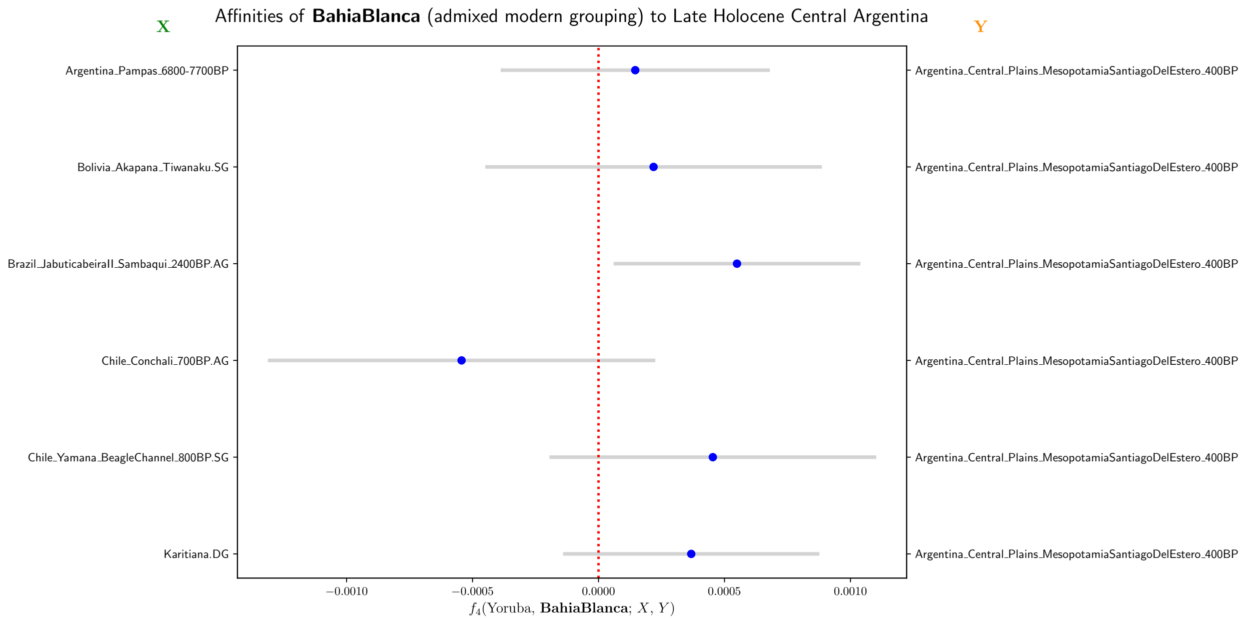

Supplementary Figure 30: Affinities of a modern Central Argentina admixed grouping from [1] to Late Holocene South American samples. Bars denote 95% confidence intervals (1.96× standard errors).

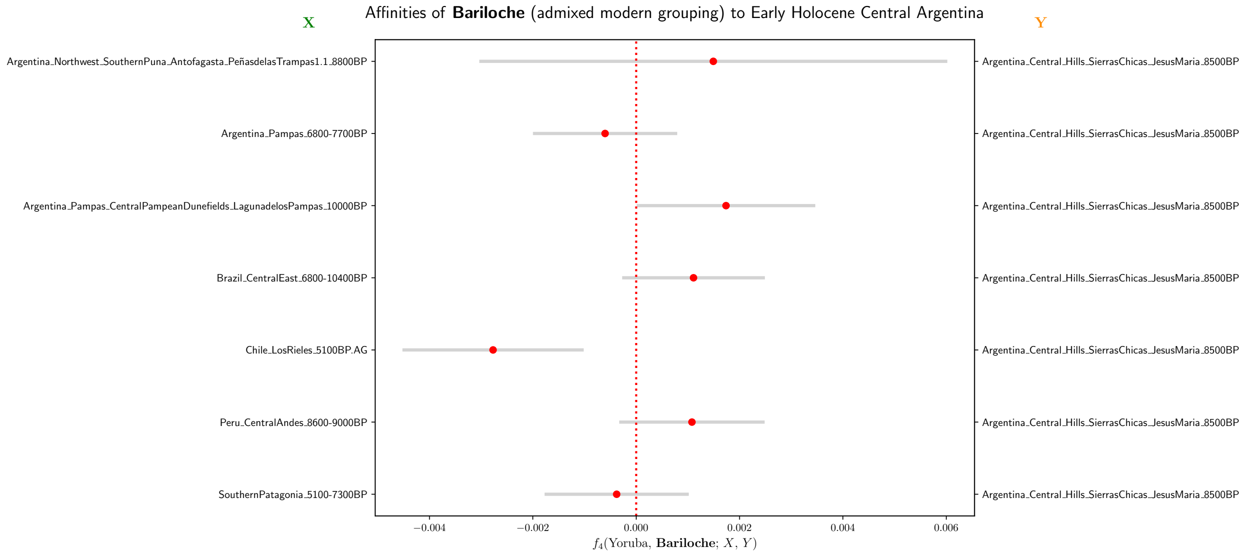

Supplementary Figure 31: Affinities of a modern Central Argentina admixed grouping from [1] to Early/Middle Holocene South American samples. Red dots denote tests with fewer than 75000 SNPs available. Bars denote 95% confidence intervals (1.96× standard errors).

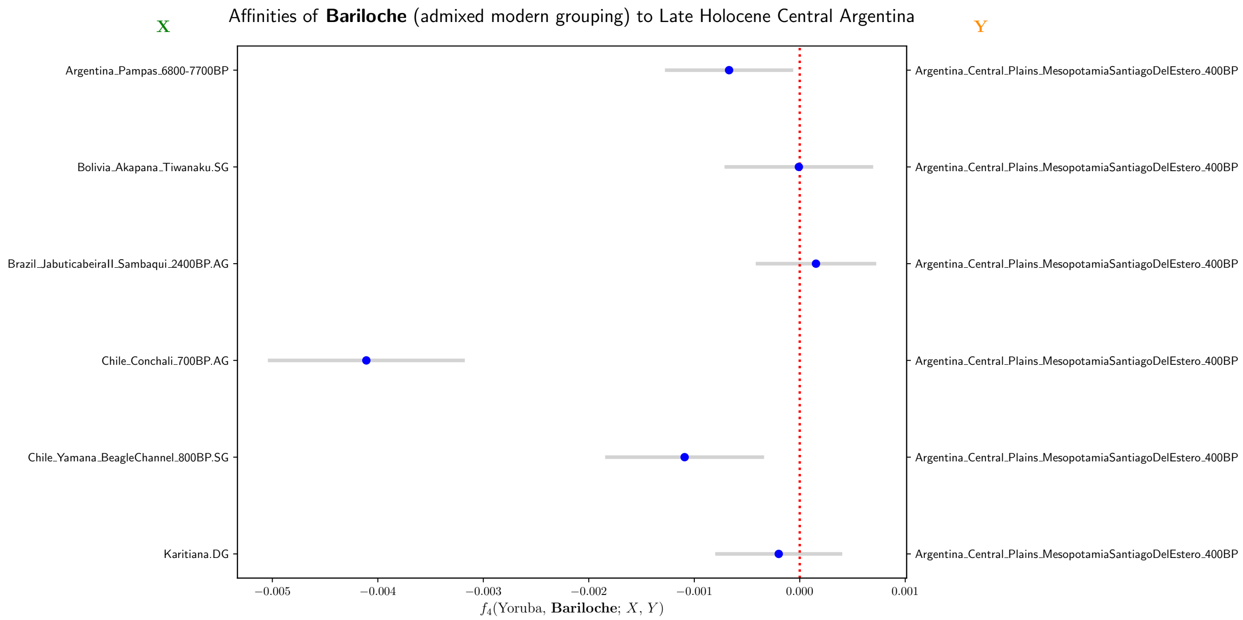

Supplementary Figure 32: Affinities of a modern Central Argentina admixed grouping from [1] to Late Holocene South American samples. Bars denote 95% confidence intervals (1.96× standard errors).

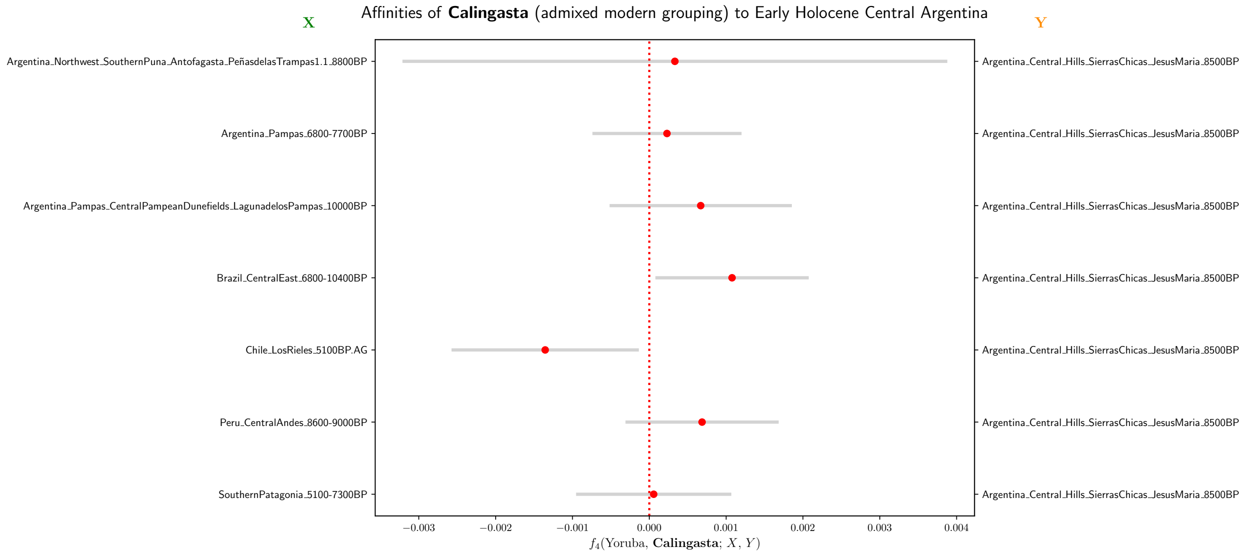

Supplementary Figure 33: Affinities of a modern Central Argentina admixed grouping from [1] to Early/Middle Holocene South American samples. Red dots denote tests with fewer than 75000 SNPs available. Bars denote 95% confidence intervals (1.96× standard errors).

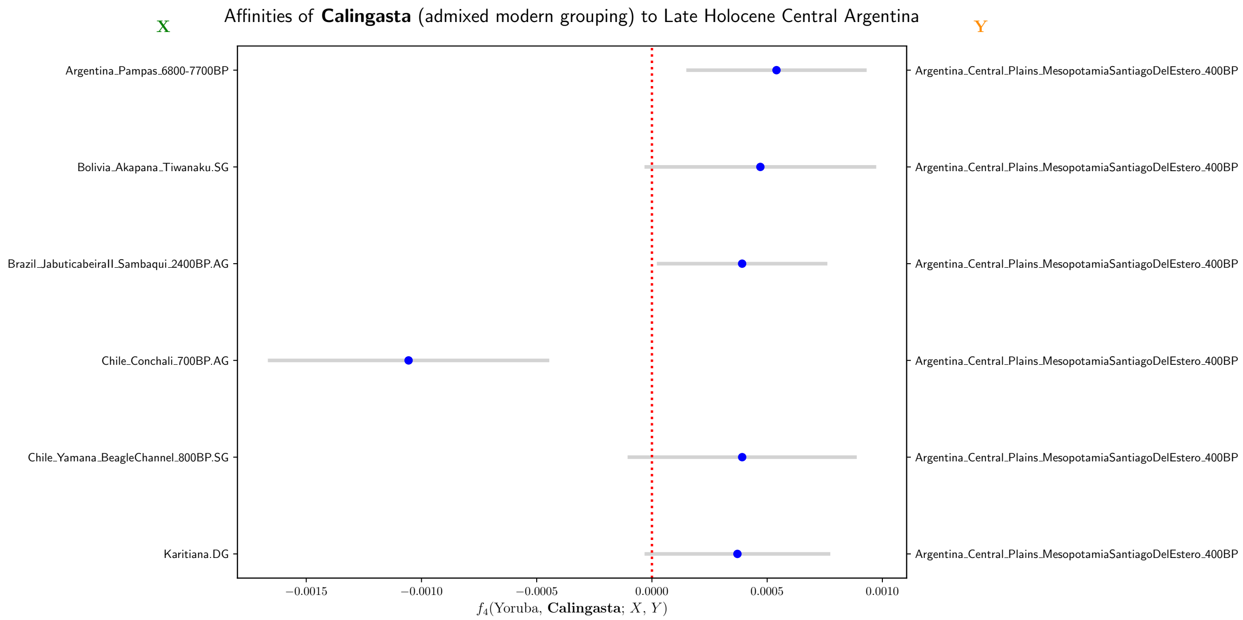

Supplementary Figure 34: Affinities of a modern Central Argentina admixed grouping from [1] to Late Holocene South American samples. Bars denote 95% confidence intervals (1.96× standard errors).

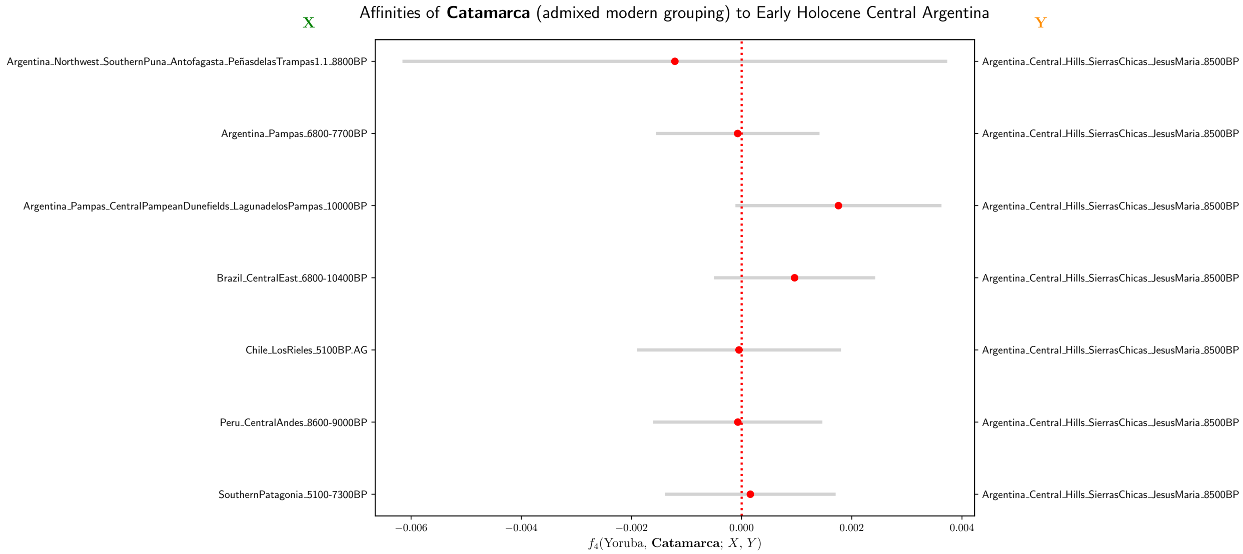

Supplementary Figure 35: Affinities of a modern Central Argentina admixed grouping from [1] to Early/Middle Holocene South American samples. Red dots denote tests with fewer than 75000 SNPs available. Bars denote 95% confidence intervals (1.96× standard errors).

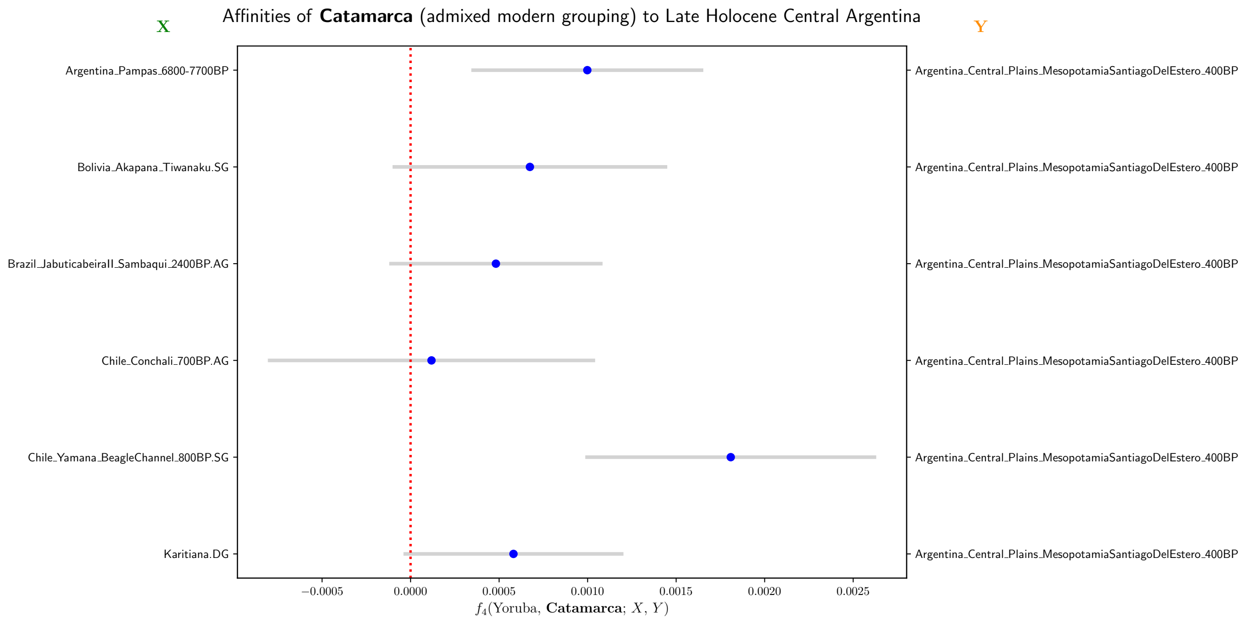

Supplementary Figure 36: Affinities of a modern Central Argentina admixed grouping from [1] to Late Holocene South American samples. Bars denote 95% confidence intervals (1.96× standard errors).

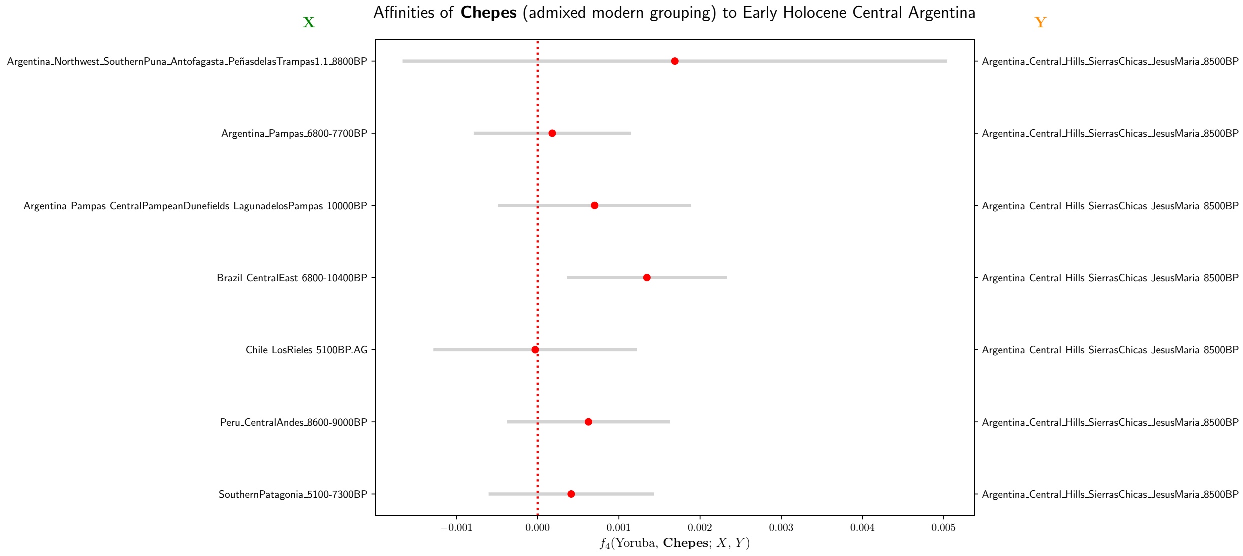

Supplementary Figure 37: Affinities of a modern Central Argentina admixed grouping from [1] to Early/Middle Holocene South American samples. Red dots denote tests with fewer than 75000 SNPs available. Bars denote 95% confidence intervals (1.96× standard errors).

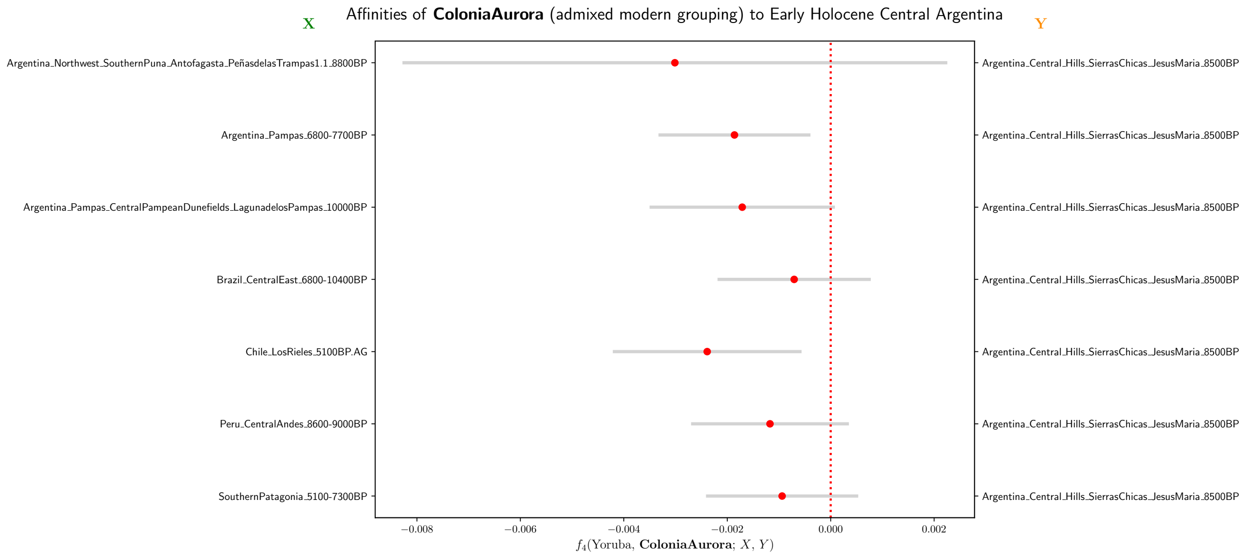

Supplementary Figure 38: Affinities of a modern Central Argentina admixed grouping from [1] to Early/Middle Holocene South American samples. Red dots denote tests with fewer than 75000 SNPs available. Bars denote 95% confidence intervals (1.96× standard errors).

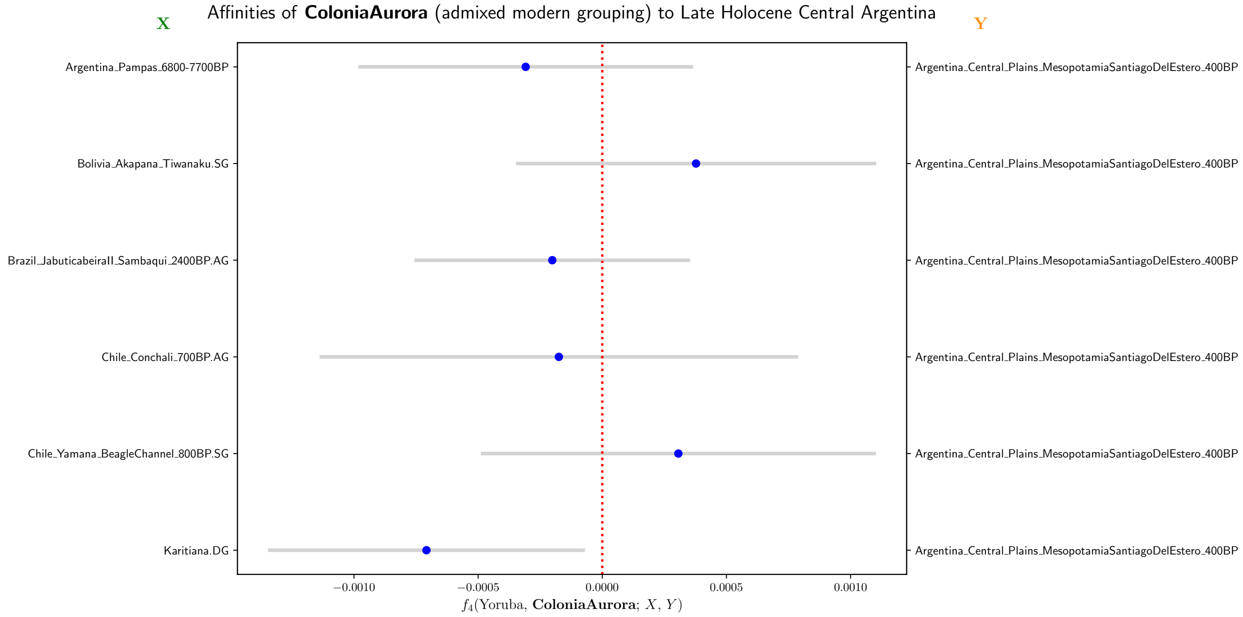

Supplementary Figure 39: Affinities of a modern Central Argentina admixed grouping from [1] to Late Holocene South American samples. Bars denote 95% confidence intervals (1.96× standard errors).

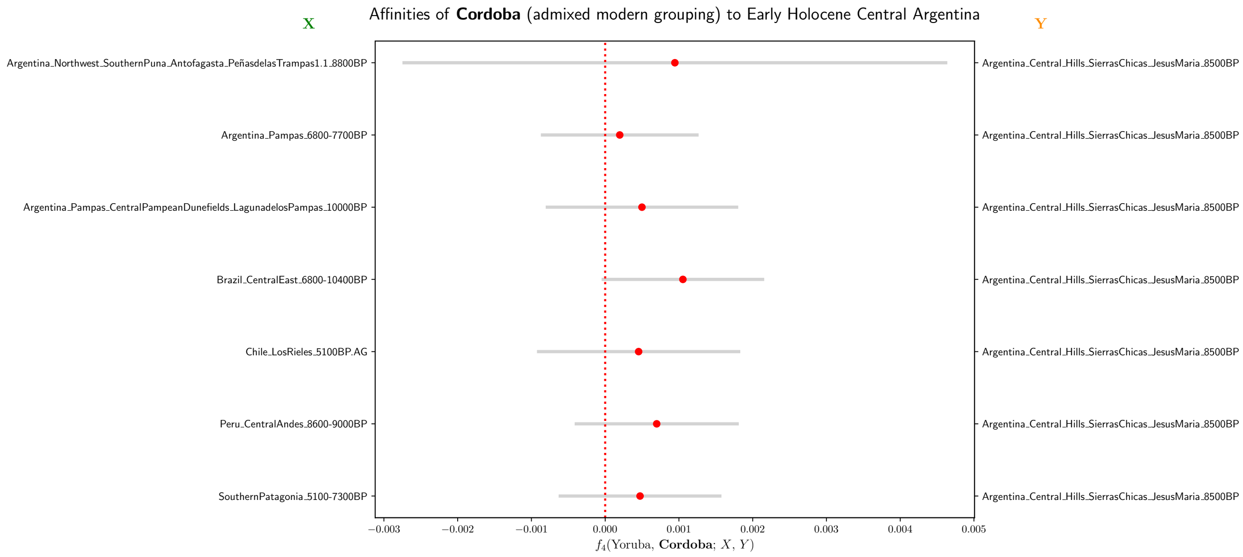

Supplementary Figure 40: Affinities of a modern Central Argentina admixed grouping from [1] to Early/Middle Holocene South American samples. Red dots denote tests with fewer than 75000 SNPs available. Bars denote 95% confidence intervals (1.96× standard errors).

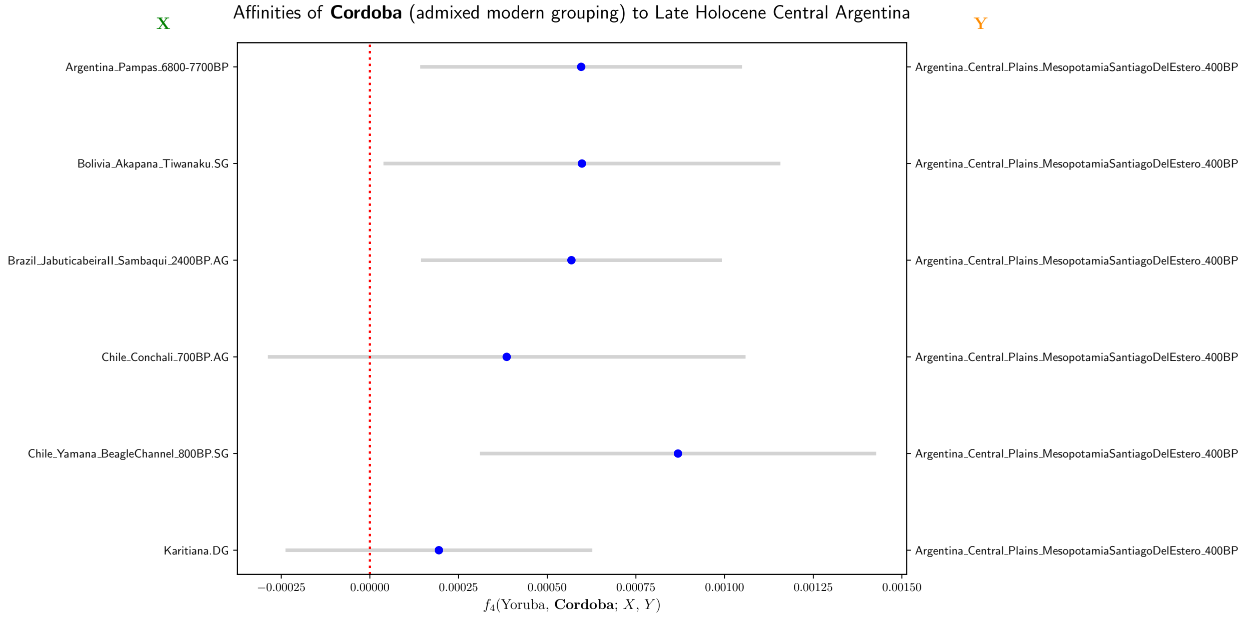

Supplementary Figure 41: Affinities of a modern Central Argentina admixed grouping from [1] to Late Holocene South American samples. Bars denote 95% confidence intervals (1.96× standard errors).

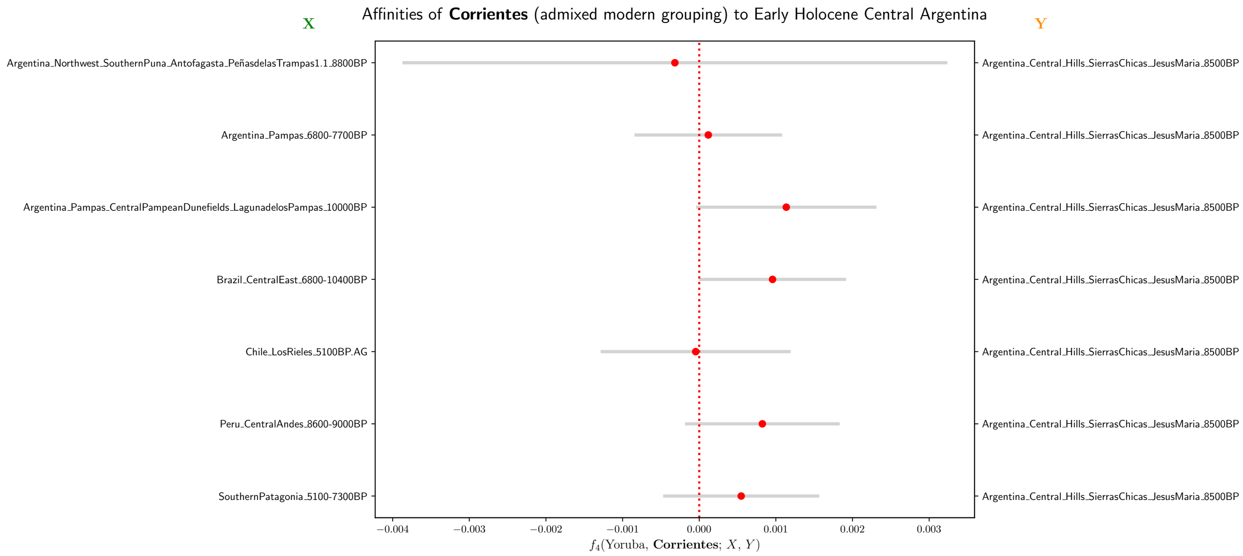

Supplementary Figure 42: Affinities of a modern Central Argentina admixed grouping from [1] to Early/Middle Holocene South American samples. Red dots denote tests with fewer than 75000 SNPs available. Bars denote 95% confidence intervals (1.96× standard errors).

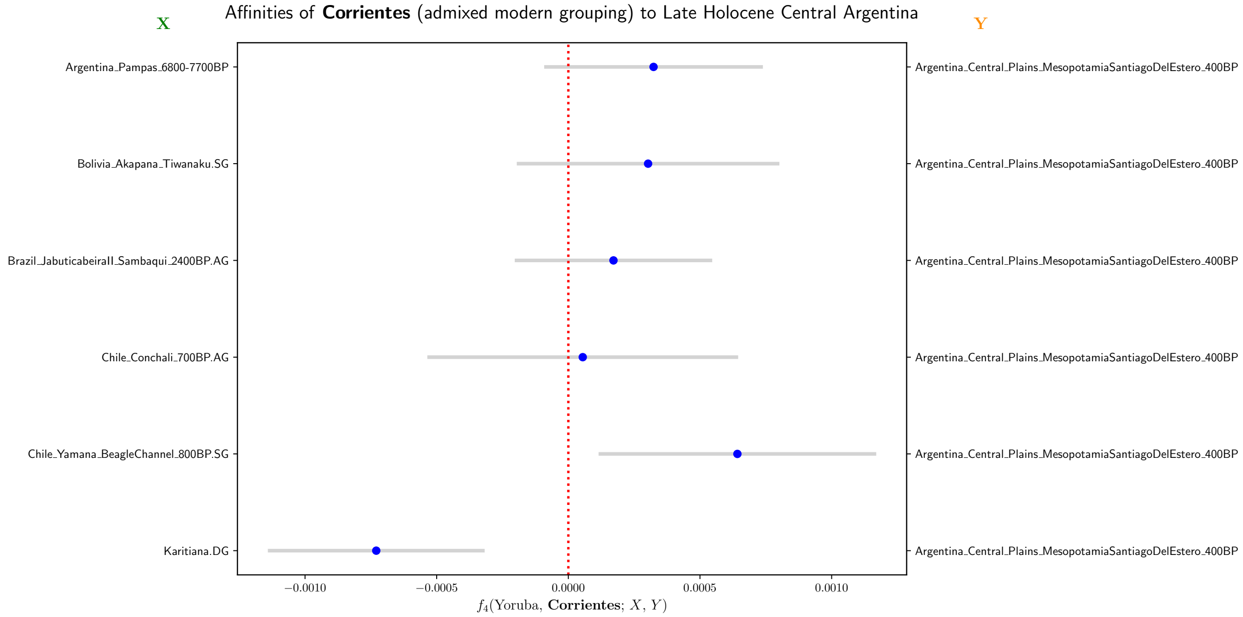

Supplementary Figure 43: Affinities of a modern Central Argentina admixed grouping from [1] to Late Holocene South American samples. Bars denote 95% confidence intervals (1.96× standard errors).

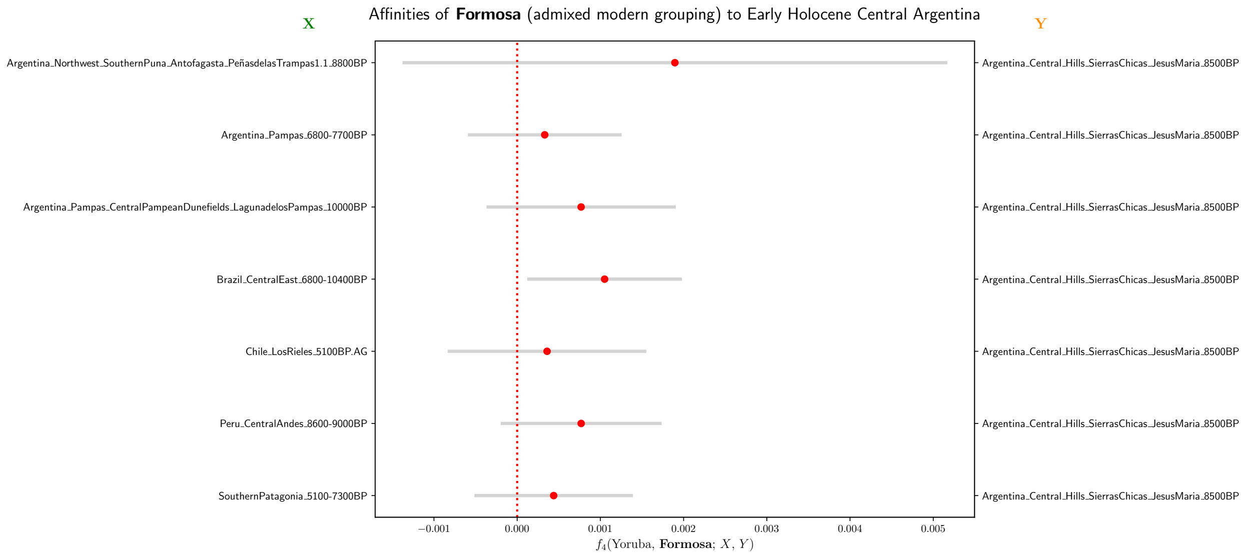

Supplementary Figure 44: Affinities of a modern Central Argentina admixed grouping from [1] to Early/Middle Holocene South American samples. Red dots denote tests with fewer than 75000 SNPs available. Bars denote 95% confidence intervals (1.96× standard errors).

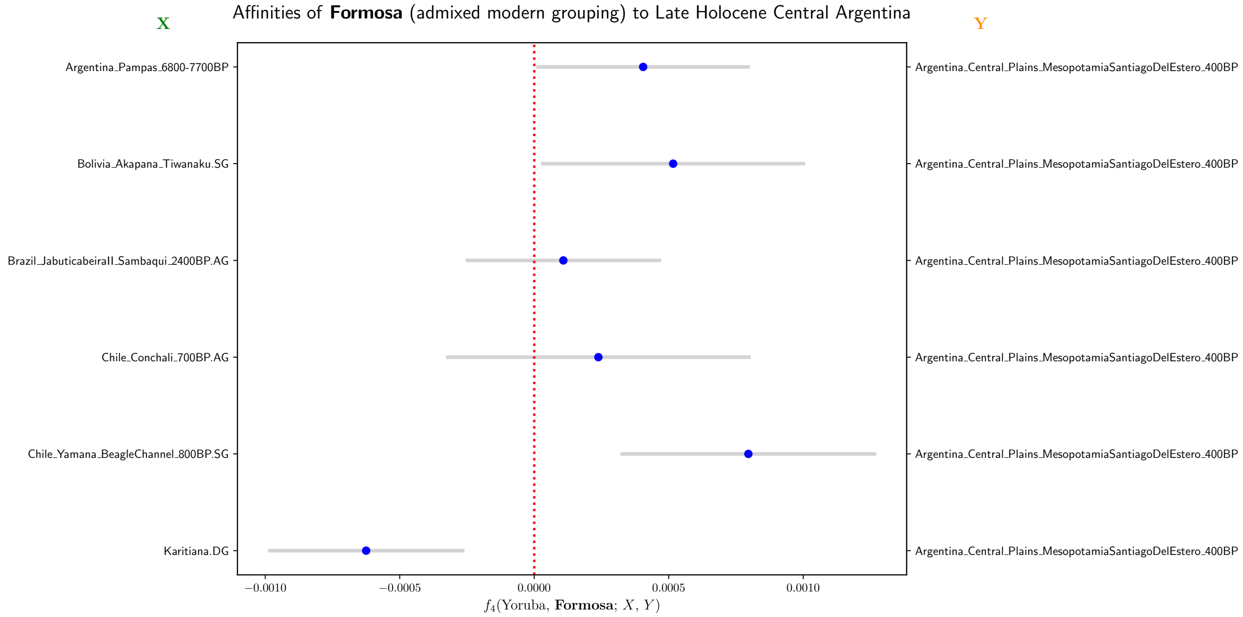

Supplementary Figure 45: Affinities of a modern Central Argentina admixed grouping from [1] to Late Holocene South American samples. Bars denote 95% confidence intervals (1.96× standard errors).

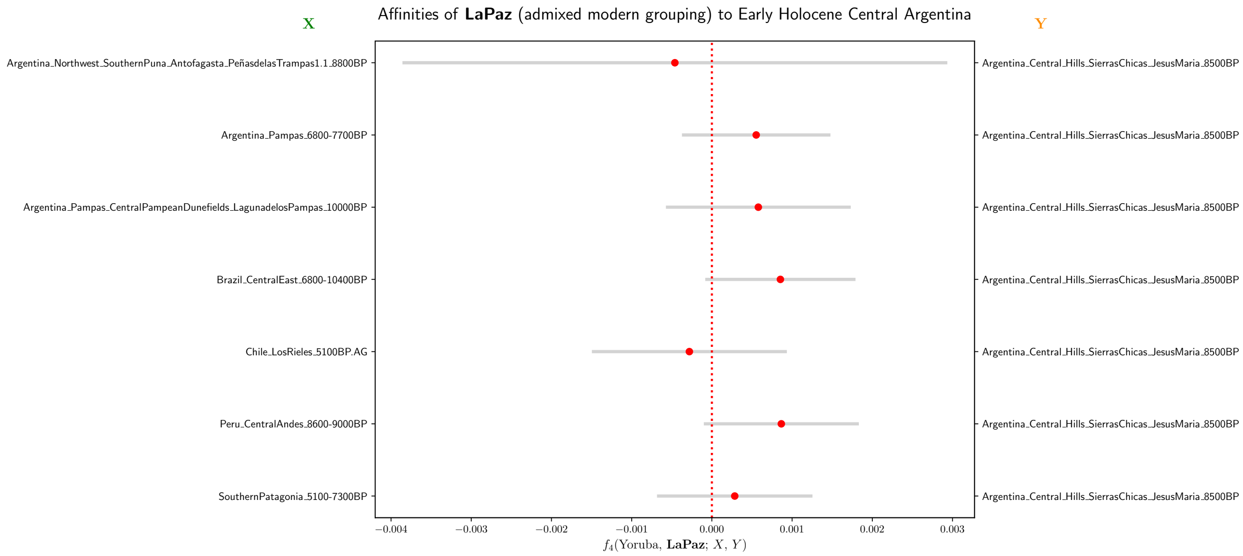

Supplementary Figure 46: Affinities of a modern Central Argentina admixed grouping from [1] to Early/Middle Holocene South American samples. Red dots denote tests with fewer than 75000 SNPs available. Bars denote 95% confidence intervals (1.96× standard errors).

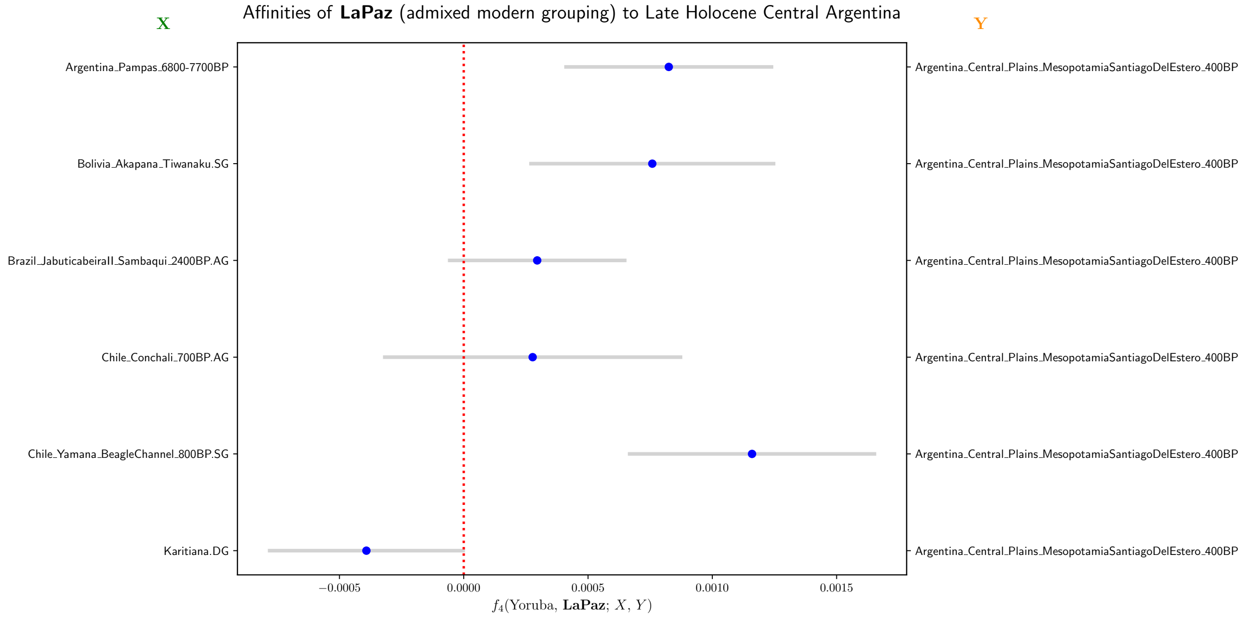

Supplementary Figure 47: Affinities of a modern Central Argentina admixed grouping from [1] to Late Holocene South American samples. Bars denote 95% confidence intervals (1.96× standard errors).

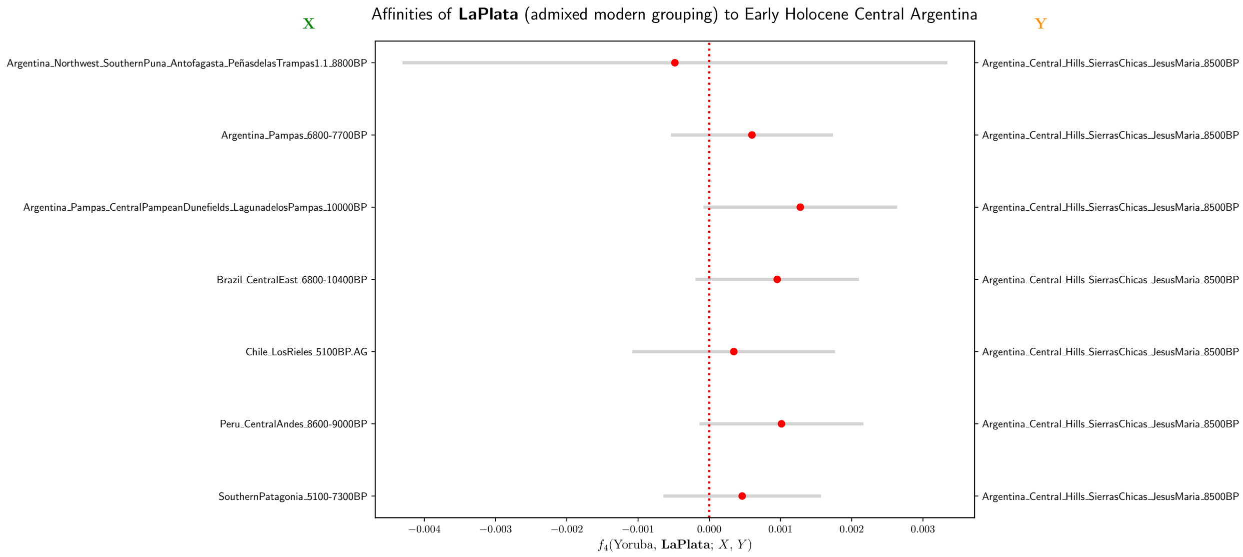

Supplementary Figure 48: Affinities of a modern Central Argentina admixed grouping from [1] to Early/Middle Holocene South American samples. Red dots denote tests with fewer than 75000 SNPs available. Bars denote 95% confidence intervals (1.96× standard errors).

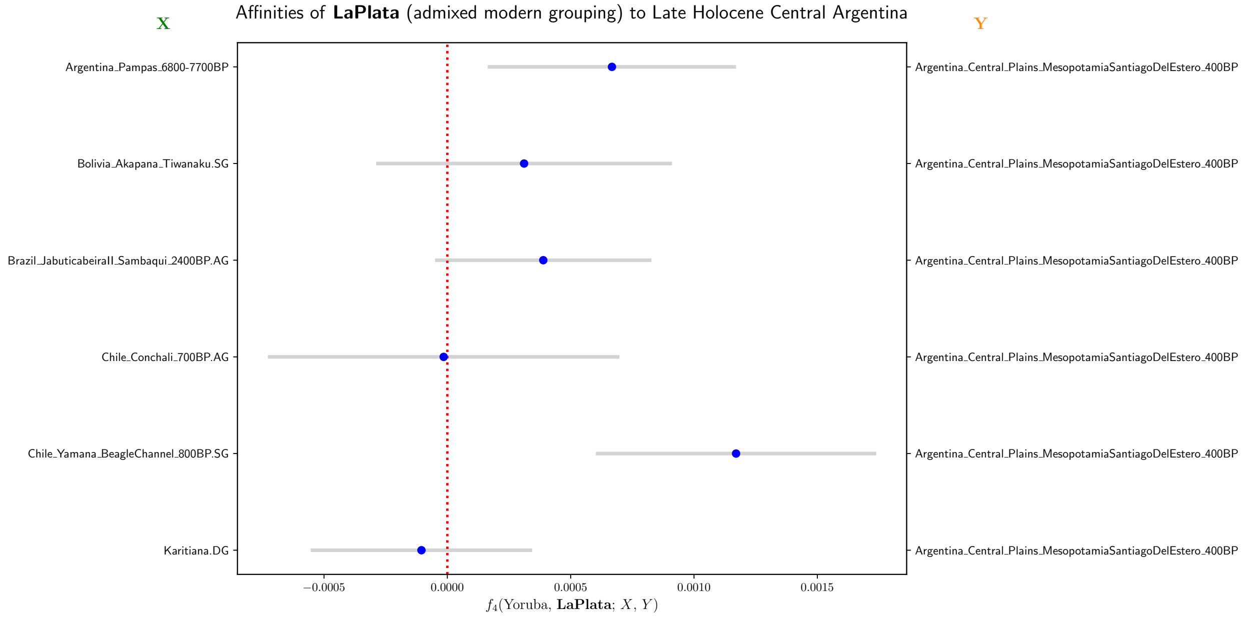

Supplementary Figure 49: Affinities of a modern Central Argentina admixed grouping from [1] to Late Holocene South American samples. Bars denote 95% confidence intervals (1.96× standard errors).

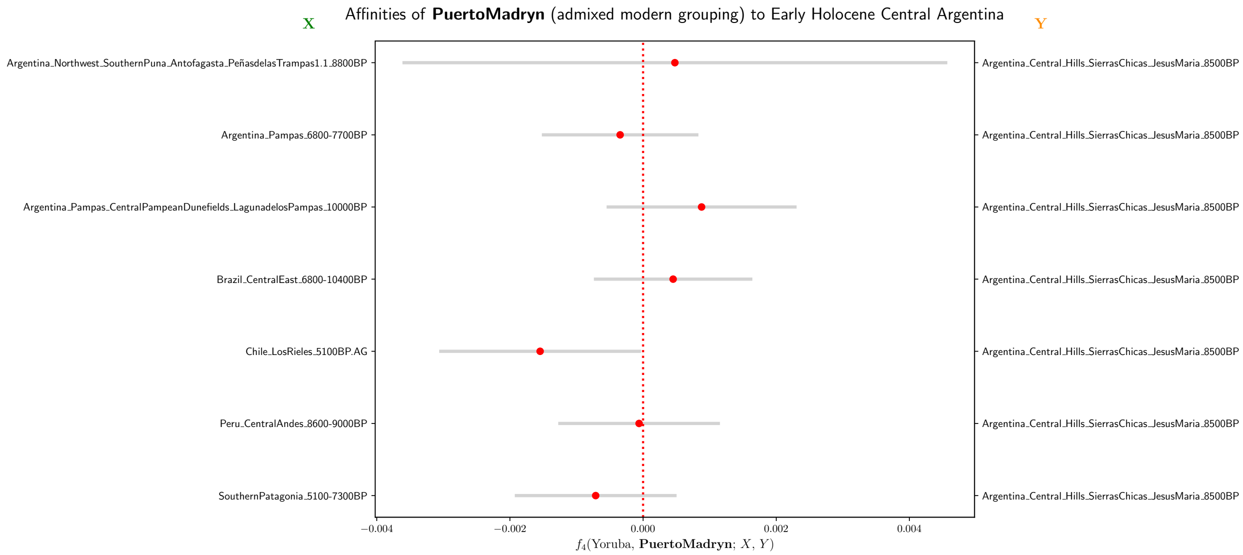

Supplementary Figure 50: Affinities of a modern Central Argentina admixed grouping from [1] to Early/Middle Holocene South American samples. Red dots denote tests with fewer than 75000 SNPs available. Bars denote 95% confidence intervals (1.96× standard errors).

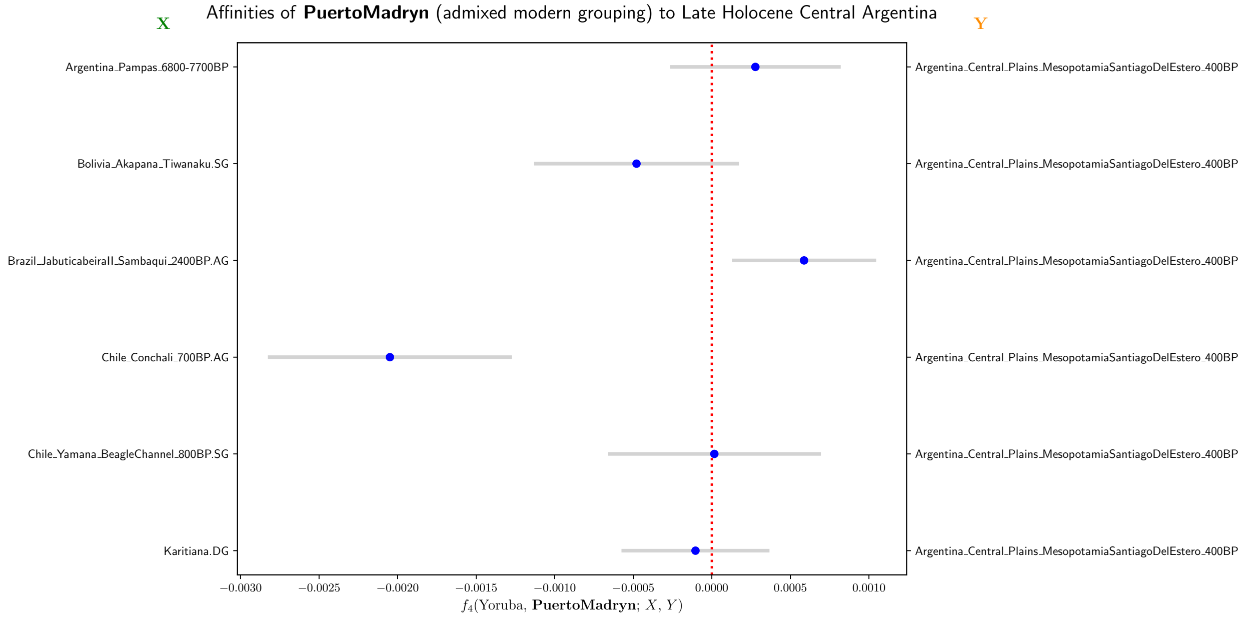

Supplementary Figure 51: Affinities of a modern Central Argentina admixed grouping from [1] to Late Holocene South American samples. Bars denote 95% confidence intervals (1.96× standard errors).

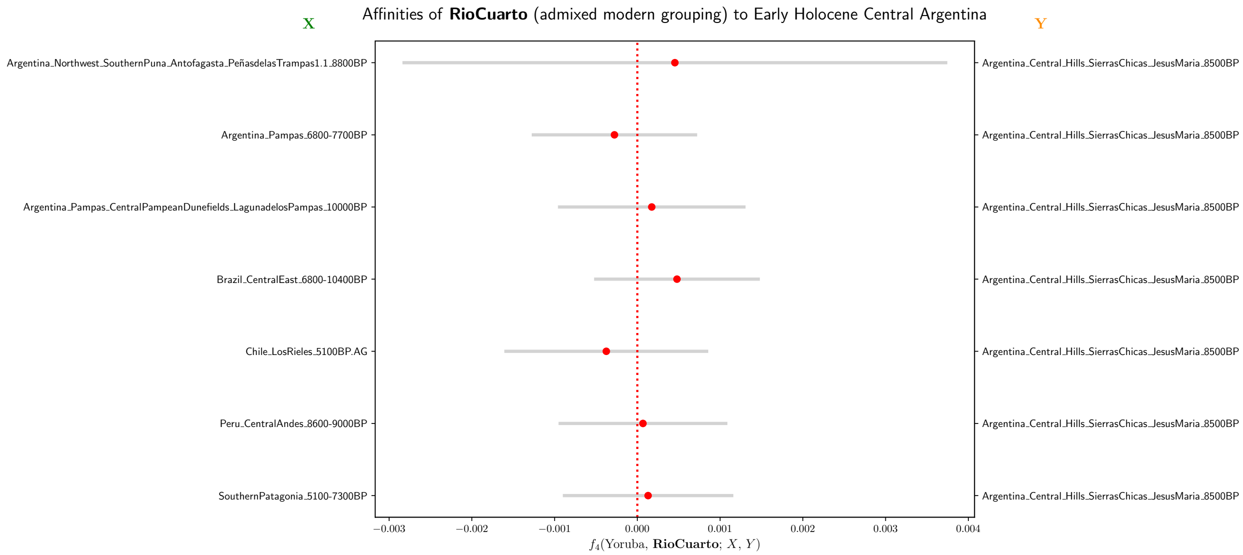

Supplementary Figure 52: Affinities of a modern Central Argentina admixed grouping from [1] to Early/Middle Holocene South American samples. Red dots denote tests with fewer than 75000 SNPs available. Bars denote 95% confidence intervals (1.96× standard errors).

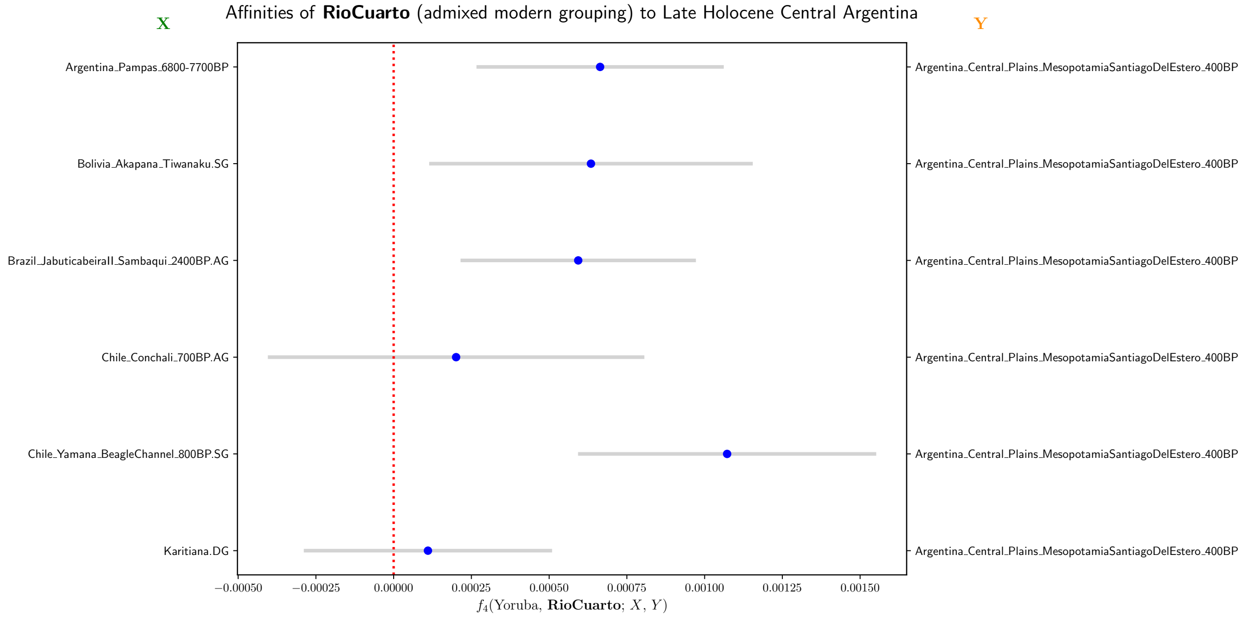

Supplementary Figure 53: Affinities of a modern Central Argentina admixed grouping from [1] to Late Holocene South American samples. Bars denote 95% confidence intervals (1.96× standard errors).

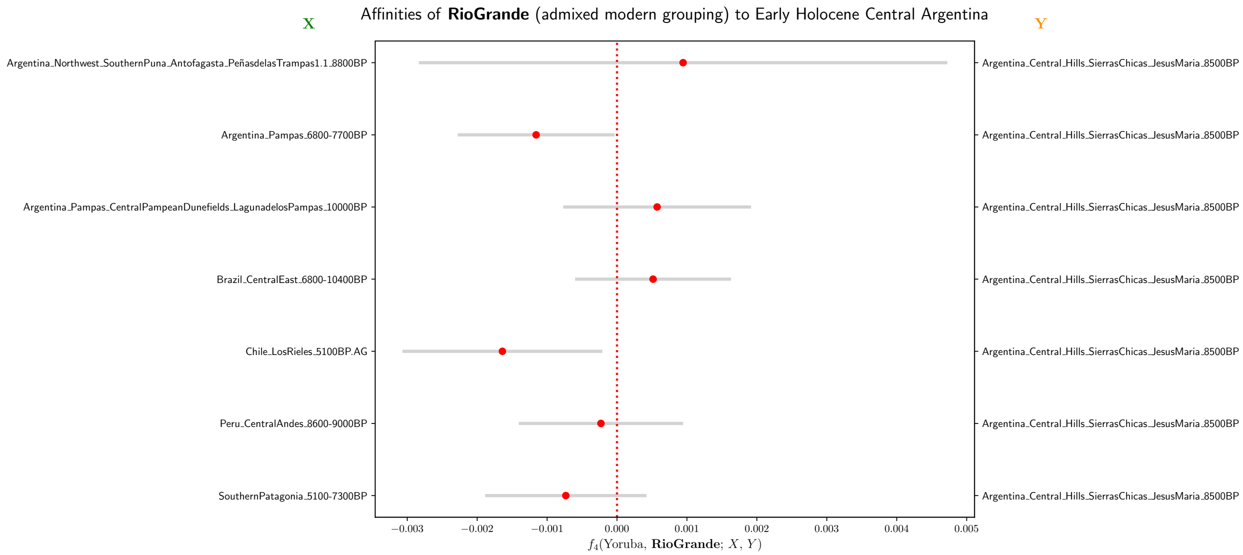

Supplementary Figure 54: Affinities of a modern Central Argentina admixed grouping from [1] to Early/Middle Holocene South American samples. Red dots denote tests with fewer than 75000 SNPs available. Bars denote 95% confidence intervals (1.96× standard errors).

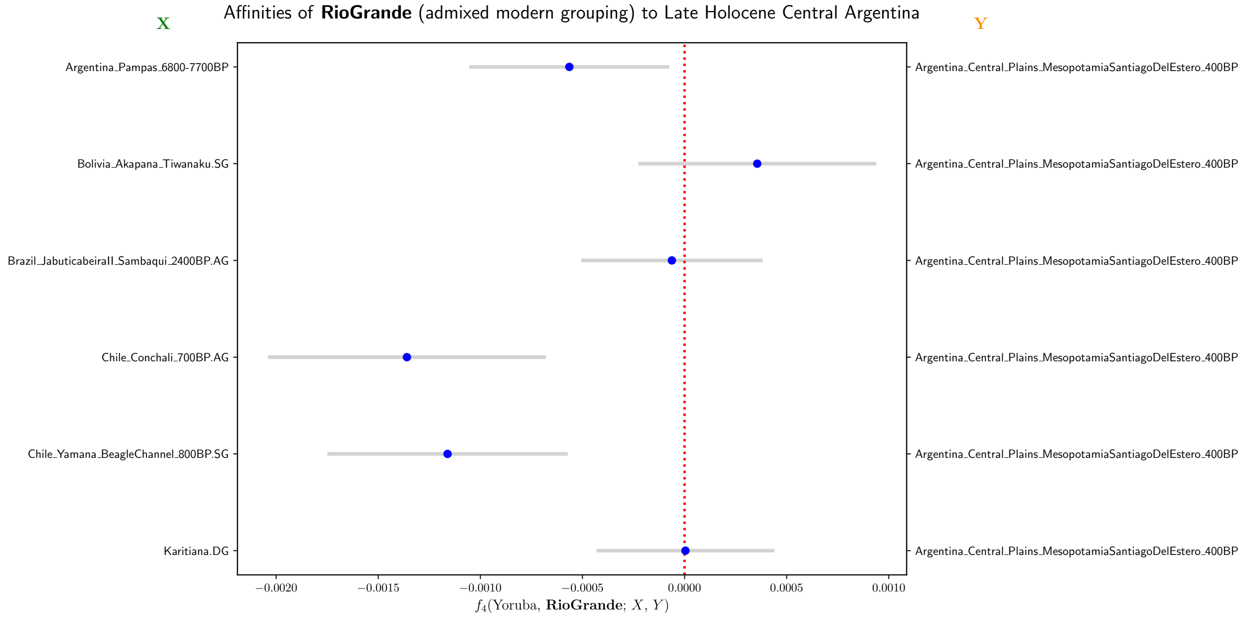

Supplementary Figure 55: Affinities of a modern Central Argentina admixed grouping from [1] to Late Holocene South American samples. Bars denote 95% confidence intervals (1.96× standard errors).

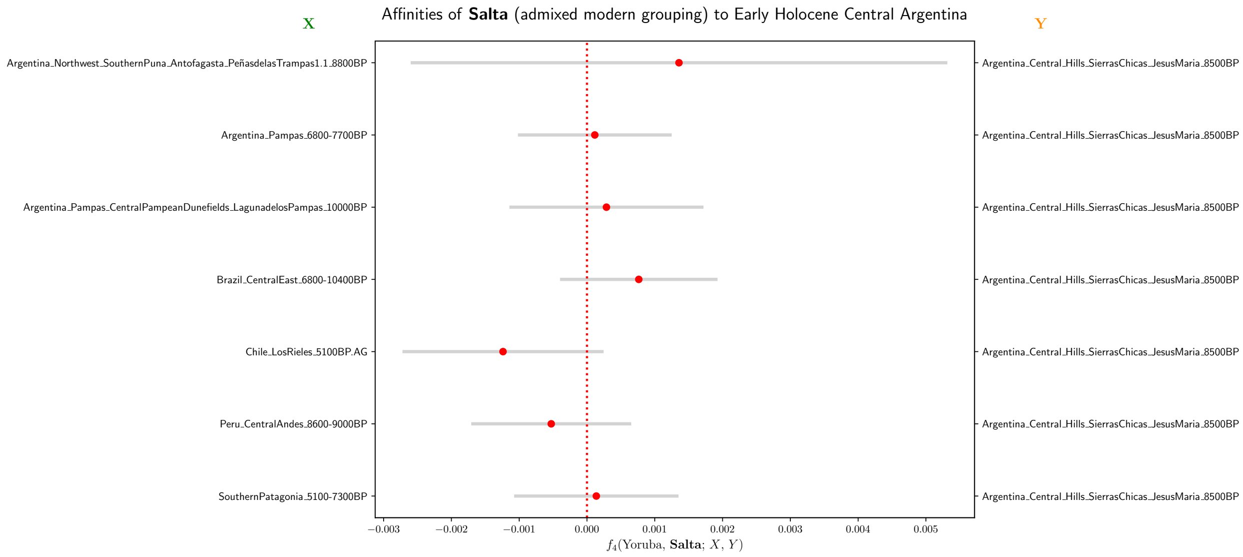

Supplementary Figure 56: Affinities of a modern Central Argentina admixed grouping from [1] to Early/Middle Holocene South American samples. Red dots denote tests with fewer than 75000 SNPs available. Bars denote 95% confidence intervals (1.96× standard errors).

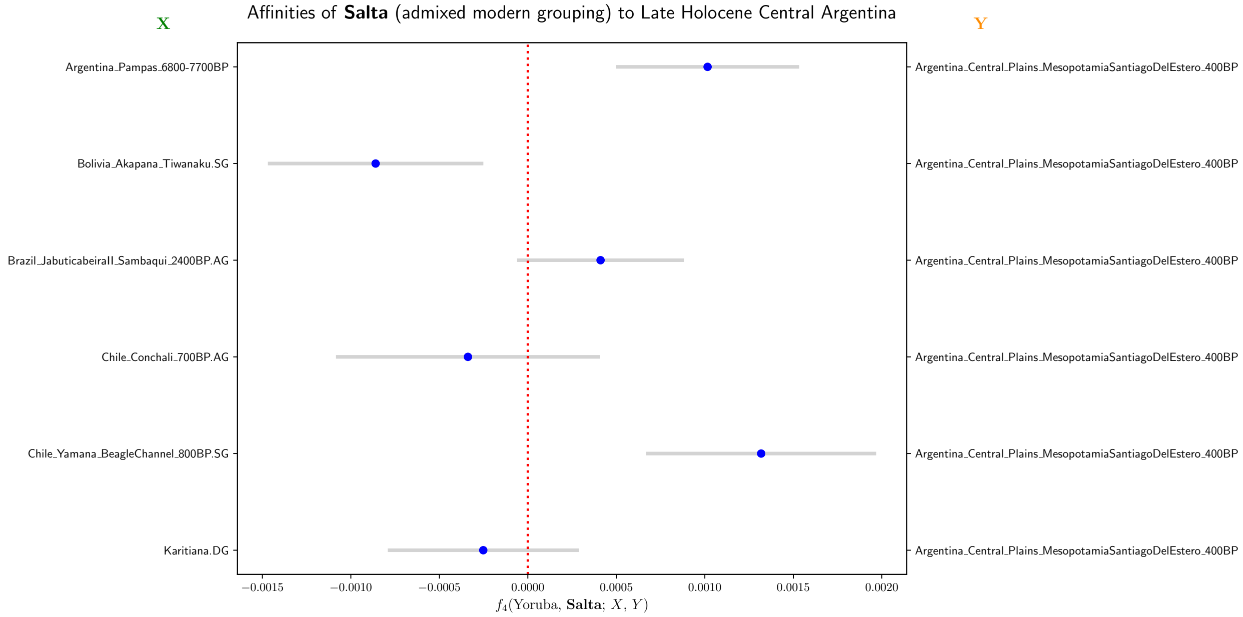

Supplementary Figure 57: Affinities of a modern Central Argentina admixed grouping from [1] to Late Holocene South American samples. Bars denote 95% confidence intervals (1.96× standard errors).

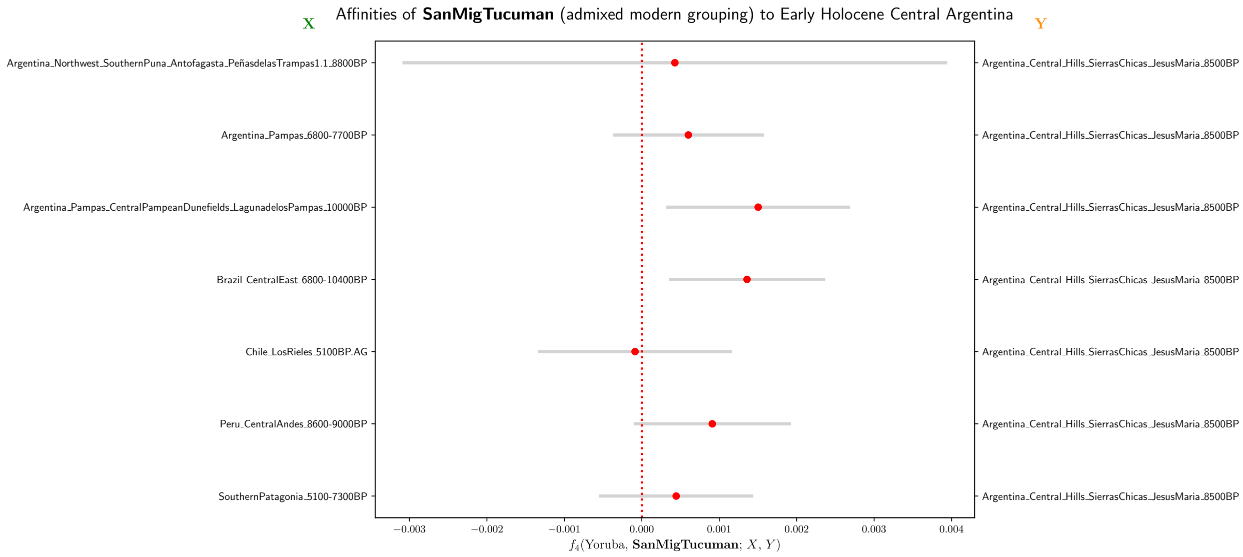

Supplementary Figure 58: Affinities of a modern Central Argentina admixed grouping from [1] to Early/Middle Holocene South American samples. Red dots denote tests with fewer than 75000 SNPs available. Bars denote 95% confidence intervals (1.96× standard errors).

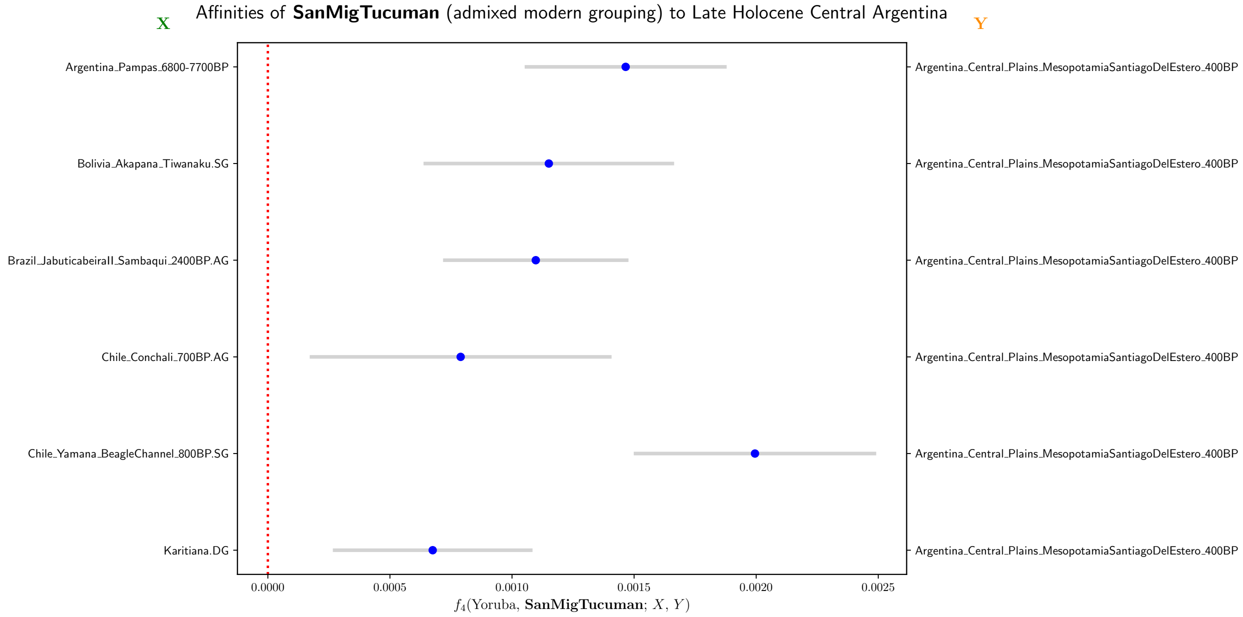

Supplementary Figure 59: Affinities of a modern Central Argentina admixed grouping from [1] to Late Holocene South American samples. Bars denote 95% confidence intervals (1.96× standard errors).

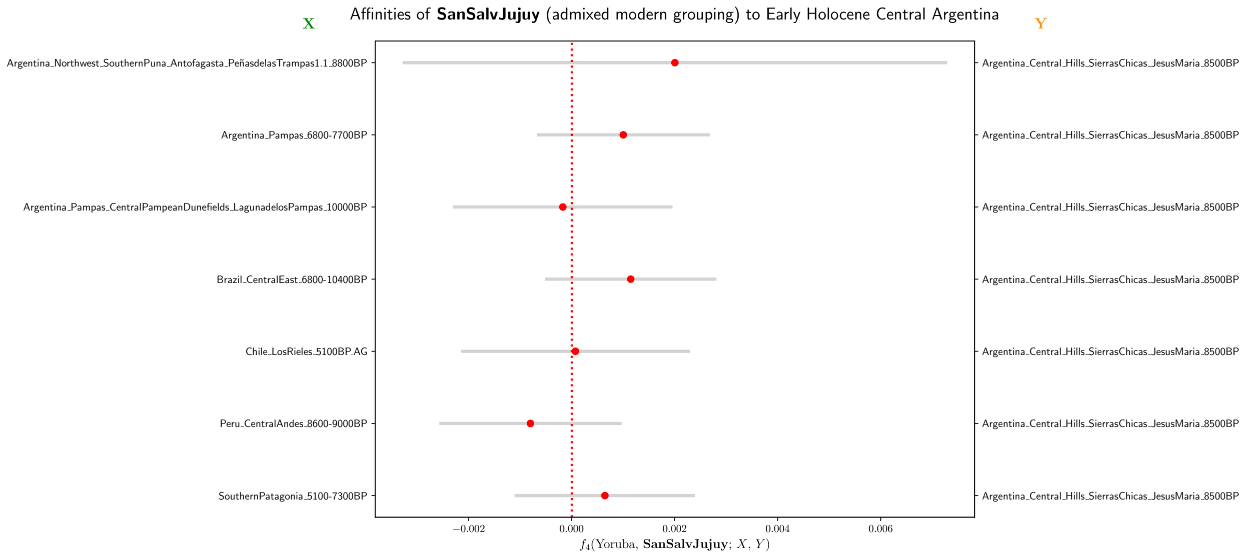

Supplementary Figure 60: Affinities of a modern Central Argentina admixed grouping from [1] to Early/Middle Holocene South American samples. Red dots denote tests with fewer than 75000 SNPs available. Bars denote 95% confidence intervals (1.96× standard errors).

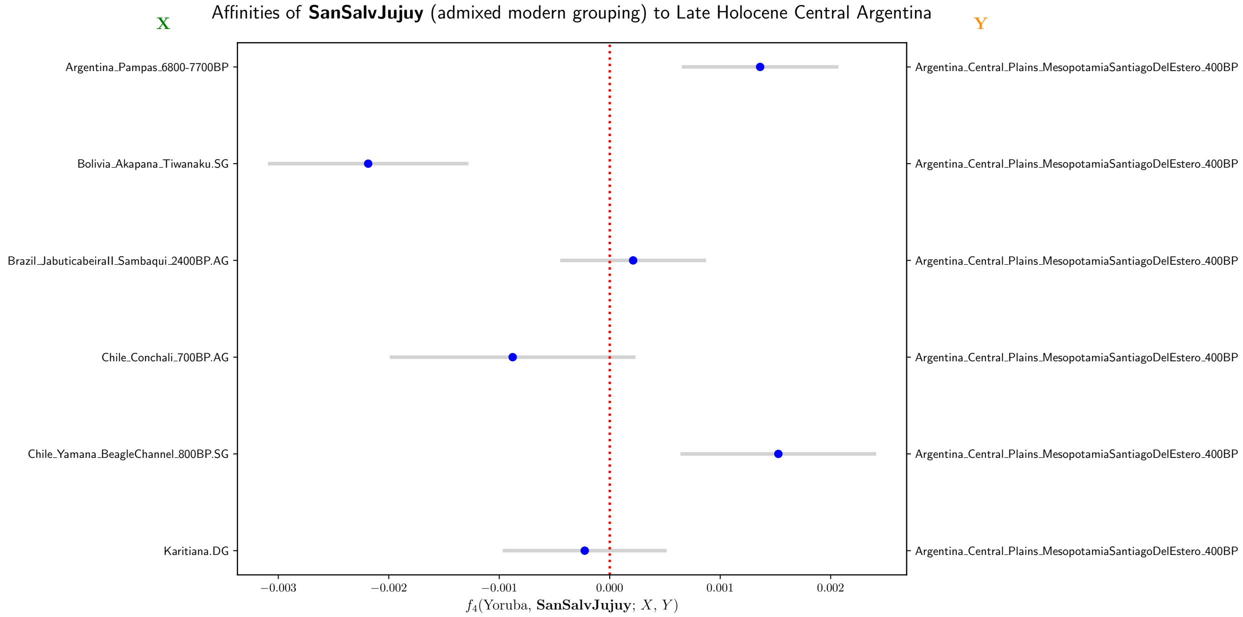

Supplementary Figure 61: Affinities of a modern Central Argentina admixed grouping from [1] to Late Holocene South American samples. Bars denote 95% confidence intervals (1.96× standard errors).

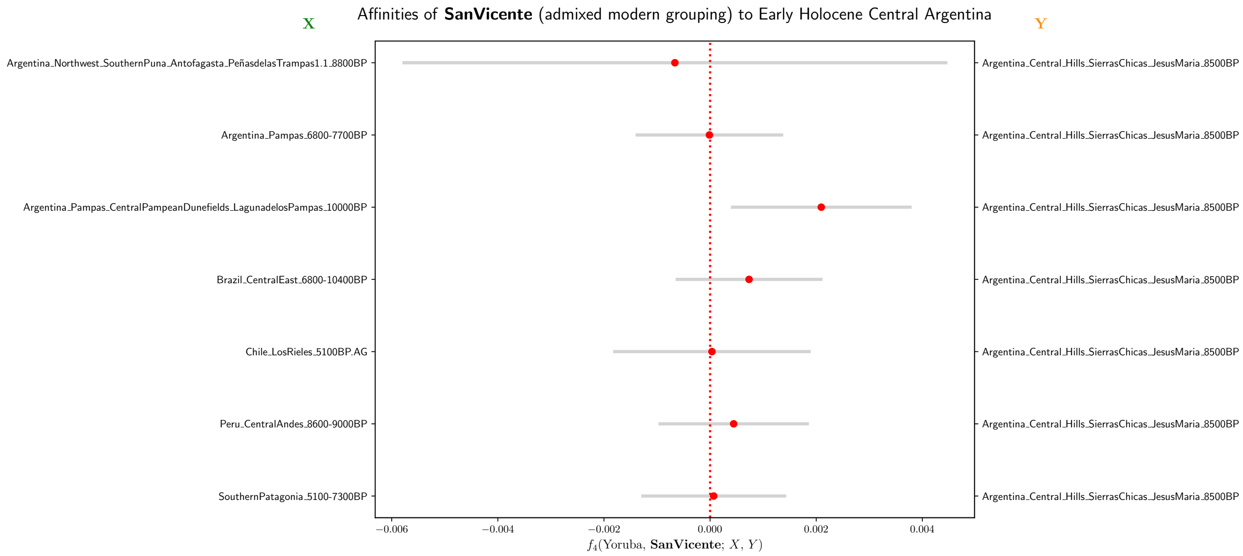

Supplementary Figure 62: Affinities of a modern Central Argentina admixed grouping from [1] to Early/Middle Holocene South American samples. Red dots denote tests with fewer than 75000 SNPs available. Bars denote 95% confidence intervals (1.96× standard errors).

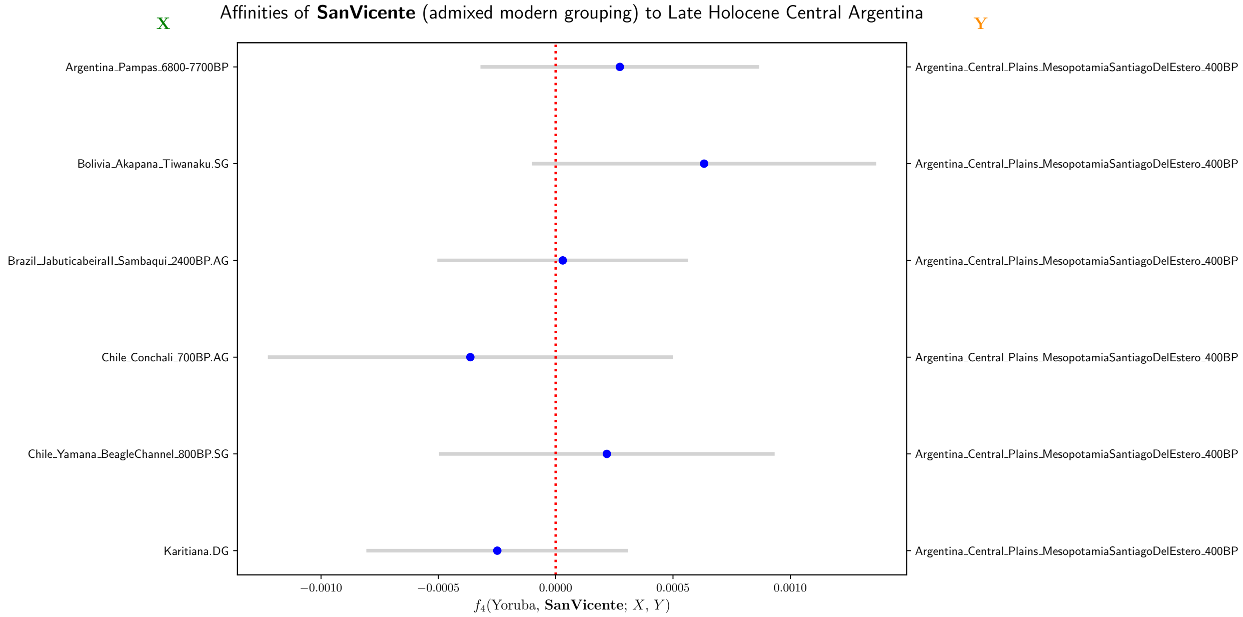

Supplementary Figure 63: Affinities of a modern Central Argentina admixed grouping from [1] to Late Holocene South American samples. Bars denote 95% confidence intervals (1.96× standard errors).

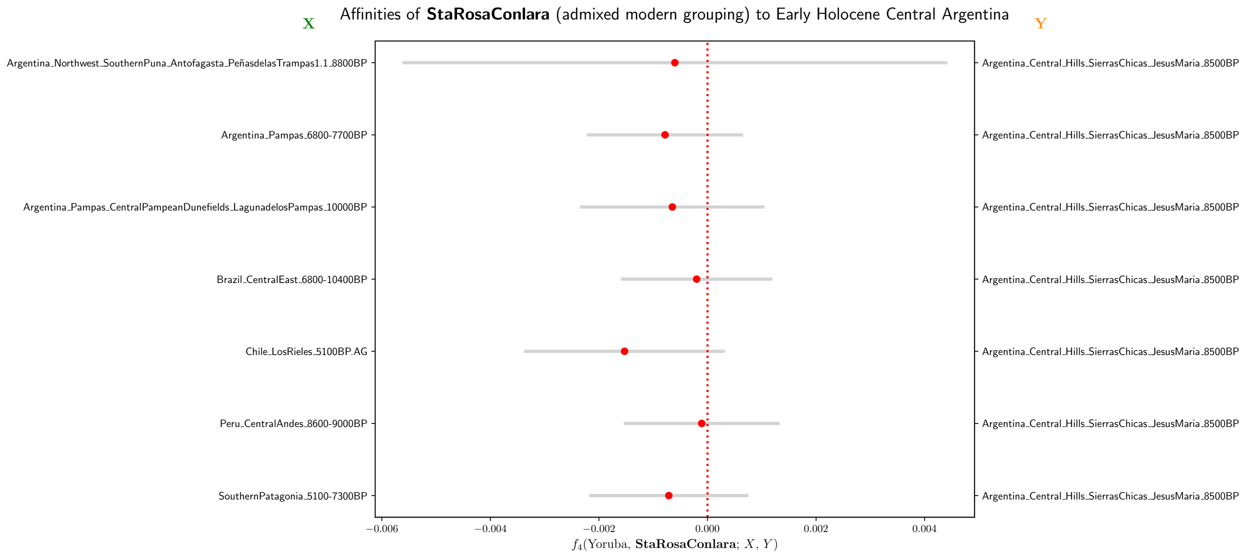

Supplementary Figure 64: Affinities of a modern Central Argentina admixed grouping from [1] to Early/Middle Holocene South American samples. Red dots denote tests with fewer than 75000 SNPs available. Bars denote 95% confidence intervals (1.96× standard errors).

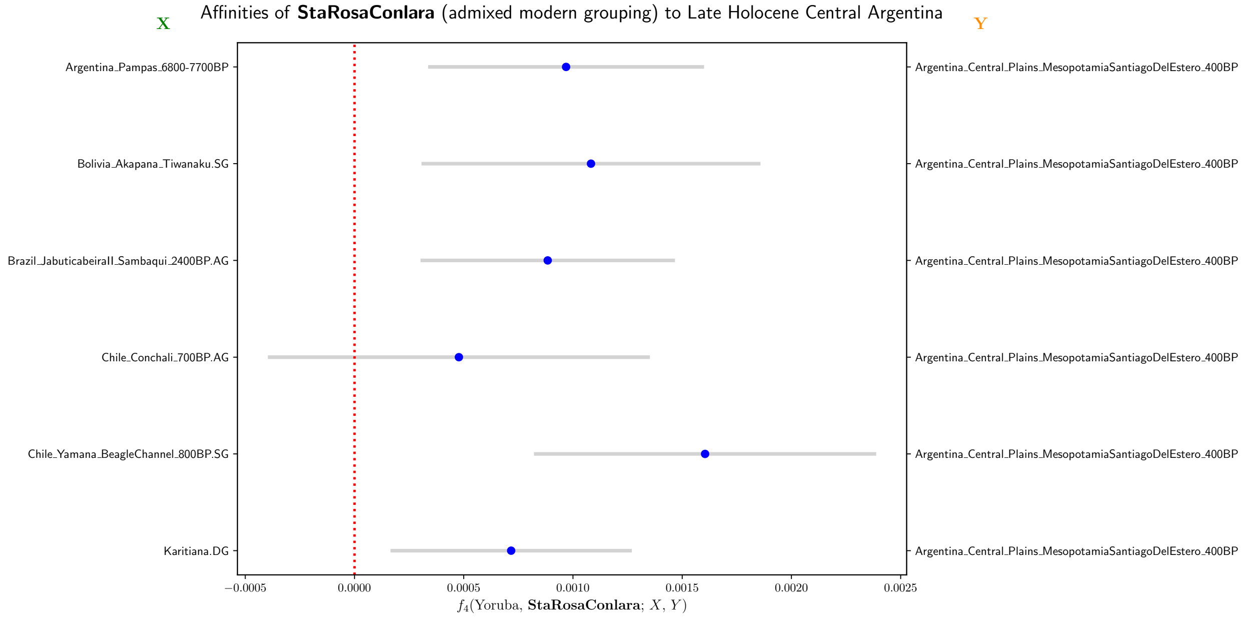

Supplementary Figure 65: Affinities of a modern Central Argentina admixed grouping from [1] to Late Holocene South American samples. Bars denote 95% confidence intervals (1.96× standard errors).

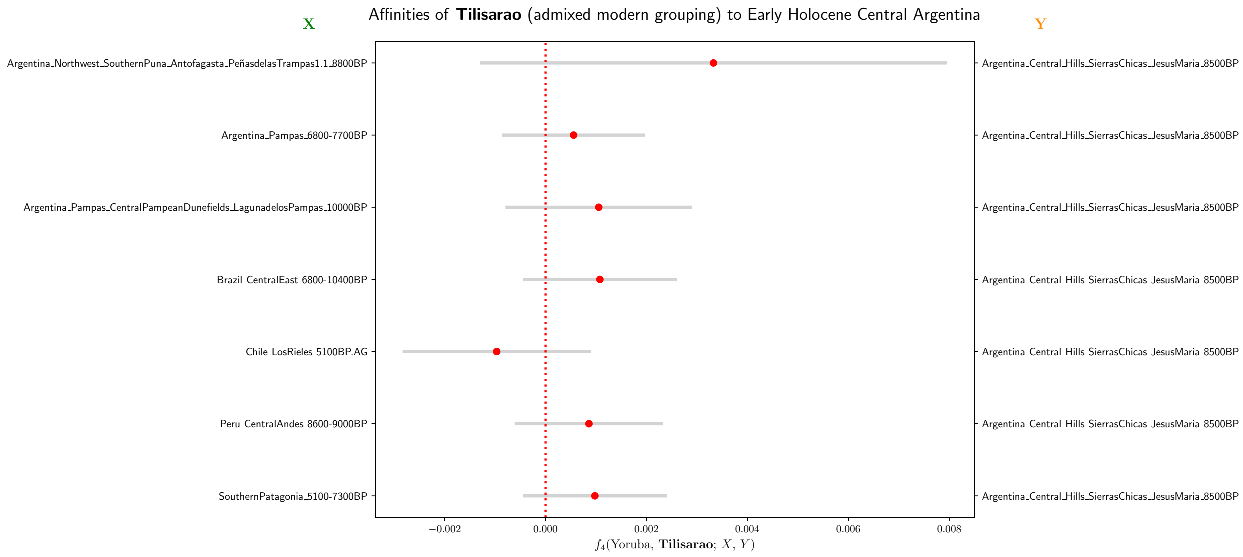

Supplementary Figure 66: Affinities of a modern Central Argentina admixed grouping from [1] to Early/Middle Holocene South American samples. Red dots denote tests with fewer than 75000 SNPs available. Bars denote 95% confidence intervals (1.96× standard errors).

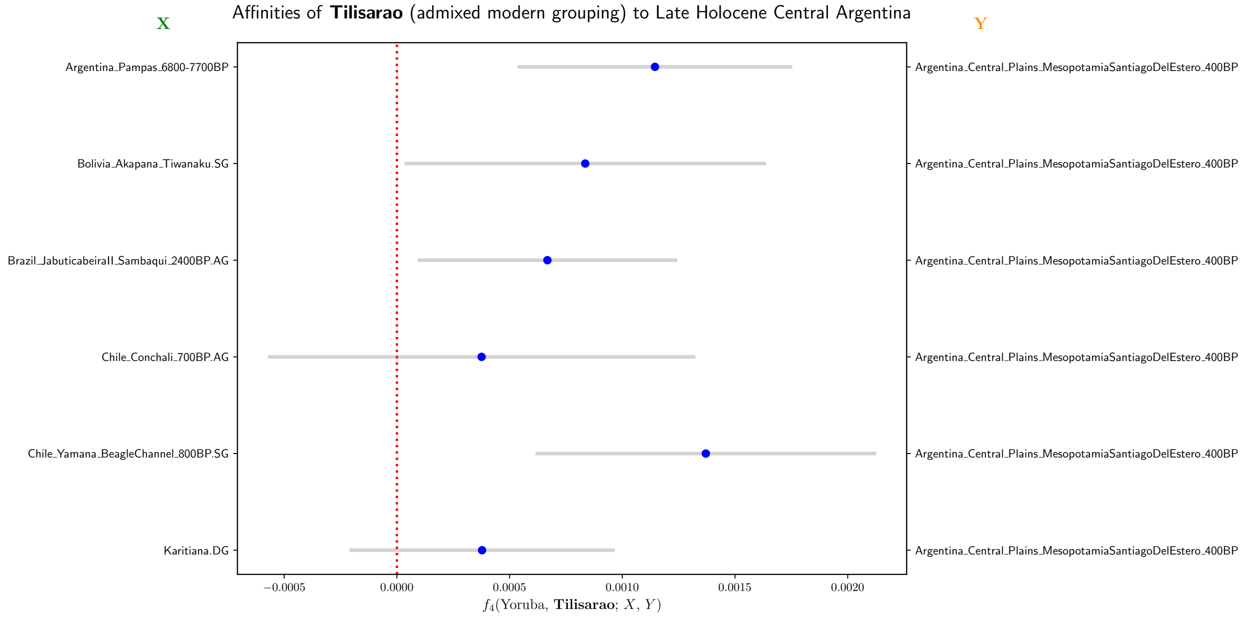

Supplementary Figure 67: Affinities of a modern Central Argentina admixed grouping from [1] to Late Holocene South American samples. Bars denote 95% confidence intervals (1.96× standard errors).

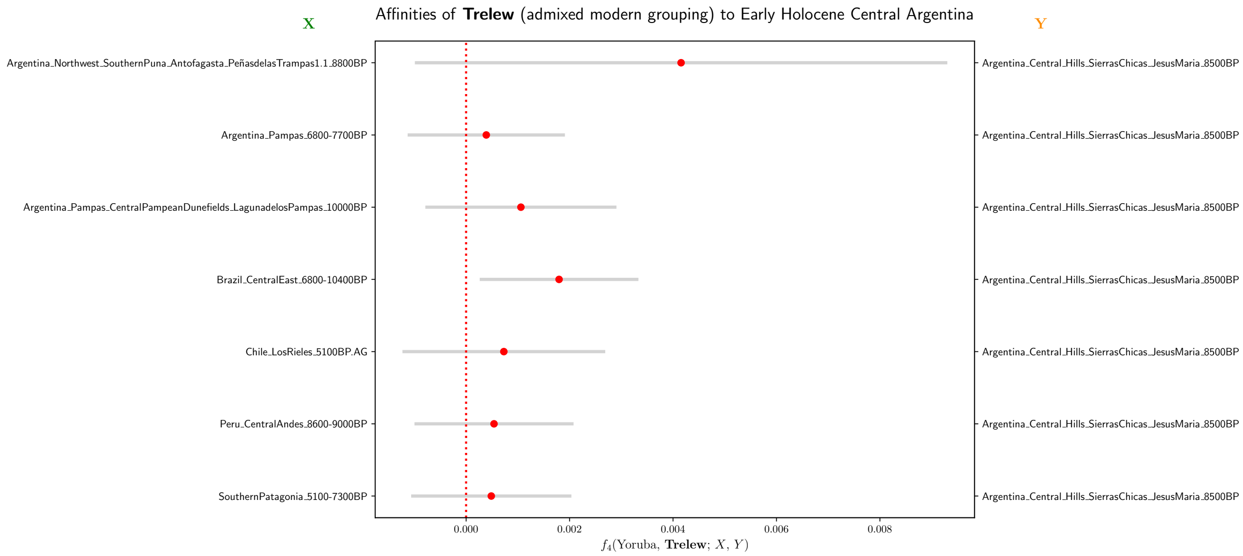

Supplementary Figure 68: Affinities of a modern Central Argentina admixed grouping from [1] to Early/Middle Holocene South American samples. Red dots denote tests with fewer than 75000 SNPs available. Bars denote 95% confidence intervals (1.96× standard errors).

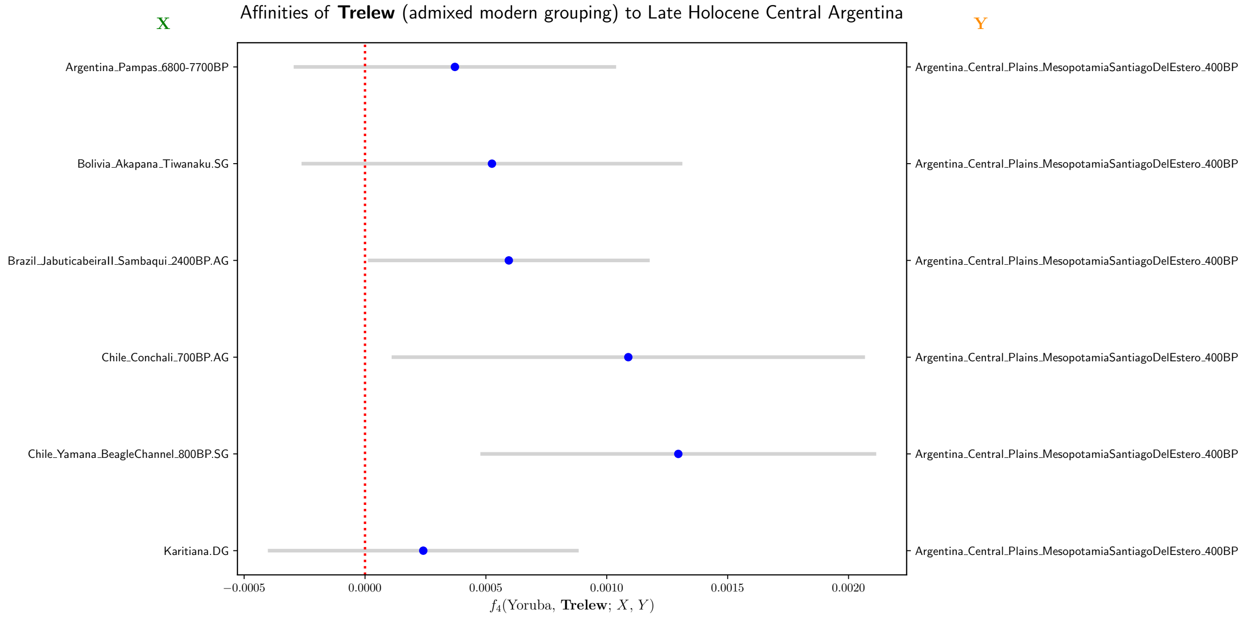

Supplementary Figure 69: Affinities of a modern Central Argentina admixed grouping from [1] to Late Holocene South American samples. Bars denote 95% confidence intervals (1.96× standard errors).

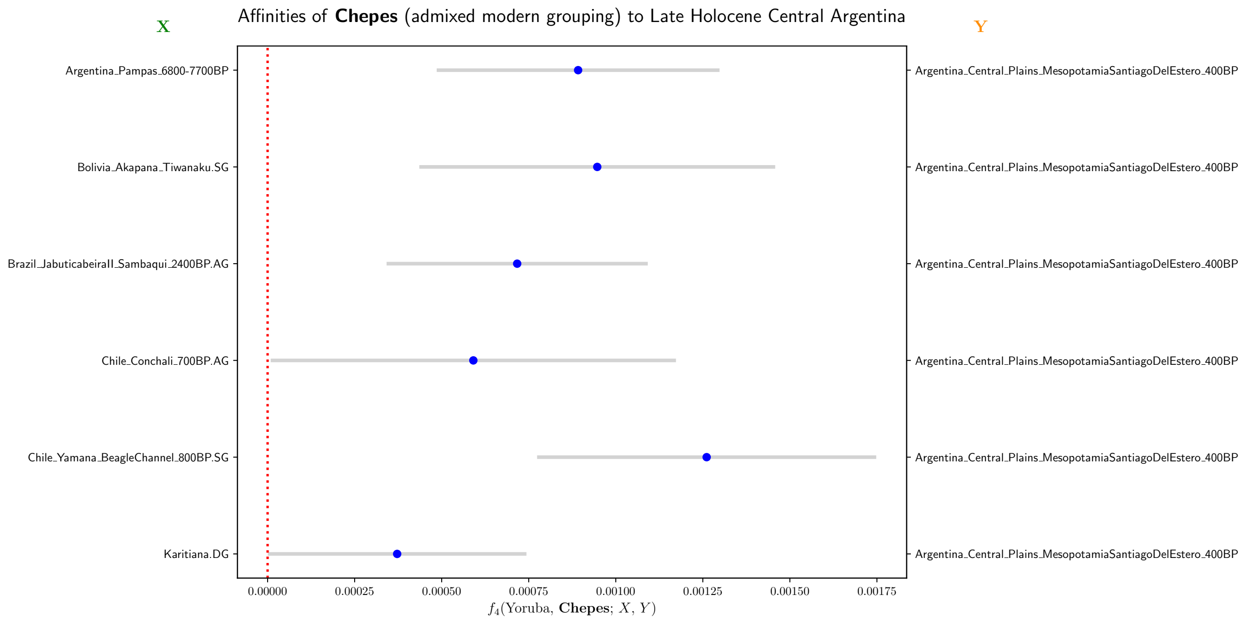

Supplementary Figure 70: Affinities of a representative modern Central Argentina admixed grouping from [1] to Late Holocene South American samples. Bars denote 95% confidence intervals (1.96× standard errors).

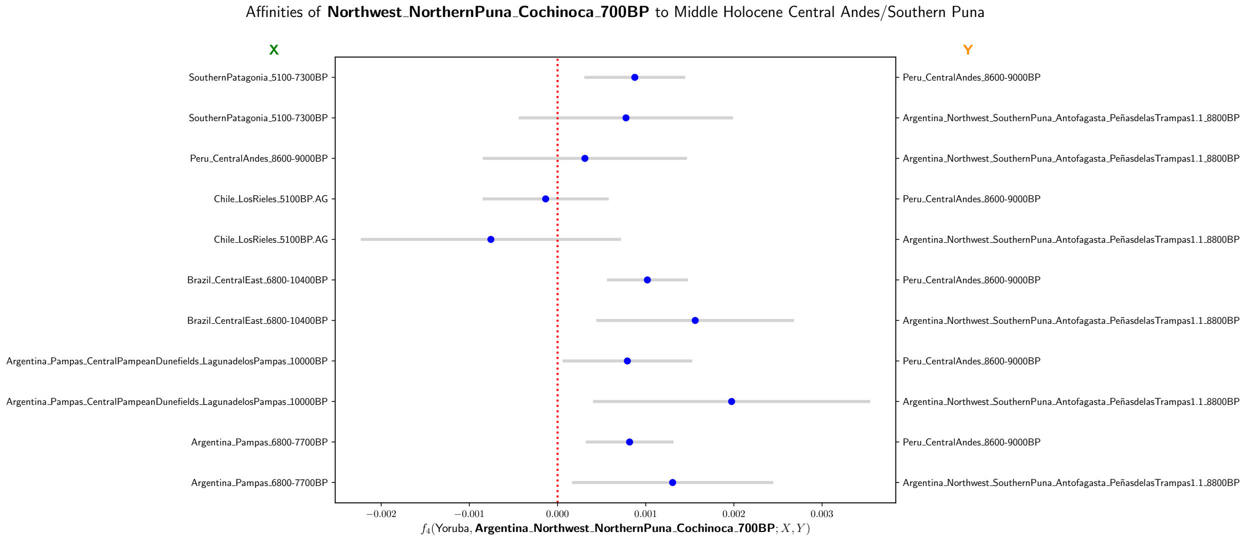

Supplementary Figure 71: Affinities of a *Northwest_Puna_Cochinoca_700BP* to Early/Middle Holocene South American samples. Bars denote 95% confidence intervals (1.96× standard errors).

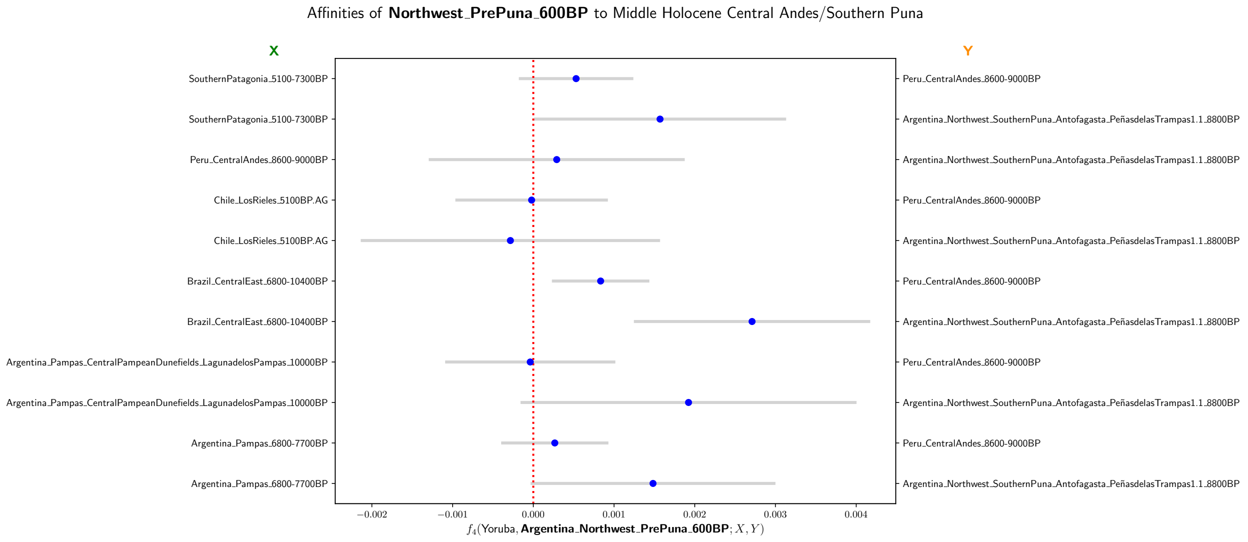

Supplementary Figure 72: Affinities of *Northwest_PrePuna_600BP* to Early/Middle Holocene South American samples. Bars denote 95% confidence intervals (1.96× standard errors).

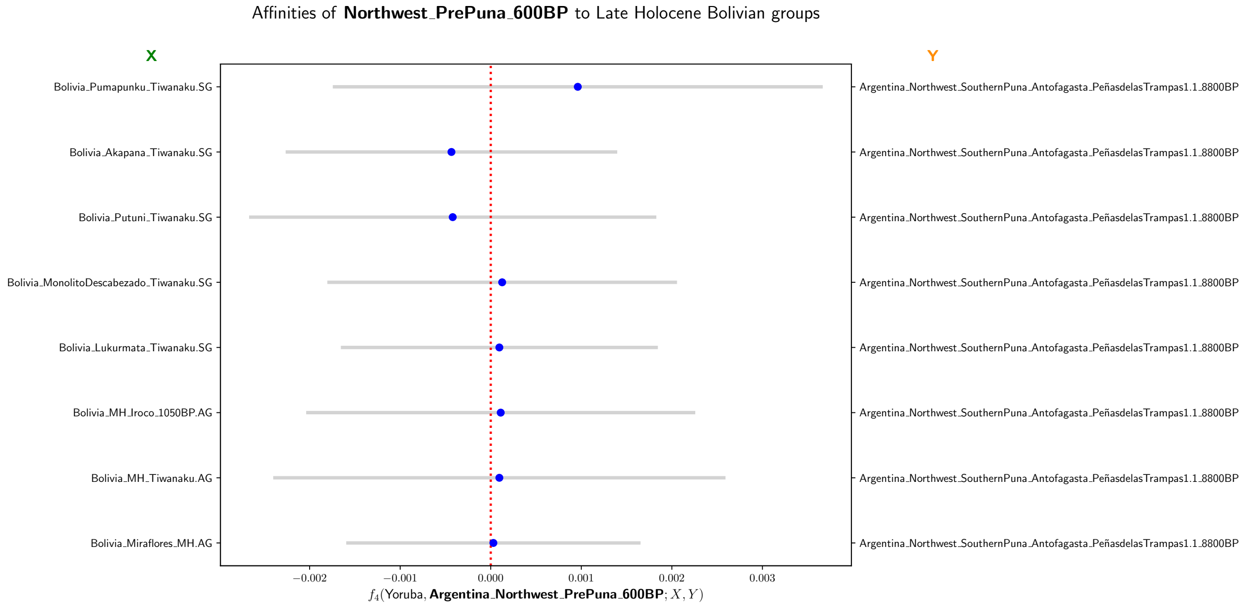

Supplementary Figure 73: Affinities of a *Northwest_PrePuna_600BP* to Late Holocene Bolivian groups. Bars denote 95% confidence intervals (1.96× standard errors).

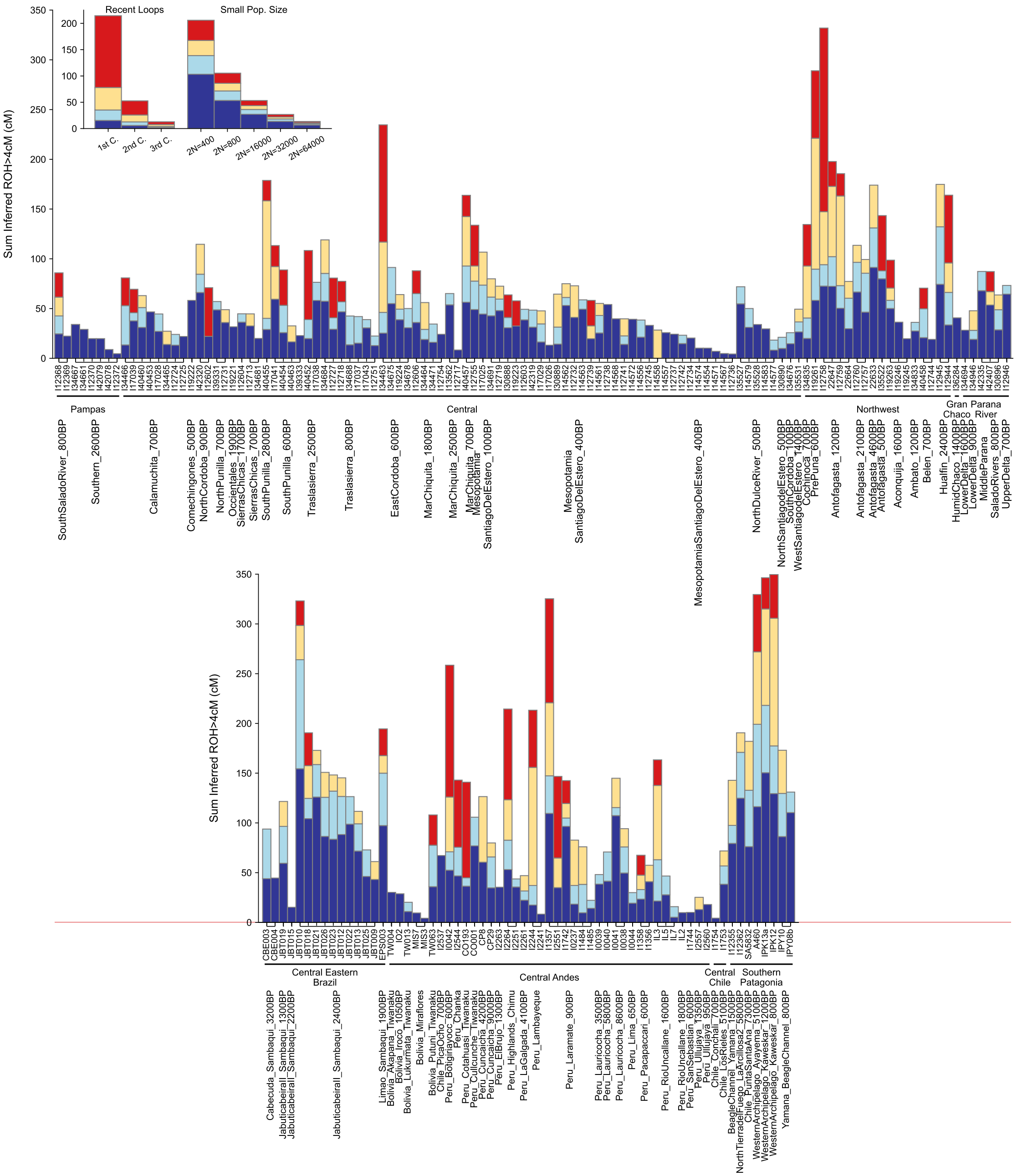

Supplementary Figure 74: `hapROH` estimates for sufficiently-covered (at least 400000 SNPs with respect to the 1240k set) newly-reported (top) and previously-published (bottom) comparison ancient individuals.

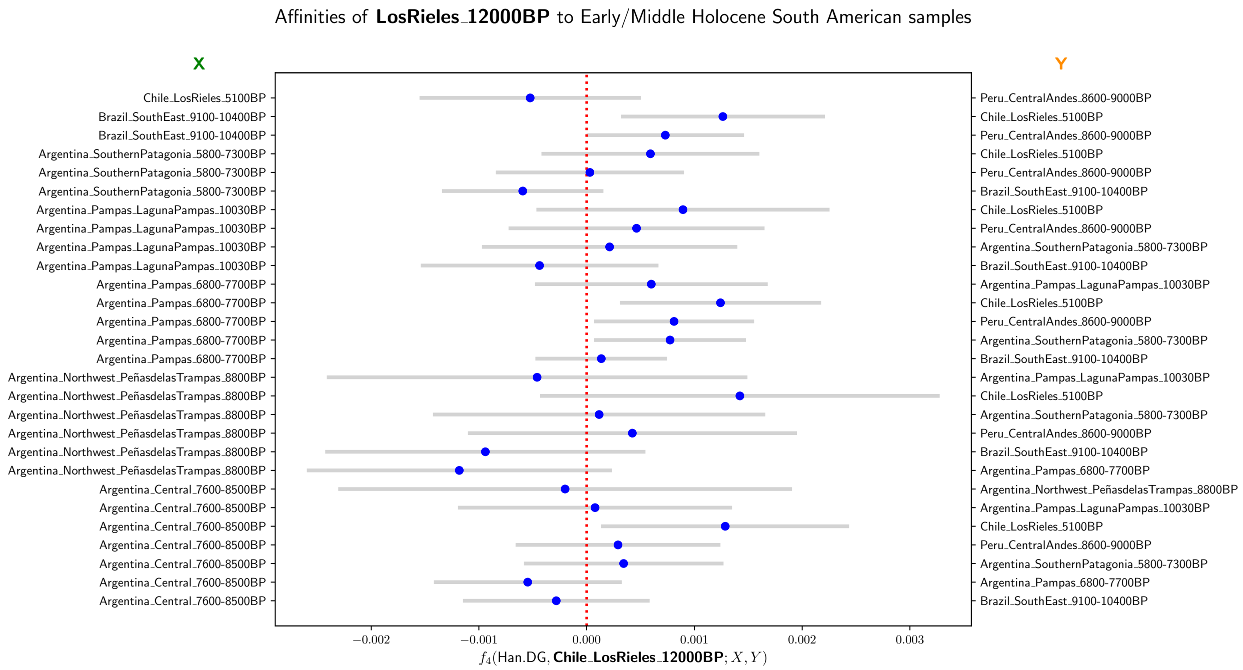

Supplementary Figure 75: Affinities of *LosRieles_12000BP* to Early/Middle Holocene South American samples. Bars denote 95% confidence intervals (1.96× SE).

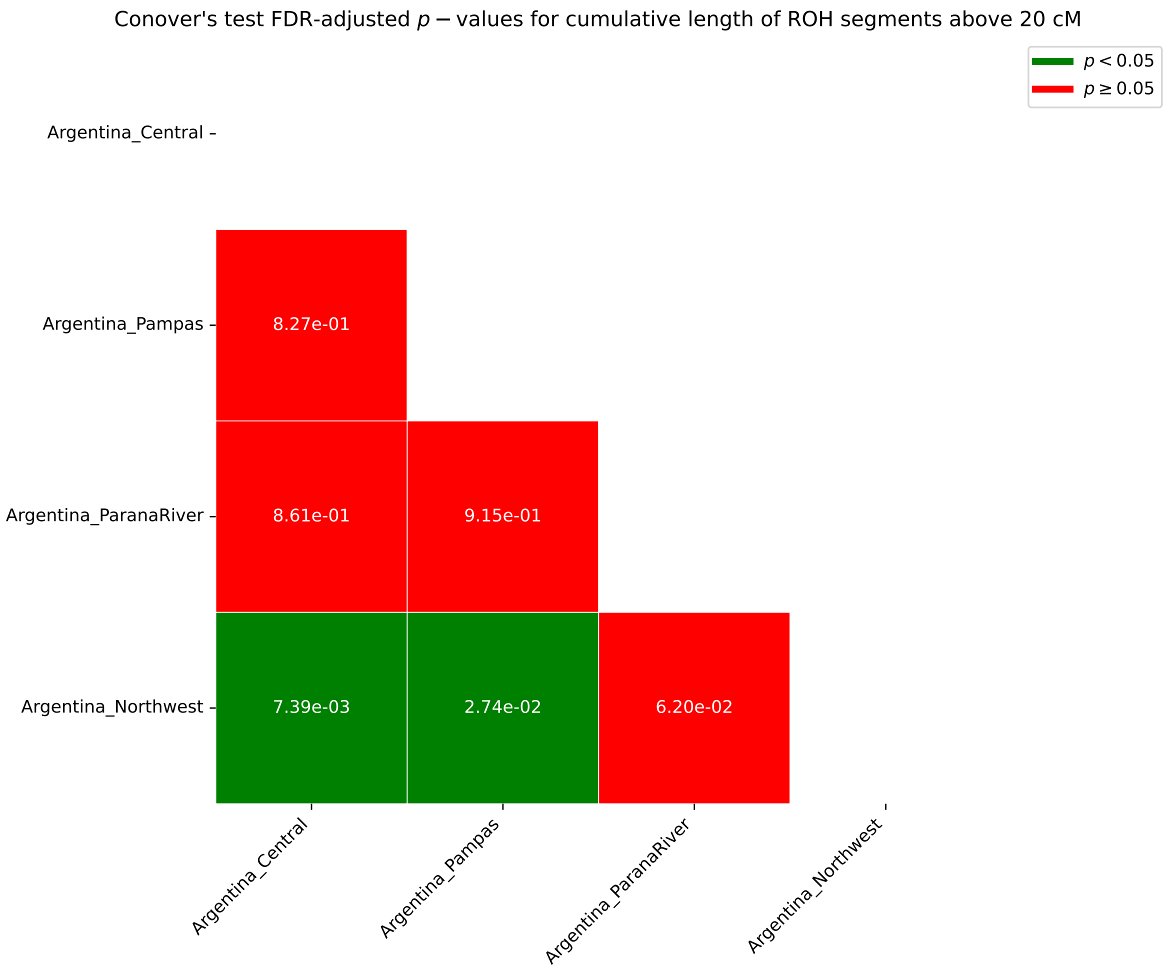

Supplementary Figure 76: FDR-adjusted $p-$values from a Conover test for significant pairwise differences in the distribution of cumulative length of ROH above 20 cM by region, up to 3000BP.

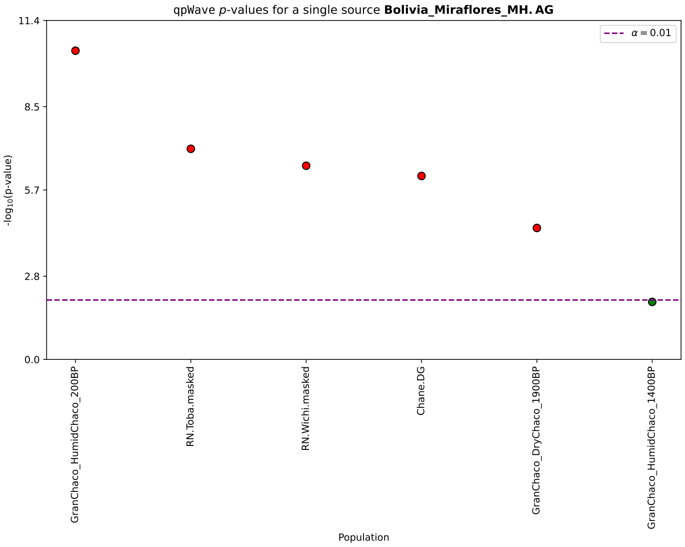

(a) Cladality with Central Andes

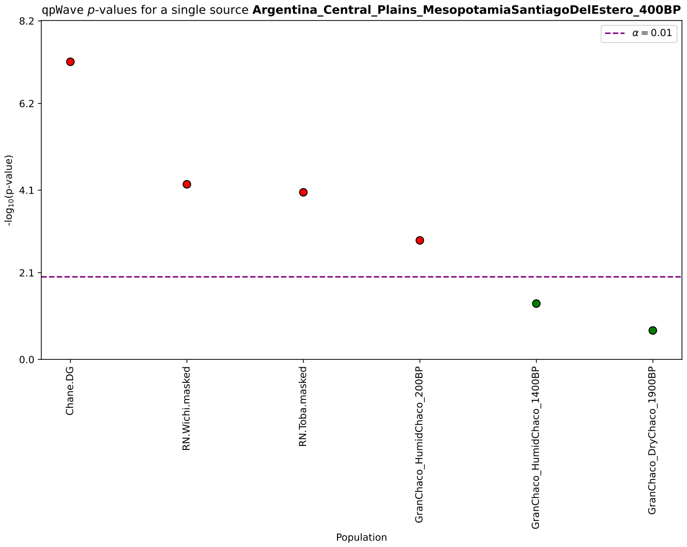

(b) Cladality with Central Argentina

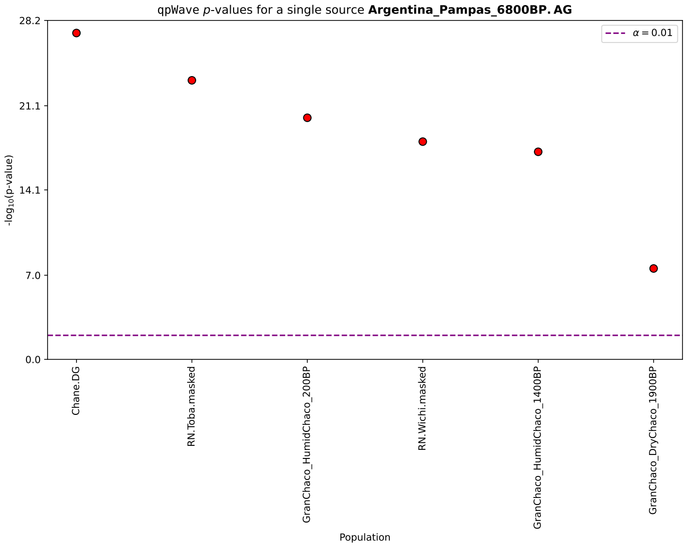

(c) Cladality with Middle Holocene Pampas

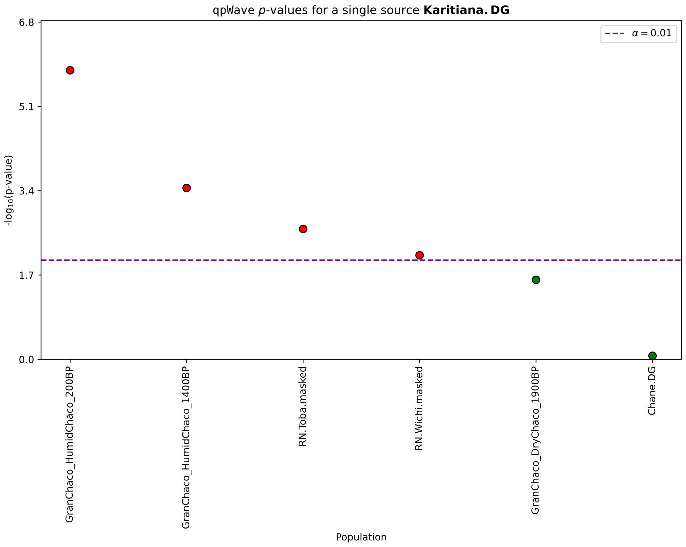

(d) Cladality with Karitiana (as a proxy for Tropical and Subtropical Forests ancestry)

Supplementary Figure 77: `qpWave` $p-$values for single-source models for Gran Chaco populations (failures at $\alpha = 0.01$ are shown in red)

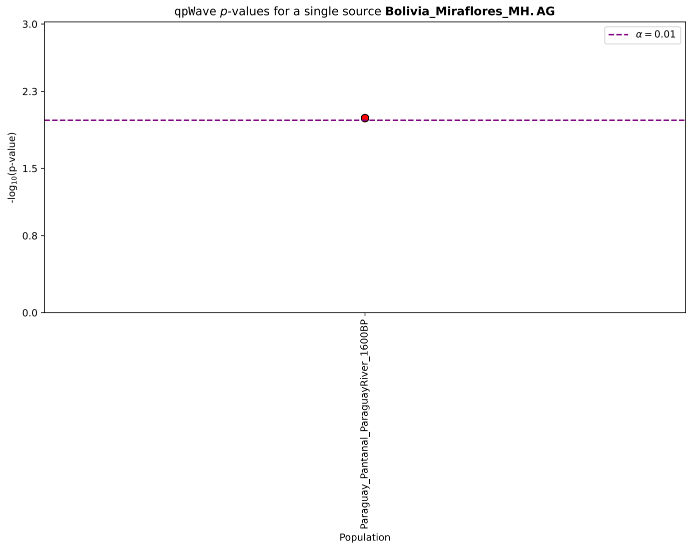

(a) Cladality with Central Andes

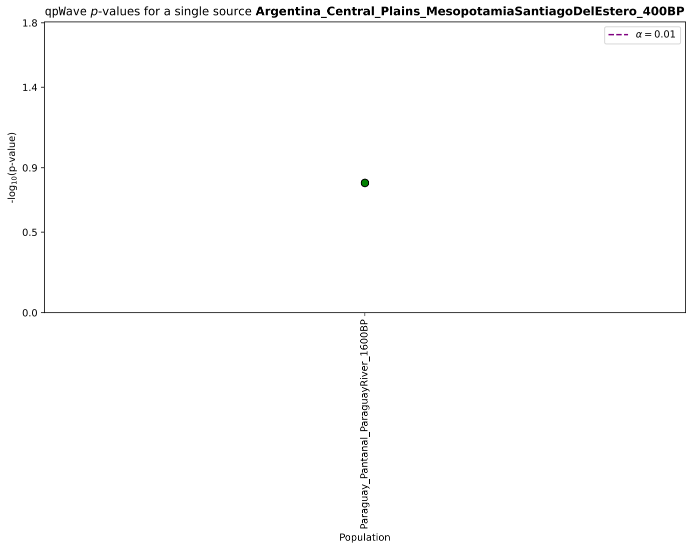

(b) Cladality with Central Argentina

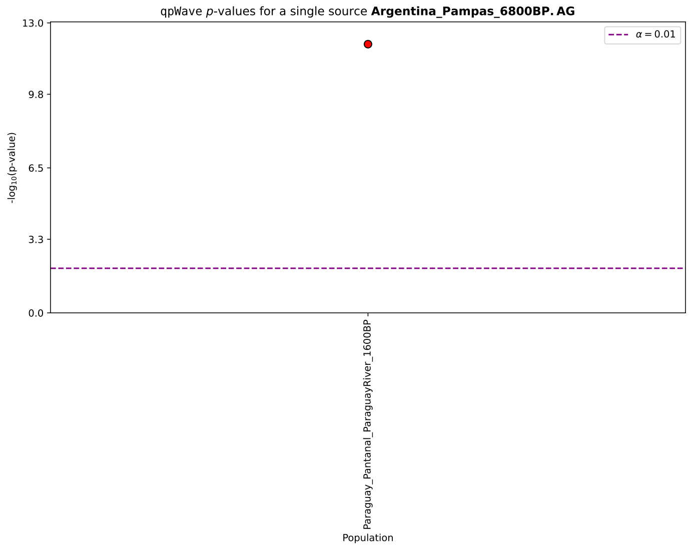

(c) Cladality with Middle Holocene Pampas

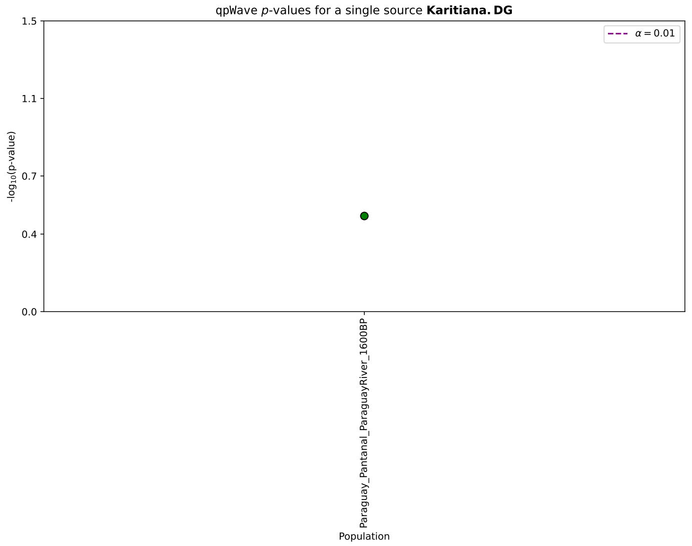

(d) Cladality with Karitiana (as a proxy for Tropical and Subtropical Forests ancestry)

Supplementary Figure 78: qpWave $p-$values for single-source models for Pantanal populations (failures at $\alpha = 0.01$ are shown in red)

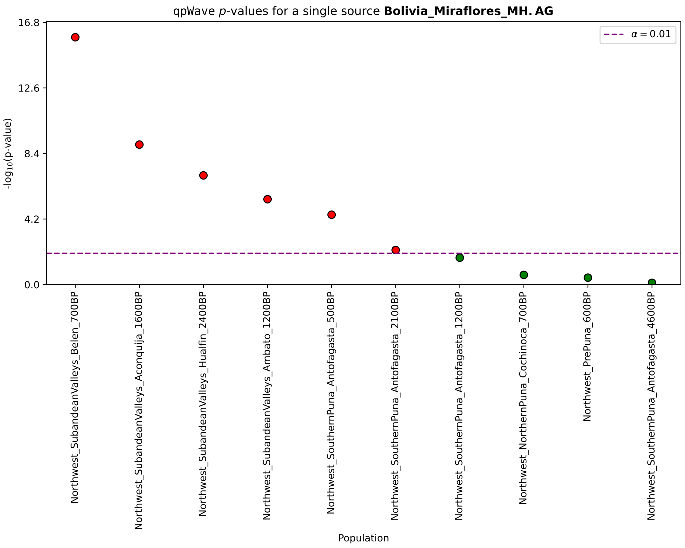

(a) Cladality with Central Andes

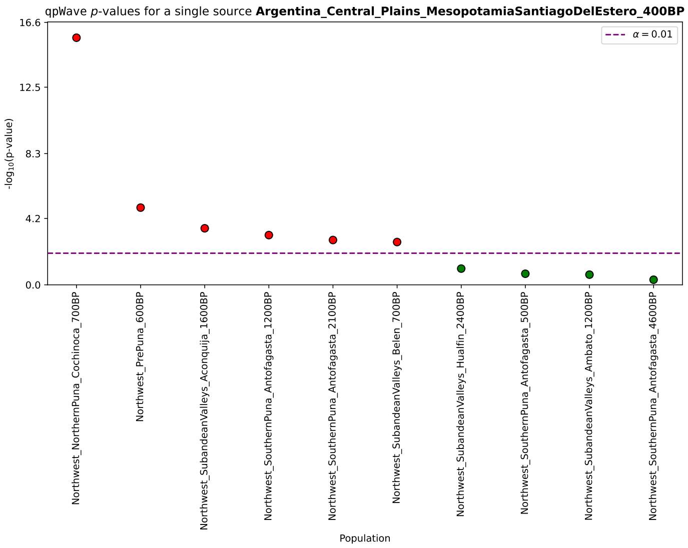

(b) Cladality with Central Argentina

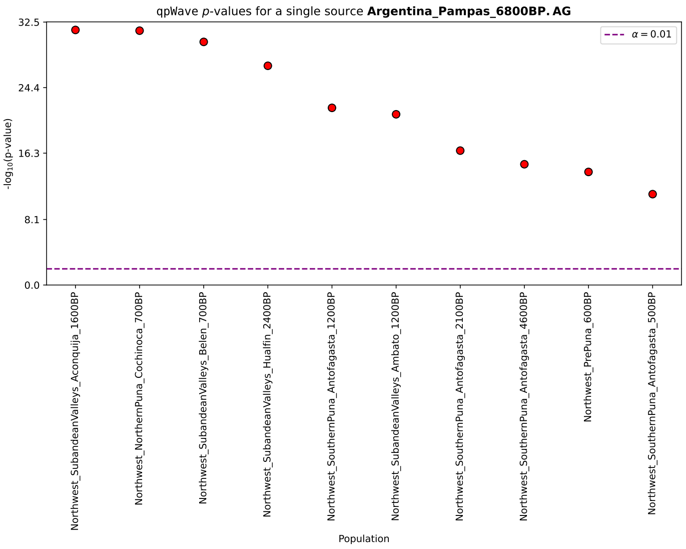

(c) Cladality with Middle Holocene Pampas

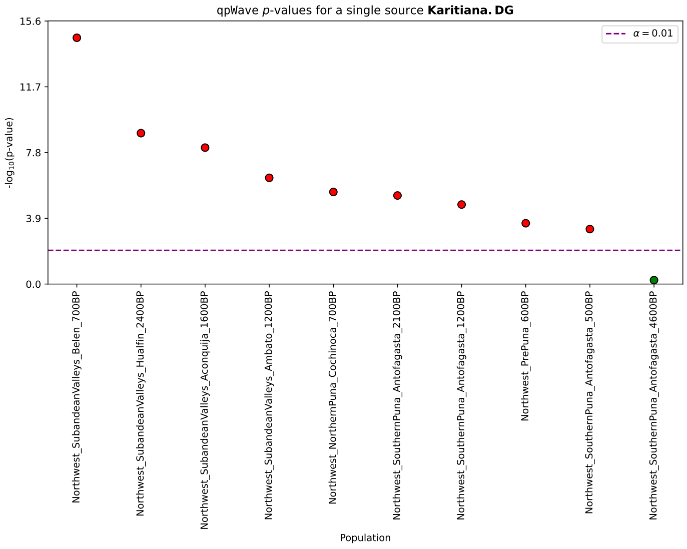

(d) Cladality with Karitiana (as a proxy for Tropical and Subtropical Forests ancestry)

Supplementary Figure 79: `qpWave` $p-$values for single-source models for Northwest populations (failures at $\alpha = 0.01$ are shown in red)

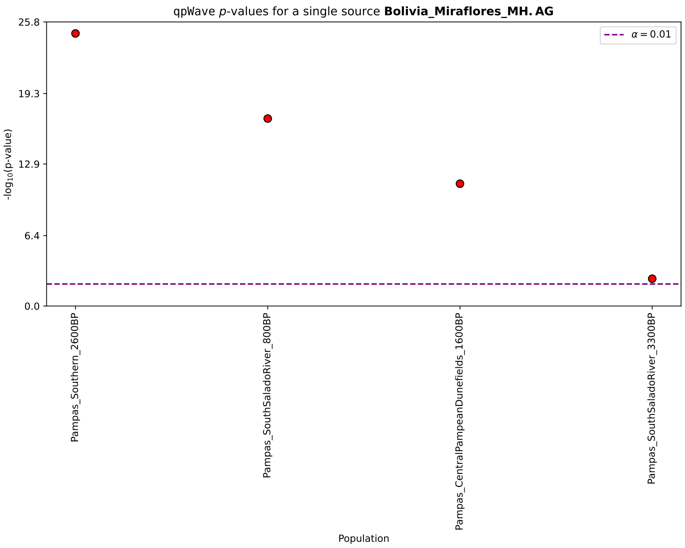

(a) Cladality with Central Andes

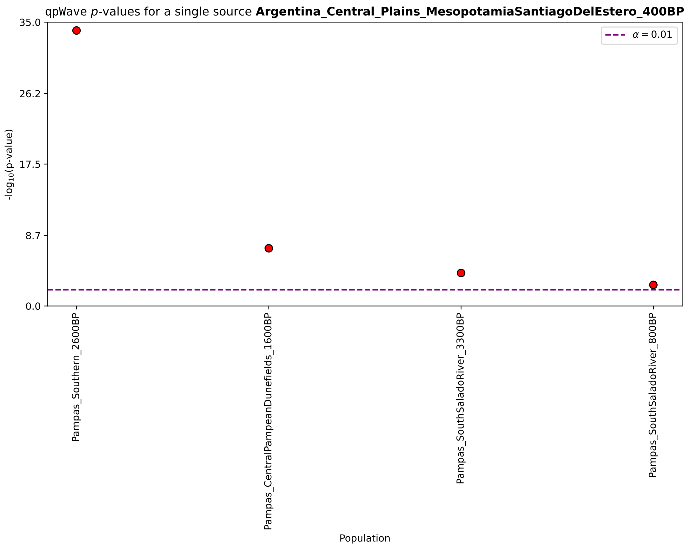

(b) Cladality with Central Argentina

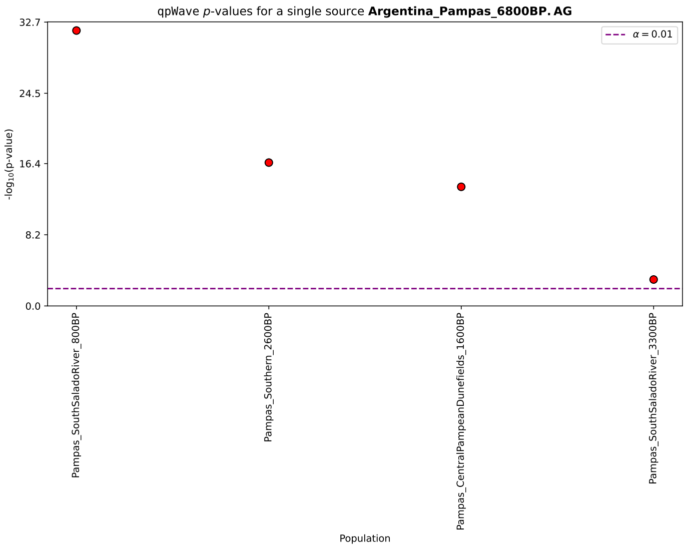

(c) Cladality with Middle Holocene Pampas

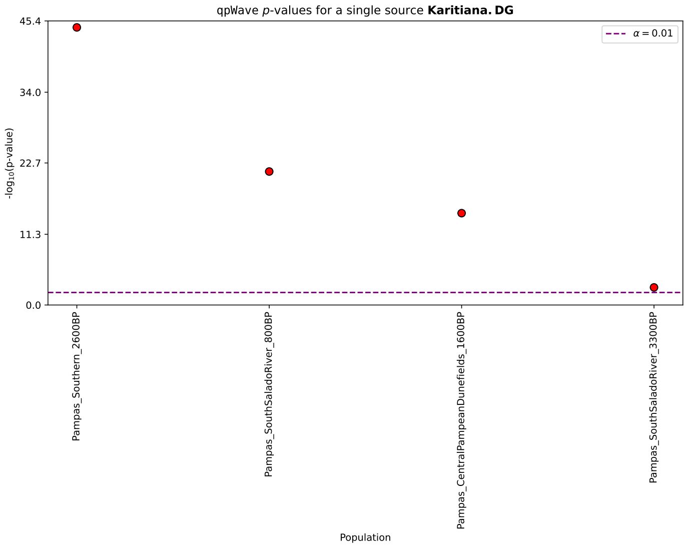

(d) Cladality with Karitiana (as a proxy for Tropical and Subtropical Forests ancestry)

Supplementary Figure 80: `qpWave` $p-$values for single-source models for Pampas populations (failures at $\alpha = 0.01$ are shown in red)

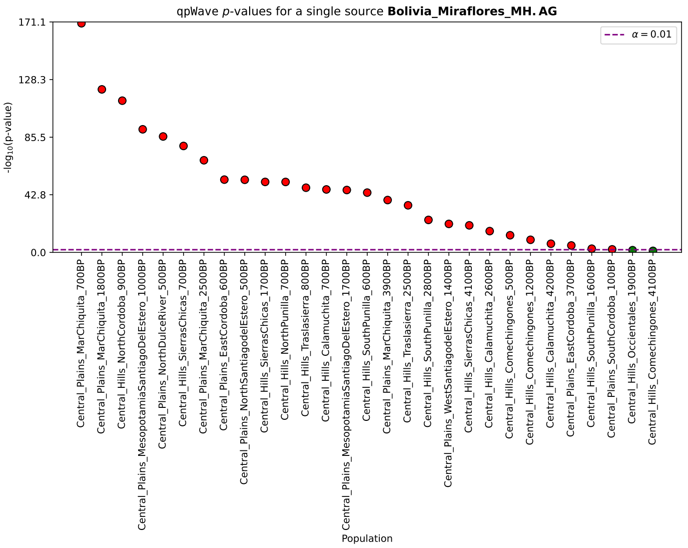

(a) Cladality with Central Andes

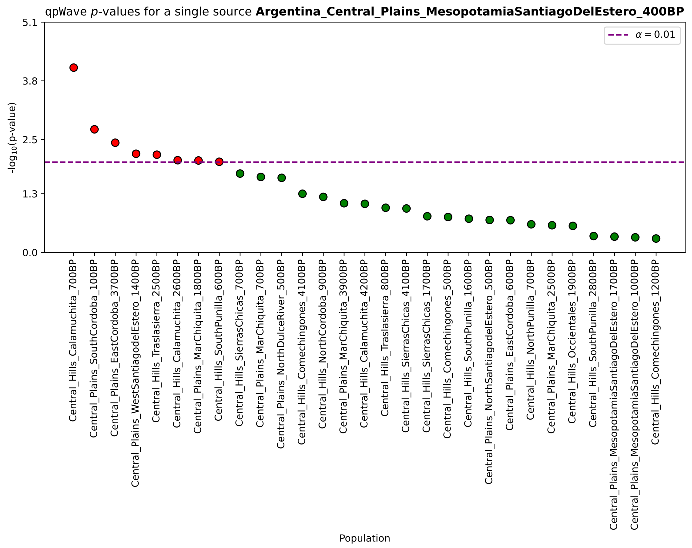

(b) Cladality with Central Argentina

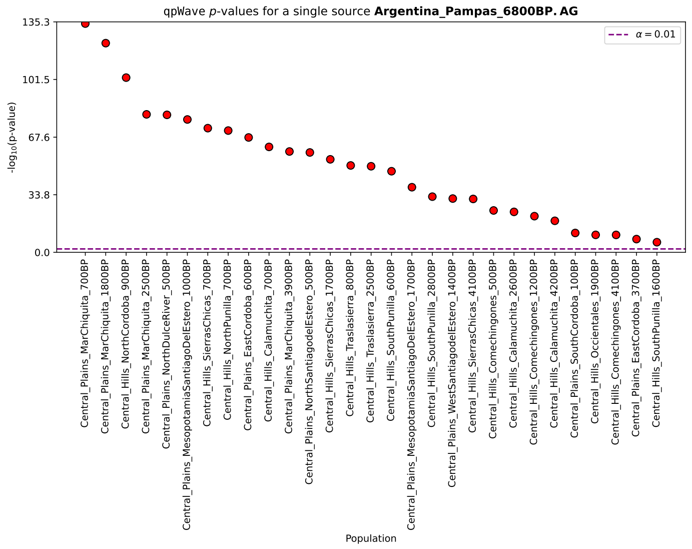

(c) Cladality with Middle Holocene Pampas

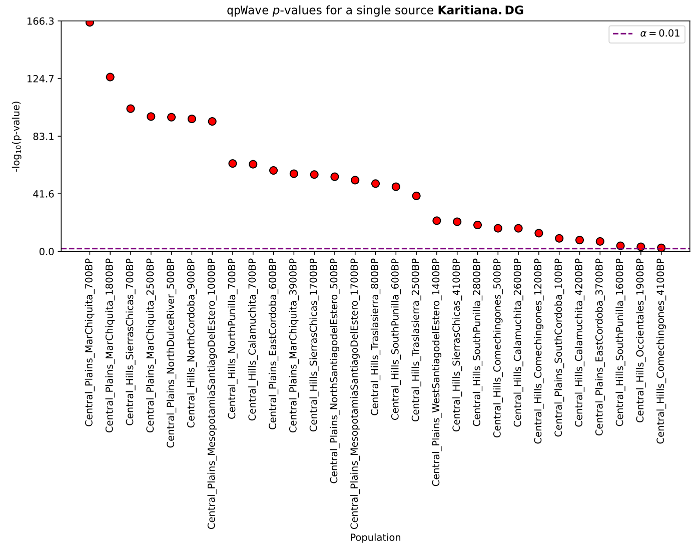

(d) Cladality with Karitiana (as a proxy for Tropical and Subtropical Forests ancestry)

Supplementary Figure 81: `qpWave` $p-$values for single-source models for Central populations (failures at $\alpha = 0.01$ are shown in red)

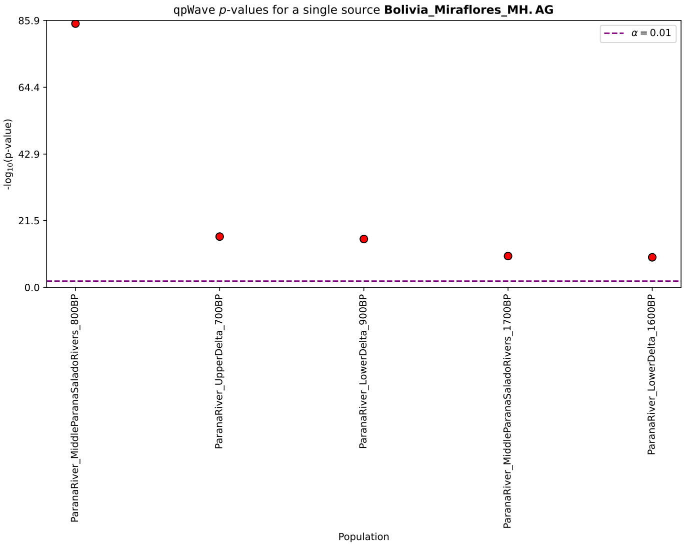

(a) Cladality with Central Andes

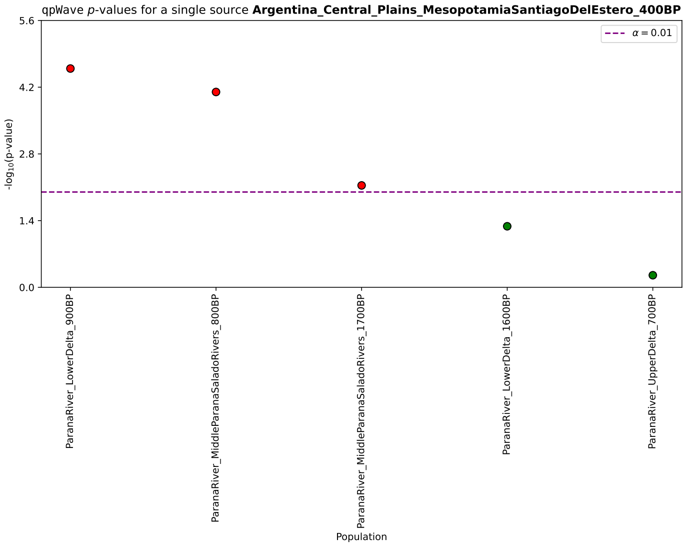

(b) Cladality with Central Argentina

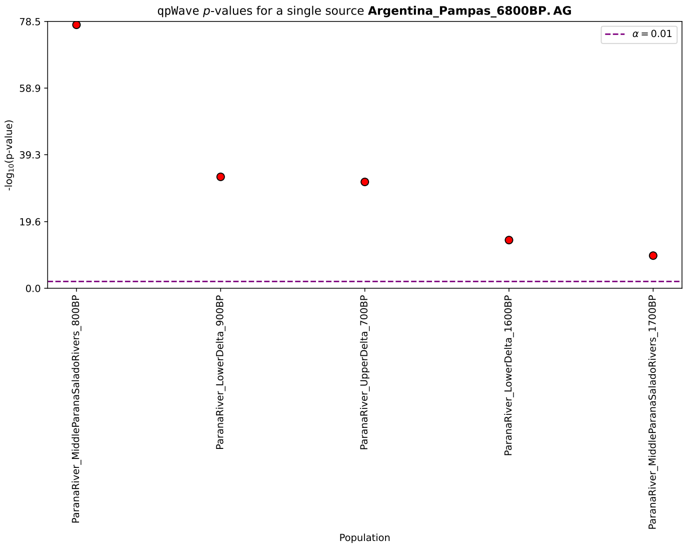

(c) Cladality with Middle Holocene Pampas

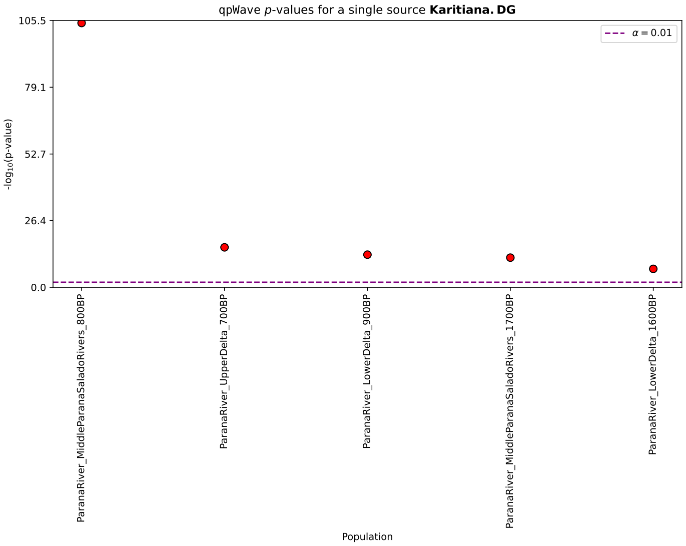

(d) Cladality with Karitiana (as a proxy for Tropical and Subtropical Forests ancestry)

Supplementary Figure 82: qpWave $p-$values for single-source models for Paraná River populations (failures at $\alpha = 0.01$ are shown in red)

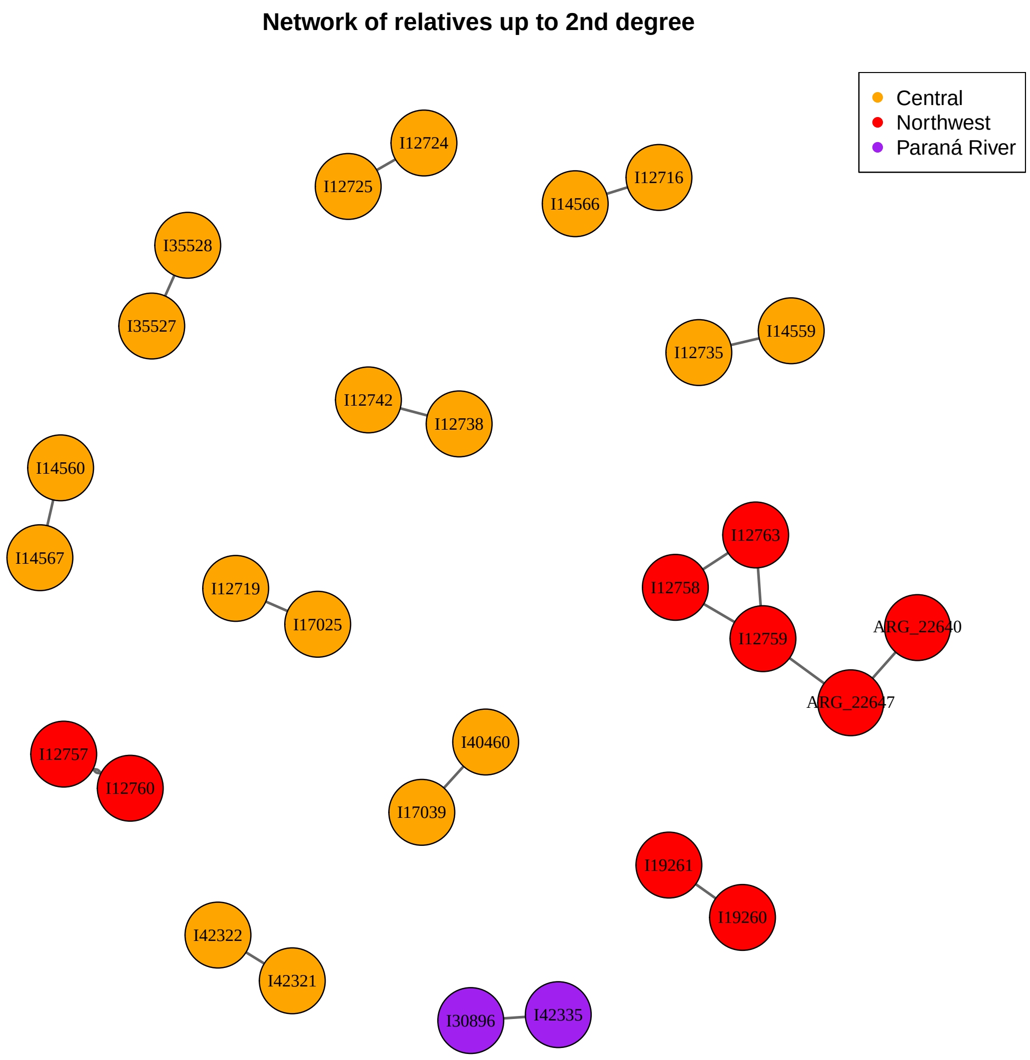

Supplementary Figure 83: Network of up-to-2nd degree relatedness connections

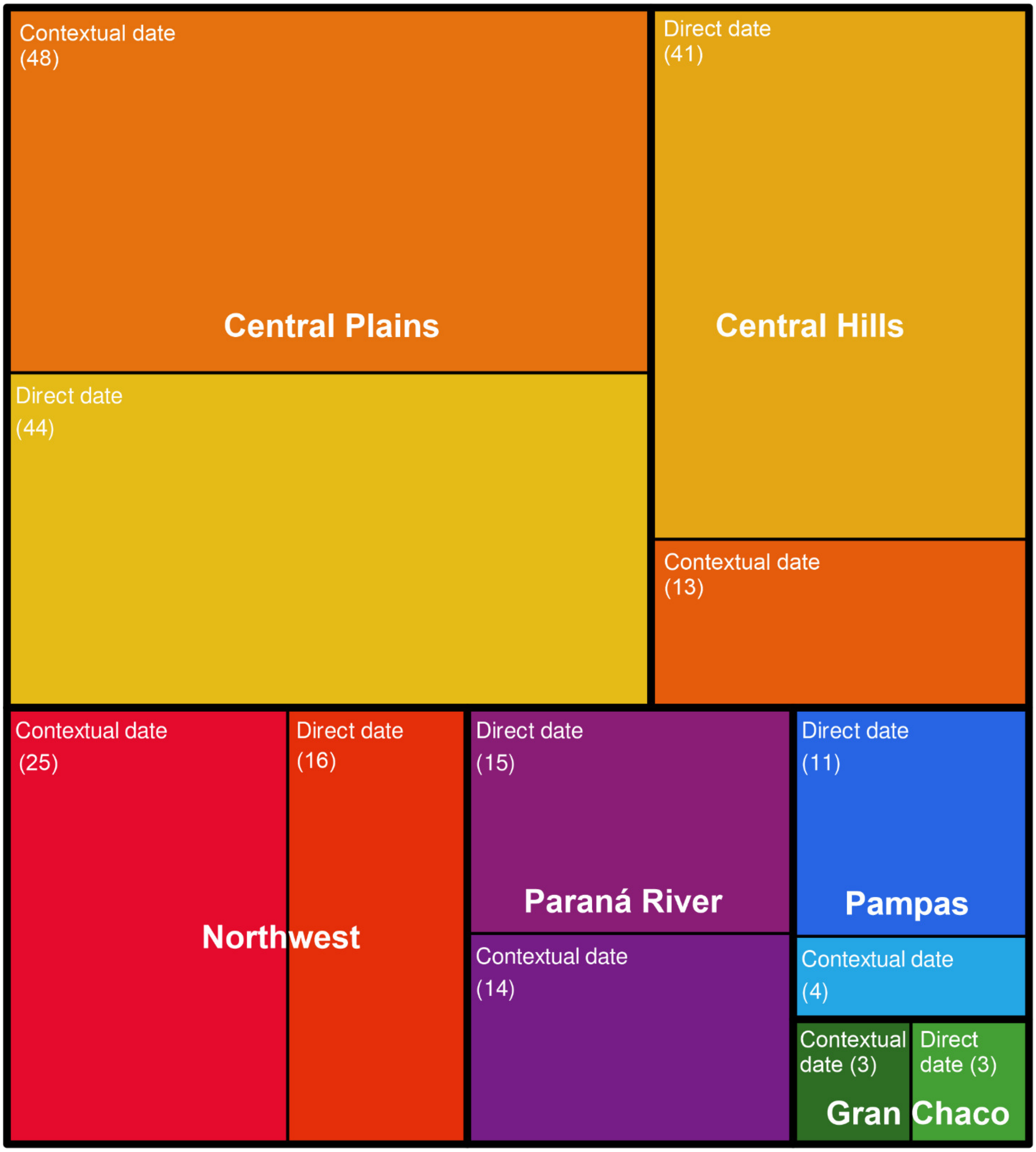

Supplementary Figure 84: Treemap representing the proportion of individuals dated directly by radiocarbon or estimated by contextual information, separated by region from Argentina. Number of individuals within each category are included in parenthesis.

SI Section 1. Summary of the sampling strategy, geographic origins of the individuals, and methods for chronological assignment

The sampling across the Central Southern Cone was designed to obtain as broad a geographical and temporal picture of the region as possible, given the available material, with particular emphasis on including Early/Middle Holocene (E/MH) individuals from each area. Our six-year sampling effort resulted in 344 samples from 310 individuals across 133 archaeological sites in six major regions. As expected, human remains from more recent periods were more abundant, while earlier samples exhibited a higher failure rate in yielding enough genomic data. Alongside the individuals for whom genomic information was successfully recovered (i.e., Pampas_LagunadelosPampas_10000BP, Northwest_PeñadelasTrampas1.1_8800BP, and Central_Hills_JesusMaria_8500BP), we also processed additional samples (Central_Hills_GrutadeCandonga_12300BP, Central_Hills_PotrerodeGaray_E/MH, Central_Plains_Fragua_E/MH, and ParanaRiver_ArroyoCululú_E/MH), none of which produced sufficient data for analysis. For the Gran Chaco region and the Paraguayan Pantanal, both known for later human settlement, the included samples (GranChaco_ElQuebracho_2200BP and Pantanal_Puerto14deMayo_1600BP, respectively) represent some of the earliest known human remains of these regions.

Of the 238 individual samples that passed quality control filters and were analyzed in this work, 55% have radiocarbon dates obtained through direct dating of human bone or teeth. For the remaining individuals, chronological attribution was based on direct association with other dated individuals, radiocarbon dates of associated faunal remains, or association with cultural materials with an established chronological framework (Supplementary Data 1). Supp. Figure 84 presents a comparative overview of all samples analyzed from present-day Argentina, showing whether the individuals were directly dated or dated by archaeological context. The only individual from Pantanal (Paraguay) is not included in the figure. The following paragraphs detail this information by region.

In the Central Hills, a total of 54 individuals were analyzed, of which 76% are directly dated. In the Central Plains, 92 individuals were studied. Here, the proportion of dated samples is lower: 48% of the individuals are directly dated, while 52% lack direct dating. However, the majority of the latter belongs to the Wagner Collection, from the Añatuya wetlands region. In this case, out of 36 samples, only 8 are directly dated, while the others share the same archaeological context. In Northwestern Argentina, 41 individuals were successfully analyzed, 39% of which have direct radiocarbon dates, while 61% do not. However, in all cases, individuals without direct dates are either directly associated with dated individuals or with funerary structures and ceramics that allow for confident chronological assignment. In the Pampean region, 15 individuals were analyzed, 73% of whom have direct radiocarbon dates. The undated individuals are directly associated with those that are dated. In the Paraná River region, 29 individuals were studied, with 51% directly dated. Once again, in all cases where individuals lack direct dates, they are associated with dated individuals from the same archaeological sites. In the Chaco region, a total of 6 individuals were analyzed, 3 of which are directly dated. The

remaining 3 undated individuals are associated with dated faunal remains. Finally, in the Paraguayan Pantanal, a single individual was analyzed, which is directly dated.

SI Section 2. NORTHWEST REGION (ARGENTINA)

Written by M. Pilar Babot, Salomon Hocsman, Paula C. Miranda De Zela, Lucia G. Gonzalez Baroni, Mariana Dantas, Germán G. Figueroa, Reinaldo A. Moralejo, Guillermo N. Lamenza, Jorge G. Martínez, Daniel E. Olivera, Diego M. Basso, Josefina M. B. Motti, and Rodrigo Nores

The Northwest region of Argentina (NOA) borders the current territories of Chile, Bolivia and a small strip of Paraguay. It comprises the provinces of Jujuy, Salta, Tucumán, Santiago del Estero, Catamarca and La Rioja. The region lies between latitudes 21°46' S and 32°00' S and longitudes 69° W to 64° W. The NOA region extends from the southern highlands of Bolivia in the north to the pre-Andean valleys of La Rioja in the south, and from the Western Andes mountain range in the west to the Yungas that descend towards the plains and the Chaco forest in the east. Because of that, it is characterized by a geographic diversity, with landscapes ranging from mountain ranges to plains, and from desert to humid and tropical areas (Raffino, 1999; Pommarés, 2017).

The Northwest region burial sites that provided the human samples analyzed in this work correspond to the mountainous arid and semi-arid areas in the NOA. Samples coming from the NOA plains in the province of Santiago del Estero, are considered and described here as part of the Central Region due to its geographical and archaeological links with the latter.

The NOA mountainous area is characterized by two sub parallel mountain ranges of North-South general direction, the Andes to the west and the Sierras Subandinas and Sierras Pampeanas to the east, which are separated by several Subandean ravines and valleys of lower altitude. The western side is higher than the eastern one. Ravines are long, narrow valleys that are the natural North-South and West-East communication routes in this area (Raffino, 1999; Pommarés, 2017). Due to the mountainous regions of the Andes has a mosaic distribution of biotic and abiotic resources, according to different altitudinal ecological zones, long-distance interaction and exchange networks were implemented to obtain them. These networks included, from very early on, different sectors of the NOA and access to distant regions such as the deserts and coast of northern Chile, the highlands of southern Bolivia, and the nearest plains of Tucumán, Santiago del Estero and Córdoba to the east and south. This led to the long-standing economic, cultural and social ties.

The pre-Hispanic history of Northwest Argentina is rich, diverse, and complex, marked by the presence of continuous human occupation from early hunter-gatherers, the last hunter-gatherers who led animal and plant domestication and the transition to food production, the early and late sedentary agro-pastoralist societies, to the Inca influence.

The oldest evidence of human occupation in the NOA region dates back to about 12,000 BP (Aschero, 1984; Somonte & Baied, 2017; Yacobaccio, 2017). The early inhabitants were highly mobile groups of hunter-gatherers located in the Puna high-altitude desert to the west and in the neighboring Subandean valleys and ravines to the east, who fed on a variety of animal species, such as wild camelids, rodents, and birds (Aschero, 1984; Martínez, 2014; Yacobaccio, 2017).

Starting 8,000-7,000 BP, arid conditions began, and patches of fertile areas with concentrated resources were occupied, along with a more regulated mobility strategy (Mondini et al., 2013). Under environmental conditions of extreme aridity in the Puna to the west and humidity in the valleys and ravines to the east, regional differences are observed among hunter-gatherer groups in rock art, ways of doing flaked stone artifacts, and the designs of projectile points among other lithic tools (Agnolin & Carbonelli, 2017; Aschero, 1998; Hocsman, 2014; Hocsman & Alderete, 2022; Martínez, 2014; Martínez et al., 2013; Podestá & Aschero, 2012; Restifo et al., 2019; Yacobaccio et al., 2008). Contemporary there is a pre-domestication intensification and specialization in the use of wild camelids (Yacobaccio, 2006).

Around 4,500 BP, current evidence suggests a transition from hunter-gatherer to agro-pastoralist societies, which involved the domestication of camelids, the management of cultivated food plants, the decline in residential mobility and the emergence of semi-sedentary lifestyles, residential stone architecture, the development of unfired pottery, and the increase in ritual and identity markers, among other aspects (Aschero & Hocsman, 2011; Babot, 2011; Hocsman & Babot, 2018; Oliszewski et al., 2018; Yacobaccio, 2006, 2021). This transition has also been linked to the presence of social complexity (Yacobaccio, 2004, 2006).

From approximately 3,000 BP to 1,000 BP, the agro-pastoralist way of life became predominant, with varying importance of agriculture, pastoralism, hunting and gathering, depending on the case. At this time, sedentary villages emerged, with a logistical mobility linked, on the one hand, to pastoralism, and on the other, to long-distance mobility associated with llama caravan trade. Technology of ceramics and metallurgy emerged at this moment (Korstanje et al., 2015; Olivera, 2001).

Settlements of the sedentary farmers spread across various Andean environments, including the northern and southern Puna, the Humahuaca and Toro ravines, and the main Subandean Valleys of Salta, Tucumán, Catamarca and La Rioja. In the *Yungas*, village groups inhabited eastern Jujuy, Salta, and northern Tucumán, particularly in the San Francisco and Bermejo valleys.

The agro-pastoralist societies of the NOA were highly variable in their material record, in terms of technology and ceramic styles, the designs of projectile points and other flaked stone tools, the characteristics of stone and earthen architecture and the shape of residential sites, and in the management of productive space (Korstanje et al., 2015; Olivera, 2001; Scattolin, 2010).

Although in the majority of cases it is considered that these are egalitarian societies, in some areas the existence of the Aguada societies has caused debates due to the differences that can be found with previous or contemporary societies (Figueroa & Dantas, 2020). According to Gordillo (2012), for many researchers there are sufficient elements in Aguada that allow them to speak of the emergence of lordships or chiefdoms, that is, complex societies with hereditary

social and political hierarchies (González, 1998; Pérez Gollán & Heredia, 1987; Pérez Gollán, 1991; among others). In contrast, other archaeologists characterize them as heterarchical societies, where there was an institutionalization of hierarchies, without centralization of power or unequal access to material and symbolic goods, based on reciprocity and not on domination (Cruz, 2007). A third position considers that, although it was a heterogeneous and internally differentiated society, where access to resources was differential (Laguens, 2007), it is not possible, currently, to try to define the type of political organization that prevailed there (Figueroa & Dantas, 2020).

From 1,000 BP to approximately 550 BP, large residential settlements were observed that had a strategic location, a larger size and various architectural and urbanistic patterns (Raffino, 1999; Tarragó, 2000). At this time there had been a demographic increase that led to an expansion of human occupation in the Puna and Subandean valleys and ravines. There was also an agricultural intensification, expanding agricultural fields and introducing more advanced irrigation and cultivation techniques by using terraces and canchones (Tarragó, 2000).

Tarragó (2000), among other authors, suggests the existence of chiefdoms with marked social inequality and institutionalized political, economic and social stratification. However, other proposals consider these societies as communal integration societies (Acuto, 2007) or corporate societies (Nielsen, 2006).

This moment is postulated as a time of conflictiveness for the control of natural resources and productive lands that resulted in the emergence of societies with well controlled and defended territories that entered into competition among them. The development of defensive sites (pukara) can be understood in this context.

From the early 15th century until the arrival of Europeans in the 16th century, the presence of the Inca State is recorded in the Northwest of Argentina (García et al., 2021, 2023; Greco, 2017; Leibowicz and Jacob, 2012; Nielsen, 1996, 1997; Palma, 2000; Williams and D'Altroy, 1998). This presence was characterized by the implementation of a centralized policy aimed at urbanizing the territory, integrating it through road networks, and practicing ceremonial hospitality toward conquered peoples. Within this framework, various architectural elements were constructed, such as *haucaypata* (plazas), *ushnu* (ceremonial platforms), *kallanka* (large rectangular structures), and *kanchas* (residential units with standardized designs), alongside an extensive road infrastructure, all conceived as material expressions of imperial power throughout the Tawantinsuyu (Hyslop, 1990; Nielsen and Walker, 1999; Raffino, 1983, 2007; Williams et al., 2005).

Bioarchaeological research in the NOA is scarce compared to that developed in other regions of the country (for example, Patagonia and Pampa). However, in recent years, there has been an increase in analyses focused on characterizing the lifestyle of the populations that inhabited this region, through the study of multiple variables. Some regions have received more attention in terms of health and nutrition studies (Quebrada de Humahuaca in the province of Jujuy; Quebrada del Toro in the province of Salta, the Calchaquí Valleys in the provinces of Salta, Tucumán and Catamarca, and the Northern and Southern Puna) compared to others (Santiago del Estero and La Rioja). Additionally, the analyses carried out on samples attributable to the Late

Period or Regional Developments (950 to 500 BP) are more numerous than those corresponding to earlier periods.

Regarding the analysis of metabolic-systemic stress indicators in Late Period sites, intra- and inter-regional variability is observed. The highest values of hypoplasia of the dental enamel (HED) were recorded in sites of Quebrada de Humahuaca (37.9% to 30%, Mendonça et al., 1992, Gheggi & Seldes, 2014). Similar but lower values are recorded in sites of Northern Puna (22.2%, Miranda De Zela, 2018) and in sites of Quebrada de Humahuaca corresponding to the Late Period after 1250 AD (24.1%, Gheggi & Seldes, 2014). The lowest values correspond to samples from Southern Puna (8.3%, Miranda De Zela, 2018), Quebrada del Toro (4.8%, Devoto & Perroto, 1973) and sites of Quebrada de Humahuaca corresponding to the Late Period before 1,250 AD, in which no cases were recorded (Seldes, 2006).

This distribution of values is striking, as much of the regional background has suggested that all these groups had a similar subsistence economy. However, it is important to highlight that the HED values recorded for the Quebrada de Humahuaca sites are associated with medium and high values of individuals with porotic hyperostosis (PH) (see below). Mendonça et al. (1992) relate the high prevalence of HED observed, along with the values of PH and *cribra orbitalia* (CO) detected, as an indicator that the affected individuals were exposed to nutritional conditions during their body development. According to Gheggi and Seldes (2014), this could be the result of population concentration processes in more densely populated sites (Gheggi & Seldes, 2014) and a high intake of corn (Seldes, 2006).

Regarding PH and CO, a great variety is also observed. The highest values correspond, to a greater extent, to sites in the Quebrada de Humahuaca (10 to 50%, Mendonça et al., 1992, Seldes, 2006) and the Northern Calchaquí Valley (PH: 21.4 to 53.3% and CO: 13.3 to 22.2%, Gheggi, 2011). As in the case of HED, Seldes (2006) proposes that the high values detected in Quebrada de Humahuaca would be related to a poorly varied and balanced diet focused on high corn intake. Other researchers (Gheggi, 2011; Mendonça et al., 1992) suggest that these individuals were more frequently exposed to infectious pathologies, nutritional deficiency, parasites, or an interrelationship between all of these, a situation that affected their health status, generating a physiological response that implied hypertrophy of the cranial hematopoietic tissue. This could be related to the moment of maximum demographic expansion that would have occurred in the NOA during the Late Period. That is, a scenario conducive to the generation of adverse situations for the general health status. This situation is not observed in other regions such as the Northern and Southern Puna, Quebrada del Toro, some sites in the Northern and Southern Calchaquí Valley, and in a few sites in Quebrada de Humahuaca, where very low values of PH and CO were detected (Gheggi, 2011; Miranda De Zela, 2018).

Regarding infectious processes, biological information indicates that in populations with subsistence economy types, such as those of the groups that inhabited the NOA during the Late period, lesion prevalences tend to be high (Cohen & Armelagos, 1984; Larsen, 1987; Powell & Cook, 2005; Tibayrenc, 2007, among others). However, the trends observed for the Northern and Southern Puna (Miranda De Zela, 2018, 2021), as well as in most sites of the Valles Calchaquíes, Quebradas de Humahuaca y Quebrada del Toro (Gheggi, 2006; Gheggi, 2011; Mendonça et al., 1992; Merlo et al., 2005; Seldes, 2006), do not align with this proposal, as low frequencies of

infectious processes at the bone level have been documented. The only exception would be the case of the Pukará de Tilcara site, located in the Quebrada de Humahuaca, where the prevalence of individuals affected by infectious lesions is 93% (Mendonça et al., 1992).

However, some evidence of systemic infectious processes has been detected: tuberculosis at the Rincón Chico 21 site in the province of Catamarca (Arrieta et al., 2011), and treponematosis at the Doncellas site and Northern Puna (Miranda De Zela, 2018, Miranda de Zela & Fuchs, 2019). According to some researchers, this situation could be a confluence of processes involving contacts between previously unknown populations, a drastic increase in occupation in already densely populated settlements, and much larger population displacements. These factors would have had a strong impact on various aspects of the health of local populations, including the spread of specific and non-specific infectious diseases (Arrieta et al., 2011; Luna et al., 2020). In the case of tuberculosis, Arrieta et al. (2011) propose that in Rincón Chico, the expression of the manifestations characteristic of a tuberculosis-type infectious disease occurred in a period between the Late Period and the effective Inca imperial penetration into the region. Therefore, they propose that the Inca expansion process that took place in the NOA may have determined potentially destabilizing situations that favored, if not the diffusion, at least the reactivation of a pre-existing tuberculous disease (Arrieta et al., 2011).

The analysis of dental pathologies presents disparity concerning the variables analyzed, as some (such as caries and periapical lesions) have received more attention than others. This first variable has been of utmost importance in the archaeology of the region, as the trends surveyed have been interpreted, to a greater extent, in relation to diet. The sites with the highest values correspond to Quebrada de Humahuaca. These values range between 79% and 100% (Mendonça et al., 1992; Seldes, 2006), while the lowest values correspond to some sites in the Northern and Southern Valle Calchaquí (Gheggi, 2011), Southern Puna (2.1%, Miranda Del Zela, 2018), and Quebrada del Toro (Gheggi, 2011). Regarding the Northern Puna, the observed trend could be considered moderate, in relation to the values observed in the other regions (Miranda De Zela, 2018).

The data obtained on carbon isotopic ratios (δ13C) for this region are associated with a diet characterized by resources with a C3 photosynthetic pattern, different from that of maize (C4) (Killian et al., 2012; Killian & Olivera, 2008; Pérez & Killian, 2011). Compared to sites in other regions of the NOA (Prepuna, Quebrada, and Valley) with similar chronologies, the isotopic data are less enriched in C4 values. Killian et al. (2012) have pointed out that in samples from the Northern Puna, the collagen δ13C values align with what is expected for consumers with a mixed diet. The results obtained could indicate access to meat obtained at high altitudes, from herbivores that graze above 3,900 meters above sea level and consume low proportions of C4 pastures (Killian & Olivera, 2008; Killian et al., 2012). Likewise, enriched δ15N values were recorded in the human bone record of samples from the Northern Puna. All values can be associated with diets with a high meat contribution (Pérez & Killian Galván, 2011). This has led to the suggestion that maize did not play a major role in the diet of the inhabitants of the Northern Puna.

The percentage of individuals with periapical lesions in Northern Puna (40%, Miranda de Zela, 2018) is similar to that recorded in samples from Quebrada de Humahuaca sites, where

values between 40% to 55% of individuals affected by various types of periapical lesions (abscesses and radicular cysts) were detected (Gheggi, 2011; Mendonça et al., 1992). Although this has been associated by researchers with a diet rich in carbohydrates, stable isotope analyses would indicate that this is not the case for Northern and Southern Puna (see previous paragraph). In contrast, in sites in Southern Puna, Valle Calchaquí Sur, Valle Calchaquí Norte, and Quebrada de Toro, the recorded values are lower (11% to 40%) (Gheggi, 2011; Miranda De Zela, 2018)

The etiology of periapical lesions is multiple (Dias & Tales, 1997; Hillson, 1996; Ortner, 2008, among others). However, the association of the frequencies surveyed with the presence of caries is dissimilar, given that the caries values recorded in the aforementioned regions are variable. In some cases (such as the Pucará de Yacoraite site, located in Quebrada de Humahuaca), researchers propose that the few cases of individuals affected with abscesses (16.6%) would be associated with the intense dental wear recorded and the inclusion of abrasive elements in the diet (Merlo et al., 2005).

Regarding the evaluation of interpersonal violence situations, in the Northern and Southern Puna, scarce evidence of traumatic lesions unequivocally attributable to such causes was observed, compared to the higher frequencies recorded in sites of Quebrada de Humahuaca and Valle Calchaquí (Gheggi & Seldes, 2012). The Puna is a particular case, as it has very few examples of elevated/defensive settlements (pukará) compared to the abundance of this type of site in surrounding areas (such as Quebrada de Humahuaca and Valle Calchaquí) (Ruiz & Albeck, 1997). Based on this, it could be suggested that the societies of the Northern and Southern Puna were subjected to a lower level of social conflict than that recorded for neighboring regions, and specifically much lower than what has been established for San Pedro de Atacama and Quebrada de Humahuaca (for example, Gheggi & Seldes, 2012; Nielsen, 2007; Seldes, 2006; Torres-Rouff et al., 2006). However, when comparing cases between regions, Gheggi and Seldes (2012) indicate that the individuals who inhabited Quebrada de Humahuaca were affected by a higher frequency of traumatic lesions than those of Valle Calchaquí, recording statistically significant differences. This would indicate that the populations of the Late Period conglomerates of Quebrada were subjected to a higher level of social conflict than their neighbors (Gheggi & Seldes, 2012).

2.1 Argentina_Northwest_PrePuna

Santa Rosa de Tastil: 1200-1450 CE

●**I19261** (D103L24, S2): 1283-1390 calCE (710±25 BP, UGAMS-68778)
●**I19260** (Cráneo 30, S1): 1200-1450 CE

Santa Rosa de Tastil is an archaeological site located in the Quebrada del Toro, Rosario de Lerma Department, Province of Salta. It is situated at 3,200 m.a.s.l. on the edge of the Puna, and comprises an area of 12 hectares at the top of one of the hills surrounding the Quebrada del Toro valley, with easy access to the Río de Las Cuevas. The site consists of the ruins of an urban settlement from the Late Period or Regional Development period of the Northwest Argentina

region (1,000 to 1,470 AD). The site was excavated by Eduardo Mario Cigliano and Rodolfo Raffino in the late 1960s. A total of 105 burials were found, most of them contained in circular constructions (cists) made from a single row of standing stones (Ribero, 2017). Two female individuals were included in this study, one of whom was a young individual with slight tabular deformation, more pronounced on the right side of the occipital.

2.2 Argentina_Northwest_NorthernPuna_Cochinoca

Doncellas: 1000-1500 CE

●**I34835** (DON108): 1422-1491 calCE (480±25 BP, UGAMS-68824)
●**I34837** (DON166): 1000-1500 CE
●**I34839** (DON189): 1000-1500 CE
●**I34842** (DON337): 1000-1500 CE

The Doncellas or Aguas Caliente site of Rachaite is located in the department of Cochinoca, Puna of Jujuy (22°49'12,28"S, 66°03'54,89"W). It is an urban settlement of the conglomerate type. In general, it is considered that this site, like others in the Puna of Jujuy, was closely linked to the Puna of Bolivia, the Quebrada de Humahuaca, the eastern valleys, and indirectly with the Chaco (Albeck, 1994; Palma, 1997-1998).

One of the greatest difficulties presented by the archaeology of the region is to establish clear differences between the Middle Period (ca. 600-1,000 AD,) and the Late Period or Period of Regional Developments (1,000-1,450 years AD). This is because there are no notable differences in the types of settlement, and there are no precise indicators of change in the decorative and formal patterns of the archaeological objects that allow them to be assigned to one period or another (Albeck, 2001). On the contrary, there seems to be evidence of population continuity between both periods with the occupation of the same residential spaces (Albeck & Ruiz, 1997). However, the dates carried out coincide with what has been proposed by different archaeological studies: the occupations would correspond, to a greater extent, to the Late Period (Albeck & Zarbulín, 2008; Alfaro de Lanzone & Suetta, 1970; Alfaro de Lanzone, 1988; Ruiz & Albeck, 1997).

During the years 1973, 1974, and 1975, Dr. Lidia Alfaro de Lanzone recovered a set of human remains, along with a large amount of archaeological material (Alfaro de Lanzone, 1988) that are currently part of the Doncellas Collection of the Instituto Nacional de Pensamiento Lationoamericano (INAPL) and the Museo del Hombre (located in the same institution). These remains underwent different processes that made it difficult to establish their provenance and association with precision. That is why, for the proposed bioarcheological studies, the set was treated as commingled remains.

The analyses carried out allowed us to establish that the sample is composed of 18 individuals. Higher frequencies of middle-aged adults (N = 10) and infants (N = 6) were recorded. Sex was inferred only based on adult individuals: females predominate (N = 6) (Miranda De Zela, 2013).

Regarding the health status, cases of periotic reactions were detected in the lower limbs (distal and proximal diaphysis and epiphyses of a right femur, two tibias, and three fibulas) of adult individuals. No porotic hyperostosis or *cribra orbitalia* were detected. Only one adult individual presents hypoplasia of the dental enamel (Miranda De Zela, 2021).

The traumatic injuries observed also correspond only to adult individuals. Ante mortem fractures were detected between the upper limbs (two radii with injuries compatible with fracture of Colles, one metacarpal, and one medial phalanx), in the rib cage (ribs with fractures in different stages of evolution: fully formed bone calluses and unconsolidated fractures) and in dorsal and lumbar vertebrae (deformation of their bodies in association with fractures and nodules of Schmörl). The fractures reported would not be the result of interpersonal violence but would be mainly related to accidental traumatic events that would be a consequence of the daily work activities that these individuals would have carried out. The proposal of a demanding lifestyle for these individuals is also supported by the large number of osteoarthritic injuries reported, which affected the spine and upper limbs to a greater extent (Miranda De Zela, 2021).

Regarding oral health status, a low frequency of caries and dental calculus was reported, although the levels of periodontitis and ante mortem tooth loss are considerable. In particular, it is proposed that the prevalence of ante mortem tooth loss recorded would be mostly correlated with the high frequency of periodontitis reported: alveolar retraction would have led to the supporting tissue not being healthy enough to support the teeth. This situation would have ultimately led to the loss of a large number of teeth (Miranda De Zela, 2018).

Cochinoca 1.4: 1250-1450 CE

●**I19252** (PVT3): 1250-1450 CE

One sample analyzed in this study comes from an excavation in Structure 2, located in the northwest sector of the Cochinoca 1.4 site, in the present-day town of Cochinoca (22°44.608'S, 65°53.866'W), at 3,658 m.a.s.l., Puna de Jujuy (Cochinoca Department, Jujuy Province). The excavation raises questions about the functionality of this space. Due to the stratigraphic complexity observed, the depth of the deposit, and the abundance of material waste, it can be suggested that the location experienced more than one occupation event, involving various activities. It is possible that the area was used as a burial sector, a waste deposit, and later as a residential area when the structure was built. Evidence aligns with occupations from the Late Period and possibly the Inca period (Basso, 2021). Human skeletal remains were found at a depth of 70 cm, consisting of a skull with a high degree of weathering, which made full extraction impossible. A date associated with the sample was obtained from faunal bone (620±40 BP, LP-3608).

Pueblo Viejo de Tucute. Recinto 1: 1163-1382 calCE

●**I19254** (PVT7): 1163-1382 calCE (810±50 BP, LuS-7358)

Pueblo Viejo de Tucute is an extensive pre-Hispanic settlement located south of Casabindo in the Cochinoca department, Jujuy Province at 23°1'29.15"S, 66°4'38.80"W (Albeck, 2010). One sample (I19254) analyzed in this study comes from a residential structure designated as Recinto 1, which was fully excavated. It reveals two cultural levels, one corresponding to the occupation period and the other to the abandonment phase, the latter characterized by a substantial accumulation of ceramic shards covering the floor. The individual corresponds to a perinate, buried at a depth of 57 cm as part of a ritual practice within a domestic context. Only the lower limbs were recovered. In another area of the same structure, a second, more complete perinate was found, though it was not analyzed in this study (Albeck et al., 2018).

Calaverioj: 950-1450 CE

●**I19253** (PVT4a): 950-1450 CE
●**I36288** (PVT4b): 950-1450 CE

The Caleverioj site (Cal-1) is a settlement located south of Casabindo (Cochinoca Department, Jujuy Province) at 23°1'23.63"S, 66°3'14.33"W, situated in a broad marshland area enclosed by rocky outcrops. The surrounding landscape is covered with cultivation terraces and features numerous walled caves that contained human skeletal remains. The samples analyzed come from one of these burial structures, known as "chullpas." Although these chullpas were looted, a few highly fragmented elements from the burials and associated grave goods were recovered; however, no remains were found to allow the identification of age, gender, or other indicators of the individuals. Two samples analyzed in this study enabled the identification of at least two individuals, one female (I36288) and one male (I19253). Only a small exploratory trench has been conducted at this site, within a rectangular structure, where a section of wall from a circular structure was observed at the base. Two dates have been obtained for this site: the upper levels (Cal-1 S: 1323-1496 calCE, 530±50, LuS-6735) correspond to the early 15th century, while the lower levels (Cal-1 I: 1330±50, 657-875 calCE, LuS-6734) correspond to the 7th century (Albeck & Zaburlín, 2008).

2.3 Argentina_Northwest_SouthernPuna_Antofagasta

El Coyparcito: 1300-1550 CE

●**I35522** (TC197): 1300-1550 CE

El Coyparcito is a defensive settlement located on the summit of the Cerros del Coypar, Antofagasta de la Sierra region, Catamarca province, in northwestern Argentina. While this site was identified originally as a defensive settlement during the Inca occupation of the region (Olivera & Vigliani, 2000-2002), later studies have characterized it culturally as a "mixed" site, due to the combination of local and Inca features ('Belén-Inca' style) (Killian Galván et al., 2021). A radiocarbon date performed on a sample of human remains (1,406-1,451 calCE, 529±26 BP;

Killian et al., 2021) would place the site in the Late Period or Regional Developments (ca. 1,000-1,480 A.D.) between the first half of the 15th century AD until the beginning of the imperial Inca occupation (ca. 1,480-1,536 A.D.).

In 2010, the structure corresponding to a circular/oval tomb was located on the slopes of the Cerros del Coypar. It measures 1.29 m (largest diameter) by 1.25 m (smallest diameter) and is built with superimposed volcanic stones (the presence of reddish mortar was observed between the stones). It is a burial site altered by looting activities that still presented a large quantity of human remains and associated archaeological materials. According to what was observed during the rescue tasks, it was proposed that the tomb was emptied of its contents and then part of the human remains and other archaeological materials extracted were redeposited along with sediment (Raices Montero et al., 2015).

The excavation of this structure allowed the recovery of a significant amount of human remains, as well as other archaeological materials (textile fragments, threads of various colors and thicknesses, gourd fragments, ceramic fragments, malachite beads and bone and dental remains of rodents). In the case of human remains, although a large amount was recovered, there were many missing parts due to the activity of the looters (for example: skulls and jaws). Given the difficulty of establishing associations of the remains with precision, for the proposed bioarchaeological studies, the set was treated as commingled remains.

The analyses carried out allowed us to establish that the group consisted of five adult individuals (one young adult female, two middle-aged adult males, and two young adults of indeterminate sex) (Miranda De Zela, 2018).

No alterations compatible with porotic hyperostosis or cribra orbitalia were found in the only skull in the sample. Neither were dental enamel hypoplasias observed on the only two teeth that this skull presents. However, within the group of loose teeth (N = 7), two with lines of dental enamel hypoplasia were detected (Miranda De Zela, 2018). Alterations compatible with non-specific infectious processes (localized periosteal lesions that would have been active at the time of the death of the individuals) were observed between the lower limbs: one tibia and four fibulae. Three cases of traumatic injuries were also identified, all antemortem: in the vertebral column (one case of spondylolysis in a fifth lumbar) and in the upper limbs (a right ulna and radius with evidence of a recovered fracture) (Miranda De Zela, 2018). Stable isotope analyses suggest a greater influence of C4 plants in the diet, than in other later sites (Killlian et al., 2011).

A large number of entomological specimens (pupariums) were recovered associated with the human remains, which would correspond to the group of flies, probably specimens of the Calliphoridae family (Oliva, 2013, personal communication, in Raices Montero et al., 2015). According to Gennard (2007), this family of Diptera is not found in buried bodies, so the hypothesis is suggested that the bodies recovered at the site were exposed to the elements for a certain time before being buried (Raíces Montero et al., 2015).

<u>Punta de la Peña 9:</u> 347 calBCE - 1146 calCE

●**I12763** / **ARG_22642** (EF47): 661-774 calCE (1356±27 BP, AA-107156)
●**I12759** (EF42): 640-845 calCE (1363±45 BP, AA-98365)

●**I12758** (EF41): 642-773 calCE (1391±27 BP, AA-107157)
●**I12757** (EF1A): 347-66 calBCE (2171±20 BP, AA-115019)
●**I12760** (EF48): 347-66 calBCE
●**ARG_22645** (E2): 500-700 calCE
●**ARG_22647** (E4): 890-1146 calCE (1090±50, UGA-15106)

The site of Punta de la Peña 9 (26°01'34.29"S, 67°20'33.19"W) is located in the Punta de la Peña archaeological locality, 13 km from the small town of Antofagasta de la Sierra in the department of the same name, in the province of Catamarca. Situated at 3,600 m above sea level on a plain along the left bank of the Las Pitas River, the site is bordered on the south by a large ignimbrite cliff. It is a multicomponent open-air site inhabited for 16 centuries (75-1559 cal CE) by the egalitarian agro-pastoralist Puna societies which were linked with peoples from the eastern Sub Andean valleys and Mountain forests and northern Chile through exchange networks by llama caravans. The site is composed of subcircular stone structures with varied functions, bead workshop areas, rocky promontories with incised rock art, bedrock mortars, and small rockshelters formed on the cliff talus. Sector I of the site consists of a broad esplanade extending from the talus border toward the river, containing a set of adjoining stone-walled enclosures where ritual commensality events took place between 516 and 895 cal CE (Babot et al., 2006; Babot & Martel, 2025; Medina Reguilón et al., 2025; Urquiza and Babot 2018). There are at least two funerary structures (EF1 and EF4) from which the analyzed human samples were obtained. Both funerary structures were constructed around small ignimbrite outcrops, dome-like in shape, and highly visible within their surroundings. These structures are slightly oval, formed by a single row of medium-sized rocks with flat faces turned inward, coated at their joints with a reddish plaster of silicate, clay, and silt. The floors were plastered with the same material, which was also applied above the contents of the tombs (Babot et al., 2009; Gonzalez Baroni, 2024; Gonzalez Baroni et al., 2019).

Funerary Structure EF1 constitutes an incomplete multiple secondary burial. From EF1, sample I12757 was obtained, corresponding to a female individual identified in this study (individual EF1A), aged between 20-50 years, dated to 2171±20 BP (AA-115019). Only the mandible of this individual was buried. EF1 also yielded the sample ARG_22642 from another female individual (individual EF1B), represented solely by the skull. However, this sample was genetically identified as a duplicate of individual EF47 from Funerary Structure 4 (Babot et al., 2009; Gonzalez Baroni, 2024).

Eight individuals of both sexes, adults and subadults, were buried in the Funerary Structure 4 (EF4). They were recovered both articulated and mixed and removed, forming an incomplete multiple secondary burial. Through the analysis of lateralities, the arrangement of each skeleton, and their spatial relationship, two burial events were identified (Gonzalez Baroni, 2024). For this study seven samples were obtained from EF4, corresponding to four individuals. Samples I12758/I12761/I12762 correspond to a single middle-aged adult male (35-50 years) (individual EF41), with a radiocarbon age of 1391±27 BP (AA-107157). This skeleton exhibits an anatomical completeness of 0.05% and was recovered dispersed with few articulated parts (Gonzalez Baroni et al., 2019). Sample S12759 belongs to a male individual, verified as such in

this study, aged 10-12 years (individual EF42), with an age of 1363±45 BP (AA-98365) and a low anatomical completeness index (0.03%), recovered dispersed with minimal articulation (Gonzalez Baroni et al., 2019). Sample I12760 corresponds to a young adult male (20-34 years) (individual EF48), with an estimated age of ca. 2171 BP based on a first-degree biological relationship with sample I12757, which dates to that period. The sample I12760 comes from an individual represented only by a fragment of the left maxilla. Sample I12763 is part of a female individual, corroborated in this analysis, aged 20-34 years (individual EF47) with a very low anatomical completeness index of 0.029%, dated to 1356±27 BP (AA-107156). As mentioned, it was a duplicate of individual EF1B (Gonzalez Baroni, 2024; Gonzalez Baroni et al., 2019).

Sector III of PP9 site is located between rock slides of the ignimbrite cliff talus. The human samples come from two funerary structures placed within two broad and multiple function structures named E2 and E4. Structure. The funerary context in E2 comes from the corridor area of this structure. It was recovered at a depth of approximately 15 cm from the surface and was constructed by excavating the ignimbrite outcrop itself and arranging medium-sized rocks that do not form a complete perimeter. Inside the structure, human bones were found scattered and unarticulated around the perimeter (López Campeny, 2001). From E2 comes the sample ARG_22645 from individual 1, male and with an estimated age between 5 and 7 years old with a very low anatomical completeness index (0.04%) (Gonzalez Baroni, 2024). The associated chronology from the dating of chañar endocarps from the funerary context is 1480±40 BP (UGA-9069) (López Campeny, 2001).

Structure E4 is located on a middle terrace approximately 10 m from E2 (Cohen 2005). The burial from which the analyzed sample ARG_22647 from individual 1 comes is located in the third occupation episode (layer 6 D). It is made up of three rocks inside which a bucket with loose sediment containing *guano* (camelid feces) and a fragmented urn were identified. The human remains were found dispersed between the urn and the perimeter rocks (Cohen, 2005). Individual 1, of indeterminate sex, is aged between 0-3 years, and has a very low completeness index (0.10%), with no evidence of pathologies (Gonzalez Baroni, 2024). The individual was dated 1090±50 BP (UGA-15106) (Cohen, 2010).

Punta de la Peña 13: 600-1000 CE

●**I12767** (PP131): 600-1000 CE
●**I12768 / ARG_22640** (PP132): 600-1000 CE

The Punta de la Peña 13 site (26°01'35.49"S, 67°20'28.58" W) is located in the Punta de la Peña archaeological locality, near the Punta de la Peña 9 site and belongs also to Puna agro-pastoralist societies. A funerary urn was recovered at the base of boulder-bearing rock paintings that depict a caravan of camelids from the Late or Late-Inca period (Martel, 2010). The urn was set within a roughly circular excavated structure, lined with flat rocks, and another individual was found within the sedimentary matrix of this structure (López Campeny et al., 2014). Samples I12768 and ARG_22640 are from the individual found in the urn (PP132), correspond to a primary burial of an early infant aged 0-1 years, female, with a completeness

index of 56.1% (Gonzalez Baroni, 2024). The urn was wrapped in a knotted vegetal fiber net, radiocarbon-dated to 1280±60 BP (LP-1723), calibrated to 666-964 calCE (López Campeny et al., 2014).

The other individual (PP131), found by the base of the urn within the interior of the excavated funerary structure, provided sample I12767. This individual is also an early infant (0-1 years), male, secondarily buried with a completeness index of 16.1% (Gonzalez Baroni, 2024).

Punta de la Peña Farallón Extremo Suroeste 1: 390-574 calCE

●**ARG_22664_66** (PPFES3-X): 390-574 calCE (1630±32 BP, AA115020)

The Punta de la Peña Farallón Extremo Suroeste site (26°1'57.43"S, 67°20'40.36"W) is located at the southwest end of the long ignimbrite cliff of the archaeological locality of Punta de la Peña. PPFES site is a cavity in between rocky blocks to which a stone wall was added, incompletely closing its perimeter. Inside, a deposit of human bones was recovered, with an NMI of 4 individuals. The sample ARG_22664_66 comes from individual 3 and individual X, which turned out to be part of the same individual 3 (PPFES3-X). This Individual, male, with an estimated age between 20-24 years old, has a very low completeness index (0.087%). Radiocarbon dating of individual 3 is 1630±32 BP (AA115020) (Gonzalez Baroni, 2024).

Peñas Chicas 1.7: 2876-2497 calBCE

●**ARG_22633_34** (PCh1.7A): 2876-2497 calBCE (4145±45 BP, AA107159)

The Peñas Chicas 1.7 site (26°01’48.62”S, 67°21’10.68”W) is a rockshelter located at 3563 masl in the Peñas Chicas archaeological locality, 1 km from Punta de la Peña 9 site. PCh1.7 human occupations correspond to the transition from hunter-gatherers to agro-pastoralist societies (Hocsman & Babot, 2018). It presents a residential occupation inside and outside a stone structure that develops over a lower funerary event. This is an indirect primary burial, delimited by a stone structure that possibly formed an enclosure and enclosed the burial site from the front. The skeleton lay in an anatomical position, supine with the limbs semi-flexed and the upper limbs resting on the pelvic girdle.

Sample ARG 22633_34 is made of two separately analyzed anatomical parts that belong to the same female individual A, whose sex was corroborated in this investigation. She is between 20-24 years old and it was dated 4145±45 BP (AA107159). The completeness index is 84%, indicating high skeletal integrity (Gonzalez Baroni, 2024).

Peñas de las Trampas 1.1: 7043-6698 calBCE

●**I12756** (PT1.1:I3-FS2, CAT): 7043-6698 calBCE (8000±30 BP, UGAMS-4999)

Peñas de las Trampas 1.1 site (26°01´14"S, 67°21´06"W) is a rockshelter located at 3,582 m.a.s.l. in Antofagasta de la Sierra (Catamarca province), in the southern Argentinean Puna. This site yields a peculiar concentration of stratified materials covering contextually different moments ranging from ca. 19,600 to 4,000 BP (non calibrated; Martínez, 2014). Given the extremely cold and dry conditions of the Puna environment, all types of organic materials have been exceptionally well-preserved. These conditions are valid for botanical macro-remains that make up the feces of megafauna (range 19,600-12,500 BP) and human remains. It corresponds to the earliest mortuary practices in this area for the period ca. 8,400-8,000 BP. Inside this rockshelter, two funerary structures -Funerary Structure 1 and 2- were detected. They are both very similar and consist of suboval-shaped diggings carefully lined with grass (*Grammineae*) sheaves at the sides and bottoms, with no lid. Inside both structures human remains were found forming multiple secondary burials, the analysis yielded a minimum number of three individuals for each structure (i.e., six in total). These human remains are associated with highly complex handicrafts, such as suede leather pieces (sewn, dyed and painted), numerous necklace beads made from non-local seeds/fruits, marine mollusc shells from Pacific Ocean and strings and fragments of red dyed mesh or net, made from non-local plant fiber (Martínez, 2014). The sample under analysis (I12756) belongs to Individual 3 of Funerary Structure 2 (FS2), which was dated to 8000±30 BP (UGAMS-4999). The bone assemblages recovered are in an excellent state of preservation in both structures, but the majority of the skeletal parts are absent. We consider that the missing skeletal parts are the result of cultural practices, given that some bones had postmortem anthropic marks and almost no signs of carnivore traces. Sex determination was impossible due to the young age of the individual and the absence of diagnostic skeletal parts. The estimated age for Individual 3 was 1 year 8 months±3 months (Martínez, 2014). Stable isotopes of Carbon (δ13C) and Nitrogen (δ15N) analysis were aimed at defining aspects related to the paleo diet of the six individuals. For the case of individual 3 (FS2), the following values were obtained: δ13C(col)= -18.35‰, δ13C(ap)= -14.17, and δ15N= 13.2‰. These values indicate a diet rich in animal proteins within the paleo-economic subsistence spectrum typical of hunter-gatherers. It takes into account the fact that this individual was a breastfed infant, therefore the isotopic data represents their mother's diet (Killian Galván et al., 2016).

2.4 Argentina_Northwest_SubandeanValleys_Belen

El Vallecito: 900-1420 CE

●**I40458** (MSH-EV-CAJA15, 5S): 1250-1650 CE

A sample included in this study (I40458) comes from an isolated skull of an adult male individual. It was discovered incidentally by Manuel Morales in El Vallecito in the area known as La Aguada (Londres, Belén Department, Catamarca Province; 27°43'5.57"S; 67°15'4.81"W). Based on other findings from the region, it can likely be assigned to the Late Period or Regional Developments (900-1420 CE). This skull is currently guarded at the El Shincal Museum in Londres, Catamarca.

<u>Londres:</u> 900-1420 CE

●**I39328** (MSH-Ind.Inf.-CAJA18, 6S): 900-1420 CE
●**I42331** (MSH-ParvuloSJ, 8S): 900-1420 CE

Two samples included in this study come from incidental finds near the town of Londres (Belén Department, Catamarca Province). The exact location of the finds is unknown. Based on other findings from the area, they can likely be assigned to the Late Period or Regional Developments (900-1420 CE). Both individuals are currently housed at the El Shincal Museum (Londres, Catamarca). The first sample (I39328) is from a subadult individual, likely male. The remains are complete and in good condition. The second sample (I42331) corresponds to a young child associated with a "San José" style funerary urn.

<u>El Shincal de Quimivil:</u> 900-1536 CE

●**I12744** (3S, MSH-SH-T1-CAJA15): 1420-1536 CE
●**I40461** (4S, MSH-SH-T2-CAJA15): 900-1420 CE

The El Shincal de Quimivil site is an Inca political, administrative, and ceremonial center located at an altitude of 1,350 m.a.s.l. in the town of Londres (Belén Department, Catamarca Province). Near the proximity of this center, the remains of two individuals were found and analyzed in this work.

One sample (I12744) comes from an individual whose skeleton was the subject of an archaeological rescue operation following a fortuitous discovery on the property of a local resident (Mr. Maximiliano Varas) in the Belén Department, Catamarca Province. The burial was located 1.2 km in a straight line southwest of El Shincal de Quimivil (27°41'32.42"S; 67°11'17.69"W). It was found within a "shaft tomb," with a vertical conduit and a lateral burial chamber. The human skeletal remains were well-preserved and corresponded to an adult male (40-50 years of age) with cranial deformation caused by external compression during life (Salceda & Raffino, 2004). The individual was found kneeling, accompanied by two ceramic pieces: a *puco* (bowl) in the Yavi or Chicha Black-on-Buff style and an aryballoid vessel in the Inca Provincial style.

Another sample (I40461) also corresponds to an archaeological rescue operation, following a chance discovery, conducted on the property of a resident of Londres (Mr. Luis Alvá) in the Belén Department, Catamarca Province. The burial was found 1.6 km in a straight line to the west of El Shincal de Quimivil and near the Quimivil River, beneath a rocky outcrop (27°41'15.20"S; 67°11'41.84"W). It lacked funerary architecture. The skeletal remains were poorly preserved and corresponded to a young adult female (24-30 years old) (Salceda & Raffino, 2004). The skeleton was found in a kneeling position and was accompanied by a *puco* or bowl in the local Belén Black-on-Red style.

2.5 Argentina_Northwest_SubandeanValleys_Aconquija

Aconquija: 435-599 calCE

●**I19246** (43-2889, A13): 435-599 calCE (1570±25 BP, UGAMS-68777)

A skull along with its corresponding mandible, originating from a burial site, was discovered during the construction of a national road in Aconquija (Andalgalá Department, Catamarca). It was collected by Mr. M. A. Aliaga, an employee of the National Highway Authority. According to the laborers involved in the roadwork, the skeleton associated with this skull was found in an inverted position, with its feet pointing upwards. Since the remains were recovered by amateurs, detailed contextual information is unfortunately lacking.

El Mollar: 229-365 calCE

●**I19263** (Enterratorio 2, T2): 229-365 calCE (1790±25 BP, UGAMS-68779)

The site is located in Tafí del Valle Department, Tucumán province, and was excavated by Osvaldo Heredia in the 1970s. The skeleton was recovered from a mound, but there is no more precise information about the discovery context. This individual is currently housed in the Patrimonial Reserve of the Museum of Anthropology (FFyH, UNC).

Alamito: 50-450 CE

●**I19244** (64-157, A11): 50-450 CE
●**I19245** (4 NW, D95L7, A12): 50-450 CE

Both individuals were found in Alamito, Andalgalá Department, Catamarca, during an expedition conducted by Víctor Núñez Regueiro in January 1966. I19244 corresponds to the skeleton of a child, found alongside blue, light blue, and white necklace beads. I19245 corresponds to an adult individual buried in the northwest section of Room 4.

2.6 Argentina_Northwest_SubandeanValleys_Hualfin

Cardón Mocho: 798-67 calBCE

●**I19226** (3CM): 387-67 calBCE (2220±60 BP, LP-2162)
●**I12944** (6(1)CM): 752-181 calBCE (2360±80 BP, LP-2981)
●**I12945** (17CM): 798-418 calBCE (2550±60 BP, LP-2970)
●**I34671** (18CM): 800-400 BCE

The Cardón Mocho site is located at the foot of Cerro Durazno, on the western slope of the Hualfín Valley near the town of Azampay (Belén Department, Catamarca) -27°19'48''S; 67°3'0''W-. This pre-Hispanic cemetery includes distinctive construction features, involving one or more individuals placed within simple structures defined by rows of rocks of varying sizes, juxtaposed with each other. Numerous malachite beads, mother-of-pearl figurines, a copper plaque, and a wooden figurine were recovered alongside subadult individuals. Noteworthy are a pendant made from a marine snail shell from the Pacific coast (*Oliva peruviana*) and a stone mask. The pendant suggests long-distance connections, while the mask is of particular importance for discussing the presence of high-value symbolic luxury items associated with ascribed status.

2.7 Argentina_Northwest_SubandeanValleys_Ambato

El Polear 1: 660-900 calCE

● **I34834** (I1, A15): 650-900 calCE

El Polear 1 is an open-air mound site with constructions measuring 30 by 40 meters, situated in a depressed area in the northern part of the valley at 1,095 meters above sea level. Its coordinates are 27°56’43.50”S and 65°49’48.11”W (Dantas et al., 2019). Radiocarbon dating of camelid bone (1290±20 BP, YU-17885, 686-871 calCE, Dantas et al., 2025), combined with recovered pottery styles and the architectural characteristics of the site, confirms that it corresponds to Aguada occupations. It is worth noting that the human skeletal remains recovered from the site were not found articulated, nor in defined primary or secondary burials; instead, isolated elements were identified, scattered at floor level and in fill across different sectors of the site (Tavarone et al., 2023). An upper left premolar (I34834) from the skull of an adult individual of undetermined sex (M. Fabra, 2024, pers. comm.) was analyzed. The skull was found intact, positioned on a ceramic fragment, and in direct association with a calcaneus, a rib fragment, and a human mandible. The skull shows cut marks on the occipital bone, near the foramen magnum.

Martinez 4: 600-1000 CE

● **I19240** (I6, A7): 600-1000 CE
● **I19241** (I4, A8): 600-1000 CE
● **I19242** (I3, A9): 600-1000 CE

The Martinez 4 site is located at the valley floor in the area of Rodeo Grande at an elevation of 1,091 m above sea level, with coordinates 27°59'22.87" S and 65°49'22.91" W. It is a small, trapezoidal site measuring 10 meters east-west and 17 meters north-south (Herrero and Ávila, 1991). The site comprises a room and a gallery or patio area. It is spatially and temporally integrated with the Martinez 2 site (Juez, 1991), a large site like El Polear 1, which has

radiocarbon dating performed on charcoal remains of 990±70 BP (LP-1317), 1690±80 (LP-444), and 1510±70 BP (LP-558) (Bonnin & Laguens, 1996; Marconetto, 2008). Similar to El Polear 1, the human remains recovered within the rooms were not found in primary or secondary burial contexts, but their association with pottery fragments and camelid bone specimens was identified. Baffi and Torres (1996) were able to calculate a minimum number of individuals (MNI) of seven, three of whom were subadults and four adults. Among the adults, two were assigned as male, one as female, and one remained undetermined. Baffi and Torres (1996) note that all teeth show wear, and hypoplastic lines in the dental enamel of the canine suggest episodes of nutritional stress experienced before the age of 9. The analyzed samples are as follows: I19240: left maxilla fragment from an adult female; I19241: maxilla in two fragments from an adult male; I19242: two fragments of the left mandible from a subadult.

At this site, some bone specimens show signs of cutting and/or burning, similar to the individuals recovered at the Martinez 2 site, leading Baffi and Torres (1996) to suggest similarities in the treatment of human remains. Various hypotheses have been proposed regarding this aspect of these two sites, ranging from association with sacrificial practices (Herrero & Ávila, 1991; Juez, 1991; Pérez Gollán, 1991) to the possibility of practices involving anthropophagy and the conservation of remains as relics or offerings (Cruz, 2004, 2006; Gordillo & Solari, 2009).

Uturunco 1: 600-1000 CE (Context, Archaeological material culture)

● **I34833** (I1, A14): 600-1000 CE

The site Uturunco 1 is located in the locality of Los Potrerillos, on the foothills of the western slope of the Ambato Valley (Ambato Department, Catamarca Province). It is an open-air mound site with constructions, approximately 40 meters on each side. Its coordinates are 27°57'2.16''S and 65°51'48.48''W, at an elevation of 1,170 m above sea level. Currently, a private home is built on its upper portion. The owner mentioned that various archaeological materials, such as ceramics, lithics, and bone fragments, often appear on the surface. The material analyzed in this study is the only one that could be found from rescue efforts conducted in a pit located outside the current house. This site has no absolute dating, but materials found on the surface and recovered during the construction of the modern house suggest that it was occupied during the Aguada period. As a result of the rescue work, a skull and a mandible of an adult male individual were recovered (Fabra, 2019). The second lower left molar (A14) was selected for analysis.

SI Section 3. GRAN CHACO REGION (ARGENTINA) AND PANTANAL (PARAGUAY)

Written by Guillermo N. Lamenza, Josefina M. B. Motti, and Rodrigo Nores

The South American Gran Chaco encompasses a vast territory that exceeds 1,141,000 km^2, located in the south-central portion of the continent. In terms of extent, it is the second largest forested region in South America after the Amazon rainforest. It stretches from 16°55' south latitude, in the tropical region, to 33°52' south latitude, in the temperate region, and from 67°50' west longitude at the foothills of the Andes, to 57°52' west longitude in the province of Corrientes (Naumann, 2006). Geomorphologically, the Gran Chaco is a plain formed by the large alluvial fans of the Salado, Bermejo, Pilcomayo, and Parapetí rivers, with a Pliocene age (5 to 2 million years) for its biogeographic identity (Iriondo, 2006). Thus, it is included within the vast Neotropical Region, Chacoan Domain, Chacoan Province (sensu Cabrera & Willink 1973). Additionally, it is an extremely flat area, with very gentle west-east slopes, resulting in extensive flooding that keeps a wide surface area waterlogged for several months in wet years (Iriondo, 1999).

Moreover, this minimal altitudinal difference, the seasonal torrentiality of the rivers, and the aeolian and fluvial contributions create a moderately irregular local topography, with levees elevated compared to the surrounding floodable areas (Ginzburg & Adámoli, 2006). These levees form the only emergent geomorphological features in areas where extraordinary floods occur (Iriondo, 1999). Currently, the Chaco climate is tropical semi-arid to humid, characterized by a permanent exchange of tropical and austral air masses. Temperatures range between 24°C and 30°C in summer, with maximums exceeding 40°C. In the eastern sector, higher air humidity makes the thermal amplitude milder than in the west (Iriondo, 2006). The rainy season corresponds to the warm season and is concentrated from October to April. The minimum precipitation occurs during the winter season, from June and August, bringing droughts and occasional frosts (Ginzburg & Adámoli, 2006).

In archaeological terms, recent research has proposed that throughout human occupation in the Gran Chaco, variations have occurred in resource exploitation strategies, settlement patterns, mobility, and technology. These changes occurred in relation to historical trajectories that articulated the environment, interaction systems, and ethnogenesis processes with their differential changes and continuities from the initial settlement of the region to the present (Lamenza et al., 2019). The pre-Hispanic Chaco cultural dynamics have been building a landscape in interaction with a changing environment in close relationship with its neighboring areas. Integrating with the general paleoenvironmental model, we now know that the earliest evidence of human activity dates to the Climatic Optimum within 7,000 and 8,000 BP. This archaeological evidence indicates differentiated human societies in terms of occupation strategies, but it is still not confirmed whether they were related to each other or came from different places of origin (Alfonso Monges & Lamenza, 2021). Subsequently, we encounter an archaeological silence during the middle Holocene, where arid to semi-arid conditions with geomorphological instability were established (May et al., 2008). In the late Holocene, geomorphological stability returned with the expansion of forests and the formation of meanders. Additionally, fluctuating climatic variations include aeolian activity and dune formation (Arellano, 2014). Between ca. 3500 and 1400 BP, the paleoclimatic model characterizes the region with a semi-arid climate, with greater thermal amplitude, deep water tables, and absence of forests (Iriondo, 2006).

During this period, there is a restricted exploitation of rivers and a dispersed settlement pattern with a wide range of action; the use of watercourses as corridors is emphasized, facilitating communication with other neighboring areas (Lamenza et al., 2019). Here, the presence of the Pantanal - Chaco Archaeological Entity begins to be evident, where its characteristic ceramic component (non-Guarani corrugated pottery) shows a displacement from the Pantanal towards the south along both banks of the Paraguay River to its confluence with the Paraná River, towards the west through all its tributaries, and along the western axis also in a north-south direction along the piedmont forest edge (Lamenza, 2013; Lamenza et al., 2019). Throughout the temporal sequence, these populations, whether related or not, with the Chané or another Arawak-origin people such as the Matará or Tonocoté, inhabited the interior of the Chaco during a time of environmental prosperity (Medieval Warm Period), likely with semi-sedentary stable settlements and extensive interaction networks with other societies from the central Argentine plains and western forests (Lamenza & Alfonso Monges, 2020). This period, between ca. 1400 and 800 BP, corresponds to a generalized increase in humidity with a tropical climate and abundant rains throughout the Gran Chaco (Iriondo, 2006). On the other hand, in recent centuries, the Little Ice Age affected the Lowlands (Iriondo & Kröhling, 1995) with a general semi-arid environment with predominant south winds (Iriondo, 2006). To this scheme, the consideration of extraordinary El Niño-Southern Oscillation (ENSO) events, which produced and continue to produce significant climatic changes worldwide causing droughts, floods, and temperature increases (Díaz & Markgraf, 1992), should be added. These events must have influenced the organization of pre-Hispanic Chacoan societies in some way. Recent research is aimed at answering whether these environmental changes and extraordinary events may have integrated processes of extreme drought in peripheral areas, restricting resource availability, prompting population displacements, and changing subsistence strategies. Conversely, during periods of increased rainfall, extraordinary floods in the Chaco area favored changes during major rivers and/or their tributaries (Lamenza et al., 2019). Later, with climate change oriented towards greater humidity, it confirms the intensification of the regionally restricted pattern, with higher population density and an interaction system with neighboring areas (Zarza et al., 2019; Zarza et al., 2024).

On the other hand, towards the end of the first millennium AD, various human occupations begin to be recorded south of the confluence of the Paraguay and Paraná rivers. These occupations are related to the Goya Malabrigo Archaeological Entity, with earlier evidence in the Paraná coastal region of Argentina, linked with an Arawak affiliation (Politis & Bonomo, 2012) and consolidate a regional integration system whose influence extends into the interior of the Chaco through the ancient spills of the Bermejo River (Lamenza et al., 2018). These societies exhibit greater variability in settlement patterns, linking levees, bluffs, and lagoon edges. To date, in the Chaco, subsistence shows a hunter-gatherer-fisher economy (Lamenza & Santini, 2013; del Papa & Lamenza, 2019).

In summary, since the settlement of the region, and throughout the entire occupational sequence, there has been positive feedback with neighboring societies that have alternated migration and expansion strategies during periods of socio-environmental tension. In the archaeological record, we identify a common pattern for the entire region with variations in

materiality, subsistence strategies, and settlement patterns due to historical and geopolitical regional changes. We understand the region as a containment area of a sensitive and more critical periphery to these variations.

3.1 Paraguay_Pantanal_ParaguayRiver

Puerto 14 de Mayo: 31-590 calCE

●**I12949** (1EC)**:** 31-590 calCE (1760±120 BP, LP-2897)

Puerto 14 de Mayo archaeological site (20°19'48''S, 58°5'24'W) is a shell midden located in the Alto Paraguay department in the Paraguay Republic and is one of the few sites reported from Northern Chaco. It was excavated in 1956 by Branislava Susnik (Susnik, 1959). Several individuals were buried at the site. One adult from which one sample was included in this study (I12949). The poor preservation did not allow the morphological sex determination. The radiocarbon date made on the adult individual gave an age of 1760±120 BP (LP 2897) (Lamenza et al., 2015). The artifacts from the site include corrugated ceramic fragments, necklace beads, and lithic instruments. Based on the ceramic style, the settlement pattern and use of space, and the exploited resources, the site was included in the Archaeological entity named Pantanal-Chaco, also registered in Argentinian Chaco (Lamenza, 2013; Lamenza et al., 2019). The material from this site is sheltered in the Museo Etnográfico "Andrés Barbero", Asunción, Paraguay.

3.2 Argentina_GranChaco_DryChaco

El Quebracho: 516 calBCE - 411 calCE

●**I12722** (13F): 122-411 calCE (1810±60 BP, LP-3055)
●**I12714** (10F): 516 calBCE - 64 calCE (2220±110 BP, LP-3014)

The site El Quebracho is a pre-Hispanic cemetery located in the homonymous locality of Ramón Lista Department in the province of Formosa (23°20'24''S, 61°52'12''W). It was found by chance during the building of a school in the locality, and the local community allowed and promoted the archaeological excavation, including wichi leaders (Calandra et al., 2012). During the excavation, 14 individuals were exhumed. They consist of primary burials in a genuflex position, most of them (ten individuals) are simple inhumations, while four individuals were buried together in a multiple burial. Morphological sex determination allowed the identification of seven males (six adults and one sub-adult), and three females (two adults and one sub-adult), while four individuals remained undetermined (three adults and one sub-adult). Some of the individuals were associated with T-shaped lithic axes and shell necklace beads manufactured with Strophocheilus sp. Direct radiocarbon dates gave ages of 2220±110 BP (LP-3014), 1810±60 BP (LP-3055), and 1700±40 BP (LP-2648); making this site the earliest from all the Argentinian

Gran Chaco (Lamenza et al., 2016). This is also an exceptional site because it is a formally delimited inhumation place with reuse, clearly corresponding to a cemetery, with signs of recurrent use of the territory, reduced mobility, and wide interaction networks. Only the samples from two adult individuals, a female (I12714) and a male associated with an axe (I12722) yielded enough DNA and they are included in this study.

3.3 Argentina_GranChaco_HumidChaco

El Cachapé archaeological locality

The archaeological locality El Cachape is in the Primero de Mayo Department in the province of Chaco. It is placed on a riverbank north of the confluence of the Paraná and Paraguay rivers, in the middle of a region of estuaries and ravines. It consists of several mounds of mixed origin resulting from natural and anthropic accretion. It constitutes a strategic location because of its proximity to the marsh resources and the waterway. The ceramic style and the radiocarbon dates allow us to assign this locality to the Pantanal-Chaco archaeological entity (Lamenza, 2013).

El Cachapé V: 250-1050 CE

●**I12951** (3EC): 650-1050 CE
●**19229** (5EC): 250-650 CE

The site El Cachape V (26°53'47.2”S; 59°00'49”W) consists of a mound of mixed origin product of natural and anthropic accretion, located near the Edivigis locality in the shores of Quiá stream. The remains of an adult individual (I12951) were found mixed with animal bones in what seems to be a dump. A radiocarbon dating on *Myocastor coypus* remains associated with this individual yielded an antiquity of 689-1025 calCE (1180±70 BP, LP-1549). Below this individual, an infant of about four years old (I19229) was buried in a secondary modality with the absence of the appendicular skeleton. The bones show anthropic postmortem cutmarks, possibly indicating a ritual treatment of the body.

El Cachapé IVB: 200-650 CE

●**I36284** (8EC)**:** 200-650 CE

The site El Cachape IVB (26°52'12''S, 58°57'W) consists of a mound of mixed origin product of natural and anthropic accretion. It is a multicomponent site with two diachronic occupations (204-637 calCE, 1680±100 BP, LP-1734; associated *Blastocerus dichotomus*), and 820±70 BP (1050-1389 calCE, LP-2506; associated fauna). One sample (I36284) from the earliest component was included in this study.

<u>El Chancho:</u> 1650-1922 calCE

●**I36281** (4EC): 1650-1922 calCE (210±40 BP, LP-2524)

The El Chancho site is in San Fernando Department, Chaco Province, near the city of Resistencia, on a fluvial levee covering approximately 780 m² (27°36' S, 58°58'48''W). This site is a shell midden with a thickness of 20 cm and contains an abundance of archaeological remains within its matrix. These remains include faunal materials (deer and fish) and ceramics (primarily plain and painted, with simple forms and varying sizes). The ceramic materials show similarities to the Paraná Component of the Goya-Malabrigo Archaeological Entity (Lamenza, 2013; Lamenza et al., 2018) and Guaycurú-affiliated groups, such as the pottery made by the Mocoví people in the 18th century, which was painted red and polished with a stone (Paucke, 1942-1944). A secondary burial of an adult in soil was also found (Mendez et al., 2001) with an age obtained by radiocarbon dating of 210±40 BP (LP-2524; Lamenza, 2013).

SI Section 4. PAMPAS REGION (ARGENTINA)

Written by Gustavo G. Politis, Mariano Bonomo, Mariela Edith Gonzalez, Pablo G. Messineo, Verónica Aldazabal, Clara Scabuzzo, Romina Frontini, Cristina Bayón, Rodrigo Vecchi, Josefina M. B. Motti, and Rodrigo Nores

The early evidence of human occupation in Pampas occurred at the end of the Pleistocene, and shows a generalized technology without any model of lithic projectile points and is associated with hunting extinct megamammals. This first phase, dated between ~14,000 and 12,900 cal BP, is documented in Arroyo Seco 2 (the lower Component at ~ 14,060 and 13,000 cal BP). These levels show the exploitation of a few taxa: guanaco, the two species of American horse (*Equus* and *Hippidion)*, and giant ground sloth (Politis et al., 2016). Notably, the earlier occupations do not differ much from those that follow, and their chronological precedence is minimal

The first technological change occurred at ~ 12,800 cal BP associated with Fishtail projectile points. Faunal exploitation continued to be restricted to several species: guanaco, *Hippidion, Equus*, *Megatherium, Doedicurus*, and *Hemiauchenia*. Prates and Perez (2021) recently proposed a substantial spatial and temporal relationship between the Fishtail point, which was directly associated with large mammal hunting, and the density and distribution of Pleistocene megafauna. On this basis, they put forward that human predatory behavior was the main factor driving the megafaunal decline in South America at the onset of the Fishtail points expansion, after 12,800 cal BP. There are two nodes in Pampa (including the *Campos*), which suggest areas of more intense occupation during the post ~ 12,900 cal BP phase: the Lower Uruguay and Negro rivers in Uruguay and Tandilia and the Interserrana area. The earliest foragers would travel faster

between concentrated resources, such as patches of megaherbivores and other food supplies or high-quality raw materials, and would slow down when reaching similarly attractive patches.

During the early Holocene times, environmental conditions changed in Pampas. All megamammals became extinct - the Holocene survival of *Doedicurus* is under debate (Messineo et al., 2021) while only a few smaller Pleistocene species, *Eutatus seguini,* and *Dusicyon avus* survived until the Holocene. Guanaco became the main prey in the region all along the Holocene and probably was the base of subsistence. The Summed Calibrated Probability Density (SCPD) curves of radiocarbon dates (see Figure 7.1 in Politis & Borrero, 2024) showed a valley at the end of the Early Holocene and, at ~ 9,000 cal BP, the onset of the curve might be related to the proposed arrival of new people. Shortly after, at 8,700 cal BP, a new type of projectile point, medium to large un-stemmed lanceolate or long triangular, appeared. This type was found nailed in the five older human burials of Arroyo Seco 2 and marked an unprecedented violent situation in the whole region. Although it is tempting to correlate the new gene flow at that time (see Posth et al., 2018) with the lanceolate/long triangular projectile point type and the novel mayhem, limited evidence still prevents it. For the mid-Holocene, there is a consensus about warmer than today's climate prevailing conditions for most of the period in eastern Pampas (*Hypsithermal*), probably with alternating wetter-drier periods for this interval (Kruck et al., 2011).

Barrientos (2009; Barrientos & Perez, 2005) proposed that a population replacement took place in the southeastern Pampas and probably that a new biological population entered in these areas sometime between 6,800 to 5,800 cal BP, after the "emigration or local extinction" of the local population. A roughly synchronic hiatus in the human signal was also proposed for the western Pampas (Berón, 2004) and southern Cuyo (Neme & Gil, 2009). In line with this model, Barrientos and Masse (2014) enlarged the geographical scale of analysis and identified a low archaeological signal in the mid-Holocene and especially a very low signal between 7,500 and 4,400 cal BP in the calibrated data sequence from Central Argentina (in which they included southern Pampas). These authors also saw a null signal in a sequence of radiocarbon dates-between 4,900-4,400 cal BP and suggested a link with the potential effect on the human population and biomass that the Rio Cuarto Cosmic event would have generated. Although some of these hypotheses are provocative, the cluster of archaeological and ancient DNA evidence from the Middle-Holocene in the Pampas does not support and extinction/migration and subsequent population replacement in Pampas. In fact, there is a concentration of radiocarbon dates in these areas within and close to the 6,800-5,700 cal BP interval, where the putative gap in the radiocarbon date has placed the hiatus in the radiocarbon dates sequence. Barrientos' model was also based on changes in craniofacial morphology and temporal variation in cranial vault deformation. However, the morphological studies were based exclusively on the analyses of the facial region of seven mid-Holocene individuals of AS2, compared with several Late Holocene samples from different sites and collections (Barrientos & Perez, 2005). The small sample from the Middle Holocene does not allow robust conclusions, and Perez and Monteiro (2009) have shown that large divergence in craniofacial features can arise in a short time interval and can be related to modifications derived from changes in diet and climate. Barrientos and Masse (2014)

did not fully explore other alternative explanations such as micro-evolutionary processes or changes in the original population due to genetic flux with neighboring populations.

One important innovation that emerged in the mid-Holocene is persistence, liminal places in the landscape. It is associated with some kind of sacrality in specific spots. The primary evidence of this is the human burials of Arroyo Seco 2. The inhumation of 51 human skeletons in 39 graves in a restricted space, dated between 8,700 and 5,100 cal BP implied the continuity of a burial place for about 3,600 years. It also means continuity in a sacred practice, loaded with strong symbolic connotations reflected in rich funerary goods and the frequent presence of red ochre around the bodies. However, the marked difference in the burial paraphernalia also indicates the existence of personal status, individual hierarchy, or spiritual power. Other burials with funerary goods in the region (Cerro de los Viejos 2, Necochea, and Arroyo del Moro) support this hypothesis. The early presence of secondary burials would result from affirming ancestry in the territory, which seems to have originated in this period. At the onset of the Middle Holocene, Pampean hunter-gatherers exploited marine resources and occupied the Atlantic coast. This adaptive pattern would have occurred earlier in the Holocene, but the rise in sea level might have destroyed this archaeological evidence, as we discussed before.

During the Late Holocene Pampas foragers experienced significant reconfigurations in economic, social, and demographic structures. In this period, the archaeological visibility of the Pampas increased significantly, a fact that also suggests a rise in the regional population density. The number of sites and their wider distribution, including areas previously unoccupied or poorly inhabited, support that during the Late Holocene, there was a demographic increase in the Pampas after ~3,500 calBP. Moreover, some ecosystems, such as the lower section of the Salado Depression, show a human signal for the first time, as a result of changes in sea level that previously prevented settlement. In the South Salado River basin, technical and stylistic similarities are observed in the ceramics with the Littoral-Paraná region; in the lithic material with the Southern Pampas, from where the raw materials also come; and some isolated elements that are interpreted as exchanges or contacts with the Patagonian region (Aldazabal, 2004).

Also during the Late Holocene some technological innovations took place in the region, producing economic and social advantages: the bow and arrow weapon system and pottery. It is unclear when Pampas hunter-gatherers adopted the bow and arrow, but the earliest dated appearance of small triangular projectile points, interpreted as arrowheads, are in Calera site, associated with an age of 3,100 cal BP. However, this date is the only one that suggests such an early chronology and therefore should be taken with caution. Roughly at the same time, pottery was recorded for the first time in the region at Zanjón Seco 2 (Politis et al., 2001). Both innovations coincide with a peak in the SCPD curve, which would suggest that they might bring some adaptive benefits that result in a population increase. The bow and arrow system was such a technological step ahead that it could have increased meat consumption and population growth rates. It may also have caused a change from communal/collective hunting to smaller groups or individual hunting (Marlowe, 2005). Pottery is a means of food processing and displaying social symbols. Ceramic vessels created unprecedented cooking and storage techniques and facilities, generating regional cooking practices. Storage facilities allowed supply

accumulation, delayed consumption, increased predictability, and provided food reserves. Stored plant or animal foodstuffs could also offer opportunities to dispose of supplementary resources for rituals or feasts. Since its first appearance Pampas pottery was decorated with different varieties of incisions (mostly drag and jab and linear incisions), providing a medium for symbolic expressions and identity at various levels (individual, social, ethnic, etc.). In this context, the design element defined as "small flags" is ubiquitous in the region and would have functioned as a diacritic for social interaction on a broad spatial scale (Di Prado, 2018).

In the Late Holocene economy was diversified and had differences among the areas. Although guanaco was the main prey, other mammals were incorporated in different proportions depending the area: Pampas deer, Rhea, armadillos, rodents etc. There is also evidence of the exploitation of fruits form the Espinal Forest, especially in the western sector

The Late Holocene innovations, along with other social behaviors (territorial circumscription, changes in mortuary practices, wide exchange networks, etc.) as well as higher demography, resulted in the process of economic intensification along with an increase in complexity in the Pampas region in this period. When European arrived into the region, at the beginning of the XVI Century the region was populated by nomadic hunter-gatherers named Querandíes.

4.1 Argentina_Pampas_CentralPampeanDunefields

Laguna de los Pampas: 8290-7812 calBCE

●**I23842** (LLP.S2.E1.I1): 8290-7812 calBCE (8971±77, AA-90127)

The Laguna de los Pampas archaeological locality is placed in the Central Pampean Dunefields area, one of the aeolian unit systems from the eastern center of Argentina. This locality is situated in the current margins of shallow lakes, with recurrent hunter-gatherer occupations through the Early and Middle Holocene (Politis et al., 2012; Messineo et al., 2018, 2019; Gonzalez et al., 2023). Nine burials were recovered in two sectors of a lake's beach, four from the Sector 1 (35°19'42''S and 61°31'50''W) and five from the Sector 2 (35°19'56''S and 61°31'53''W) due to re-exposition by water erosion. Moreover, isolated human bone remains corresponding to other nine individuals were found from both sectors. Eleven human individuals (eighth from the burials and three from isolated mandibles) were dated to the Early and Middle Holocene. Burial 1 includes a multiple primary burial composed of two individuals: one adult female dating from 8971±77 BP (LLP.S2.E1, AA-90127) and one sub-adult of 2-4 years old dating from 8835±83 BP (L.LLP.S2.43, AA-93221). One sample (I23842) from the adult individual was included in this study. Burials 2, 6 and 7 are simple primary and they dated back to 5688±36 BP (L.LLP.S2.1062, AA-108848), 7575±30 BP (L.LP.S2.E6.5, PSUAMS#13968), and 5760±25 BP (L.LP.S2.E7.3, PSUAMS#12067), respectively. Burial 3 is multiple primary dated to 5819±24 BP (L.LLP.S1.E3, MAMS-24770). Bone remains of burial 4 and 5 were found on the surface and the burial mode was not determined. They dated back to 5924±40 BP (L.LLP.S2.E4, AA-106730) and 7089±37 BP (L.LLP.S1.2706, AA-110832), respectively . Burials 8 and 9 are simple primary but

they still do not have radiocarbon dates. Three isolated mandibles were dated in 7653±39 BP (L.LLP.S1.32, AA-110831), 6580±30 BP (L.LP.S2.1695, PSUAMS#12068) and 6543±37 BP (L.LLP.S1.5134, AA-110829) (Messineo et al., 2019; Gonzalez et al., 2023; Scheifler et al., 2024).

Isotopic analyses ($\delta 13_{ccoll}$ and $\delta 15_{N}$) from nine individuals of Laguna de los Pampas were performed and these individuals obtained nutrients primarily from terrestrial sources throughout the Holocene. The values for the two oldest individuals from the Early Holocene (Burial 1) show low variability and suggest that they exploited mostly high trophic level species, such as armadillos, foxes and skunks. This could be associated with generalist and opportunistic subsistence strategies during the human colonization of the area. The dispersion of the values for the Middle Holocene individuals, including the Burial 6 dated in the last part of the Early Holocene, suggests that the guanaco was the main source of proteins. The low interindividual human variation would be linked to differential consumption in low proportions of smaller prey exploited during this period (Scheifler et al., 2024). The identification of an oblique tabular modification in an individual from the Middle Holocene (Burial 4, ~6000 BP) represents novel data, since it would be the earliest record corresponding to this type of cranial deformation for the Pampas region (Gonzalez et al., 2023).

Stratigraphic excavations have been performed in Laguna de Los Pampas S2E site and abundant lithic materials and faunal bone remains have been found with Middle Holocene chronology (between 7024±45 and 5815±40 BP). The lithic materials are characterized by a predominance of orthoquartzite and chert, which outcrops in the Tandilia Hill Range, 200 km far from the site. Other lithic raw materials, such as granite, silicified dolomite, silex, micaceous schist, and obsidian, were present in low frequencies. Some of these rocks come from diverse sectors of the Pampas and extra-regional areas. A high diversity of lithic tools was found, including side scrapers, end-scrapers, knives, multipurpose tools, triangular projectile points, etc. (Messineo et al., 2018, 2019). In addition, a standardized bone technology was also identified in Laguna de los Pampas locality and centered in the production of beveled tools and blunted points made on guanaco tibiae during the Middle Holocene (Álvarez, 2014; Messineo et al., 2019).

The analysis of the diverse archaeological materials indicates that the Laguna de los Pampas locality was occupied during Early and Middle Holocene times. It might represent a succession of residential camps and human burials in the border of the shallow lakes by hunter-gatherers focused on the exploitation of guanaco (*Lama guanicoe*), although Pampas deer, armadillo, foxes and Greater rhea were also represented. In addition, complete eggs of greater rhea with a small perforation at the minor pole were found. They were possibly employed as flasks for storing water by hunter-gatherer groups in environments with shortage of sources of fresh water (Messineo et al., 2019).

4.2 Argentina_Pampas_Southern

El Guanaco 1: 800-150 BCE

●**I12371** (69JM): 391-190 calBCE (2,280±30 BP, BETA-137745)

●**I12370** (68JM): 763-400 calBCE (2,470±60 BP, BETA-128180)
●**I12372** (70JM): 763-396 calBCE (2,460±60 BP, PTA-8520)
●**I12373** (71JM): 800-150 BCE
●**I12374** (72JM): 800-150 BCE

El Guanaco archaeological locality (38°45´S; 59°39´W) is located near the El Lucero shallow lake, in the Interserrana area (Buenos Aires province). An important surface collection was recovered and two sites, El Guanaco 1 and El Guanaco 2 were identified; they are 500 m distant from one another (Bayón et al., 2004; Bayón et al., 2006; Flegenheimer et al., 2010). The lithic collection is composed by raw materials and tools from diverse rocks, including Sierras Bayas Orthoquarzite Group, basandesite and phtanite (chert). Considering that the location of the Locality lacks rocks, all the lithic materials (weighted 140 kg) were intentionally transported. The provenance of rocks include Atlantic littoral, Tandilia range and Ventana range (Bayón et al., 2004). Based on this information, the locality was interpreted as a preferential landscape intentionally supplied with rocks to be recurrently occupied during Late Holocene (Bayón & Flegenheimer, 1998; Bayón et al., 2006).

El Guanaco 1 site yielded occupations from the Early Holocene to Late Holocene. Lower levels, dated to the Early Holocene, yielded lithic tools and faunal remains including extinct megafauna without anthropic evidence. These levels were partially incercepted by the Late Holocene human burial pits (Bayón et al., 2004). Seven individuals were recovered in primary burial, secondary burial and isolated human remains. The assemblage is composed of 4 subadults (3 juvenile and one infant), 2 adults and an indeterminate. Regarding sex, they are 3 males/probably males; 1 females/probably female and 3 undetermined. Human remains show regular preservational conditions, being roots marks the most represented taphonomic effect (Mazzia et al., 2004).

Three radiocarbon dates on human bones were obtained. Individual 1 (68JM; I12370) yielded 2470±60 BP (BETA-128180; δ13CCOL= -18.4‰); Individual 2 (69JM; I12371) yielded 2280±30 BP (BETA-137745; δ13CCOL= -18.5‰) and Individual 3 (70 JM; I12372) yielded 2.460±60 BP (PTA-8520, δ13CCOL= -17.7‰; δ15N= 10.5‰). Stable isotopes analyses on bone organic fraction evidenced that the human diet was based on terrestrial prey (Flegenheimer et al., 2002). This is concordant with the predominance of guanaco (*Lama guanicoe*) and vizcacha (*Lagostomus maximus*) remains in the archaeofaunistic assemblage (Frontini, 2013). Finally, the mitogenomes of 5 individuals were analyzed in a previous work (Motti et al., 2023).

Túmulo de Malacara: 1000-500 BCE

●**I34660** (MLP-DA-6420): 802-547 cal BCE (2585±20 BP, PSUAM-S12926/LI0340)
●**I34667** (MLP-DA-6417a): 806-547 cal BCE (2595±25 BP, PSUAMS-13095/LI0343)
●**I34661** (MLP-DA-6418): 897-777 cal BCE (2680±20 BP, PSUAMS-12925/LI0339)
●**I34668** (MLP-DA-6419): 919-782 cal BCE (2710±40 BP, AA-24049)
●**I42078** (MLP-DA-6429): 1000-500 BCE
●**I42079** (MLP-DA-6433): 1000-500 BCE

Túmulo de Malacara was located in 1913 by Torres and Ameghino (1913a, 1913b), who, due to its morphology and the discovery of human burials, classified the site as a burial mound. It is located near the mouth of the La Malacara stream in the Pampean coastal region (38°26'46.77"S, 58°14'58.59"O, Buenos Aires Province, Lobería District). More detailed information about the burials found at the site was provided by Vignati (1960). Recently, the materials from this site deposited at the MLP were reanalyzed (Bonomo et al., 2024), including 1669 human bone specimens, 57 shell bead ornaments, and 104 lithic artifacts (scrapers, abrasives, bipolar cores, and debitage).

Regarding human remains, 266 elements were analyzed, allowing for the estimation of 13 individuals from maxillae. Based on the comprehensive analysis of the osteological collection and historical documentation, the individuals were distributed among eight burials: four primary burials, one probable multiple primary burial, and three secondary burials. The six specimens studied include a left upper second molar (MLP-DA-6419, I34668) from a male individual estimated to be 20-30 years old, with a radiocarbon date of 2710±40 BP (AA-24049) (Politis et al., 2011) and recent isotopic data: $\delta^{13}C_{COL}$= -17‰, $\delta^{13}C_{AP}$= -6.6‰, and $\delta^{15}N$= 12.4‰ (LIECA-1366-1695). A right petrous bone (MLP-DA-6418, I34661) from a male individual was dated to 2680±20 BP (PSUAMS12925/LI0339) with isotopic data: $\delta^{13}C_{COL}$= -17.5‰, $\delta^{13}C_{AP}$= -11‰, and $\delta^{15}N$= 12.9‰ (PSUAMS12925, LIECA-1365). The same catalog number (MLP-DA-6418) also includes a left tibia with a pathological condition showing increased diameter in the medial diaphysis, mainly towards the anterolateral sector, macroporosity, and a groove on the medial face possibly indicating a vascular trace. A third upper molar (MLP-DA-6417a, I34667) corresponds to a male individual in a primary genupectoral position and lying on its side, covered by a block of *tosca* stone. A radiocarbon date of 2595±25 BP (PSUAMS13095/LI0343) and isotopic data: $\delta^{13}C_{COL}$= -17.3‰, $\delta^{13}C_{AP}$= -12‰, and $\delta^{15}N$= 14.7‰ (LIECA-1363-1692) were obtained. Another analyzed sample (MLP-DA-6420, I34660) is the left fibula of an adolescent/young adult, dated to 2585±20 BP (PSUAMS12926/LI0340) with isotopic data: $\delta^{13}C_{COL}$= -21.5‰, $\delta^{13}C_{AP}$= -11.2‰, and $\delta^{15}N$= 12.7‰ (LIECA-1367-1696). Additionally, a left petrous bone (MLP-DA-6429, I42078) was analyzed, with the following isotopic data: $\delta^{13}C_{COL}$= -23.7‰ and $\delta^{15}N$= 13.1‰ (LIECA-2039-2239). Finally, another petrous bone (MLP-DA-6433, I42079) was analyzed, with the following isotopic data: $\delta^{13}C_{COL}$= -19.7‰ and $\delta^{15}N$= 12.7‰ (LIECA-2041-2241).

4.3 Argentina_Pampas_SouthSaladoRiver

<u>**Mar Chiquita 1:**</u> 1000-1300 CE

●**I12368** (66JM): 1045-1270 calCE (910±50 BP, LP-3256)
●**I12369** (67JM): 1000-1300 CE

The Mar Chiquita 1 site (37°42'S 57°23'W) is located in the southeast of the province of Buenos Aires, Argentina in the homonymous District and adjacent to the Atlantic coast. It is a

sector that was considered a recent landscape, formed about 3,000 to 2,200 years ago by sedimentary accumulation produced by littoral drift after the marine ingressions occurred until the middle of the Holocene (Violante et al., 2001). An increase in coastal erosion in recent years (Merlotto & Bértola, 2009) resulted in the exposure of archaeological remains, such as the human remains studied here that were found by chance. The site includes only human burials. The analysis of these remains allowed determining that they belonged to three individuals: one adult male (I12368), dated in 787 years calBP, one sub-adult female (I12369) and another indeterminate sub-adult that was not analyzed in this work (Aldazabal et al., 2020). Along with the skeletons, three artifacts were found, two made on coastal pebbles and another on guanaco (*Lama guanicoe*) bone. The mitogenomes of the two adult individuals were analyzed in a previous work (Motti et al., 2023).

Laguna La Colorada: 1505-1128 calBCE

●**I34672** (VA1): 1505-1128 calBCE (3140±70 BP, LP-807)

The Laguna La Colorada site (36°28'48.0"S 58°37'12.0"W) is located in the South Salado river basin in the Buenos Aires province, Argentina. The site is situated on the northern shore of the La Colorada lagoon in the department of Rauch. Based on the archaeological evidence, the primary prey species hunted were guanaco (*Lama guanicoe*), followed by deer (*Ozotoceros bezoarticus*). The artifact assemblage consists mainly of lithic tools made from flakes, with bifacial retouching and a predominance of scrapers. Grinding instruments were also recovered. In terms of raw material, quartzite predominates. Some pottery fragments were also found, though they are scarce. Human remains were discovered separately from the settlement area, exposed by the collapse of a natural ravine. No tools or goods were found in association with the skeletons. The remains belong to two individuals, one of whom (I34672), identified as female through morphological characteristics shows evidence of cranial artificial deformation (Aldazabal & Cáceres, 1997). A radiocarbon date from a long bone yielded an age of 3140±70 BP (LP-807). Stable isotope values indicate a primarily terrestrial diet (δ15N +9.2‰, δ13C(col) -19.5‰) (Murgo, 2002; Murgo & Aldazabal, 2001).

SI Section 5. PARANÁ RIVER REGION (ARGENTINA)

Written by Silvia E. Cornero, Mariano Bonomo, Gustavo G. Politis, Josefina M. B. Motti, and Rodrigo Nores

Over the past two decades, there has been a significant increase in archaeological data that has expanded and refined our understanding of past societies in the Lower and Middle Paraná floodplains. This region, located in the lowlands of the Southern Cone, was inhabited by ranked societies with a relatively low degree of social complexity, characterized by a mixed economy that lasted for at least 2,400 BP, according to dating from the northern sector of the Middle

Paraná River, extending up to European contact. The archaeological expression of these societies is identified as the Goya-Malabrigo archaeological entity (Ceruti, 2003; Politis & Bonomo, 2012, 2018), while the ethnographic name used to refer to these people during the 16th and 17th centuries was "Chana-Timbú." Under this generic term, several subgroups were recognized, including the Chaná, Chaná-mbeguá, Timbú, Caracaráes, Corondas, and Mocoretás, among others.

The pre-Hispanic peoples of the Lower and Middle Paraná based their subsistence on wild resources, supplemented by small-scale horticulture. Archaeobotanical analyses have revealed the presence of maize, beans, and squash (e.g., Bonomo et al., 2011a; Cornero & Rangone, 2015; Colobig & Ottalagano, 2016; Colobig et al., 2015). Their diet also included an abundance of fish, such as tararira (*Hoplias malabaricus*), sábalo (*Prochilodus platensis*), boga (*Leporinus obtusidens*), chanchita (*Cichlasoma facetum*), and common armado (*Pterodoras granulosus*), along with other species. In addition to gathering wild plants, their diet was supplemented by hunting marsh deer (*Blastocerus dichotomus*), capybara (*Hydrochoerus hydrochaeris*), coypu (*Myocastor coypus*), and other animals, as shown by archaeofaunal research (Cornero et al., 2007; Pérez Jimeno, 2007; Bonomo et al., 2011b; Politis et al., 2011; Barboza, 2014; Sartori & Galligani, 2014; Piccoli, 2015; Bastourre & Azpelicueta, 2020). Within the fauna associated with these indigenous groups, the presence of the domestic dog (*Canis familiaris*) stands out (Castro et al., 2020). The use of dugout canoes, carved from hardwoods as a means of navigation for fishing, transporting goods, moving people, and engaging in warfare, has been documented for the region (Bonomo & Ramos, 2023). These canoes were crucial for the occupation of the insular environments of the Paraná Delta.

The Goya-Malabrigo settlements were located in the floodplains, in naturally elevated areas such as ridges and dunes, but they also constructed mounds, locally referred to as *cerros* or *cerritos* and identified as *túmulos* in early archaeological studies of the region (Torres, 1911). Current research confirms that the indigenous populations of the Lower and Middle Paraná River (Bonomo et al., 2011b; Bonomo & Politis, 2020; Politis et al., 2011; Castiñeira et al., 2013, 2014) and the Lower Uruguay River (Castro 2016) not only occupied natural high ground but also modified the landscape by constructing mounds. These studies have demonstrated that many settlements were mounds formed either partially or entirely through human activity, resulting from both the accumulation of materials due to repeated occupation and the intentional elevation of the ground using clays with plastic properties and other cohesive materials such as pottery sherds. These mounds had three primary functions: as settlements for small villages, as burial sites for the dead, and as areas for crop cultivation, likely through household horticulture. The burial practices, which included both primary and secondary burials and assemblages of skeletal remains (Gaspary, 1950; Cornero, 2009; Ceruti, 2013; Scabuzzo et al., 2015), likely turned these spaces dedicated to ancestors into places where collective memory was created and reinforced. Although many of the earth mounds in the Lower Paraná and Lower Uruguay rivers have been attributed to the Goya-Malabrigo archaeological entity, other mounds have been identified in sites that are not associated with this entity (e.g., Túmulo II del Brazo Largo, Túmulo I and II del Paraná Guazú in the Lower Paraná Delta), some of which have been assigned to the Ibicuí or Ibicueña facies (Caggiano, 1984; Serrano, 1950).

The characteristic *campanas* (bells) made of pottery were iconic elements of the Goya-Malabrigo archaeological entity (Ceruti, 2003; Di Prado, 2015; Gaspary, 1950; Politis & Bonomo, 2012; Schmitz et al., 1972; Serrano, 1950). These bells are cylindrical or truncated-cone-shaped pieces, open at the bottom, often with a zoomorphic appendage at the top. Other artifacts found include vessels with three inner compartments, spindle-whorls, and bi-dimensional and three-dimensional appendages representing birds, mammals, reptiles, and mollusks. Notable appendages include the heads of birds with colorful, bright plumage (psittacines), birds of prey (falconiforms), and dangerous solitary animals (e.g., snakes and jaguars) (Bonomo et al., 2021). The predominant decorative technique was drag-and-jab, used to create straight and stepped lines, zigzags, and geometric patterns. The deep symbolic meaning of the Goya-Malabrigo iconography has been discussed from various perspectives (Bastourre, 2022; Bonomo et al., 2021; Cornero, 2018; Ottalagano, 2013).

Lithic artifacts are relatively rare, with raw materials sourced from distant geological formations (more than 170 km). The scarcity of stone tools in the region promoted the use of bone technology. Various bone artifacts have been recovered, including harpoons, grooved, semi-grooved, and flat tips with or without peduncles, as well as pendants (Pérez Jimeno, 2007; Buc, 2010). The Lower and Middle Paraná people had trade and exchange relations with distant societies, as evidenced by the presence of foreign artifacts and ethnohistorical records (Ceruti, 2013; Cornero & Green, 2022; Cornero & Tissera, 2024). These people appear to have been integrated into both local systems of intragroup interaction and broader supra-regional exchange networks (Apolinaire & Bastourre, 2016; Bonomo et al., 2017; Politis & Tissera, 2023), which facilitated the arrival of personal adornments made from metal and stone, along with other exotic goods. In the Paraná region, raw materials and artifacts originating from Northwest Argentina and the Córdoba Hills have been found, including externally manufactured metal pieces, particularly copper (Villegas Basavilbaso, 1938; Ceruti, 2018; Bonomo et al., 2017 and others), as well as malachite, turquoise, and other rocks (Crouzeilles, 1936, 1939; Frenguelli, 1948; Ceruti, 2018; Bonofiglio, 2004; Bonomo et al., 2011), flanged axes and bolas (Ceruti, 2018: 165, 169), and human remains (Canova et al., 2020). Connections between the interfluvial regions of the Salado and Dulce Rivers in the Santiagueña plains and the Paraná have also been demonstrated by several authors (Angiorama et al., 2017; Bonomo et al., 2011; Cornero, 2021; Cornero & Green, 2024; Cornero & Tissera, 2024; Crouzeilles, 1939; Graneros & Cornero, 2022; Politis & Tissera, 2023; Serrano, 1945; among others) through various lines of archaeological and ethnohistorical evidence. Regarding ceramics, Crouzeilles (1939) was the first to establish symbolic links between the Santiagueña plateau and the Paraná coast in Santa Fe. Ceramic analysis has revealed direct correlations and suggested possible Paranean influences in the Santiagueña Mesopotamia (Von Hauenschild, 1949) and the Santiagueño Impenetrable (Angiorama et al., 2017). In the Santiagueña Mesopotamia, exotic items such as *Urosalpinx rushi* shells—originating from the Uruguayan coast and used ornamentally for making necklaces—have been discovered. These worked gastropods have been found in Córdoba, Mendoza, parts of Santiago del Estero, and at the Ombú de Basualdo site on the lower Paraná coast of Santa Fe (Doello Jurado, 1940; Martínez Soler, 1964).

Authors such as Politis and Tissera (2023) and Cornero and Tissera (2024) have demonstrated long-distance exchange circuits based on mobility studies and ceramic records. Ethnohistorical sources also report that in 1561, the governor of Santiago del Estero, Gregorio de Castañeda, interviewed Jofré, a Coronda Indigenous man “who was a prominent native of the Río de la Plata,” who had arrived from the Paraná and provided information about the travel routes. This indicates that Jofré moved with familiarity across more than 600 km from the Coronda River—connecting the Paraná to Santiago del Estero—and was aware of the journey's duration and able to communicate with Indigenous groups speaking the same language or a lingua franca (Cornero & Green, 2022).

Finally, Guaraní occupations have been identified in the Lower Paraná Delta from 600 BP based on a series of diagnostic traits, primarily their pottery, characterized by repeated forms, decorations, and functions (Bonomo et al., 2015). There are six main types of vessels, mostly for domestic use: yapepó (pot), ñaetá (casserole), ñaʼẽ, ñaʼẽmbé, tembiyru or tembeʼyʼu (plate), kambuchi (jug), kambuchi kaguava (cup), and ñamypyũv (roaster) (Noelli et al., 2018). The Guaraní economy was based on the cultivation of crops and studies of Guaraní pottery from the Paraná and Uruguay rivers have confirmed this by the presence of maize, squash, and beans inside these vessels (Costa Angrizani et al., 2021). Their diet also included fishing, hunting, and gathering wild plants (such as carob). The Guaraní introduced the practice of burying the dead in urns, often near homes or village edges. These practices have been observed in the Lower Paraná Delta, at sites like Arroyo Malo, Arroyo Fredes, and Arroyo La Glorieta, where funerary urns containing the bones of children and/or adults were recovered (Lothrop, 1932; Mazza et al., 2016; Ramos van Raap & Bonomo, 2016).

5.1 Argentina_ParanaRiver_MiddleParanaSaladoRivers

Isla del Indio/Paraná Miní I: 1150-1950 CE

●**I42333** (IdI-1, SEC3): 1150-1950 CE

The Isla del Indio site (29°1'50.00"S; 59°20'0.00"W) is located on the left bank of the Paraná Miní River, 300 m from the Ocampito Creek mouth, near Reconquista (Gral. Obligado Department, Santa Fe Province). Originally excavated by Palavecino (1948), Rex González and the Museo de La Plata team (Schmitz et al., 1972), and later by the Museo de Reconquista team led by D. Ruggeroni between 1966 and 1975 (Ruggeroni, 1975). The site consists of a semi-buried mound with extensive ceramics, featuring biomodeled appendages, temper pottery (1-3 mm), domestic vessels, bowls with spouts, bone tools such as harpoons and awls, and perforated antlers. Human burials were exhumed, with a primary burial, IdI-1, sampled for this study. Dating was conducted by Rex González and later published by Ceruti (2003). Calibration of direct dates from the same site (385±145/500±130/640±115 BP, Geochron Lab., no lab codes available) yielded a time range for the site between 1184-1920 calCE.

La Lechuza: 143-1300 CE

●**I42335** (LZA36, SEC6): 1045-1280 calCE (890±60 BP, LP-3306)
●**I30896** (LZA23, SEC4): 1000-1300 CE
●**I42408** (LZA31, SEC14): 251-574 calCE (1680±60 BP, LP-1725)
●**I30897** (LZA5, SEC5): 143-522 calCE (1750±60 BP, LP-3456)

Located on a 2 m high artificial mound, 14 km west of Alejandra, San Javier Department, Santa Fe Province (29°54'10" S, 59°55'36.9" W). The site, covering 121 excavation grids, contains archaeofaunal evidence, with remains of guanacos, dogs, birds, and fish; selective hunting of otters and deer; abundant temper pottery, ornamented with Diplodon shell beads, and scattered fired earth fragments (Cornero, 1999). A total of 41 burials were identified, with diverse modes and orientations, representing 52 individuals, some with red ochre pigments of ritual origin (Cornero, 2016). The basal level date, based on a dog mandible, was 2413±28 BP (D-AMS 025193). Another date of 1760±60 (LP-853) corresponds to a simple secondary burial of a young adult male. Four burials were included in this study. I42335 was a primary burial of a mature adult female in a prone position; I30896 was a primary burial of an adult female in a supine position; I42408 was an individual funerary bundle, possibly of an adult male, discovered alongside bundle LZA30; and finally, I30897 was a primary burial of an adult male in a supine position.

La Criolla: 750-1250 CE

●**I42407** (SEC13): 750-1250 CE

The La Criolla site (San Justo Department, Santa Fe Province, 30°13.116' S; 60°36.189' W) was excavated by Carlos Echegoy in the 1990s. Located on the shore of La Blanca Lagoon, in an area with subterranean kiln outcrops and scarce surface ceramics and lithics, excavations were also conducted by Ceruti (1988) and later by del Río et al., (2016), yielding a date of 1060±90 (774-1219 calCE, LP-2912). The sample from this study comes from a primary adult female skeleton excavated by Echegoy, currently housed at the La Criolla Museum. The date is not direct but the closest available.

Laguna del Plata: 750-1250 CE

●**I42409** (E1, SEC15): 750-1250 CE

The Laguna del Plata Archaeological Site (LdP) is located in a marsh and semi-permanent lagoon environment on the interfluvial plain between the San Javier and Salado Rivers, near the Laguna del Plata, at the junction of the San Justo, Vera, and San Javier departments, Santa Fe Province (30°8'30.34"S; 60°10'37.70"W). The site is situated on the lagoon's bluff, about 29 m above sea level. Initial surveys and soundings were conducted by Frenguelli, Zapata Gollán, and

later by Ceruti (1988), who obtained three radiocarbon dates: 2000±100 BP (339 calBCE - 326 calCE), 1000±140 BP (772-1296 calCE), and 325±80 BP (1446-1940 calCE) (Laboratory INGEIS). Excavations resumed in recent years (del Río et al., 2021), recovering three human burials exposed along the edge of the bluff. Part of the skeletons were found at the basal level of the bluff and were partially exposed. Nearby, subterranean kilns were identified, some submerged in the lagoon water. Ceramic fragments, both plain and decorated, were recovered. The bone sample included in this study has no direct date.

El Camping: 773-1177 calCE

●**I30899** (CPG-I3, SEC7): 773-1177 (1090±70 BP, LP-3204)

The El Camping (CPG) site (29°54'37.46"S; 59°49'5.49"W) is on an extensive coastal levee on the right bank of the San Javier River, near Alejandra (San Javier Department, central-northern Santa Fe Province), approximately 234 km NE of Santa Fe. Named after its location within the current community campground, the site is exposed on the surface, semi-buried, and highly disturbed by anthropic activity. Human burials with a minimum number of individuals (MNI) of 20 were found, including primary (partial and complete), secondary (multiple funerary bundles), and isolated bone elements. Three dates were obtained: 180±70 BP (LP-1002), 290±70 BP (LP-2139), and 1090±70 BP (LP-3204), the last being a direct date for the sample included in this study, an adult male. Personal ornaments made of *Diplodon* shells were also found.

Arroyo Aguilar: 200 BCE - 400 CE

●**I42332** (AAI-D, SEC2): 200 BCE - 400 CE

The Arroyo Aguilar site (29°14'29.95"S; 59°35'25.81"W) is located on a levee along the bank of the Aguilar Creek, near Reconquista Port, 12 km from the town of Reconquista (General Obligado Department). Excavations began in 1975 under D. Ruggeroni, with the Museo de Reconquista team (M.E. Moreira, C. Echegoy, R. Viola), continuing until 2008. Human burials of both sexes and various ages were recovered, with evidence of ochre pigments in some burials, indicating ritual origins. Abundant ceramics, modeled and engraved with reddish paint; remains of local faunal species and mollusks were also found. Two radiocarbon dates were obtained (Ruggeroni & Gallagher, 2006; Echegoy, 1994), one direct (1830±50 BP, 119-366 calCE, LP-195) for sample SEC17 = AAI-A, assigned to a primary burial of an adult male, supine. The second date (2050+60 BP, 161 calBCE - 123 calCE, LP-512) corresponds to a funerary bundle found in AAII.

5.2 Argentina_ParanaRiver_UpperDelta

Laguna de los Gansos 2: 1322-1451 calCE

●**I12946** (LDLG2-1, 1MB): 1322-1451 calCE (570±43 BP, AA-98851)

The site Laguna de los Gansos 2 (32°29'37.1"S; 60°38'25.7"W) was excavated by Bonomo and Politis between 2012 and 2014 (Bonomo et al., 2016). It is located on a natural levee at the entrance of a corral adjacent to a rural outpost (Province of Entre Ríos, Department of Diamante), where 17 m² were excavated. From this site, 587 ceramic fragments (plain, incised, and with red pigment and zoomorphic appendages) and 461 faunal remains were analyzed, with the most represented mammals being *Myocastor coypus*, *Hydrochoerus hydrochaeris*, and *Blastocerus dichotomus*, along with Actinopterygii and Siluriformes fish and Mollusca.

Human skeletal elements corresponding to two individuals buried primarily and secondarily were recovered, dated between 570 and 590 BP (Bonomo et al., 2016). The specimen analyzed here (LDLG2-1, I12946) is a left first phalanx of a young adult male, between 22 and 26 years old. This incomplete skeleton, found in a dorsal decubitus position, consists of the pelvic girdle with articulated lower limbs and the skull relocated over the pelvis. The remaining skeletal elements were absent. This individual was dated to 570±43 BP (AA-98851), with the following isotopic data: $\delta^{13}C_{COL}$= −19.9‰, $\delta^{13}C_{AP}$= −14.4‰, and $\delta^{15}N$= 10.2‰ (UGAMS-11476) (Bonomo et al., 2017).

<u>Los Tres Cerros 1:</u> 1156-1408 calCE

●**I12947** (LTC1-P1, 2MB): 1291-1408 calCE (657±43 BP, AA-98852)
●**I12948** (LTC1-P3, 3MB): 1156-1286 calCE (849±45 BP, AA-98853)

The archeological site Los Tres Cerros 1 (32°51'17.3''S; 60°33'37.6''W) was excavated by Politis, Bonomo, and Scabuzzo between 2008 and 2022 (Bonomo et al., 2010; Politis et al., 2011; Scabuzzo et al., 2015). It is an earth mound located on Las Moras island, on the left bank of the Almada ditch parallel to the Paranacito stream (Province of Entre Ríos, Department of Victoria). Over 70 m² were excavated, obtaining 23 radiocarbon dates indicating site occupation between 1227 and 500 BP, with periods of stability alternated with episodes of abandonment and reoccupation (Politis et al., 2011, 2019). Throughout this period, populations engaged in hunting, gathering, fishing, and horticulture used the mound to develop domestic activities and bury their dead. The chronology, geographic location, and stylistic and technological characteristics of the ceramics relate these occupations to the Goya-Malabrigo archaeological entity (Politis and Bonomo, 2023). Ceramics dominate the site's artifact assemblage with over 30,000 fragments (zoomorphic appendages, plain, incised, corrugated, and unguiculated sherds) (Di Prado, 2019). At LTC1, *campanas*, a three-compartment vessel, and a spindle whorl were recovered. Lithic artifacts are scarce (25 registered so far), and various bone artifacts (grooved points, harpoon heads, and a pendant) were also recorded. Numerous bones and teeth corresponding to coypu (*Myocastor coypus*) and capybara (*Hidrochoerus hidrochaeris*), along with wild cavy (*Cavia aperea*), pampas fox (*Lycalopex gimocercus*), freshwater clams (*Diplodon* sp.), and infrequent Ampullariids were found. Additionally, fish skulls, spines, and vertebrae (*Hoplias malabaricus*, *Leporinus obtusidens*, *Cichlasoma facetum*, and *Pterodoras granulosus*) were identified.

Archaeobotanical studies identified globular phytoliths of Arecaceae, mameloned and/or corrugated surfaces of *Equisetum* sp., faceted edges of *Canna* sp. (achira), and crosses of Orizeae (family including wild rice). This evidence of wild plant manipulation is complemented by cross-shaped morphotypes related to *Zea mays* (maize) and hollowed-out cucurbitaceous morphotypes.

In addition to this domestic context, human skeletal remains of at least 43 individuals in primary and secondary burials (Scabuzzo et al., 2015; Scabuzzo & Ramos van Raap, 2017) were found. Differential manipulation of skeletal remains at the site reflects elaborate mortuary practices, including primary and secondary burials, cremation, and isolated anatomical parts. Thus, the bodies were subjected to different treatments, following various burial, exhumation, and re-burial trajectories. The specimens analyzed here consist of a right carpal of a teenage female from primary burial 1 (LTC1-P1, I12947), dated to 657±43 BP (AA-98852), with the following isotopic data: $\delta^{13}C_{COL}$= −18.4‰, $\delta^{13}C_{AP}$= −13‰, and $\delta^{15}N$= 11‰ (UGAMS-11475). Additionally, a right first phalanx of an adult female buried face down and covered with abundant red pigment from primary burial 3 (LTC1-P3, I12948) was analyzed. This individual was dated to 849±45 BP (AA-98853), with the following isotopic data: $\delta^{13}C_{COL}$= −20.1‰, $\delta^{13}C_{AP}$= −14.7‰, and $\delta^{15}N$= 12.4‰ (UGAMS-11477) (Bonomo et al., 2017).

<u>Cerro Grande del Paraná Pavón:</u> 1250-1450 calCE

- **I12748** (62-251)**:** 1250-1450 calCE
- **I12750** (62-255): 1250-1450 calCE
- **I19249** (62-254, ER2): 1250-1450 calCE

Cerro Grande del Paraná Pavón was excavated by Alberto Rex Gozález in 1939 (González, 1947). It is a mound located on the left bank of a small stream on an island adjacent to the Vuelta del Sur of the Paraná Pavón River (Province of Entre Ríos, Department of Gualeguay). Fragments of ceramics (plain, incised, painted, handles, zoomorphic appendages, spoons), bone tools (flat and semi-grooved points), evidence of hearths, mammal remains (*Myocastor coypus*, *Hydrochoerus hydrochaeris*), fish (*Pterodoras granulosus*), and freshwater clams (*Diplodon* sp., *Ampullaria* sp.) were found. A radiocarbon date of 630±45 BP (1297-1426 calCE, AA-103900) was obtained from a skeleton of an indeterminate sex individual (MA n°62-252). Among the human remains, primary incomplete burials, secondary burials, isolated elements, and cremations were found. The samples from Cerro Grande del Paraná Pavón include a right upper canine (62-251, I12748), a molar from an incinerated individual (62-255, I12750), and a molar (62-254, I19249), curated at the Museo de Antropología de Córdoba (Córdoba Province, Argentina).

<u>Cerro Grande de la Isla de los Marinos:</u> 750-1650 CE

- **I19248** (ER1): 750-1650 CE
- **I42413** (SEC19): 774-1214 calCE (1,070±90 BP, LP-2132)

The Cerro Grande site is located on an anthropogenic mound on Los Marinos Island (32°55'26.6"S; 60°33'48.9"W, Victoria Department, Province of Entre Ríos), at 10 meters above sea level, near the confluence of the Paranacito River and Los Confines Creek, between Victoria (Entre Ríos province) and Rosario (Santa Fe province). Excavated by F. Gaspary in 1947, it yielded numerous plain, incised, unguiculated, painted, and modeled pottery (including *campanas*, spoons, spindle whorls, zoomorphic appendages, and ornaments), lithic and bone tools, and faunal remains (jaguar, marsh deer, coypu, fish, and mollusk shells). During recent fieldwork (Bonomo et al., 2010), a topographic survey was conducted, and 27 plain sherds and two lithic debris were recovered from the surface of the site. Gaspary (1950) found 48 human skeletons, some covered with red pigment. Human bones were recorded in primary and secondary burials, dispersed, and burned. Part of these materials has been subject to bioanthropological studies (Kozameh & Brunás, 2011, 2013). The site has four dates based on human bones (Cornero, 2009; Kozameh et al., 2018), 460±50, 590±60, 660±70, and 1070±90 BP, the oldest being sample SEC19 (I42413) obtained for this study from a funerary bundle with pigment remains. The remains of individual I19248 are part of a collection housed in the Patrimonial Reserve of the Museum of Anthropology (FFyH, UNC).

5.3 Argentina_ParanaRiver_LowerDelta

Cementerio Indígena Arroyo Paicarabí y Fredes: 1445-1628 calCE

● **I34939** (MLP-DA-6042): 1445-1628 calCE (421±45 BP, AA-103896)

The Cementerio Indígena Arroyo Paicarabí y Fredes was surveyed in 1921 during an expedition by the La Plata Museum led by the institution's director, Dr. Luis María Torres, along with student Pablo Gaggero and archaeology section preparer Octavio Fernández. Only an unpublished report specified the work conducted at this archaeological site (Gaggero, 1921). This Guarani site is on a raised section of an island in the Paraná Delta, bordered by the Paycarabí and Fredes streams (Buenos Aires Province, San Fernando District). Ceramic fragments and human skeletal remains were recovered there. The bone specimen analyzed here (MLP-DA-6042) consists of a third cervical vertebra covered with red pigment (hematite) from a possibly adult individual of indeterminate sex. An AMS date yielded a radiocarbon age of 421±45 BP (AA-103896) (Bonomo et al., 2015).

El Cerrillo: 1320-1450 calCE

● **I34940** (MLP-DA-6450): 1320-1450 calCE (576±42 BP, AA-93215)

El Cerrillo (34°1´S; 58°41´W) was excavated by Samuel K. Lothrop in 1925 (Lothrop, 1925, 1932). This is the same archaeological site investigated by Torres in 1905, known as Túmulo I del Paraná Guazú (Torres, 1911). It is an earth mound situated on a levee adjacent to a small stream that flows into the right bank of the Paraná Guazú River, between the Paraná Miní course and

the Segunda Campana channel (Buenos Aires Province, Campana District). Considering what was published by Torres (1911) and Lothrop (1932), both excavations covered an area of about 755 m^2, recovering more than 60 human skeletons. The material recovered at this site is housed in the La Plata Museum, Argentina (MLP), and the National Museum of the American Indian, USA (NMAI). From the reanalysis of collections from this site at both institutions (Bonomo et al., 2009; Bonomo, 2013; Ramos van Raap & Bonomo, 2016), a predominance of pottery was observed with 1074 sherds (plain, incised, and corrugated), a small bowl (7.7 cm high by 7.5 cm mouth diameter), three clay masses, and an unfired clay roll. Additionally, four copper sheets, 95 lithic materials (bola stones, mortars, mills, hammers, anvils, projectile points, and unmodified pebbles), and 260 bone materials, including 111 bone tools (conical points, harpoons, bevels, tubes, and perforated cervid forks), were recorded. Bones of unidentified birds and cervids (*Blastocerus dichotomus*, *Ozotoceros bezoarticus*, *Mazama* sp.), Siluriform fish, Ampullariid exoskeletons (*Pomella megastoma*), and freshwater clam shells (*Diplodon parallelopipedon* and *Diplodon* aff. *variabilis*), as well as charred endocarps of pindó palm (*Syagrus romanzoffiana*), were identified.

In the case of human remains, the reanalyzed assemblage (Ramos van Raap & Bonomo, 2016) consists of 241 specimens, from which a MNI of 19 individuals was estimated for Túmulo I del Paraná Guazú and a MNI of 9 individuals for El Cerrillo. The specimen analyzed in this study (MLP-DA-6450) corresponds to a molar from an adult individual of undetermined sex. An AMS radiocarbon date from a right first metatarsal with this same catalog number yielded an age of 576±42 BP (AA-93215) (Bonomo et al., 2011). This incomplete human skeleton, with traces of adhered red pigment, was found alongside mixed faunal, lithic, and ceramic materials, and a layer of ashes beneath the bones. Isotopic data from this individual include $\delta^{13}C_{COL}$= −20.1‰, $\delta^{13}C_{AP}$= −14.5‰, and $\delta^{15}N$= 9.8‰ (UGAMS-11466) (Bonomo et al., 2017).

Arroyo Sarandí: 650-1400 CE

- **I34949** (MLP-DA-6480): 1286-1397 calCE (688±42 BP, AA-93219)
- **I34941** (MLP-DA-6463): 650-1400 CE
- **I34943** (MLP-DA-6467): 650-1400 CE
- **I34944** (MLP-DA-6469): 650-1400 CE
- **I34945** (MLP-DA-6471): 650-1400 CE
- **I34948** (MLP-DA-6478): 650-1400 CE
- **I34946** (MLP-DA-6473): 903-1042 calCE (1080±25 BP, UGAMS-68825)
- **I34947** (MLP-DA-6477): 679-885 calCE (1290±40 BP, UGA-10788)

Arroyo Sarandí (34°23´S; 58°39´W) was excavated by Lothrop in 1925 (Lothrop, 1925, 1932). It is located on the south bank of the Luján River in Pacheco field on an island in Tigre (Buenos Aires Province, Tigre District). This archaeological site consists of two mounds, A and B. In the northern elevation (mound B), a single burial was recorded in 80 m^2 excavated, while 41 burials were found in the southern elevation (mound A) in 225 m^2. The materials from this site are housed in the MLP and NMAI. Among the recently reanalyzed materials (Bonomo et al., 2009;

Bonomo, 2013), ceramics dominate with 395 fragments (plain and incised), two perforated pendants, an entire bowl (14 cm high by 27 cm mouth diameter), a spindle whorl, and a possible pipe mouthpiece, along with twelve masses of clay. Additionally, 40 lithic materials (bola stones and spheroids, projectile points, earplugs, hammers, anvils, mortars, mills, and unmodified clasts) were recorded. Materials on *Glycymeris longior* and cf. *Urosalpinx haneti* shells, used to make 220 beads, were also noted. The 182 bone materials surveyed include 80 tools (points, harpoons, bevels, propulsors, perforated forks, and a possible spoon). Bones of *Ozotocerus bezoarticus*, *Mazama* sp., *Lama* sp. were identified. As for human remains, the reanalyzed assemblage (Ramos van Raap & Bonomo, 2016) consists of 331 elements, from which a MNI of 17 was estimated. The specimens analyzed in this study include MLP-DA-6480 (I34949)**,** a metatarsal from an adult individual of indeterminate sex found articulated, but incomplete (missing both legs and most of the spine). An AMS date from the right clavicle with this same catalog number yielded a radiocarbon age of 688±42 BP (AA-93219) (Bonomo et al., 2011). Isotopic data from this individual include $\delta^{13}C_{COL}$= −19.6‰, $\delta^{13}C_{AP}$= −13.1‰, and $\delta^{15}N$= 12.4‰ (UGAMS-11468) (Bonomo et al., 2017). Other specimens analyzed here include a lower left third molar (MLP-DA-6473, I34946) dated to 1080±25 BP (UGAMS-68825) and a patella (MLP-DA-6477, I34947) from an individual with an earlier chronology of 1290±40 BP (UGA-10788) (Loponte, 2008). These radiocarbon dates on human bones indicate at least three burial events at the site. The other five materials analyzed from this site consist of two molars—one lower left (MLP-DA-6463) and one upper right (MLP-DA-6467)—two left metacarpals (MLP-DA-6469 and MLP-DA-6478), and one right metatarsal (MLP-DA-6471), which have not yet been dated.

Túmulo I del Brazo Gutiérrez: 1226-1389 calCE

●**I35517** (MLP-DA-107, 107MB): 1226-1389 calCE (752±41 BP, AA-72635)

Túmulo I del Brazo Gutiérrez was excavated in 1906 by Luis María Torres (1911), and the collection is housed at the MLP. It is located on a levee 700 m inland from the Brazo Gutiérrez stream (Entre Ríos Province, Islas del Ibicuy Department). From this site, 26 lithic materials (tools, debris, and ecofacts), 6 copper objects (five beads and a perforated plate), and faunal materials from fish (Doradidae) and freshwater mollusks (*Diplodon* aff. *variabilis*) were analyzed (Bonomo et al., 2009). It was characterized as a *tumulus* due to the presence of human skeletons, from which 17 skulls were recovered. Here, a lower left molar (MLP-DA-107, I35517) from an adult of indeterminate sex was analyzed, yielding a radiocarbon age of 752±41 BP (AA-72635) (Bonomo et al., 2011).

Túmulo de Campana: 212-427 calCE

●**I34694** (MLP-DA-402)**:** 212-427 calCE (1754±49 BP, AA-100007)

Túmulo de Campana was excavated by Estanislao Zeballos and Pedro Pico in 1877 (Zeballos & Pico, 1878; Torres, 1907), on the banks of the Paraná River 6 km south of Campana (34°11'27.35"S; 58°55'16.38"W, Buenos Aires Province, Campana District). Recently, the only available archaeological materials from this site, housed at the MLP (sixteen bone artifacts and thirteen ceramic sherds) were reanalyzed (Bonomo et al., 2009; Politis & Bonomo, 2015). Additionally, a human skull of an adult female deposited in the Division of Anthropology of this institution was radiocarbon dated to 1754±49 BP (AA-100007; Loponte & Acosta, 2015). From this specimen, a right lower molar (MLP-DA-402, I34694) was analyzed.

SI Section 6. CENTRAL REGION (ARGENTINA)

Written by Mariana Fabra, M. Pía Tavella, Andrés D. Izeta, G. Roxana Cattáneo, Diego Catriel Leon, Gisela M. Sario, Hilton Drube, Josefina M. B. Motti, and Rodrigo Nores

Plains

The Central Plains, as defined in this work, cover a broad territory that includes the Santiagueña Plains. in the province of Santiago del Estero, as well as the Laguna Mar Chiquita area and the eastern and southern plains of the province of Córdoba. The Santiagueña Plains are located in the transitional sector between the Córdoba plains and the southwestern Chaco region, within the province of Santiago del Estero. This region exhibits marked seasonality, with a subtropical climate and precipitation concentrated during the summer months. Both seasonal flooding, caused by insufficient slope, and periodic droughts have prompted temporary relocations among present-day inhabitants, and may have had similar effects in the past (Taboada, 2017).

Two main watercourses with torrential regimes cross the province from northwest to southeast: the Salado and Dulce rivers. The source of both rivers is in the mountains of Salta and Catamarca provinces, in the Northwest of Argentina, so they have worked as communication routes for human populations from that region and those on the southern and eastern lowlands (Dulce river's mouth is the Mar Chiquita lake in Córdoba, whereas Salado's mouth is the Paraná river in Santa Fe province).

The Dulce and Salado rivers flow almost parallel one to the other, dividing the province in three sectors: the Guasayan and Sumampa-Ambargasta hills to the west and south of Dulce river; the Mesopotamia in between rivers; and to the east are the bushy plains connecting Santiago del Estero to the Gran Chaco and Littoral regions. The mountainous area is considered the source of hallucinogenic seeds and silica material found in archaeological contexts of the plains. The Mesopotamia sector is the most fertile, while the western sector the most arid.

There is uncertainty regarding the dates of the earliest occupation of Santiago del Estero, given the scarce presence of pre-Ceramic archaeological sites with lithic artifacts and big flakes obtained from direct percussion, located in the Sumampa-Ambargasta hills at the south of the province (Gomez, 1975; Gramajo de Martinez Moreno, 1992; Togo, 2004). The following period is

characterized by lanceolate projectile points similar to those from the Intihuasi site (San Luis province, Argentina), dated around 8,000 BP (González, 1960). The human groups associated with these records occupied the Guasayan hills and its surrounding areas, including the ravines of northern Dulce river (Togo, 2004). Triangular-shaped points like those from II and III Intihuasi levels have also been recovered in the area (del Papa, 2012; Gomez, 1975; Gramajo de Martinez Moreno, 1992; Togo, 2004). Recently, based on the analysis of rock art motifs in the southern hills of Santiago del Estero, a connection between hunter-gatherers and the creation of part of the rock art in this area was proposed (Carden & Leon, 2025).

On the other hand, the lowlands between Dulce and Salado river were apparently occupied later than the mountainous sector. The earliest evidence of settlement in the plains of Santiago del Estero includes incised and painted ceramics retrieved from sites in the northern and central areas of the Dulce River. Although traditionally grouped under the Las Mercedes cultural complex (Gómez, 1966), recent research suggests that this designation encompasses multiple ceramic styles (Taboada, 2020; Taboada & Angiorama, 2021). These contexts have been dated between 1,580±60 BP and 1,060±70 BP, with an anomalous case from 700±70 BP (Togo, 2007). They present cultural expressions akin the ones registered simultaneously in the Andean foothills and valleys, such as camelid raising, rock art motifs, and ceramic styles similar to Cienaga, Cortaderas and Candelaria from the Subandean Valleys in the Northwest of Argentina (Carden & Leon, 2022, 2025; Gomez, 1966; Nuñez Regueiro & Tartusi, 1990; Serrano, 1958; Togo, 2004), although with some modifications. The inhabitants of such sites didn't construct mounds (Lorandi, 1978), dams or other interventions which characterized later lowland settlements (Taboada, 2019).

Towards 800-1,000 AD, the bearers of these ceramic styles (broadly classified as the Las Mercedes complex) interacted with groups associated with a ceramic style called Sunchituyoj in the plains of Santiago del Estero, transmitting them some decorative features. At the same time, changes in the settlement patterns marked the rise of what has been called the "Chaco-Santiagueña" Cultural Tradition (Lorandi, 1978) that spread along the Dulce and Salado basins, the Mesopotamia in between, and an area to the east and west of both rivers of unknown extension. This "Tradition" persisted until 1,600 AD and comprised two main ceramic types: Sunchituyoj y Averías. The groups that produced these ceramics installed their settlements atop of mounds, constructed dams of diverse sizes and had a livelihood based on low-yield agriculture combined with haunting, gathering and fishing. They buried their dead either inside urns or at the base of the mounds. While the Sunchituyoj ceramic style was characterized by the owl as a decorative motif, it was not the main figure in Averías; instead, geometric patterns, expressed in different local variants, were predominant.

It has been postulated that the late pre-Hispanic ceramic styles of Santiago del Estero had different geographic origins. Sunchituyoj might be an earlier development from the Gran Chaco (Lorandi, 1978) that didn't spread outside the region. Other less frequent ceramic types, like the incised ones observed in the Mesopotamia and the Salado basin, have been interpreted as a sign of contact with the peoples inhabiting the north of the Dulce river and the mountainous area of Córdoba province. From 1,200 to 1,400 AD, there is evidence in the archaeological record of persistent interactions between Sunchituyoj and Averías ceramics bearers. Some stylistic

elements of Averías complex signaled an origin in the eastern plateau of Bolivia, an area with a mixture of influences from the Andean and Amazonian cultures (Lorandi, 2015: 154). The next stage in Lorandi's chronological typology, estimated between 1,420 and 1,610 AD, is defined by a merging process of Sunchituyoj and Averías ceramic traditions and features. The author's hypothesis is that at least two ethnic groups with different origins might have shared the same territory and settlements in Santiago del Estero's lowlands (Lorandi, 2015). Recent studies have pointed out that the region could have also been inhabited by groups with residential mobility, based on differential patterns of mound installation (Taboada, 2016, 2019).

Towards the end of the pre-Hispanic period, Averías pottery spreads outside the province, reaching the north of Córdoba, the valleys of Catamarca, Tucuman and north of La Rioja provinces, perhaps as a consequence of population movements related to the domination practices of the Inca empire (Lorandi, 1980). Simultaneously, Averías ceramics is associated with an increase in population density and an intensification of agricultural and textile production practices in Santiago del Estero's lowlands. This process is particularly remarkable in the area called Bañados de Añatuya, in the Salado basin's middle sector. The groups settled in this territory held political alliances with the Inca empire from at least 1,200 AD until colonial times. However, earlier connections with populations from the northwestern valleys have been proposed, dating back to the second half of the first millennium AD. (Carden & Leon, 2022). These social networks are reflected in the local archaeological record by objects made with allochthonous materials such as metals, seashells from the Pacific coast and wool knits (Taboada & Angiorama, 2010, which have been interpreted as gifts received in reciprocity for services and workforce (Parssinen, 2003). The presence of bone projectile points, ceramic pipes and other material culture related to the Gran Chaco region in Santiago del Estero has also been interpreted as evidence for pacific interactions with those nomadic groups (Taboada, 2017), at least since 1,000 AD until the arrival of the Spaniard conquerors.

The Laguna Mar Chiquita region lies between 30°-35° S latitude and 62°-66° W longitude, occupying the northern sector of Córdoba Province's eastern and southern plains, which are bounded to the west by the Southern Pampean Hills (Central Hills). These plains converge in the north and northeast of the province into the natural depression of Laguna Mar Chiquita (also known as the Ansenuza Sea). Laguna Mar Chiquita is a large, shallow saltwater lake situated in a tectonic depression formed during the Middle Pleistocene. The area is characterized by a flat landscape interspersed with lowlands and depressions, and features the distinctive vegetation of the Chaco forest, and is crossed by significant rivers flowing southwest-northeast (Río Primero or Suquía, and Río Segundo or Xanaes), with considerable seasonal fluctuations in their flow, which at times create levees, small lagoons, and marshes in the lowlands (Piovano et al., 2006).

Bioarchaeological research has focused on studying the lifestyles of populations that inhabited various ecological environments within the current territory of Córdoba (Fabra, 2009; 2013; 2014; Fabra & González, 2015, 2019). These studies have revealed continuities and changes in the lifestyle of these populations over the last 4,500 years, particularly through differences in the frequency of certain pathologies (Fabra & González, 2015), as well as changes in δ13C isotopic values (Fabra & González, 2019; Laguens et al., 2009). These suggest shifts in diet (e.g., incorporation of produced foods) and food preparation methods around 1,500-1,200

BP, without abandoning a mixed subsistence strategy (González & Fabra, 2018). In the region surrounding Laguna Mar Chiquita, archaeobotanical evidence indicates that cultivated plants, such as common beans (*Phaseolus vulgaris*), may have been incorporated into the diet around 1,200 BP, while the consumption of maize (*Zea mays*) and squash (*Cucurbita maxima*) occurred later, approximately 1,000 BP (Tavarone et al., 2019). In the mountainous region, results suggest the consumption and/or manipulation of *Geoffroea decorticans, Prosopis* sp., and, for the first time, *Trithrinax campestris* throughout the late Holocene. Additionally, they revealed the incorporation of *Zea mays* (Poaceae) around 1,200 BP, followed by *Cucurbita* sp., *Phaseolus* sp., and *Solanum* sp. (Tavarone et al., 2020a). These studies indicate that maize consumption was not predominant among these populations (Fabra & González, 2019). The δ15N values suggest a diet rich in animal proteins after approximately 1,200 BP, particularly among males, which poses an unusual scenario for the inclusion of domesticated plant resources.

The analysis of dental pathologies suggesting systemic-metabolic stress led to the inference of a deterioration in health in the centuries preceding the Spanish conquest (Da Peña Aldao et al., 2024; Fabra & González, 2015), as well as an increase in physical activity levels and its onset at an early age (Salega, 2016; Salega & Fabra, 2013). The deterioration in quality of life, competition for scarce resources, and population growth may have contributed to instances of interpersonal violence, as suggested by bioarchaeological records from the late Holocene (Fabra et al., 2015). Other studies identified an increase in sexual dimorphism over time, characterized by a secular negative trend in stature among females and a slight increase in male height (Loupias & Fabra, 2019). The authors suggest that these trends cannot be explained by a single factor but rather by a combination of environmental and genetic influences shaping stature over time.

Research into body usage began with studies on degenerative changes and Schmorl's nodes (Salega & Fabra, 2013), followed by analysis of modifications in muscle attachment sites (Salega, 2017; Salega & Fabra, 2017). New research lines have been developed, including archaeomalacological, mortuary, and paleopathological studies focused on congenital diseases (Fabra & Salega, 2016; Salega & Fabra, 2018). Notably, biological connections were established within a context of interpersonal violence, dated to 800 BP (Fabra et al., 2015a; Nores et al., 2020). Previous research on burial practices (Fabra et al., 2009) highlighted a wide variety of inhumation practices, both in the mountains and plains (González & Fabra, 2019). Taphonomic studies were also conducted on human remains, particularly those recovered from lagoon contexts (Tavarone et al., 2017).

In terms of biological variability, studies have advanced knowledge of the microevolutionary processes that occurred over time among these populations, through analysis of craniofacial morphological variability (Fabra, 2008; 2013, 2014; Fabra & Demarchi, 2009, 2011, 2012), epigenetics (Fabra et al., 2005), and ancient DNA (Nores et al., 2011, 2017). Molecular evidence suggests biological differentiation between populations inhabiting the Sierras (mountains) and Plains after 1,200 BP (Nores et al., 2011). Significant differences in the distribution of mitochondrial haplogroups in the subpopulations occupying the Sierra region and plains surrounding Laguna Mar Chiquita suggest distinct migratory contributions to these subregions after approximately 1,200 BP. It was proposed that the high frequency of lineage C among the

older Sierras samples and the predominance of lineage B among the more recent ones (47.6%) could be due to migration from populations with high incidence of haplogroup B, such as the Andean (puna and Atacama) and Chaco (Wichí and Toba) groups, or the ancient Pampa Grande population from Salta (Nores et al., 2011).

However, subsequent studies on mitochondrial DNA control region sequences and complete mitogenomes from archaeological samples challenge this interpretation, as the sub-lineages within haplogroup B typical of the Andean and Chaco regions were not found. Instead, unique sub-lineages for central Argentina with significant temporal depth were identified, suggesting a local origin dating back to the early settlement period (García et al., 2021). On the other hand, the epigenetic traits do not show a clear trend, although differences have been suggested among the various regions that make up the current province of Córdoba (Fabra et al., 2005). The results obtained from ancient mitochondrial DNA studies (Nores et al., 2011, 2017), in line with findings from other research conducted by our group based on morphological traits of ancient populations and morphogeometric analyses (Fabra, 2008, 2014; Fabra & Demarchi, 2011, 2013), as well as genetic studies of the contemporary population (García et al., 2012, 2015, 2018), suggest that the population dynamics of human groups in central Argentina have been deeply influenced by geography. Its intermediate location, relatively distant from other regions, and its function as a crossroads without insurmountable geographic barriers, have shaped population evolution over time, with gene flow being the predominant evolutionary force.

Southern Pampean Hills

The Southern Pampean Hills, referred to in the main text as the Central Hills, are formed by a series of mountain ranges that extend in a north-south direction in parallel in the provinces of Córdoba and San Luis (Cattáneo et al., 2015; Laguens & Bonnin, 2023). These mountain ranges offer a greater diversity of spaces with more varied flora and fauna compared to the plains (Silva et al., 2011).

In the mountainous area, human occupations dating back to the Pleistocene-Holocene boundary have been identified, allowing for the observation of persistent occupation throughout the Holocene (Izeta et al., 2017; Rivero, 2012). To date, more than 2,300 archaeological sites or locations have been recorded, which evidence a succession of occupations by hunter-gatherer groups, producers, and later, the incorporation of indigenous societies into a globalized economy (Izeta & Cattáneo, 2023). This process began with the arrival of the Spanish in the late 16th century.

The earliest human occupations in the mountainous sector were defined based on the discovery of archaeological material in stratigraphic and surface contexts (Cattáneo et al., 2016; Rivero, 2012; Rivero et al., 2018). Although radiocarbon dating for this period is scarce, the evidence suggests the presence of hunter-gatherer groups carrying bifacial technology, characterized by "fishtail" projectile points (PCP or FTPP) (Cattáneo & Izeta, 2023; Izeta, 2024). As in other regions, this technology was replaced by others, which in the Córdoba area has been interpreted as the result of a failed colonization of the space by these early Paleoindian

hunter-gatherers (Recalde & Rivero, 2018; Rivero, 2012). However, this model lacks sufficient support, as the archaeology of hunter-gatherers in the region is far from being resolved. Therefore, models from neighboring areas have been adapted to fill information gaps, a consequence of scientific and academic policies that have not prioritized the study of these societies (Cattáneo & Izeta, 2023; Izeta, 2024).

In fact, occupations corresponding to the Middle Holocene and the initial Late Holocene have only begun to be studied in depth in the last 15 years, through a multidisciplinary approach that includes taphonomic studies and formation processes in various archaeological contexts (Cattáneo & Izeta, 2016; Laguens & Bonnin, 2023).

At the beginning of the first millennium CE, ceramic technology was introduced in the region, a phenomenon that occurred simultaneously in neighboring areas such as Cuyo and northern Patagonia (Izeta, 2024; Nóbile et al., 2025). Along with the adoption of domesticated plants, a demographic increase is observed, reflected in the number of sites and archaeological remains associated with this period (Cattáneo et al., 2015; Laguens, 1999; Medina, 2015). Additionally, a new weapon system, the bow and arrow, was incorporated during this time, marking a significant shift from the technologies used for millennia (Rivero et al., 2024). However, residence and mobility patterns remain similar to those of earlier societies, so a full process of sedentarization cannot be proposed for this time frame (Cattáneo et al., 2022; Laguens & Bonnin, 2023; Medina, 2015).

In the 16th century, contact with the Spanish occurred, leading to a profound social, political, and economic restructuring of these societies. These communities were integrated, in terms of subalternity, into the colonial world and, later, into the Argentine nation-state. It was not until 1992 that a process of reemergence of indigenous communities began, for whom archaeological, bioanthropological, and genetic information has been of particular interest.

6.1 Argentina_Central_Plains_MesopotamiaSantiagoDelEstero

Tiestituyoj: 1506-1647 calCE

●**I14569** (38SE): 1506-1647 calCE (335±20 BP, PSUAMS-15102)

Lugones: 1450-1700 CE

●**I12741** (2SE):1450-1700 CE

Both Tiestituyoj and Lugones sites are located in between Dulce and Salado river courses, in the Avellaneda department (Santiago del Estero Province). Although there is no detailed information in the literature regarding them, other archaeological sites of the same mesopotamic area -such as Santa Rosa and Quimili Paso- were probably occupied at least since 1200 C.E. These settlements present a combination of their cultural features and others associated with neighboring areas, such as incised ceramics similar to those found in Córdoba

province but decorated with the owl motif, typical from Santiago del Estero's lowlands (Taboada, 2019).

Bañados de Añatuya:

There is no precise contextual information provided for the following individuals, since samples were obtained from a museum collection formed mainly by Emilio & Duncan Wagner in the beginning of the 20th century, which did not possess sufficient records. Nonetheless, it is safe to assume that they proceed from the Bañados the Añatuya area, where the Wagner brothers carried almost all their archaeological work.

- **I14575** (45SE): 1507-1649 calCE (330±20 BP, PSUAMS-15104)
- **I12738** (25SE): 1450-1700 CE
- **I14554** (1SE): 1400-1700 CE
- **I14555** (3SE): 1400-1700 CE
- **I14556** (5SE): 1400-1700 CE
- **I14558** (10SE): 1400-1700 CE
- **I14565** (33SE): 1400-1700 CE
- **I14573** (42SE): 1400-1700 CE
- **I14574** (44SE): 1400-1700 CE
- **I12745** (46SE): 1400-1700 CE
- **I14561** (28SE): 1452-1623 calCE (415±20 BP, PSUAMS-15098)
- **I14564** (31SE): 205-340 calCE (1820±20 BP, PSUAMS-15099)

For the remaining Wagner collection's individuals analyzed in this study, information obtained from archaeological prospections and literature research conducted by Taboada & Angiorama (2021) in an attempt to geographically locate the sites originally excavated by the Wagner brothers, is summarized below.

Laguna Muyoj: 1500-1650 CE

- **I12742** (32SE): 1505-1646 calCE (340±20 BP, PSUAMS-15097)
- **I12723** (13SE): 1500-1650 CE
- **I12730** (16SE): 1500-1650 CE
- **I12736** (23SE): 1500-1650 CE
- **I12739** (26SE): 1500-1650 CE

Laguna Muyoj was excavated by Wagner & Wagner (1934), Reichlen (1940), Lorandi (1974) and Gramajo de Martínez Moreno (1991). Taboada and Angiorama (2021) conducted prospections in a site with the same name but are not certain it corresponds to the one from the pioneer studies. Reichlen (1940) claimed to have found a remarkable amount and variety of bone artifacts. There is also a varied ceramic collection proceeding from Laguna Muyoj, as well

as nine metal objects from pre-Inca, Inca, and colonial times (Angiorama & Taboada, 2008; Gramajo de Martínez Moreno, 1991; Reichlen, 1940).

Chilca Pozo: 1500-1650 CE

●**I12735** (21SE): 1502-1643 calCE (350±20 BP, PSUAMS-15096)
●**I14559** (15SE): 1500-1650 CE
●**I12734** (20SE): 1500-1650 CE
●**I14571** (40SE): 1500-1650 CE
●**I14572** (41SE): 1500-1650 CE

Chilca Pozo is located in the Avellaneda department (Santiago del Estero province). According to Reichlen (1940), foreign objects coming from this site are housed in museum collections (e.g., a silver Inca brooch or *tupu*). However, these materials are fewer and less varied than those found in nearby sites such as Sequía Vieja or Averías (Taboada, 2014).

Icaño: 1400-1700 CE

●**I12737** (24SE): 1400-1700 CE

Icaño site, located in the Avellaneda department (Santiago del Estero province), is mentioned in Wagner & Wagner (1934), Reichlen (1940), Lorandi (1974), and Gramajo de Martínez Moreno (1991). Lorandi (1974) ascribes nearly all the ceramics recovered on this site exclusively to the Averías complex (a presumably late ceramic type). Both Lorandi (1974) and Gramajo de Martínez Moreno (1991) report finding objects from colonial times. Taboada and Angiorama (2021) prospected a sector of Icaño field site and found ceramic fragments classified as Sunchituyoj type (considered as belonging to the middle stage of the local chronological typology). They also registered part of a human skull. According to the authors, it is possible that Icaño had various components or sectors, associated with different moments of a prolonged occupation period (Taboada & Angiorama, 2021).

Tulip Loman: 1400-1700 CE

●**I12740** (27SE): 1400-1700 CE
●**I14560** (19SE): 1400-1650 CE

This site from the Avellaneda department (Santiago del Estero province) was studied by Wagner & Wagner (1934), Reichlen (1940) and Lorandi (1974). Lorandi (1978) ascribes it to the latest stage of her chronological typology due to the preponderance of the Averías ceramic style over other types. Most of the thousands of spinning whorls stored in museum collections come from this site, as well as seven metal objects: two from the Inca period, two possibly from

colonial times and three of undetermined age (Angiorama & Taboada, 2008). The finding of glass beads has been interpreted as evidence for occupation until colonial times.

<u>Pueblo Nuevo:</u> 1400-1700 CE

●**I14557** (8SE): 1400-1700 CE
●**I14562** (29SE): 1400-1700 CE
●**I14563** (30SE): 1400-1700 CE

This site is located in the Figueroa department, 10-15 km east of the current course of the Salado River. It was studied by the Wagner brothers, who recovered several human remains, three of which are included in this study.

<u>Las Marías:</u> 1400-1700 CE

●**I14570** (39SE): 1400-1700 CE

This site is located on the eastern margin of the Salado River, approximately 35 km up north from the Bañados de Añatuya area. Although there is no specific information regarding Las Marías in the literature, other archaeological sites from the same area, like Llajta Mauca, Las Represas de los Indios, and Oloma Bajada, have been exhaustively studied and could provide a reference. The occupation of these sites has been estimated to be from late pre-Hispanic to colonial times, based on radiocarbon dates (Taboada & Farberman, 2014). It is worth mentioning that no metal or other Andean objects have been retrieved from this area. It has neither yielded large amounts of ceramic pipes nor spinning whorls. The ceramic fragments, though similar to the Averías style, have several variations in decoration and shape (Bleiler, 1948). When Averías ceramics do appear, they are associated with Sunchituyoj pieces. The sites located east of the Salado River don't appear to have other evidence of long-distance interactions. It has also been hypothesized that they were never subjected to Spanish domination (Taboada & Farberman, 2014).

<u>Cañitas:</u> 1450-1650 CE

●**I14566** (34SE): 1457-1626 calCE (395±20 BP, PSUAMS-15100)
●**I14568** (37SE): 1450-1650 CE

Cañitas site is located in the Avellaneda department. It was excavated by Wagner & Wagner (1934), Reichlen (1940), Lorandi (1974), and Gramajo de Martínez Moreno (1991). Based on the ceramic styles found on the site, Lorandi considered this settlement as belonging to the latest stage of her chronological typology. However, grey ceramic fragments found in recent prospections could indicate Cañitas had an earlier component yet to be identified. There is evidence to hypothesize the site could have been occupied around, at least, 1000 BC. (Taboada &

Angiorama, 2021).

<u>Averías:</u> 1400-1650 CE

●**I12716** (11SE): 1400-1650 CE
●**I12743** (35SE): 1400-1650 CE

Averías site is located in the Avellaneda department, in Santiago del Estero's lowlands. The archaeological site named "Averías" by Emilio & Duncan Wagner (1934) is one of the most remarkable ones in the Bañados de Añatuya area due to its abundance in metal artifacts, ceramic pipes and spinning whorls. According to Silverio Carrizo, Emilio's former collaborator, this site corresponds to the one called "Mancapa" in more recent archaeological research (Taboada, 2014). There are three radiocarbon dates from "Mancapa" site that confirm it was permanently occupied between 1250 and 1650 cal. d.C. (Taboada, 2017; 2019).

<u>Mancapa:</u> 1455-1627 calCE

●**I12732** (18SE)**:** 1455-1627 calCE (400±25 BP, PSUAMS-15103)

Mr. Carrizo pointed out a site 3 km away from Averías as the Mancapa site excavated by the Wagner brothers (Reichlen, 1940; Wagner & Wagner, 1934), but this affirmation has not been proven yet (Taboada & Angiorama, 2021). Recent archaeological prospections in the site suggest it could have been part of a broader archaeological field.

<u>Pozo del Medio:</u> 1457-1626 calCE

●**I14567** (36SE): 1457-1626 calCE (395±20 BP, PSUAMS-15101)

There is no precise contextual information provided for this site, from where at least one individual was recovered by Emilio & Duncan Wagner in the beginning of the 20th century. Nonetheless, it is safe to assume that they proceed from the Bañados the Añatuya area, where the Wagner brothers carried almost all their archaeological work.

<u>Estancia La Lorenza:</u> 450-1450 CE

●**I30889** (A909, cl4): 450-1450 CE

Estancia La Lorenza is located approximately 8 km northeast of the town of Pinto, in the Aguirre department, on the right bank of the Salado River, within a xerophytic forest. On a mound at this site, amateurs discovered 47 human remains of an adult, along with five assorted ceramic fragments. Due to the presence of these archaeological materials on a mound, they can be assigned a relative chronology of between 500 and 1000 BP. Staff from the former Emilio and

Duncan Wagner Provincial Museum (now the Directorate General of Cultural Heritage) collected these remains from the town of Pinto and assigned them the census identifier A909. One sample (I30889) from this collection was analyzed in this study. The approximate coordinates are 29°4'54.03''S, 62°38'43.92''W.

Villa Salavina: 450-1450 CE

- **I30891** (VS01/19, CL6): 450-1450 CE
- **I30892** (VS02/19, CL7): 450-1450 CE

The Villa Salavina site (28°48'33.36"S, 63°24'32.58"W) is located within a xerophytic forest in the town of Villa Salavina, Salavina department. In 2019, a local guide unearthed an inverted ceramic container holding human skeletal remains, possibly from three individuals, and sediment. The Directorate General of Cultural Heritage of the province retrieved it from the site and cataloged it as a secondary burial in an urn (CICPSGE 01/19). The container exhibits subglobular morphological characteristics, with a rough, plain finish and flat horizontal handles, allowing it to be assigned a relative chronology between 500 and 1000 BP. Two samples (VS01/19 and VS02/19), analyzed in this study confirm the presence of at least two individuals.

6.2 Argentina_Central_Plains_NorthSantiagodelEstero

Campo Gallo: 1404-1443 calCE

- **I30890** (A907, CL5): 1404-1443 calCE (550±20 BP, UGAMS-68798)

The Campo Gallo site (26°36'33.7”S, 62°51'28.7”W) is located in the Alberdi department, in the northeast of Santiago del Estero Province, within a xerophytic forest. Following reports in the media about archaeological discoveries, staff from the former Emilio and Duncan Wagner Provincial Museum (now the Directorate General of Cultural Heritage) carried out a salvage operation in November 2011, on the estate of Mr. Llarul. They recovered a primary burial of an adult individual in a fetal position, accompanied by three projectile points (two lithic and one bone). A total of 147 specimens were recovered, many of them fragmented, and they were assigned the census identifier A907.

6.3 Argentina_Central_Plains_NorthDulceRiver

Bocatoma/Vilmer: 1300-1700 CE

- **I14576** (H48-3846, 47SE): 1300-1700 CE
- **I14577** (H48-3846, 48SE): 1300-1700 CE
- **I14578** (H48-3844, 49SE): 1300-1700 CE
- **I14579** (H48-3889, 50SE): 1300-1700 CE

● **I14584** (H48-5888, 57SE): 1300-1700 CE
● **I14580** (H48-5886, 52SE): 1396-1439 calCE (570±20 BP, PSUAMS-15105)
● **I14583** (66-22, 55SE): 1307-1399 calCE (655±15 BP, PSUAMS-7676)

The von Hauenschild archaeological collection, housed in the Anthropology Museum of the National University of Córdoba, was created from the materials excavated by the German engineer Jorge von Hauenschild from the 1920s until his passing in 1951. He began his first explorations in a locality called Los Quiroga, on the left margin of the Dulce River, in the Banda department, Santiago del Estero. From there, he moved forward following the river course from Southeast to Northwest. He excavated around 25 archaeological sites in an area of 50 km, centering his work in the localities of Vilmer and Bocatoma (Lindskoug, 2008). Although these individuals lack precise provenance records, they were attributed to the latter sites.

Bocatoma: 1484-1633 calCE

●**I14582** (54SE): 1484-1633 calCE (370±20 BP, PSUAMS-15106)

Bocatoma is an archaeological locality formed by five sites nearby an ancient water outlet by the Dulce River, in the Banda department. One of the sites of the left margin of the Dulce, called Bajadita Norte, might have yielded "an accumulation of funerary urns disposed as a sort of cemetery" (von Hauenschild, 1949: 63). The Bocatoma site itself is an extension of Bajadita Norte and has similar features. However, Bocatoma was greatly altered by the construction of a water outlet in the 18th century and, later on, a canal. According to von Hauenschild (1949), at the end of the 17th century Bocatoma was briefly occupied by the Quilmes people (from Northwest Argentina) on their exodus. On the other hand, another sector of the site called Bajadita Sud would have been a much earlier settlement. In Bocatoma, abundant projectile points and stone axes were found on the surface, but no bone artifacts (von Hauenschild, 1949). Both Bajadita Norte and Bocatoma yielded ceramic fragments of the "black over orange" type. According to Gómez ([1976] 2009: 65), this ceramic type could be assigned to an early stage of the Sunchituyoj complex.

Media Flor-Vilmer: 1300-1800 CE

●**I35526** (VL09): 1300-1800 CE
●**I35527** (VL10): 1300-1800 CE
●**I35528** (VL14): 1300-1800 CE

Media Flor-Vilmer is a site located in the Robles Department, on the border with the Banda Department. The archaeological site extends between the adjacent locations of Media Flor and the northern area of the small town of Vilmer. The settlement is located on the northeastern side of an old branch of the Dulce River, barely noticeable today due to intense agricultural practices and the advances in urbanization in the region. The geographical coordinates correspond to

27°46'09.36" S and 64°09'33.41" W. The elevation of the area is 183 m.a.s.l. Archaeological work was carried out in 2000 and, mostly, in 2001, although the site was not completely excavated (Togo, 2004; Drube, 2009). Before the archaeological work, residents of the area had recovered human remains from burial sites and ancient ceramic material, currently deposited in the Laboratory of Anthropology of the National University of Santiago del Estero. Unfortunately, the area has been significantly changed by anthropic activities in recent years, with the development of commercial enterprises, peri-urban housing, and crop fields. I35526 corresponds to a subadult individual of indeterminate sex, estimated to be approximately 7 years old (± 24 months) based on dental development and eruption (Drube, 2009). It was a secondary burial in an urn, recovered by the local population. I35527 is an adult, likely male, with an estimated age between 24-30 years and a height of 164.11 cm±3.8 (Drube, 2009). This individual was found as a primary burial, discovered incidentally by rural workers. Lastly, I35528 consists of a fragmentary and incomplete skull belonging to an adult of indeterminate sex. These remains were unearthed at the base of a low mound and collected by local residents following its incidental discovery (Drube, 2009).

The radiocarbon dates obtained from remains of hearths, dated on charcoal from 490±70 BP (1327-1628, LP-1357) to 250±70 BP (1505-1950 calCE, LP-1307), chronologically place the site at the time of contact, even in times during the Spaniard colonization (Togo, 2004). However, no cultural elements that denote the Hispanic presence have been detected in the excavated areas, except for a single bone element from *Bos taurus* among the archaeofauna, considered probably intrusive at the site (del Papa, 2012). The ceramics of the site are assigned to the Averías complex, with a predominance of the tricolor decorated group, with strong and thick colors, and an important presence of the polished red group. The presence of ceramics that could be assigned to the Sunchituyoj type is practically nonexistent (Togo, 2004).

6.4 Argentina_Central_Plains_WestSantiagodelEstero

Villa La Punta-Guayacán: 400-650 CE

●**I35531** (VP07): 400-650 CE

Villa La Punta-Guayacán is an archaeological site located in the Choya department, in the eastern piedmont area of the southern end of the Guasayán hills, north of the town of Villa La Punta. This site was called Guayacán due to the presence of an impressive specimen of the tree species *Libidibia paraguariensis*. The geographical coordinates correspond to 28°20'50.66" S and 64°47'4.25" W. The elevation of the area is 410 m.a.s.l. Villa La Punta-Guayacán site was excavated in 2003 and 2004, although skeletal remains and cultural material had been previously recovered by residents of the area, and are currently deposited in the Laboratory of Anthropology of the National University of Santiago del Estero. The settlement is characterized by being crisscrossed by shallow gullies, which remain without water for most of the year. The ceramics of the site correspond entirely to the Las Mercedes cultural complex and are characterized by the predominance of fine gray ceramics and a notable presence of tricolor

ceramics, with geometric decorative designs. Incised forms are scarce (Togo, 2004). At the site, absolute dates obtained from hearth samples correspond chronologically to the oldest dates for the Las Mercedes cultural entity to the present, with radiocarbon ages on charcoal of 1550±60 BP (422-645 calCE, LP-1443) and 1580±60 BP (412-641 calCE, LP-1438) (Togo, 2004). One sample analyzed in this study (I35531) comes from a female adult individual, exhumed from a direct primary burial. Estimated age within the range of 25-30 years old with an approximate height of 156.86±4.24 cm. This individual shows lesions consisting of calcified subperiosteal hematomas in the facial skeleton, neurocranium, and appendicular bones, compatible with a probable case of scurvy. It also displays marked alveolar retraction and antemortem teeth loss (Drube et al., 2016).

6.5 Argentina_Central_Plains_SouthCordoba

Barrio Alberdi Río Cuarto: 1688-1950 calCE

●**I34676** (292/11, 180): 1688-1950 calCE (150±25 BP, UGAMS-68821)

This open-air site was identified by the discovery of human remains during construction work at a private residence in Barrio Alberdi (Río Cuarto, Río Cuarto Department, Córdoba, coordinates 33°8’161”S, 64°18’627”W). The remains were partially exhumed by workers. Recovery was later completed by the Human Remains Recovery Team (ERARO, PJ, FFyH) in 2011. The individual was found in a flexed right lateral position. Laboratory analysis indicated the remains belong to an adult female, over 35 years of age. The remains are housed in the Archaeology Area, Cultural Heritage Directorate, Córdoba Culture Agency, Government of Córdoba Province.

6.6 Argentina_Central_Plains_EastCordoba

Barranca del Lagarto: 1425-1625 calCE

●**I34675** (1332/17, 183): 1425-1625 calCE (450±40 BP, LP-3882)

This open-air site was identified by the discovery of human remains in the current camping grounds of Río Primero (Río Primero Department, Córdoba), about 150 m north of the current course of the Río Primero (31°20.046"S, 63°36.428"W). The remains were recovered from an artificial mound by the Human Remains Recovery Team (ERARO, PJ, FFyH) and the Public Archaeology Program (SEU, FFyH, UNC) in 2017. Alongside the human remains, archaeological ceramics were also recovered. No bioanthropological analysis has been conducted on the remains to date. The remains are housed in the Archaeology Area, Cultural Heritage Directorate, Córdoba Culture Agency, Government of Córdoba Province.

Pampayasta: 1250-1500 CE

●**I17035** (I. 1186, 20): 1250-1500 CE
●**I17036** (22): 1321-1417 calCE (610±20 BP, UGAMS-68771)

These remains correspond to two adult individuals (samples 1186 and Pampayasta 22) housed at the Florentino Ameghino Museum (located in Río Tercero, Tercero Arriba Department, Córdoba). They were recovered by amateurs, resulting in a lack of detailed contextual information.

Arroyito La Villa: 1250-1500 CE

●**I34463** (137/09 I. 3, 156): 1314-1405 calCE (640±20 BP, UGAMS-68812)
●**I40451** (137/09 I. 2, 154): 1250-1500 CE
●**I34462** (137/09 I. 1, 155): 1250-1500 CE

This open-air site is defined by human remains discovered in an urban area during sewer installation along a 20 m stretch of Osvaldo Navarro Street. The police recovered the remains, with no contextual information or burial orientation. Exhumation was conducted by the Rescue Archaeology Team (Museum of Anthropology, FFyH, UNC) in 2009. Bioanthropological analysis identified a minimum of three individuals: one young female (Individual 1) and two adult males (Individuals 2 and 3). The remains are housed in the Archaeology Area, Cultural Heritage Directorate, Córdoba Culture Agency, Córdoba Provincial Government.

Campo Piergentilli: 1220-1394 calCE

●**I34678** (226/19, 175): 1220-1394 calCE (750±60 BP, LP-3794)

This open-air site was identified by the discovery of human remains and archaeological materials (ceramics, animal bones, lithic) during gravel extraction on a private property in Capilla del Carmen (Río Segundo Department, Córdoba, 31°30'12.5''S, 63°22'21.3''W). The remains were recovered from an artificial mound by the Human Remains Recovery Team (ERARO, PJ, FFyH) and the Public Archaeology Program (SEU, FFyH, UNC) in 2019. Subsequent bioanthropological analysis identified a minimum of three individuals, though no burial information or associations with other materials were available. The remains are housed in the Archaeology Area, Cultural Heritage Directorate, Córdoba Culture Agency, Government of Córdoba Province.

Arroyito Campo Sismondi: 1150-1350 CE

●**I17034 (**705/15, 163): 1219-1280 calCE (825±23 BP, AA111061)
●**I19224 (**648/15, 161): 1150-1350 CE

This is an open-air site identified by the discovery of human remains during agricultural work on land owned by the Sismondi family in the town of Arroyito, San Justo Department, Córdoba Province. Excavations were carried out in two campaigns: the first at the site designated as 648/15 Arroyito Campo Sismondi (31°26’27.6”, 63°05’1.5”W) and the second at site 705/15 Arroyito Campo Sismondi (LS 31°23’284, LW 64°11’210’’). Both excavations were conducted by the Human Remains Recovery and Analysis Team (ERARO) in 2015.

At site 648/15, two individuals were recovered, one of whom was identified as possibly male, aged between 20 and 35 years at the time of death. Due to fragmentation and the absence of diagnostic elements, it was not possible to determine the age or sex of the second individual. One of the individuals displayed a reddish coloration, suggesting the presence of pigments as part of a mortuary treatment.

Site 705/15, located near the first site, contained the semi-buried remains of a single individual, an adult male, aged approximately 29±2 years at the time of death, associated with three bone projectile points. The remains of the individuals recovered from both sites are housed in the Archaeology Area, Cultural Heritage Directorate, Córdoba Culture Agency, Government of Córdoba Province.

Arroyito Osvaldo Navarro: 886-1144 calCE

●**I34677** (158/17,164): 886-1144 calCE (1100±50 BP, LP-3660)

This site was defined by the discovery of human remains during construction in a private home (31°24'47.13"S, 63°3'4.35"W) in the city of Arroyito (San Justo Department, Córdoba), on the same street where remains had been recovered in 2009 (samples 154, 155, 156). Exhumation was conducted by the Human Remains Recovery Team (ERARO, PJ, FFyH) and the Public Archaeology Program (SEU, FFyH, UNC) in 2017. A single individual was identified, buried in a right lateral decubitus position, with hyperflexed legs to the same side. The remains are housed in the Archaeology Area, Cultural Heritage Directorate, Córdoba Culture Agency, Córdoba Provincial Government.

Tío Pujio/James Craik: 1876-1626 calBCE

●**I17030** (213/11, 134): 1876-1626 calBCE (3460±25 BP, UGAMS-68769)

This open-air site is located 15 km from Tío Pujio and about 2.5 km south of Route 9 (between Tío Pujio and James Craik), in the General San Martín Department, Córdoba Province (32°16'40.2''S, 63°29'33.9''W). At the request of the Third Turn Prosecutor's Office, Secretariat No. 5, Villa María City, excavation was conducted in 2011. The remains, partially exposed due to the intervention of machinery during the construction of an irrigation ditch, include portions of the skull, facial bones, right scapula, proximal portion of the right tibia, and distal portion of the right femur. Bioanthropological analysis identified a middle-aged adult male, approximately 45

years old±5 years at death. The remains are housed in the Archaeology Area, Cultural Heritage Directorate, Córdoba Culture Agency, Government of Córdoba Province.

6.7 Argentina_Central_Plains_MarChiquita

El Diquecito: 803 calBCE - 1445 calCE

●**I40457** (I1CP, 125): 1313-1445 calCE (597±41 BP, AA93744)
●**I17029** (I1CA, 124): 1282-1395 calCE (698±42 BP, AA93743)
●**I17025** (I1CB, 101): 1160-1410 calCE (750±85 BP, MTC-12807)
●**I12719** (I2CB, 130): 1160-1410 calCE
●**I40456** (I1CL, 96): 774-1394 calCE (937±150 BP, MTC-13214)
●**I17026** (I1CE, 102): 772-992 calCE (1192±40 BP, MTC-13247)
●**I17027** (I1CG, 103): 41 calBCE - 332 calCE (1911±59 BP, MTC-14023)
●**I13562** (I1CS, 126): 460-188 calBCE (2331±46 BP, AA-93745)
●**I12717** (I1CQ, 121): 750-203 calBCE (2400±47 BP, AA-93740)
●**I12712** (I1CO, 122): 756-388 calBCE (2438±47 BP, AA-93741)
●**I13561** (I1CR, 123): 803-425 calBCE (2562±47 BP, AA-93742)

It is an open-air site, defined by the appearance on the surface of archaeological remains—ceramic fragments, lithic material, circular structures or jars, and dispersed human skeletal remains over an area of 500 m along the current coast of the lagoon (Fabra et al., 2008). The human remains from this site were recovered between 2008 and 2010 through public archaeology work on the coast of the Mar Chiquita lagoon (San Justo Department, Córdoba), 15 km from the town of La Para. The excavation was conducted by members of the Rescue Archaeology Team (Museum of Anthropology, FFyH, UNC). A total of 12 individuals were recovered, with chronologies ranging from 2,500 to 500 BP, associated with contexts of interpersonal violence (Fabra et al., 2015; Canova et al., 2024) and mortuary accompaniments with materials from other regions (Fabra et al., 2012; Fabra & Gordillo, 2015; Canova et al., 2020). Bioanthropological, bioarchaeological (Fabra & Demarchi, 2012; Fabra et al., 2012), archaeomalacological (Fabra et al., 2012; Salega & Fabra, 2013), genetic (Nores et al., 2011), archaeobotanical (Tavarone et al., 2020b), and taphonomic (Tavarone et al., 2017) studies have been carried out, demonstrating the importance of this site for regional archaeology. The individuals are housed in the Museum of Anthropology (FFyH, UNC).

El Mistolar: 1224-1432 calCE

●**I12603** (MIR1 - ZMIS5, 55): 1224-1432 calCE (690±85 BP, MTC-12805)

An adult male with stable isotope values δ13C(col)= -15.6‰ and δ15N= 12.3‰ (mixed diet). The remains are housed in the Aníbal Montes Museum of Natural Sciences, Miramar, Córdoba.

<u>Isla Orihuela:</u> 1000-1500 CE

●**I12755** (I2, 99): 1225-1386 calCE (761±41 BP, AA93746)
●**I12605** (I1, 129): 1000-1500 CE

The human remains of two individuals were recovered through rescue archaeology efforts (sample 99) and by an amateur (sample 129) on Orihuela Island, located in Mar Chiquita Lagoon (San Justo Department, Córdoba). The excavation was carried out in 2007 by the Public Archaeology Program (SEU, FFyH, UNC). Individual 1 corresponds to a young adult, with no information regarding burial practices or archaeological context. Individual 2 is a middle-aged male, buried in a primary supine position, with the right arm extended, the left arm semi-flexed with the hand over the pelvis, and the lower limbs extended. The orientation was south (head) to north (feet). No artifacts that could be considered grave goods were found (Fabra, 2007; Fabra et al., 2014). Stable isotope analysis of $\delta 13C(col) = -15.2‰$ suggests a mixed diet, confirmed by archaeobotanical studies on dental calculus and ceramic materials (Fabra et al., 2009; Tavarone et al., 2023). Both individuals are housed in the Museum of Anthropology (FFyH, UNC).

<u>Isla Larga:</u> 1000-1500 CE

●**I42319** (I1, 75): 1000-1500 CE

The skeletal remains are housed at the San José de la Dormida Museum (Tulumba Department, Córdoba) and were recovered by amateurs, thus lacking more precise contextual information.

<u>Punta del Silencio:</u> 1214-1390 calCE

●**I34691** (932/18 I2, 171): 1214-1390 calCE (770±50 BP, LP-3624)

This open-air site is characterized by the discovery of human remains and archaeological materials (ceramics, underground fired-earth structures or ESTC) along the southern shore of Mar Chiquita Lagoon, in an area known as Punta del Silencio (San Justo Department, Córdoba, 30°53'15.3''S, 62°49'32.9''W). The exhumation was conducted by the Human Remains Recovery Team (ERARO, PJ, FFyH) and the Public Archaeology Program (SEU, FFyH, UNC) in 2018. Surveys identified four individuals in different areas of the beach, as well as 16 ESTC. For individual 1, lower limbs were found on the surface, not anatomically connected; individual 2, a young adult male, was partially buried in a dorsal position with the left arm semi-flexed over the chest and hyperflexed lower limbs to the right. Individual 3 included part of an upper limb; individual 4, a middle-aged female, was buried in a dorsal position with the skull oriented north and feet south, hands placed beside the body (Fabra et al., 2019). The remains are housed in the Archaeology Area, Cultural Heritage Directorate, Córdoba Culture Agency, Government of Córdoba Province.

Colonia Mackinlay: 1023-1151 calCE

●**I30888** (A911, CL3): 1023-1151 calCE (1010±25 BP, UGAMS-68797)

Colonia Mackinlay is a locality in the southern part of the Rivadavia Department, Santiago del Estero. To the east of this locality, in the marshes of Laguna de Mar Chiquita, within an area of grasslands and halophytic shrubs, amateurs discovered 33 human remains of an adult, 40 various fragments of rough ceramics, and a highly deteriorated metal ring, all of which appear to belong to the same context. The presence of *Heleobia parchappii* in the sediment from the skull of this individual, along with the condition of the exoskeletons of these gastropods, confirms the saline and water-rich environment of the find. Personnel from the former Emilio and Duncan Wagner Provincial Museum (now the Directorate General of Cultural Heritage) collected these remains from the departmental capital, Selva, and assigned the census identifiers A863 and A911. The sample (I30888) analyzed in this study comes from remains designated as A911.

Miramar: 991-1145 calCE

●**I19223** (200): 991-1145 calCE (1040±20 BP, UGAMS-68776)

These remains correspond to an adult female, currently housed in the Aníbal Montes Museum of Natural Sciences, Miramar, San Justo Department, Córdoba. No contextual information is available.

Colonia Müller: 436-580 calCE

●**I12754** (I1, 94): 436-580 calCE (1585±15 BP, UCIAMS-39102)

This is an open-air site located 50 m from the shore of Mar Chiquita Lagoon and 8 km from the city of Miramar (San Justo Department, Córdoba). The excavation was conducted in 2007 by the Rescue Archaeology Team (Museum of Anthropology, FFyH, UNC). The remains belong to a middle-aged adult female, buried in a simple primary burial in a supine position, with the lower limbs hyperflexed to the left, the right arm extended, and the left arm semi-flexed over the pelvic region. The body was oriented from east (head) to west (feet). The skull was displaced from its original anatomical position. No associated archaeological material was found (Fabra et al., 2009, 2014). The remains are housed in the "Aníbal Montes" Natural Sciences Museum of the Ansenuza Region (Miramar, Córdoba).

Estancia La Elisa/El Belga: 31-335 calCE

●**I12606** (I1S2, 142): 31-335 calCE (1890±49 BP, AA-102656)

This open-air site is located 150 m northwest of the Mar Chiquita lagoon (30°43'55"S,

62°59'75"W) (San Justo Department, Córdoba). Excavation was carried out in 2009 by members of the Rescue Archaeology Team (Museum of Anthropology, FFyH, UNC), at the request of the "Municipal Historical Museum of La Para". Bioanthropological analyses identified an adult male, approximately 33 to 42 years old, with an average age of 37.5 years at death. The remains were found in a simple individual primary burial, in a flexed left lateral decubitus position, body oriented south to north, with the skull facing west. Evidence suggests interpersonal violence related to the individual's death (Fabra et al., 2015). The remains are housed in the Archaeology Area, Cultural Heritage Directorate, Córdoba Culture Agency, Government of Córdoba Province.

Campo Bocassi/Agua Mansa: 98 calBCE - 243 calCE

●**I34464** (718/12, 135): 98 calBCE - 243 calCE (1980±70 BP, LP-3750)

This open-air site is defined by human remains discovered on private land (Río Primero Department, Córdoba Province, 30°42’40’’S, 63°01’49”W). The remains were found by locals, who informed the Municipal Museum of La Para, which then involved the Public Archaeology Team from the Museum of Anthropology (FFyH, UNC). The analysis identified a primary burial of an adult male, left lateral decubitus with semi-flexed limbs, oriented SW (skull) to NE (feet), with eye sockets facing west. Bioanthropological analysis indicated a male aged 50-59 years (Fabra et al., 2014). The remains are housed in the Archaeology Area, Cultural Heritage Directorate, Córdoba Culture Agency, Córdoba Provincial Government.

Playa Grande Marull: 200 BCE - 300 CE

●**I34471** (437/18 I2, 170): 139 calBCE - cal243 CE (1990±80 BP, LP-3741)
●**I34689** (437/18 I1, 189): 200 BCE - 300 CE

This open-air site was identified by the discovery of human remains along the southern shore of Mar Chiquita Lagoon (San Justo Department, Córdoba), specifically in the Playa Grande area (30°53'949"S, 62°47'487"W). The exhumation was carried out by members of the Human Remains Recovery Team (ERARO, PJ, FFyH) and the Public Archaeology Program (SEU, FFyH, UNC) in 2018. Dispersed ceramic materials were found along the coast. Bioanthropological analysis confirmed the presence of two female individuals: one young adult (individual 1) and one middle-aged adult (individual 2). No burial information is available (Fabra et al., 2019). The remains are housed in the Archaeology Area, Cultural Heritage Directorate, Córdoba Culture Agency, Government of Córdoba Province.

Rolo de Depetri: 765 - 187 calBCE

●**I19225** (1105/18, 172): 765-187 calBCE (2380±100 BP, LP-3625)

This open-air site, located on the southwestern shore of El Mistolar Island, Laguna Mar Chiquita, San Justo Department, Córdoba Province, is defined by the discovery of human remains on the surface (30°53'13.8''S, 62°56'38.9''W). The exhumation was carried out by members of the Human Remains Recovery Team (ERARO, PJ, FFyH) and the Public Archaeology Program (SEU, FFyH, UNC) in 2018. Anatomical parts were found dispersed over a 1-square-meter area (including the complete skull, jaw, femur, left and right tibia, ulna and radius, scapula, and vertebrae). Based on the degree of fusion, this was a subadult male, approximately 16-20 years old. Burial location and orientation could not be recorded, as the remains were scattered by water. Prospecting along the beach identified a total of 14 circular earth structures known as “hornitos,” common in regional archaeological sites. Additional finds included ceramics, lithic materials, and faunal remains, both modern and archaeological, with some of paleontological interest (Fabra et al., 2019). The remains from both sites are housed in the Archaeology Area, Cultural Heritage Directorate, Córdoba Culture Agency, Córdoba Provincial Government.

La Para: 798-418 calBCE

●**I34468** (1387/15, 158): 798-418 calBCE (2550±60 BP, LP-3754)

This open-air site is defined by human remains found on the surface along the shore of Mar Chiquita Lagoon, near the municipal campground in La Para (Río Primero Department, Córdoba, 30°55’6.90”S, 62°53’32.1”W). Exhumation was carried out by members of the Human Remains Recovery Team (ERARO, PJ, FFyH) and the Public Archaeology Program (SEU, FFyH, UNC) in 2015. Bioanthropological analysis identified a single young adult, aged 20-34 years, buried in a right lateral decubitus position, with hyperflexed legs to the right, the left arm flexed over the chest, and the right arm semi-flexed (Tavarone et al., 2016). The remains are housed in the Archaeology Area, Cultural Heritage Directorate, Córdoba Culture Agency, Córdoba Provincial Government.

Estancia La Elisa: 2874-1204 calBCE

●**I17032** (718/12 I1, 139): 1501-1204 BCE (3140±60 BP, LP-3720)
●**I13563** (718/12 I2, 140): 2874-2297 calBCE (4058±89 BP, AA-102655)

This open-air site is located on a private estate called "La Elisa" (Río Primero Department, Córdoba Province, 30°44.361’S, 62°56.628’W). The skeletal remains were found by locals who informed the Municipal Historical Museum of La Para, which then called upon the Public Archaeology Team from the Museum of Anthropology (FFyH, UNC). The inventory revealed two individuals: a possibly male juvenile (Individual 1) and an adult male (Individual 2). The excavation of the first area uncovered a simple primary burial, with a skeleton in a supine position, limbs extended (Individual 1). The second area revealed a simple primary burial, with a seated individual, limbs hyperflexed, and eye sockets facing downward (Individual 2, Fabra et al., 2014). The remains are housed in the Archaeology Area, Cultural Heritage Directorate, Córdoba Culture Agency, Government of Córdoba Province.

6.8 Argentina_Central_Hills_NorthCordoba

Cerro Colorado: 950-1300 CE

●**I12602** (54/III S4, 33): 1047-1218 calCE (935±20 BP, PSUAMS-6805)
●**I42327** (356/15, 194): 950-1300 CE

Human remains of individual I42327 were discovered at an open-air site located on a public street in Cerro Colorado (Tulumba Department, Córdoba) - 30°5'52.3''S, 63°55'51.4''W-. The recovery of the skeletal material, which had already been removed and placed on an artificial mound, was conducted by ERARO and the Public Archaeology Program in 2015. Excavation revealed a secondary burial that initially appeared to contain an adult individual. Subsequent bioanthropological analysis determined that the remains belonged to a subadult whose sex could not be identified.. These remains are in the process of being returned to the Comechingona Indigenous community of Cerro Colorado (Zabala et al., 2023) and are currently housed in the Archaeology Area, Cultural Heritage Directorate. The remains of individual I12602, identified as an adult female, are part of a collection housed in the Patrimonial Reserve of the Museum of Anthropology (FFyH, UNC).

Guayascate: 500-1500 CE

●**I42320** (G-A400, 76): 500 -1500 CE

The human remains are housed at the San José de la Dormida Museum, recovered by amateurs and thus lacking precise contextual or locational information. Individual G-A400 is an adult female.

6.9 Argentina_Central_Hills_Calamuchita

Loteo 5 Santa Rosa de Calamuchita: 1300-150 CE

●**I12724** (1435/12 I1, 151): 1326-1461 calCE (533±42 BP, AA-102659)
●**I12725** (1435/12 I2, 153): 1300-1500 CE

This is an open-air site located on private property in the town of Santa Rosa de Calamuchita (Calamuchita Department, Córdoba Province) -32°5’560’’S, 64°32’11.5’’W-. The skeletal remains were discovered by construction workers. The excavation was carried out by members of the Human Skeletal Remains Recovery Team (ERARO, PJ, FFyH) and the Public Archaeology Program (SEU, FFyH, UNC) in 2012. The inventory and minimum-number-of-individual assessment revealed the presence of skeletal remains belonging

to two adult female individuals, aged between 35 and 50 years (Individual 1) and between 33 and 42 years (Individual 2) at the time of death. Excavation of the area where the first remains were found uncovered a primary burial, with a skeleton (designated as Individual 1) in a right lateral recumbent position with the lower limbs hyperflexed and the right upper limb extended beneath the legs. The orientation of the skeleton was East (head) to West (feet), with the eye sockets facing Northeast. The sediment contained fragments of eggshells, pottery (base with net impressions), and one snail shell. Extending the excavation about 25 cm to the North revealed another skeleton (designated as Individual 2). This individual was buried in a primary burial, also in a right lateral recumbent position with hyperflexed lower limbs and flexed upper limbs between the legs. Its orientation was East (head) to West (feet), with the eye sockets facing downward. Associated materials included pottery fragments and unshaped lithics. One of the individuals exhibited a congenital developmental defect (Salega & Fabra, 2018). The remains are housed in the Archaeology Area, Cultural Heritage Directorate, Córdoba Culture Agency, Government of Córdoba Province.

Constantinopla 1215: 1299-1433 calCE

●**I34465** (2354/09, 149): 1299-1433 calCE (619±43 BP, AA-102658)

This open-air site was defined by human remains found during construction in a private yard in Villa Oviedo neighborhood, Alta Gracia (Santa María Department, Córdoba). Exhumation occurred in 2009 by the Rescue Archaeology Team from the Museum of Anthropology (FFyH, UNC). Since the remains were partially exhumed, it is estimated the individual was buried in a flexed left lateral decubitus position, with the right arm semi-flexed. Bioanthropological analysis confirmed a single adult male aged 19-45 years. The remains are housed in the Archaeology Area, Cultural Heritage Directorate, Córdoba Culture Agency, Córdoba Provincial Government.

Banda Meridional del Lago: 1289-1391 calCE

●**I40453** (I1, 70): 1289-1391 calCE (695±20 BP, UCI AMS 39104)

This open-air site was identified by the discovery of human skeletal remains on the opposite shore of the Río Tercero Reservoir from Villa Rumipal and the Atomic Plant of Embalse, between the Grande and Quillinzo rivers (Calamuchita Department, Córdoba). The exhumation was conducted by the Archaeology Area staff in 2006. Field records indicate it was a primary simple burial, with the individual flexed on the right side, positioned north to south, with the skull oriented west. A child inhumed in a ceramic vessel was found at the feet of this individual. Bioanthropological analysis identified the adult as female, within the middle-aged range (Fabra et al., 2009; Zarate et al., 2020). The remains are housed in the Archaeology Area, Cultural Heritage Directorate.

Despeñaderos: 1213-1394 calCE

●**I34466** (915/15, 165): 1213-1394 calCE (760±60 BP, LP-3874)

This is an open-air site identified by the discovery of human remains during construction work at a private residence in the city of Despeñaderos (Santa María Department, Córdoba, at coordinates 31°48'28.05"S, 64°17'42.96"W). During the exhumation, a single primary burial was identified, with the individual lying on their right side, legs hyperflexed over the chest. The remains are housed in the Archaeology Area, Cultural Heritage Directorate, Córdoba Culture Agency, Government of Córdoba Province.

Potrero de Garay: 691-1406 calCE

●**I40460** (E6/56, 36): 894-1406 calCE (881±150 BP, MTC-13215)
●**I17039** (E8/53, 34): 691-1383 calCE (995±161 BP, MTC-13246)

These remains, all associated with adult males exhibiting erect tabular cranial deformation, are stored in the Patrimonial Reserve of the Museum of Anthropology (FFyH, UNC). Isotopic analysis performed on these individuals yielded δ13C(col)= -10.5‰ and δ15N= 9.1‰ for I40460, and δ13C(col)= -15‰ and δ15N= 8.9‰ for I17039. These values suggest a mixed diet, with a higher C4 plant intake.

Los Molinos: 1045-1280 calCE

●**I17028** (E4/57, 116): 1045-1280 calCE (889±59 BP, MTC-14028)

These remains, all associated with adult males exhibiting erect tabular cranial deformation, are stored in the Patrimonial Reserve of the Museum of Anthropology (FFyH, UNC). Isotopic analysis performed on this individual yielded δ13C(col)= -11.4‰ and δ15N= 8.5‰, which associates with a mixed diet, with a higher C4 plant intake.

Potrerillo de Larreta: 775-1210 calCE

●**I13564** (422/11, 152): 775-1210 calCE (1067±77 BP, AA-102660)

This is an open-air site characterized by the discovery of human remains during excavation work for building a house in the gated community "Potrerillo de Larreta" in Alta Gracia, Santa María Department, Córdoba Province. The excavation was conducted by members of the Human Skeletal Remains Recovery Team (ERARO, PJ, FFyH) and the Public Archaeology Program (SEU, FFyH, UNC) in 2011. The bioanthropological analysis of the remains suggested that the individual was a young adult, approximately 24±5 years old at the time of death, buried in a right lateral recumbent position with hyperflexed legs. Nearby, faunal remains, lithic artifacts, and pottery fragments were recovered. Additionally, evidence of paleopathological lesions suggested

interpersonal violence (Fabra et al., 2015). The remains are housed in the Archaeology Area, Cultural Heritage Directorate, Córdoba Culture Agency, Government of Córdoba Province.

El Pariente/Campo Cannavo: 787-486 calBCE

●**I17031** (1141/11, 138): 787-486 calBCE (2540±25 BP, UGAMS-68770)

This open-air site is located on a riverbank along Route No. 5 in Villa Rumipal, on the road to Amancay, approximately 200 m west of the San Ignacio Bridge, Calamuchita Department, Córdoba Province (32°09'25.3"S, 64°30'32.6"W). The excavation was carried out in 2011 by members of the Human Skeletal Remains Recovery Team (ERARO, PJ, FFyH) and the Public Archaeology Program (SEU, FFyH, UNC). The human remains were recovered along with lithic material. The remains correspond to an adult male in a supine position with semi-flexed legs, the skull oriented northward, and lower limbs extending southward. Bioanthropological analysis confirmed that the remains belonged to a middle-aged adult male, between 20 and 35 years old at the time of death. The remains are housed in the Archaeology Area, Cultural Heritage Directorate, Córdoba Culture Agency, Government of Córdoba Province.

Club UBSISA/Amboy: 2466-1953 calBCE

●**I12601** (16): 2466-1953 calBCE (3810±90 BP, LP-3623)

The burial site is situated on the banks of the Río Calamuchita Reservoir (Río III Dam), approximately 5 km north of the Río Grande, near the towns of San Ignacio, Villa Amancay, and Amboy in the Calamuchita Department, Córdoba, Argentina. The remains were discovered accidentally by local residents in a ravine at a depth of 1.80 m. Various post-depositional processes led to the loss and deterioration of some anatomical pieces and the archaeological context. Bioarchaeological, genetic, malacological, and lithic analyses were performed (Fabra et al., 2023). The remains belong to an adult male, currently housed in the Dalmacio V. Sarsfield Museum, Amboy, Córdoba.

6.10 Argentina_Central_Hills_Comechingones

Quebrada del Zapato Bayo: 1321-1417 calCE

●**I19222** (46-145, 38): 1321-1417 calCE (610±20 BP, UGAMS-68775)

This site is a rock shelter located in the Sierra de la Estanzuela, in the Chacabuco department, San Luis province. It was a chance discovery. The skeleton belongs to an adult female individual.

Puerta del Tala: 5841-1898 calBCE

●**I19257** (RIV3): 1507-1649 calCE (330±20 BP, UGAMS-74310)
●**I19258** (RIV4): 687-881 calCE (1280±25 BP, UGAMS-74311)
●**I19259** (RIV5): 2269-1980 calBCE (3750±25 BP, UGAMS-74312)

Puerta del Tala is an open-air hunter-gatherer site, located 10 km southwest of Alpa Corral locality (Río Cuarto Department, Córdoba Province). Three individuals dating back up to 2,300 years BCE were included in this study.

6.11 Argentina_Central_Hills_SierrasChicas

Nunsacat: 1457-1633 calCE

●**I12713** (I2, 107): 1457-1633 calCE (387±41 BP, MTC-13250)

The human remains were recovered by road personnel in January 2004 and subsequently transferred to the Museum of Anthropology (FFyH, UNC). They were found at the Nunsacat site (Ischilín Department, Córdoba Province). The excavation was conducted by personnel from the Archaeology Area, Córdoba Culture Agency, S.E. The remains belong to two middle-aged adults, one female (Individual 1) and one male (Individual 2). There is no available information on the archaeological context or burial type for either individual. Isotopic information is available for Individual 2, with carbon (δ13C = -13.4‰) and nitrogen (δ15N = 8.5‰) values indicating a mixed diet. The remains are housed in the Museum of Anthropology (FFyH, UNC).

Ischilín: 1418-1623 calCE

●**I34467** (I1, 109): 1418-1623 calCE (459±40, MTC-13256)

The analyzed remains belong to a male individual, part of the Museum of Anthropology collection, though the specific location and archaeological context are unknown.

Alero Deodoro Roca/Ongamira: 2846 calBCE-1395 calCE

●**I12764** (ADR 60-60): 1296-1395 calCE (673±20 BP, YU-7740)
●**I12765** (ADR 60-98-1): 1028-1152 calCE (995±20; YU-7739)
●**I12766** (ADR 60-98-12): 1874-1629 calBCE (3457±20; YU-7738)
●**I12726** (Ue 166c): 2846-2473 calBCE (4097±22; YU-14910)

The Alero Deodoro Roca (ADR or DDR) site (30°46'28.506"S, 64°24'49.183"W) is located beneath a rockshelter formed by an outcrop of the Saldán Formation (Zárate, 2016) in the Ongamira Valley, at the northernmost region of the Sierras Chicas in the Ischilín Department, Córdoba. This amphitheatre-shaped formation, 100 meters wide, faces south and east. Montes

(1943) divided the rockshelter into two sectors (A and B), separated by a small semi-permanent waterfall that feeds a stream draining into the Ongamira River network. The site is situated at an elevation of approximately 1,200 meters above sea level. All radiocarbon dates for the site are from Izeta et al. (2021). Three individuals originate from Sector A, initially recovered during excavations conducted in the 1940s and 1950s by the late Aníbal Montes. These individuals were stored in the Museo de Antropología repository at the Universidad Nacional de Córdoba since the 1960s. In 2010, the museum began digitizing its collections, leading to the identification of many previously unknown items. Human remains were selected for various analyses, including radiocarbon dating, stable isotope analysis, and aDNA testing (two teeth from adult individuals and a vertebra from a juvenile). One sample (I12726), assigned to ADR Sector B, was recovered during a stratigraphic excavation. It corresponds to an adult exhumed in a primary position.

Deán Funes: 1226-1281 calCE

●**I34681** (1591/11, 179): 1226-1281 calCE (810±14 BP, MAMS-65758)

The skeletonized remains of this individual were discovered in 2011 close to the city of Deán Funes in the Ischilín Department, Córdoba Province. Regrettably, detailed contextual information is lacking. The remains are believed to belong to a young male who likely passed away between the ages of 15 and 20. The remains are currently curated in the Archaeology Area, Cultural Heritage Directorate, Córdoba Culture Agency, Government of Córdoba Province.

La Granja: 689-878 calCE

●**I17042** (LG98/I1, 42/67): 689-878 calCE (1280±20 BP, UCIAMS-22282)

This open-air site was identified by the discovery of human remains during construction work on a private home, located along the road connecting Ascochinga to Agua de Oro, in La Granja (Colón Department, Córdoba). There is no georeferenced information on the find. The skeletal remains were recovered by the Rescue Archaeology Team of the Anthropology Museum (FFyH, UNC) in 1998. Bioanthropological analyses identified a single middle-aged adult female, interred in a primary burial in a supine position with hyper-flexed lower limbs to the right and upper limbs flexed over the chest (Fabra et al., 2009). The remains are housed in the Patrimonial Reserve of the Anthropology Museum.

El Vado: 383 calBCE - 56 calCE

●**I12604** (I1, 104): 383 calBCE - 56 calCE (2156±86 BP, MTC-12808)

This is an open-air site defined by the presence of human remains found on a riverbank of the Santa Catalina River, 5 km from the homonymous estate, Sinsacate district (Totoral Department, Córdoba). The excavation was conducted by members of the Rescue Archaeology

Team (Museum of Anthropology, FFyH, UNC) in 2008. The remains belong to a middle-aged adult female, buried at a depth of 1.20 m in a simple primary burial, with the skull oriented to the south and eye sockets facing east, positioned in a right lateral decubitus posture, with semi-flexed arms over the chest and hyperflexed legs to the right (Zarate et al., 2021). She presented a developmental defect in the spine (Fabra & Salega, 2016). The remains are housed in the Museum of Anthropology (FFyH, UNC).

Paraje Los Tártagos: 50 BCE - 950 CE

●**I12720** (589/11, 132): 50 BCE - 950 CE

The human remains were discovered by chance at a bend in the Copacabana River, a few kilometers from the town of San Pedro de Toyos in the Ischilín Department, Córdoba Province, by local residents. The Second Judicial District Prosecutor's Office of the Tenth Judicial Circuit of Córdoba intervened. The excavation was carried out in 2011 by members of the Human Skeletal Remains Recovery Team (ERARO, PJ, FFyH) and the Public Archaeology Program (SEU, FFyH, UNC). According to information provided by locals, the remains were found in compact sediment associated with charcoal. However, access to the site was not possible, and the remains were handed over directly by local people. The remains correspond to an adult male individual, aged between 24 and 60 years. There is no contextual information regarding the find or the burial method. The remains are housed in the Archaeology Area, Cultural Heritage Directorate, Córdoba Culture Agency, Government of Córdoba Province.

Cementerio de Copacabana: 50 BCE - 950 CE

●**I12721** (C4, 1321): 50 BCE - 950 CE

The Cementerio site is located in the Copacabana Valley, in the margins of the Copacabana river. Excavations revealed, beneath an occupation by groups with ceramics, the existence of a much older, earlier occupation by hunter-gatherers. This occupation continued until a time prior to the conquest, with no recorded evidence of contact elements (Laguens & Bonnin, 2023). One individual was recovered from this site, which was included in this study.

Jesús María: 6641-6473 calBCE

●**I42080** (I2, SEC11): 6641-6473 calBCE (7765±35 BP, PSUAMS-9846)

The Jesús María site (30°59'48.60"S, 64°10'5.12"W) is located on the bluff of the La Cabaña River, west of the town of Jesús María (Colón Department, Córdoba Province). These bluffs were formed on a light-yellow loess terrace on the right bank of the La Cabaña River valley, west of Jesús María. The remains of three individuals were exhumed (Castellanos, 1957), found in varying states of preservation, semi-exposed in caves along the bluffs. No other archaeological

records were found. Stable isotope data (δ13C(col) = -15.5‰ and δ15N = 11.6‰) indicate a terrestrial diet that included herbivores consuming C3 and C4 photosynthetic pathway plants, likely guanacos and armadillos (Cornero et al., 2025).

6.12 Argentina_Central_Hills_NorthPunilla

Ayampitin/Valle Hermoso: 1323-1423 calCE

●**I12731** (I1, 17): 1323-1423 calCE (600±20 BP, UCIAMS-22287)

The human remains are currently stored at the Cap. J. de Zevallos Museum (located in Villa Hermoso, Córdoba). They were recovered by amateurs, hence there is no precise contextual or provenance information.

San Esteban: 1045-1179 calCE

●**I39331** (I1, 95): 1045-1179 calCE (965±15 BP, UCIAMS-39103)

This open-air site is located 500 meters from the Dolores stream, on its right bank, in San Esteban (Punilla Department, Córdoba). Recovery of the skeletal remains was requested by an individual who recognized human remains emerging from a cliff, formed along the railway line between Córdoba and Cruz del Eje. The skeletal material, already extracted and placed on an artificial mound, was recovered by the Rescue Archaeology Team of the Anthropology Museum in 2006. This was a primary simple burial, with the individual lying on the left side, legs hyper-flexed over the chest, and arms flexed between the legs and chest. Bioanthropological analysis identified a female individual between 15 and 20 years old (Fabra et al., 2009). The remains are housed in the Patrimonial Reserve of the Anthropology Museum.

6.13 Argentina_Central_Hills_SouthPunilla

Tejas 4 Malagueño: 1350-1700 CE

●**I40463** (321/21 I1, 177): 1448-1632 calCE (410±50 BP, LP-3797)
●**I39333** (809/21 I2, 191): 1350-1700 CE

This open-air site was identified by the appearance of human remains during construction at a private residence in the gated community of Tejas 4, in Malagueño (Santa María Department, Córdoba, 31°26’59.27"S, 64°26’1.95"W). In the case of individual 321/21, the remains were partially exhumed by workers. Recovery was later completed by the Human Remains Recovery Team (ERARO, PJ, FFyH) in 2021. It was a simple primary burial, with the individual lying on their back, head to the west and feet to the east. Individual 809/21 was found

nearby, but no burial or bioanthropological information is available for either individual. Both remain in the Archaeology Area, Cultural Heritage Directorate, Córdoba Culture Agency, Government of Córdoba Province.

Arroyo de la Palma 1/Copina: 1006 calBCE - 1398 calCE

●**I17041** (I2, 41): 1289-1398 calCE (680±40 BP, MTC-13248)
●**I40455** (I1, 115): 1006-596 calBCE (2707±61 BP, MTC 14027)

These remains were discovered in Copina, Punilla Department, Córdoba, in 1984. Individual 1 was buried in a dorsal position with legs hyperflexed over the torso, while Individual 2 was buried on their left side with legs semi-flexed towards the head. Isotopic analysis performed on these individuals yielded $\delta 13C(col)= -13.6\pm0.5‰$ and $\delta 15N= 9.3\pm1.4‰$ for I17041, and $\delta 13C(col)= -16.8\pm0.0‰$ and $\delta 15N= 8.3\pm0.7‰$ for I40455. These values suggest a mixed diet, with a higher C4 plant intake in later times (Sario et al., 2023). They are stored in the Patrimonial Reserve of the Museum of Anthropology (FFyH, UNC).

Cuesta Blanca: 896-1146 calCE

●**I40454** (I1, 40): 896-1146 calCE (1080±40 BP, MTC-13249)

The remains of this individual were recovered in Cuesta Blanca, Punilla Department, Córdoba, and identified as I1. There is no contextual information on the find, burial positions, or association with other archaeological materials. The remains are those of an adult male and are housed in the Patrimonial Reserve of the Anthropology Museum (FFyH, UNC).

Punilla Valley: 1000-1500 CE

●**I19234** (A1): 1000-1500 CE

The analyzed remains belong to a female individual, part of the Museum of Anthropology collection, though specific location and archaeological context are unknown.

Caaguazú: 441-665 calCE

●**I35514** (I1, 3GS, 192): 441-665 calCE (1510±50 BP, LP-3790)

Caaguazú (31°28'42.2''S, 64°34'16.3''W) is an open-air site located 300 m from the Icho Cruz River, in the Punilla Department, Córdoba province. Archaeological work revealed a burial on a street in the locality. The human remains found belong to an adult individual, with the skull, spine, and pelvis in a supine position, and the lower limbs in a right lateral flexed position. The orientation is west-east, at an average depth of 20 cm. The burial was bordered by large rocks,

some placed on the skeleton, and was associated with lithic materials, mainly grinding stones, along with some faunal bone fragments. A radiocarbon date of was obtained, corresponding to the transition between the early and late Late Holocene (Sario et al., 2023).

Ecoterra: 72-319 calCE

●**I17033** (I1,150): 72-319 calCE (1881±39 BP, AA104742)

This open-air site was discovered during construction work on a private property within the "Ecoterra" cabin complex in the commune of Cabalango (Punilla, Córdoba, 31°23'34"S, 64°32'24"W). Located 50 m from the La Salina stream and 100 m from the Los Chorillos stream, it is noteworthy that two fixed mortars were found about 20 m from the burial. Excavation was conducted in 2009 by the Rescue Archaeology Team (Museum of Anthropology, FFyH, UNC). The remains were found in a simple individual primary burial, in a flexed left lateral decubitus position, oriented north to south, with the skull facing east (Tavarone et al., 2020a). The remains are housed in the Museum of Anthropology (FFyH, UNC).

6.14 Argentina_Central_Hills_Traslasierra

Panaholma: 1456-1621 calCE

●**I17037** (I1, 26): 1456-1621 calCE (410±15 BP, PSUAMS-10210)

The remains of a single adult male individual were recovered by amateurs near Mina Clavero (San Alberto Department, Córdoba) in 1974. The individual shows tabular oblique fronto-lateral cranial deformation. The remains are currently housed in the Rocsen Multifaceted Private Museum in Nono.

Rosca Yaco: 1045-1480 calCE

●**I17043** (I1, 98): 1045-1480 calCE (705±131 BP, MTC-13252)

The remains of this individual were recovered in the town of San Carlos Minas (Minas Department, Córdoba) during a rescue archaeology operation in 2006 by the Córdoba Culture Agency. No detailed contextual information is available, except for photographs taken by police personnel showing that the remains were located in a rock shelter within a wooden box. The remains belong to a middle-aged adult male (Fabra, 2006). Stable isotope values of δ13C(col) = -14.8‰ and δ15N = 18.8‰ indicate a mixed diet. The remains are housed in the Museum of Anthropology (FFyH, UNC).

Guasmara: 1000-1250 CE

●**I12751** (I2, 35/66): 1053-1220 calCE (920±20 BP, UCIAMS-22281)
●**I12727** (I1, 167): 1000-1250 CE

This open-air site is defined by the appearance of human remains on the southern slope of a private field, beside the path linking Guasmara's spa with the Las Chacras River, known locally as "Camino al Pueblito" in Villa de las Rosas municipality (San Javier Department, Córdoba). The exhumation was conducted by the Rescue Archaeology Team (Museum of Anthropology, FFyH, UNC) in 1998. Two adult males were recovered. Individual 1 was buried in a fetal position, right lateral decubitus, with the skull facing east and arms crossed in front of the legs. Individual 2 was buried in a flexed left lateral decubitus position, arms flexed over the skull, again facing east (Fabra et al., 2009). One individual displayed a spinal developmental defect (Fabra & Salega, 2016). Both individuals are housed in the Museum of Anthropology (FFyH, UNC).

La Paz: 1046-1215 calCE

●**I34684** (1166/15, 185): 1046-1215 calCE (940±20 BP, UGAMS-68822)

The site is located in La Paz (San Javier Department), in a private cabin complex (32°13'134"S, 65°02'404"W). The skeletal remains appeared during excavations for the installation of electrical wiring, approximately 50 cm below the current ground level. Archaeological recovery work identified a primary simple burial of an individual in a left-side lying position, oriented southwest-northwest, with the upper limbs flexed and the lower limbs hyper-flexed. Bioanthropological analysis indicates that this was an adult male.

Loma Bola: 900-1350 CE

●**I12718** (I1A, 105): 992-1274 calCE (954±85 BP, MTC-12806)
●**I12729** (I1B, 169): 900-1350 CE

This is an open-air site defined by three funerary structures, approximately 10 m² in size. The site is located south of the Los Talas stream, in the locality of Loma Bola (San Javier Department, Córdoba). The excavation was conducted by members of the Rescue Archaeology Team (Museum of Anthropology, FFyH, UNC) in 2007. The bioanthropological analysis of the remains identified the presence of three individuals: an adult male, an adult female, and a juvenile, designated as Individual 1A, Individual 1B, and Individual 2A (juvenile). Information on the burial characteristics can only be inferred for Individual 1B, as the others (1A and 2A) were removed by personnel who did not document the findings. Individual 1B was buried in a right lateral decubitus position, with hyperflexed lower limbs and the right upper limb encircling the legs (Fabra & Gonzalez, 2009). One of the individuals exhibits a congenital defect (Salega & Fabra, 2018). The remains are housed in the Archaeology Area, Cultural Heritage Directorate, Córdoba Culture Agency, Government of the Province of Córdoba.

<u>Paraje Los Molles:</u> 1000-1400 CE

●**I34470** (1222/16, 159): 1000-1400 CE

The archaeological rescue took place in October 2016 by members of ERARO at the site known as "Paraje Los Molles", located in Villa de Las Rosas, San Javier Department (31°57’40.43”S, 65°0’49.24”W). The excavation, conducted in 2012, was performed by members of the Human Skeletal Remains Recovery Team (ERARO, PJ, FFyH) and the Public Archaeology Program (SEU, FFyH, UNC). The site, on private land, contained human skeletal remains found along an internal path. Due to climatic and environmental conditions, a gully exposed part of a skull. Following its identification, an excavation revealed a simple primary burial in a right lateral decubitus position, with the lower limb hyperflexed, oriented North-South, in very poor condition (Ginarte, 2016 MS). Laboratory analyses identified it as a young adult (20-34 years), female. The remains are housed in the Archaeology Area, Cultural Heritage Directorate, Córdoba Culture Agency, Government of Córdoba Province.

<u>Las Caleras:</u> 1000-1400 CE

●**I34685** (1057/20, 176): 1000-1400 CE

This open-air site is located in Villa de las Rosas (San Javier Department, Córdoba). The exhumation, carried out by the Human Remains Recovery Team (ERARO, PJ, FFyH) in 2020, indicates a secondary burial, likely a bundled burial. No bioanthropological analysis has yet been conducted. The remains are housed in the Archaeology Area, Cultural Heritage Directorate, Córdoba Culture Agency, Government of Córdoba Province.

<u>Paraje La Cuesta:</u> 772-987 calCE

●**I34688** (175/15, 184): 772-987 calCE (1200±25 BP, UGAMS-68823)

This is an open-air site identified by the appearance of human remains on a rural road connected to Provincial Route No. 15, 3 km from the town of Cura Brochero (San Alberto Department, Córdoba) - 31°40'959''S, 65°06´621'W-. The recovery work on the skeletal material, already extracted and placed on an artificial mound, was carried out by members of the Human Remains Recovery Team (ERARO, PJ, FFyH) and the Public Archaeology Program (SEU, FFyH, UNC) in 2015. The excavation work identified a primary simple burial, with the individual positioned on their right side, with hyper-flexed upper and lower limbs over the chest. Bioanthropological analyses estimated the individual as an adult male. The remains are housed in the Archaeology Area, Cultural Heritage Directorate, Córdoba Culture Agency, Government of Córdoba Province.

<u>San Sebastián:</u> 755-407 calBCE

I17038 (I3, 29): 755-407 calBCE (2480±25 BP, UGAMS-68772)

The remains of an adult male, identified as I3, were discovered near Mina Clavero (San Alberto Department, Córdoba) by amateurs. No contextual information is available. The remains are housed at the Comechingon Private Museum in the same locality.

Alem Villa de Soto: 800-300 BCE

●**I40452** (589/18, 187): 800-300 BCE

This open-air site was identified by the discovery of human remains during construction work on a private residence in the 25 de Mayo neighborhood, Villa de Soto (Cruz del Eje Department, Córdoba) - 30°51’25’’S, 64°59'806"W-. The skeletal material, already extracted and placed on an artificial mound, was recovered by members of ERARO and the Public Archaeology Program in 2018. Excavation identified a single individual in a primary burial, left-side lying. There is no bioanthropological information regarding the age or sex of the individual. The remains are housed in the Archaeology Area, Cultural Heritage Directorate, Córdoba Culture Agency.

Los Mates: 800-300 BCE

●**I34686** (497/20, 188): 800-300 BCE

This open-air site was defined by the appearance of human remains on an unnamed public street in Los Mates (La Paz, San Javier Department, Córdoba). The human remains were located along a dirt road and discovered when a leveling machine was performing road improvements. The skeletal material, already extracted and placed on an artificial mound, was recovered by ERARO members in 2020. The adult individual, possibly male, was positioned lying on the left side with flexed upper limbs over the chest. The remains are housed in the Archaeology Area, Cultural Heritage Directorate.

6.15 Argentina_Central_Hills_Occidentales

La Yesera: 26-196 calCE

●**I19221** (IY4, 23): 26-196 calCE (1960±20 BP, UGAMS-68774)

The site is located between the towns of La Calera and Desaguadero, in the Belgrano department, San Luis province. The skeleton is housed in the Municipal Historical Museum of San Luis, but there is no precise information about the discovery context.

References

Acuto, F. (2007). Fragmentación vs. integración comunal: Repensando el Período Tardío del Noroeste Argentino. *Estudios Atacameños, 34*, 71-95.

Agnolin, A., & Carbonelli, J. P. (2017). Diseños de puntas de proyectil en el valle de Santa María (Catamarca, Argentina): Una aproximación a la ocupación cazadora-recolectora. *Chungara. Revista de Antropología Chilena, 49*(4), 511-527.

Albeck, M. E., Basso, D. M., Tolaba, J. L., & Zaburlín, M. A. (2018). Aportes a la historia ocupacional de Pueblo Viejo de Tucute, Puna de Jujuy. *Estudios Sociales del NOA, 21*, 85-112.

Albeck, M. (1994). La Quebrada de Humahuaca en el intercambio prehispánico. En M. Albeck (Ed.), *Taller de costa a selva. Producción e intercambio entre los pueblos agroalfareros de los Andes Centro Sur* (pp. 117-127). Instituto Interdisciplinario Tilcara, Universidad de Buenos Aires.

Albeck, M. (2010). Pueblo Viejo de Tucute. Una sociedad interpretada a través de la construcción de espacio. En M. E. Albeck, M. C. Scattolin, & M. A. Korstanje (Eds.), *El hábitat prehispánico: Arqueología de la arquitectura y de la construcción del espacio organizado*. EdiUNJu.

Albeck, M. (2001). La Puna Argentina en los Períodos Medio y Tardío. En E. Berberian & A. Nielsen (Eds.), *Historia argentina prehispánica* (Tomo I, pp. 347-388). Editorial Brujas.

Albeck, M. E., & Zaburlín, M. A. (2008). Aportes a la cronología de los asentamientos agropastoriles de la Puna de Jujuy. *Relaciones de la Sociedad Argentina de Antropología, XXXIII*, 155-186.

Albeck, M., & Ruiz, M. (1997). Casabindo: Las sociedades del período tardío y su vinculación con las áreas aledañas. *Revista de Arqueología*.

Aldazabal, V. (2004). *La ocupación humana en el sector centro oriental de la Pampa Deprimida, Provincia de Buenos Aires, Argentina* [Tesis doctoral inédita]. Facultad de Filosofía y Letras, Universidad de Buenos Aires.

Aldazabal, V., & Cáceres, L. (1997). Primeras observaciones en el sitio La Colorada, Pdo. de Rauch, Bs. As. *Congreso Nacional de Arqueología Argentina, t.1*, 95-103. La Plata.

Aldazabal, V., Eugenio, E., & García Laborde, P. (2020). Restos humanos en la costa marina del Partido de Mar Chiquita, Provincia de Buenos Aires. *Revista Argentina de Antropología Biológica, 22*(2), 11-12. https://doi.org/10.24215/18536387e022

Alfaro de Lanzone, L. (1988). Investigación arqueológica en la Cuenca del río Doncellas. Dto. Cochinoca, Jujuy. Reconstrucción de una cultura olvidada en la Puna Jujeña. Departamento de Antropología y Folklore, Ed. Argentina.

Alfaro de Lanzone, L., & Suetta, J. (1970). Nuevos aportes para el estudio del asentamiento humano en la Puna de Jujuy. Revisión del Pucará de Rinconada. *Antiquitas, X*, 1-10.

Alfonso Monges, M., & Lamenza, G. (2021). Periodo prehispánico. *Historia definitiva de Paraguay*. Goya.

Álvarez, M. C. (2014). Tecnología ósea en el Oeste de la región pampeana: Identificación de las técnicas de manufactura a partir de evidencias arqueológicas y experimentales. *Chungará, 46*(2), 193-210.

Angiorama, C., & Taboada, C. (2008). Metales andinos en la llanura santiagueña (Argentina). *Revista Andina, 4*, 117-150.

Apolinaire, E., & Bastourre, L. (2016). Nets and canoes: A network approach to the pre-Hispanic settlement system in the Upper Delta of the Paraná River (Argentina). *Journal of Anthropological Archaeology, 44*, 56-68.

Arellano, J. A. (2014). El Chaco boliviano: Del Paleoindio al período alfarero tardío. *Folia Histórica del Nordeste, 22*, 147-168.

Arrieta, M. A., Bordach, M. de la A., & Mendonça, O. J. (2011). Tuberculosis precolombina en el noroeste argentino (NOA). El cementerio de Rincón Chico 21 (RCH 21), Santa María, Catamarca. *Intersecciones en Antropología, 12*, 245-260.

Aschero, C. A. (1984). El sitio ICC4: Un asentamiento precerámico en la Quebrada de Inca Cueva (Jujuy, Argentina). *Estudios Atacameños, 7*, 62-72.

Aschero, C. A. (1996). Arte y arqueología. Una visión desde la puna argentina. *Chungara, 28*(1-2), 175-197.

Aschero, C. A., & Hocsman, S. (2011). Arqueología de las ocupaciones cazadoras-recolectoras. *Revista de Arqueología*.

Babot, M. P. (2011). Cazadores-recolectores de los Andes Centro-Sur y procesamiento vegetal. Una discusión desde la Puna Meridional Argentina (ca. 7.000-3.200 años A.P.). *Chungara. Revista de Antropología Chilena, 43*(1), 413-432.

Babot, M. P., & Martel, A. R. (2025). Marks on the floor. Instant and memory in the foundation of an agro-pastoralist place in the high desert of Argentinian Northwest (ca. 1500 BP). *Journal of Anthropological Archaeology, 77*, 101657.

Babot, M. P., Gonzalez Baroni, L. G., & Becerra, M. F. (2023). Objetos de cobre arsenical y sociedades agropastoriles en la Puna meridional argentina. Nuevas perspectivas sobre la metalurgia del segundo milenio AP en los Andes Centro-Sur. *Latin American Antiquity, 34*(2), 295-313.

Babot, M. P., Gonzalez Baroni, L. G., Urquiza, S. V., Aguirre, M. G., Colaneri, M. G., Hocsman, S., & Haros, M. C. (2009). Dinámicas de formación y transformación de un entierro en el desierto puneño (Antofagasta de la Sierra, Puna Meridional Argentina). *Intersecciones en Antropología, 10*, 183-201.

Baffi, E. I., & Torres, M. F. (1996). Los restos óseos humanos del sitio Martínez 4 (Ambato, Catamarca). *Publicaciones del CIFFyH, Arqueología, 48*, 55-63.

Barboza, M. C. (2014). Análisis arqueofaunístico del sitio Paso del Tala (Goya, Corrientes, Argentina). *Revista del Museo de Antropología, 7*(2), 219-226.

Barrientos, G., & Perez, S. I. (2005). Was there a population replacement during the late mid-Holocene in the Southeastern Pampas of Argentina? Archaeological evidence and paleoecological basis. *Quaternary International, 132*, 95-105.

Barrientos, G., & Masse, W. B. (2014). The archaeology of cosmic impact: Lessons from two mid-Holocene Argentine case studies. *Journal of Archaeological Method and Theory, 21*, 134-211.

Barrientos, G. (2009). El estudio arqueológico de la continuidad/discontinuidad biocultural: El caso del sudeste de la Región Pampeana. En R. Barberena, K. Borrazzo, & L. A. Borrero (Eds.), *Perspectivas actuales en arqueología argentina* (pp. 189-214). CONICET-IMHICIHU.

Basso, D. M. (2021). Estudios sobre cambios y continuidades entre el periodo prehispánico y la etapa colonial en la localidad de Cochinoca (Puna de Jujuy). A la memoria de María Ester Albeck. *Mundo De Antes, 15*(1), 103-134. https://doi.org/10.59516/mda.v15.224

Bastourre, M. (2022). Las relaciones humanos/animales en las sociedades prehispánicas del Paraná Medio e Inferior: Un giro ontológico. *Revista del Museo de Antropología, 14*(2), 65-80.

Bastourre, M. L., & Azpelicueta, M. M. (2020). Del registro ictioarqueológico a las prácticas alimentarias: El caso de Los Tres Cerros 1 (Delta Superior del Paraná, Entre Ríos). *Relaciones de la Sociedad Argentina de Antropología, XLV*(1), 13-57.

Bayón, C., & Flegenheimer, N. (1998). Un ejercicio de aplicación: Procedencia de rocas en el sitio El Guanaco. Trabajo presentado en el I Congreso de Arqueología de la Región Pampeana Argentina, Santa Fe.

Bayón, C., Flegenheimer, N., Zárate, M., & Deschamps, C. (2004). "...Y vendrán los arqueólogos en busca de un hueso"... Sitio El Guanaco, partido de San Cayetano. En G. Martínez, M. Gutiérrez, R. Curtoni, M. Beron, & P. Madrid (Eds.), *Aproximaciones Arqueológicas Pampeanas: Teorías, Métodos y Casos de Aplicación Contemporáneos* (pp. 249-258). INCUAPA-UNCPBA.

Bernal, V. (2008). Procesos de diferenciación biológica entre poblaciones humanas del Holoceno tardío de Patagonia: Una aproximación desde la variación métrica dental [Tesis doctoral inédita]. Facultad de Ciencias Naturales y Museo, Universidad Nacional de La Plata, La Plata, Argentina.

Berón, M. (2004). Dinámica poblacional y estrategias de subsistencia de poblaciones prehispánicas de la cuenca Atuel-Salado-Chadileuvú-Curacó, provincia de La Pampa [Tesis doctoral]. Facultad de Filosofía y Letras, Universidad de Buenos Aires, Buenos Aires.

Bleiler, E. (1948). The East. En Bennett, Bleiler, & Sommer (Eds.), *Northwest Argentine Archaeology* (pp. 120-139). Yale University Publications in Anthropology 38.

Bonnin, M. I., & Laguens, A. G. (1996). Evaluación de series de fechados radiocarbónicos del Valle de Ambato, Catamarca. *Publicaciones del CIFFyH, Arqueología, 48*, 65-101.

Bonomo, M. (2013). Reanálisis de la colección de Samuel Lothrop procedente del Delta del Paraná. *Relaciones de la Sociedad Argentina de Antropología, 38*(1), 169-198.

Bonomo, M., & Politis, G. (2020). Mound building, social complexity and horticulture in the Lower Paraná River. En C. Smith (Ed.), *Encyclopedia of Global Archaeology* (pp. 1-20). Springer International Publishers.

Bonomo, M., & Ramos, S. (2023). Study of dugout canoes from the coast of La Plata river and the islands of the Paraná Delta, Argentina. *Journal of Island & Coastal Archaeology, 18*(1), 75-99.

Bonomo, M., Angrizani, R., Apolinaire, E., & Noelli, F. (2015). A model for the Guaraní expansion in the La Plata Basin and Litoral Zone of Southern Brazil. *Quaternary International, 356*, 54-73.

Bonomo, M., Capdepont, I., & Matarrese, A. (2009). Alcances en el estudio de colecciones. Los materiales arqueológicos del Delta del río Paraná depositados en el Museo de La Plata (Argentina). *Revista de Arqueología Sudamericana, 5*, 68-101.

Bonomo, M., Cabanillas, E., & Montero, R. (2017). Archaeometallurgy in the Paraná Delta (Argentina): Composition, manufacture, and indigenous routes. *Journal of Anthropological Archaeology, 47*, 1-11.

Bonomo, M., Aceituno, F., Politis, G., & Pochetino, M. A. (2011). Pre-Hispanic horticulture in the Paraná Delta (Argentina): Archaeological and historical evidence. *World Archaeology, 43*(4), 557-579.

Bonomo, M., Politis, G., & Gianotti, C. (2011). Montículos, jerarquía social y horticultura en las sociedades indígenas del Delta del río Paraná (Argentina). *Latin American Antiquity, 22*(3), 297-333.

Bonomo, M., Politis, G., Bastourre, L., & Moreira, G. (2021). Humanized nature: Symbolic representation of fauna in pottery from the Paraná River of South America. En M. Bonomo & S. Archila (Eds.), *South American Contributions to World Archaeology* (pp. 411-446). Springer.

Bonomo, M., Politis, G., & Castro, J. C. (2010). Primeros resultados de las investigaciones arqueológicas en el Delta Superior del Paraná y su contribución al atlas arqueológico de la provincia de Entre Ríos. *Folia Histórica del Nordeste, 18*, 33-58.

Bonomo, M., Politis, G., Silva, C., Bastourre, L., Ramos van Raap, M. A., Castiñeira Latorre, C., Scabuzzo, C., & Apolinaire, E. (2017). Estado actual de las investigaciones en la localidad arqueológica Laguna de los Gansos (Diamante, Entre Ríos). *Revista del Museo de Antropología, 9*(2), 51-66.

Bonomo, M., Ramos van Raap, M. A., Cenizo, M., Del Papa, M., Alvarez, M. C., Bonnat, G. F., & Isla, F. (2024). Nuevas investigaciones del túmulo de Malacara (partido de Lobería, provincia de Buenos Aires). Enviado a *Comechingonia. Revista de Arqueología*.

Bonomo, M., Scabuzzo, C., Politis, G., & Zucol, A. (2017). Stable carbon and nitrogen isotope studies in the Paraná River Delta (Argentina): An approach to prehispanic diets. *Latin American Antiquity, 28*(1), 105-126.

Buc, N. (2010). Tecnología ósea de cazadores-recolectores del humedal del Paraná inferior (Bajíos Ribereños Meridionales). En D. Loponte & A. Acosta (Eds.), *Series Monográficas, Arqueología de la Cuenca del Plata*. Buenos Aires: Instituto Nacional de Antropología y Pensamiento Latinoamericano.

Cabrera, A., & Willink, A. (1973). *Biogeografía de América Latina*. Serie Biología. Monografía 13, 122 pág. OEA.

Caggiano, M. A. (1984). Prehistoria del noreste Argentino, sus vinculaciones con la República Oriental del Uruguay y sur de Brasil. *Pesquisas, Antropología, 38*, 1-109.

Calandra, H., Lamenza, G., & Salceda, S. (2012). Arqueología pública y pueblos originarios en el Chaco Central: Ancestría e identidad. En Maeder et al. (Coords.), *Estudios y Contribuciones: Homenaje a la doctora Norma Cristina Meichtry* (pp. 45-70). Resistencia: Contexto Libros.

Canova, R., Salega, S., Valenzuela, L., & Fabra, M. (2020). "La Viajera": Aproximaciones osteobiográficas a la historia de vida de una mujer que habitó la costa sur de la Laguna Mar Chiquita (noroeste de la región pampeana, Córdoba, Argentina). *Boletín de Antioquia, 35*(60), 73-99.

Canova, R., Salega, S., & Fabra, M. (2024). Reconstrucción de las historias de vida de dos personas que habitaron la costa sur de la Laguna Mar Chiquita (norte de la región pampeana, Córdoba, Argentina). *Intersecciones en Antropología*. En prensa.

Carden, N., & Leon, C. (2025). Entre montes, arroyos y cerros: Una aproximación comparativa a la diversidad morfológica, técnica y espacial de los grabados rupestres del sur de Santiago del Estero, Argentina. *Arqueología, 31*(1), 14376.

Castellanos, A. (1957). Consideraciones acerca del hallazgo efectuado por Florentino Ameghino de restos humanos fósiles en el cordobense de la ciudad de Córdoba a propósito de nuevos descubrimientos en Jesús María (prov. de Córdoba). *Ameghiniana, I*(3), 32-36.

Castiñeira, C., Blasi, A., Politis, G., Bonomo, M., Del Puerto, L., Huarte, R., Carbonari, J., Mari, F., & García Rodriguez, F. (2013). Origin and construction of mounds in the Upper Paraná Delta Wetlands (Argentina). *Archaeological and Anthropological Sciences, 5*(1), 37-57.

Castiñeira, C., Blasi, A., Bonomo, M., Politis, G., & Apolinaire, E. (2014). Modificación antrópica del paisaje durante el Holoceno tardío: Las construcciones monticulares en el Delta Superior del río Paraná, Argentina. *Revista de la Asociación Geológica Argentina, 71*(1), 33-47.

Castro, J. C., Bonomo, M., González Venanzi, L., & Cornero, S. (2020). Perros indígenas en el Nordeste argentino. *Latin American Antiquity, 31*(4), 853-870.

Castro, J. C. (2016). Late-Holocene indigenous occupation of the Uruguay river (Argentina). *DIGit. Journal of the Flinders Archaeological Society, 3*, 42-53.

Cattáneo, G. R., & Izeta, A. D. (2023). Reflexiones al pie de las sierras: De datos y modelos sobre el poblamiento humano del Centro de Argentina. En M. Nuñez Camelino, M. C. Barboza, C. Píccoli, M. V. Roca, & C. Scabuzzo (Eds.), *Libro de resúmenes XXI Congreso Nacional de Arqueología Argentina* (p. 663). http://congresoscnaa.org/congreso/wp-content/uploads/2023/08/Libro-de-Resumenes-XXI-CNAA.pdf

Cattáneo, R., & Izeta, A. D. (Eds.). (2016). *Arqueología en el Valle de Ongamira, 2010-2015* (1st ed.). Universidad Nacional de Córdoba. http://suquia.ffyh.unc.edu.ar/handle/suquia/495

Cattáneo, R., Izeta, A. D., & Caminoa, J. M. (2016). A Fishtail Projectile Point from the Southern Pampean Hills, Characato, Córdoba, Argentina. https://doi.org/10.1080/20555563.2016.1200348

Cattáneo, R., Izeta, A. D., & Costa, T. (2015). El patrimonio arqueológico de los espacios rurales de la provincia de Córdoba. Museo de Antropología-IDACOR. https://suquia.ffyh.unc.edu.ar/handle/suquia/526

Cattáneo, R., Robledo, A. I., Martinelli, M., Brizuela, C. F., & Izeta, A. D. (2022). Late Holocene triangular lithic projectile points, their morphometric variability and hafting systems in the Southern Pampean Hills (Córdoba, Argentina). *Journal of Archaeological Science: Reports, 42*, 103359. https://doi.org/10.1016/j.jasrep.2022.103359

Ceruti, C. (1988). Modificación ambiental y adaptación cultural en la cuenca del Paraná Medio. *Actas IX Congreso Nacional de Arqueología Argentina*, Buenos Aires.

Ceruti, C. (2003). Entidades culturales presentes en la Cuenca del Paraná Medio. *Revista Mundo de Antes, 3*, 111-135.

Ceruti, C. N. (2013). Indicios de complejidad social en un enterratorio de la entidad cultural arqueológica Goya-Malabrigo. Hernandarias, Dpto. Paraná, provincia de Entre Ríos. Paper presented at the V Encuentro de Discusión Arqueológica del Noreste Argentino, Goya, August 14-16, 2013.

Cohen, M. (2005). Entre guano y arena… Ocupaciones recurrentes: Un caso de estudio en el sitio Punta de la Peña 9 III, Antofagasta de la Sierra, Catamarca [Tesis de licenciatura]. Facultad de Ciencias Naturales e IML, Universidad Nacional de Tucumán, San Miguel de Tucumán.

Cohen, M., & Armelagos, G. (1984). *Paleopathology at the origins of agriculture*. Academic Press. Orlando.

Cohen, M. L. (2010). Prácticas sociales, estrategias de visibilidad y construcción de la cartografía social durante el lapso ca. 1000-1500 AD en Antofagasta de la Sierra, Catamarca. Perspectivas desde el sitio Peñas Coloradas 3 Cumbre [Tesis doctoral]. Facultad de Filosofía y Letras, Universidad de Buenos Aires, Buenos Aires.

Colazo, S., Méndez, M. G., Calandra, H. A., Ferrarini, S. O., & Salceda, S. A. (2002). Estudio preliminar del sitio arqueológico "El Chancho", Departamento de San Fernando, Provincia del Chaco. En *Actas del XXI Encuentro de Geohistoria Regional* (pp. 68-74).

Colobig, M. M., & Ottalagano, F. V. (2016). Estudio arqueobotánico de los residuos orgánicos adheridos en alfarerías prehispánicas de la cuenca del Paraná medio. *Arqueología, 22*(1), 193-210.

Colobig, M. M., Sánchez, J. O., & Zucol, A. (2015). Análisis de macrorestos vegetales en el sitio arqueológico Los Tres Cerros 1 (isla Las Moras, Victoria, Entre Ríos). *Revista del Museo de Antropología, 8*(1), 115-124.

Combès, I. (2008). La mala fe potorera. Apóstatas, donecillos y dinámicas étnicas en Chiquitos. *Campos, 9*(2), 23-41.

Cornero, S. (2016). Sitio La Lechuza: Aportes para la investigación arqueológica del norte de Santa Fe. *Actas XIX Congreso Nacional de Arqueología*, Tucumán. Serie Monográfica y Didáctica Vol. 54, 480-484.

Cornero, S., Rivero, D., Politis, G., & Capriles, J. M. (2025). Nuevas dataciones sobre restos humanos del centro de Argentina y su aporte a la discusión del poblamiento de la región. *Revista Arqueología, 31*.

Cornero, S. (1999). Enterratorios humanos en el litoral: Sitio La Lechuza, Alejandra, Santa Fe. *Actas XII Congreso Nacional de Arqueología Argentina*, UNLP. Tomo II, 384-388. La Plata.

Cornero, S. (2009). Apuntes de arqueología de islas. Sitio El Castaño, boca de la Milonga, río Paraná. *Anuario de Arqueología, 1*(1), 153-160.

Cornero, S. (2014). El Camping. Rescate y cronologías para un sitio Goya-Malabrigo tardío. Alejandra, Santa Fe. *7° Jornadas Ciencia y Tecnología*, UNRosario, 507-511.

Cornero, S. (2014). La Balsa de los Confines. Arqueología de la isla Los Marinos, Bajo Paraná. Edad Radiocarbónica Convencional: LP-2132. LaTyR UNLP Colección Castellanos. Informe Proyecto Secyt ING483. Universidad Nacional de Rosario.

Cornero, S. (2018). En las puertas del mito: Loros y peces en el arte cerámico de la costa del río Paraná. En G. Politis & M. Bonomo (Eds.), *Goya-Malabrigo: Arqueología de una sociedad indígena del Noreste argentino* (pp. 89-106). UNICEN, Tandil.

Cornero, S., & Ragnone, L. (2015). Análisis arqueobotánicos en sitios de la entidad arqueológica Goya-Malabrigo ubicados en el centro-norte de Santa Fe. *Anuario de Arqueología, 7*, 85-94.

Cornero, S. E., & Green, A. G. (2022). Los indígenas Corondas del Litoral Bajo Paranaense y su señor. Un caso de liderazgo en el siglo XVI. *Sociedades De Paisajes Áridos y Semiáridos, 4*(1), 10-55.

Cornero, S., & Tissera, L. E. (2024). Arqueología de largas distancias y circulación de objetos e ideas en la región pampeana argentina. *Sociedades De Paisajes Áridos y Semiáridos, 18*(1), 64-86.

Cornero, S., Rivero, D., Politis, G., & Capriles, J. M. (2025). Nuevas dataciones sobre restos humanos del centro de Argentina y su aporte a la discusión del poblamiento de la región . Arqueología, 31(2), 15240. https://doi.org/10.34096/arqueologia.t31.n2.15240

Cornero, S., Solomita, F., & Curetti, P. (2007). Componente arqueofaunístico del sitio La Lechuza (provincia de Santa Fe). En F. Oliva, N. de Grandis, & J. Rodríguez (Eds.), *Arqueología Argentina en los inicios de un nuevo siglo* (pp. 167-171). Rosario: Escuela de Antropología.

Costa Angrizani, R. C., Colobig, M. M., & Bonomo, M. (2021). Taxonomia funcional e análise de microvestígios botânicos em vasilhas arqueológicas guarani na Argentina. *Habitus, 18*(2), 420-448.

Cruz, P. (2004). Archéologie de la mort dans la Vallée d'Ambato. Homme et milieu dans le Bassin de Los Puestos (Catamarca-Argentine) durant la Période d'Intégration Régionale (IV siècles après J.-C.) [Tesis doctoral, Universidad de Paris I Pantheon Sorbonne].

Crouzeilles, A. L. de. (1939). Correlaciones entre la alfarería indígena encontrada en la región de Santa Fe y la de la provincia de Santiago del Estero. *Anales de la Sociedad Científica Argentina*, 128, 196-211.

Cruz, P. (2006). La muerte y sus manifestaciones en el valle de Ambato (Cuenca de Los Puestos, Catamarca, Argentina). En M. A. Costa & A. Llagostera (Eds.), *La Cultura de la Aguada y su dispersión. IV Mesa Redonda* (pp. 43-51). Universidad Católica del Norte.

Cruz, P. J. (2007). Hombres complejos y señores simples. Reflexiones en torno a los modelos de organización social desde la arqueología del valle de Ambato (Catamarca). En A. Nielsen, M. Rivolta, V. Seldes, M. Vázquez, & P. Mercolli (Comps.), *Procesos Sociales Prehispánicos en los Andes Meridionales* (pp. 99-123). Editorial Brujas, Córdoba.

Da Peña Aldao, G., Fabra, M., & Novellino, P. (2024). Primeros abordajes sobre patologías infecciosas en tejido óseo de poblaciones arqueológicas del Centro Oeste de Argentina. *Revista Argentina de Antropología Biológica*.

Dantas, M., Bachmeier, A., Villafañez, E., & Figueroa, G. G. (2019). Prospección arqueológica en el sector septentrional del Valle de Ambato, Catamarca. *Anales de Arqueología y Etnología, 74*(2), 219-245.

Dantas, M., Takigami, M., Márquez, M. A., Milone, O. E., Sechino Mosquera, L. M., Vera, S., Arellano González, M. C., & Tokanai, F. (2025). Dimensiones temporales de las ocupaciones en el sector norte del Valle de Ambato, Catamarca. Manuscrito en preparación.

Del Papa, L. (2012). Una aproximación al estudio de los sistemas de subsistencias a través del análisis arqueofaunístico en un sector de la cuenca del Río Dulce y cercanías a la Sierra de Guasayán [Tesis doctoral inédita]. Facultad de Ciencias Naturales y Museo, Universidad Nacional de La Plata.

Del Papa, L. M., & Lamenza, G. (2019). Avances en la zooarqueología chaqueña. El sitio La Ilusión I (Chaco, Argentina). *Arqueología, 25*(3), 143-166.

Del Río, P., & Cornero, S. (2021). Localidad Arqueológica Laguna del Plata, Vera y Pintado, Depto. San Justo, Santa Fe. Nuevas contribuciones. *Innovare Revista de Ciencia y Tecnología. XV Jornadas SECYT, UNRosario*.

Del Río, P., Cornero, S., Ceruti, C., & Echegoy, C. (2016). Arqueología de los Bajos Submeridionales: sitios con hornos de tierra cocida en la localidad arqueológica Laguna La Blanca (La Criolla, Departamento San Justo, provincia de Santa Fe). *Revista de Antropología del Museo de Entre Ríos, 2*(2), 67-82.

Devoto, F., & Perroto, B. (1973). Patología macroscópica dento-alveolar de la población precolombina de Tastil. En E. Cigliano (Ed.), *Tastil, una ciudad preincaica argentina*. Ed. Cabargon.

Di Prado, V. (2015). Estudio comparativo de las prácticas de elaboración y uso de la alfarería prehispánica del centro-este de Argentina desde una perspectiva macrorregional [Tesis doctoral inédita]. Universidad Nacional de La Plata.

Di Prado, V. (2018). Prácticas alfareras prehispánicas y procesos de interacción social en el centro-este de Argentina durante el Holoceno tardío. *Latin American Antiquity, 29*(3), 552-531.

Dias, G., & Tayles, N. (1997). Abscess cavity-a misnomer. *International Journal of Osteoarchaeology, 7*, 548-554.

Díaz, H., & Markgraf, V. (Eds.). (1992). *El Niño: Historical and paleoclimatic aspects of the Southern Oscillation*. Cambridge University Press.

Doello-Jurado, M. (1940). Los aborígenes de Santiago del Estero. Síntesis malacológica. *Relaciones de la Sociedad Argentina de Antropología*, 2, 124-144.

Drube, H., Silvera, E., Martínez, S., Desántolo, B., Lamenza, G., & Salceda, S. (2016). Scurvy in past populations of Argentina: A probable case study from the Gran Chaco Area. *Book of Abstracts of the 21st European Meeting of the Paleopathology Association*. Moscow, Russia.

Drube, H. (2009). Las poblaciones aborígenes prehispánicas de Santiago del Estero. Evaluación de sus características bioantropológicas y de sus condiciones de salud, enfermedad y nutrición [Tesis doctoral inédita]. Facultad de Ciencias Naturales y Museo, Universidad Nacional de La Plata.

Echegoy, C. (1994). Arqueología del Paraná. Los fechados de Arroyo Aguilar. Museo de Reconquista.

Fabra, M., Gordillo, S., Colombo, F., Nores, R., & Sario, G. (2023). A human body, a necklace and a stone axe: Osteobiographical and relational perspectives of a 4,000-year-old burial in hunter-gatherer societies in central Argentina. *Latin American Antiquity, 34*(2), 349-365. https://doi.org/10.1017/laq.2022.39

Fabra, M. (2019). Informe bioantropológico de restos óseos humanos. Sitio Uturunco, individuo 1. Manuscrito en preparación.

Fabra, M., Alderete, A. P., & Ferreyra, E. S. (2019). Investigaciones bioarqueológicas en costa este de la Laguna del Plata e Isla El Mistolar (Laguna Mar Chiquita, Córdoba). *Revista Voces del ayer para leer hoy, revista del Museo Histórico Municipal de Marull, Serie Cuadernos del Museo Nro. 6*, 9-45.

Fabra, M., González, C. V., & Robin, S. (2015). Evidencias de violencia interpersonal en poblaciones del piedemonte y las llanuras de Córdoba (Argentina) a finales del Holoceno tardío. *Revista RUNA, 36*(1), 5-27.

Fabra, M., & Demarchi, D. (2012). Morfología craneofacial y estructura genética en poblaciones del centro de Argentina. *Revista Argentina de Antropología Biológica, 14*(1), 45-56.

Fabra, M., González, C., & Salega, S. (2012). Modos de vida e historia biológica de poblaciones de las Sierra y Llanuras de Córdoba (Argentina): Aproximaciones desde el registro bioarqueológico. *Revista Argentina de Antropología Biológica, 17*, 87-104.

Fabra, M., Gordillo, S., & Piovano, E. L. (2012). Arqueomalacología en las costas de Ansenuza: Análisis de una almeja nacarífera (Anodontites trapesialis) hallada en contexto funerario del sitio El Diquecito (Laguna Mar Chiquita, Córdoba). *Revista Arqueología, 18*, 257-266.

Fabra, M., & Gordillo, S. (2015). Estimaciones acerca del uso de una almeja de agua dulce (Diplodon parallelopipedon) hallada en contexto arqueológico en el Mar de Ansenuza (Córdoba, Argentina). En H. Hammond & M. Zubimendi (Eds.), *Arqueología y malacología: Abordajes metodológicos y casos de estudio en el Cono Sur* (pp. 129-144). Vazquez Mazzini Editores.

Fabra, M., Salega, S., González, C., Smeding, R., & Pautassi, E. (2008). Arqueología de rescate en la costa sur de la Laguna Mar Chiquita: Sitio arqueológico El Diquecito. *Memorias del Pueblo: Revista del Museo Histórico Municipal La Para, 8*(8), 37-46.

Fabra, M., Salega, S., & González, C. (2009). Comportamiento mortuorio en poblaciones prehispánicas de la región austral de las Sierras Pampeanas durante el Holoceno. *Revista Arqueología, 15*, 165-186.

Fabra, M., Salega, S., González, C. V., & Tavarone, A. (2014). Lo que el agua nos dejó: Investigaciones bioarqueológicas en la costa sur de la laguna Mar Chiquita (Córdoba, Argentina). *Jangwa Pana, 13*, 51-64.

Fabra, M., & Salega, M. S. (2016). Developmental defects in the spine: Initial findings in archaeological cases from Córdoba highlands (Argentina). *International Journal of Osteoarchaeology, 26*, 397-407. https://doi.org/10.1002/oa.2429

Figueroa, G. G., & Dantas, M. (2020). Estado de avance de las investigaciones arqueológicas en el valle de Ambato, siglos VI al XI d.C., Catamarca, Argentina. *Cuadernos del Instituto Nacional de Antropología y Pensamiento Latinoamericano, 29*(2), 96-128.

Flegenheimer, N., Guichon, R., & Scabuzzo, C. (2002). Restos óseos humanos en el sitio El Guanaco, partido de San Cayetano. En D. L. Mazzanti, M. A. Beron, & F. W. Oliva (Eds.), *Del mar a los salitrales. Diez mil años de historia pampeana en el umbral del tercer milenio* (pp. 121-126). Universidad Nacional de Mar del Plata.

Gaggero, P. (1921). Expedición al Delta del Paraná 1921. Arroyo Fredes (enero de 1921). MLP-Ar-D36. Repositorio Culturalis, División Arqueología, Museo de La Plata, FCNyM, UNLP.

García, A., Demarchi, D. A., Tovo-Rodrigues, L., Pauro, M., Callegari-Jacques, S. M., Salzano, F. M., & Hutz, M. H. (2015). High interpopulation homogeneity in Central Argentina as assessed by ancestry informative markers (AIMs). *Genetics and Molecular Biology*, 38(3), 324-331. https://doi.org/10.1590/S1415-475738320140300

García, A., Moralejo, R. A., & Ochoa, P. A. (2021). Radiocarbon chronology of the Inca expansion in Argentina. *Antípoda. Revista de Antropología y Arqueología*, 42, 51-83. https://doi.org/10.7440/antipoda42.2021.03

García, A., Greco, C., Moralejo, R. A., & Ochoa, P. A. (2023). Aplicación de estadística bayesiana al estudio de la cronología de la expansión incaica en Argentina. *Arqueología*, 29(1), 1-21. https://doi.org/10.34096/arqueologia.t29.n1.11140

García, A., Nores, R., Motti, J. M. B., Pauro, M., Luisi, P., Bravi, C. M., Fabra, M., Gosling, A. L., Kardailsky, O., Boocock, J., Solé-Morata, N., Matisoo-Smith, E. A., Comas, D., & Demarchi, D. A. (2021). Ancient and modern mitogenomes from Central Argentina: New insights into population continuity, temporal depth and migration in South America. *Human Molecular Genetics*, 30(13), 1200-1217. https://doi.org/10.1093/hmg/ddab105

García, A., Pauro, M., Bailliet, G., Bravi, C. M., & Demarchi, D. A. (2018). Genetic variation in populations from Central Argentina based on mitochondrial and Y chromosome DNA evidence. *Journal of Human Genetics*, 63(4), 493-507. https://doi.org/10.1038/s10038-018-0417-5

García, A., Pauro, M., Nores, R., Bravi, C. M., & Demarchi, D. A. (2012). Phylogeography of mitochondrial haplogroup D1: An early spread of subhaplogroup D1j from Central Argentina. *American Journal of Physical Anthropology*, 149(4), 583-590. https://doi.org/10.1002/ajpa.22154

Gaspary, F. (1950). Investigaciones arqueológicas y antropológicas en un cerrito de la Isla Los Marinos (Pcia. de Entre Ríos). *Publicación del Instituto de Arqueología, Lingüística y Folklore, XXIII*, 3-66.

Gennard, D. (2007). *Forensic entomology. An introduction*. John Wiley & Sons Ltd.

Gheggi, M. (2006). Más allá de los huesos. El estudio integral de la evidencia de los enterratorios de Esquina de Huajra (Dto. Tumbaya, Quebrada de Humahuaca) en el contexto histórico regional. *Arqueología, 13*, 47-78.

Gheggi, M. (2011). Un enfoque biocultural aplicado al estudio de entierros arqueológicos del Noroeste Argentino (ca. 1000-1550 A. D.) [Tesis doctoral]. Facultad de Filosofía y Letras, Universidad de Buenos Aires.

Gheggi, M., & Seldes, V. (2012). Evidencias bioarqueológicas de conflicto ca. 1000-1432 AD en la Quebrada de Humahuaca y el Valle Calchaquí. *Intersecciones en Antropología, 13*, 103-115.

Gheggi, M., & Seldes, V. (2014). Social change and health status in prehispanic Northwest Argentina (Quebrada de Humahuaca, Jujuy) ca. 500-1550 AD. *Journal of Anthropology and Archaeology, 2*, 17-38.

Ginzburg, R., & Adámoli, J. (2006). Situación ambiental en el Chaco Húmedo. En A. Brown, M. Martínez Ortiz, M. Acerbi, & J. Corcuera (Eds.), *La Situación Ambiental Argentina* (pp. 103-113). Fundación Vida Silvestre Argentina.

Gómez, R. (1966). *La Cultura de Las Mercedes (Contribución a su estudio)*. Edición privada.

Gómez, R. (1975). Contribución al conocimiento de las industrias líticas tempranas de Santiago del Estero. *Revista del Instituto de Antropología, 2*, 171-187.

Gómez, R. (2009). Arqueología santiagueña: Un diseño de investigación para el Formativo Inferior. Fase explorativa. *Revista del Museo de Antropología, 2*, 53-66.

Gonzalez Baroni, L. G. (2014). Un entierro secundario múltiple del primer milenio A.D. en la Puna Meridional (Antofagasta de la Sierra, Catamarca, Argentina). En L. H. Luna, C. Aranda, & J. Suby (Eds.), *Avances Recientes de la Bioarqueología Latinoamericana* (pp. 169-186).

Gonzalez Baroni, L. G. (2024). Prácticas sociales en torno a la muerte: Las costumbres funerarias y el registro bioarqueológico en grupos cazadores-recolectores y agropastoriles de la Puna Argentina ca. 9000-1000 AP [Tesis doctoral]. Facultad de Ciencias Naturales e I.M.L., Universidad Nacional de Tucumán.

Gonzalez Baroni, L. G., Aranda, C. M., & Luna, L. H. (2019). Sujetos de violencia en sociedades agropastoriles de la Puna Meridional Argentina: Prácticas mortuorias y evidencias de traumas en esqueletos humanos del sitio Punta de la Peña 9.I, Antofagasta de la Sierra, Catamarca, Argentina. *Latin American Antiquity, 30*(3), 490-509.

González, A. (1947). Investigaciones arqueológicas en las nacientes del Paraná Pavón. Universidad Nacional de Córdoba.

González, A. R. (1960). Nuevas fechas de la cronología arqueológica argentina obtenidas por el método del radiocarbono (IV). *Revista del Instituto de Antropología, 1*, 303-331.

González, A. R. (1998). *Cultura La Aguada. Arqueología y diseños*. Filmediciones Valero.

Gonzalez, M. E., Messineo, P. G., Marini, N., & Politis, G. G. (2023). Modalidad de entierro, edad, cronología y dieta en los cazadores-recolectores del Campo de Dunas del Centro Pampeano: El sitio Laguna de los Pampas (región pampeana, Argentina). *Chungara, Revista de Antropología Chilena, 55*(2). http://dx.doi.org/10.4067/S0717-73562023005000801

Gordillo, I. (2012). Eso que llamamos Aguada. Su lugar en la arqueología. Trabajo presentado en "Arqueología del Periodo Formativo en Argentina: Un encuentro para integrar áreas y subdisciplinas, revisar significados y potenciar el impacto de las investigaciones en curso", Tafí del Valle, Tucumán.

Gordillo, I., & Solari, A. (2009). Prácticas mortuorias entre las poblaciones Aguada del valle de Ambato (Catamarca, Argentina). *Revista Española de Antropología Americana, 39*(1), 31-51.

Gramajo de Martínez Moreno, A. (1991). *El Proceso Fundacional en el Antiguo Tucumán*. Editorial V Centenario.

Gramajo de Martínez Moreno, A. (1992). La arqueología santiagueña. Marco teórico, sustentos metodológicos y nuevos aportes. *Serie Estudio, 4*, 1-19. Museo de Ciencias Antropológicas y Naturales "Emilio y Duncan Wagner".

Granero, C., & Cornero, S. (2022). Improntas en cerámicas: Un análisis comparativo de estructuras textiles en la región central de Argentina. *Revista Sociedades de Paisajes Áridos y Semi-Áridos*, 12(16), 11-41.

Greco, C. (2017). Statistical analysis of radiocarbon datings from the south central sector of Quebrada de Humahuaca. In A. Scaro, C. Otero, & M. B. Cremonte (Eds.), *Pre-Inca and Inca pottery, Quebrada de Humahuaca, Argentina* (pp. 169-188). Springer.

Herrero, R., & Ávila, A. (1991). Excavaciones en la unidad residencial ScatAmb 004 (Martínez 4) del Período de Integración Regional. *Publicaciones del CIFFyH, Arqueología, 46*, 111-130.

Hillson, S. (1996). *Dental Anthropology*. Cambridge University Press.

Hocsman, S. (2014). Continuities and discontinuities in the process of transition to food production in Antofagasta de la Sierra (Southern Argentine Puna): The case of flaked stone tools. En E. Pintar (Ed.), *Hunter-gatherers from a High-Elevation Desert: People of the Salt Puna Northwest Argentina* (pp. 201-230). Archaeopress.

Hocsman, S., & Alderete, M. (2022). Similar no es necesariamente algo idéntico: Reclamación de un diseño de punta de proyectil en el tránsito a la producción de alimentos en Antofagasta de la Sierra (provincia de Catamarca, Argentina). *Arqueología, 28*(1), 9935.

Hocsman, S., & Babot, M. P. (2018). La transición de cazadores-recolectores a sociedades agropastoriles en Antofagasta de la Sierra (Puna de Catamarca, Argentina): Perspectivas desde la agencia y las prácticas. *Chungara. Revista de Antropología Chilena, 50*(1), 51-70.

Hyslop, J. (1990). *Inca settlement planning*. University of Texas Press.

Ilivera, D. E. (2001). Sociedades agropastoriles tempranas: El formativo inferior del noroeste argentino. En E. E. Berberián & A. E. Nielsen (Eds.), *Historia argentina prehispánica* (Tomo I, pp. 83-125). Editorial Brujas.

Iriondo, M. (1999). Climatic changes in the South American plains: Records of a continent-scale oscillation. *Quaternary International, 57/58*, 93-112.

Iriondo, M. (2006). Cambios ambientales en el Chaco argentino y boliviano en los últimos miles de años. *Folia Histórica del Nordeste, 16*, 39-50.

Iriondo, M., & Kröhling, D. (1995). El Sistema Eólico Pampeano. *Comunicaciones del Museo Provincial de Ciencias Naturales "Florentino Ameghino", 5*(1), 1-68.

Izeta, A. (2024). ¿Es la arqueología cordobesa “FAIR”? Una mirada desde la arqueología pública digital y la ciencia abierta. *Anales de Arqueología y Etnología, 79*(1), 149-168. https://doi.org/10.48162/rev.46.041

Izeta, A. D., & Cattáneo, R. (2023). Towards an open digital ecosystem for archaeology in South America: The BADACor (Córdoba Archaeological Sites Database) as a case of an open digital archaeological source for heritage management in central Argentina.

Izeta, A. D., Cattáneo, R., Robledo, A. I., Takigami, M., Yoneda, M., Tokanai, F., Kato, K., & Matsuzaki, H. (2021). New radiocarbon evidence for human occupation in central Argentina during the Middle and Late Holocene: The Ongamira Valley case. *Radiocarbon*, 1-20. https://doi.org/10.1017/RDC.2021.22

Izeta, A., Cattáneo, R., Robledo, A. I., & Mignino, J. (2017). Aproximación multiproxy a los estudios paleoambientales de la provincia de Córdoba: El valle de Ongamira como caso. *Revista del Museo de Antropología, 10*(Supl. Esp. 1), 33-42. https://doi.org/10.31048/1852.4826.v10.n0.14401

Juez, M. S. (1991). Unidad arqueológica Rodeo Grande, Valle de Ambato: Excavación en el sitio Martínez 2. *Publicaciones del CIFFyH, Arqueología, 46*, 87-110.

Killian Galván, V. A., Grant, J. L., Morales Puente, P., Cienfuegos Alvarado, E., Otero, F. J., Pérez, M. I., & Olivera, D. E. (2021). Empire and stable isotopes: Assessing the impact of Inka expansion on local diet in the southern Puna, Argentina. *Antiquity*, 1-17.

Killian Galván, V., & Olivera, E. (2008). First δ13C values for human skeletal remains from southwestern Puna (Jujuy, Argentina). *VI South American Symposium on Isotope Geology*. San Carlos de Bariloche, Argentina.

Killian Galván, V., Olivera, D., & Gallegos, E. (2012). Una aproximación isotópica al consumo de maíz en la localidad arqueológica Río Doncellas (Depto. De Cochinoca, Prov. de Jujuy). En M. Babot, M. Marschoff, & F. Pazzarelli (Eds.), *Las manos en la masa. Arqueologías, antropologías e historias de la alimentación en Suramérica* (pp. 319-338).

Killian Galván, V., Martínez, J. G., Cherkinsky, A., Mondini, M., & Panarello, H. (2016). Stable isotope analysis on human remains from the final Early Holocene in the southern Puna of Argentina: The case of Peñas de las Trampas 1.1. *Environmental Archaeology, 21*(1), 1-10.

Korstanje, A., Lazzari, M., Basile, M., Bugliani, F., Lema, V., Pereyra Domingorena, L., & Quesada, M. (2015). *Crónicas materiales precolombinas. Arqueología de los primeros poblados del Noroeste Argentino*. Sociedad Argentina de Antropología.

Kozameh, L., Testa, N., López, M., Mango, L., & Cornaglia, J. (2018). Huesos rojos en el Delta superior del río Paraná. El caso del sitio Cerro Grande de la isla Los Marinos (Entre Ríos, Argentina). En G. Politis & M. Bonomo (Eds.), *Goya Malabrigo Arqueología de una sociedad indígena del Noreste argentino* (pp. 129-148). UNI-CEN.

Kozameh, L., & Brunás, O. (2011). Paleopatología: Paget óseo en un resto prehispánico. Microscopía y datación. *Actualizaciones en Osteología, 7*(2), 93-95.

Kozameh, L., & Brunás, O. (2013). Enfermedad de Paget en un individuo prehispánico del delta del Paraná, confirmado por examen histológico y datación radiocarbónica. *Cuadernos del Instituto Nacional de Antropología y Pensamiento Latinoamericano, 1*(1), 114-120.

Kruck, W., Helms, F., Geyh, M. A., Suriano, J. M., Marengo, H. G., & Pereyra, F. (2011). Late Pleistocene-Holocene history of Chaco-Pampa sediments in Argentina and Paraguay. *Quaternary Science Journal, 60*, 188-202.

Laguens, A. (1999). *Arqueología del contacto hispano indígena. Un estudio de cambios y continuidades en las Sierras Centrales de Argentina*. John & Erica Hedges.

Laguens, A. G. (2007). Contextos materiales de desigualdad social en el valle de Ambato, Catamarca, Argentina, entre los siglos VII y X d.C. *Revista Española de Antropología Americana, 37*(1), 27-49.

Laguens, A., & Bonnin, M. (2023). *Sociedades Indígenas de las Sierras Centrales. Arqueología de Córdoba y San Luis* (2nd ed.). Editorial Universitaria, Universidad Nacional de Córdoba.

Lamenza, G. (2013). *El hombre y el ambiente en el Holoceno Tardío del Chaco Meridional*. Universidad Nacional de Catamarca, Facultad de Ciencias Exactas y Naturales.

Lamenza, G. (2015). Utilización del análisis multivariante para la sistematización del componente alfarero del Chaco prehispánico. *Arqueología Iberoamericana, 28*, 52-61.

Lamenza, G., & Alfonso Monges, M. (2020). Branislava Susnik y su contribución a la arqueología paraguaya. *Suplemento Antropológico, LV*(1), 43-76.

Lamenza, G., & Plischuk, M. (2015). Avances en bioarqueología del Chaco boreal. *Arqueología Iberoamericana, 28*, 75-80.

Lamenza, G., & Santini, M. (2013). Arqueología del Chaco Meridional: Nuevos aportes a la comprensión de la dinámica cultural chaqueña prehispánica. *Suplemento Antropológico, XLVIII*(2), 145-220.

Lamenza, G., Desántolo, B., Drube, H., Calandra, H., Salceda, S., & Sempé, C. (2016). Nuevos aportes a la arqueología del Valle de Hualfín: El sitio Cardón Mocho de Azampay (Belén, Catamarca). *Revista Española de Antropología Americana, 46*, 305-328.

Lamenza, G., Calandra, H., & Salceda, S. (2015). Primera datación radiocarbónica del sitio puerto 14 de mayo (Bahía Negra, Alto Paraguay). *Relaciones de la Sociedad Argentina de Antropología, 40*(1), 351-358.

Lamenza, G., Calandra, H., & Salceda, S. (2016). Nuevos aportes a la arqueología de Formosa (Argentina): Cronología del sitio arqueológico El Quebracho. *Arqueología, 22*(2), 399-408.

Lamenza, G., Calandra, H., & Salceda, S. (2018). Identificación de componentes arqueológicos a través de técnicas numéricas: Un caso de aplicación. *Arqueología Iberoamericana, S3*, 35-37.

Lamenza, G., Calandra, H., & Salceda, S. (2019). Arqueología de los ríos Pilcomayo, Bermejo y Paraguay. *Revista del Museo de La Plata, 4*(2), 481-510.

Larsen, C. (1987). Biarchaeological interpretations of subsistence economy and behavior from human skeletal remains. *Advances in Archaeological Method and Theory, 10*, 339-445.

Leibowicz, I., & Jacob, C. (2012). La conquista Inka de Humahuaca, Jujuy, Argentina. Nuevos fechados y visiones desde los desarrollos regionales. *Inka Llaqta*, 3, 191-210.

Lindskoug, H. (2008). En la sombra de la arqueología argentina: Jorge von Hauenschild y la formación de la colección von Hauenschild del Museo de Antropología (Universidad Nacional de Córdoba). *Revista del Museo de Antropología, 1*(1), 61-70. https://doi.org/10.31048/1852.4826.v1.n0.5396

López Campeny, S. (2001). Actividades domésticas y organización del espacio intrasitio. El sitio Punta de la Peña 9 (Antofagasta de la Sierra-Catamarca) [Tesis de licenciatura]. Facultad de Ciencias Naturales e IML, Universidad Nacional de Tucumán.

López Campeny, S. M. L., Romano, A. S., Rodríguez, M. F., Martel, A. R., & Corbalán, M. (2014). De aquí y de allá: Análisis integral de un contexto funerario. Vínculos e interacciones sociales entre Puna meridional y Tierras Bajas orientales. *Intersecciones en Antropología, 15*, 201-218.

Loponte, D., & Acosta, A. (2015). Los sitios arqueológicos Túmulo de Campana 1 y 2 dentro del contexto regional del humedal del Paraná Inferior. *Revista de Antropología del Museo de Entre Ríos, 1*(2), 11-40.

Lorandi, A. M. (1974). Espacio y tiempo en la prehistoria santiagueña. *Relaciones de la Sociedad Argentina de Antropología, 8*, 199-236.

Lorandi, A. M. (1978). El desarrollo cultural prehispánico en Santiago del Estero, Argentina. *Journal de la Société des Amèricanistes, 65*, 61-85.

Lorandi, A. M. (2015). *Tukuma-Tukuymanta. Los pueblos del búho. Santiago del Estero antes de la Conquista*. Subsecretaría de Cultura de la Provincia de Santiago del Estero.

Lothrop, S. K. (1925). The Thea Heye La Plata Expedition. *Indian Notes, 2*(4), 257-266.

Lothrop, S. K. (1932). Indians of the Paraná Delta, Argentina. *Annals of the New York Academy of Science, 32*, 77-232.

Luna, H. L., Aranda, C. M., Santos, A. L., Donoghue, H. D., Ying-Chi Lee, O., Tin Wu, H. H., Singh Besra, G., Minnikin, D. E., Llewellyn, G., Williams, C. M., & Ratto, N. (2020). Oldest evidence of tuberculosis in Argentina: A multidisciplinary investigation in an adult male skeleton from Saujil, Tinogasta, Catamarca (905-1030 CE). *Tuberculosis, 125*, 1-13.

Marconetto, M. B. (2008). Recursos forestales y el proceso de diferenciación social en tiempos prehispánicos en el valle de Ambato, Catamarca, Argentina. *British Archaeological Reports - South American Archaeology Series*.

Marlowe, F. (2005). Hunter-gatherers and human evolution. *Evolutionary Anthropology, 14*, 54-67.

Martel, A. R. (2010). Arte Rupestre de Pastores y Caravaneros. Estudio Contextual de las Representaciones Rupestres durante el Período Agroalfarero Tardío (900-1480 DC) en el

Noroeste Argentino [Tesis doctoral inédita]. Facultad de Filosofía y Letras, Universidad Nacional de Buenos Aires.

Martínez, J. G. (2014). Contributions to the knowledge of natural history and archaeology of hunter-gatherers of Antofagasta de la Sierra (Southern Argentinian Puna): The case of Peñas de las Trampas 1.1. En E. L. Pintar (Ed.), *Hunter-gatherers from a high-altitude desert. People of the Salt Puna (northwest Argentina)* (pp. 71-93). British Archaeological Reports (BAR), International Series 2641.

Martínez, J. G., Mauri, E. P., Mercuri, C., Caria, M. A., & Oliszewski, N. (2013). Mid-Holocene human occupations in Tucumán (northwest Argentina). *Quaternary International, 307*, 86-95.

Martinez Soler, B. J. (1964). Acerca de la determinación de las rutas de desplazamientos étnicos y culturales. *Anales de la Universidad del Salvador*, 1, 259-276.

Mazza, B., Acosta, A., & Loponte, D. (2016). Nuevos datos para las inhumaciones en urnas de sitios arqueológicos guaraníes del extremo meridional de la Cuenca del Plata. *Revista Chilena de Antropología, 34*, 81-96.

Mazzia, N. I., Scabuzzo, C., & Guichon, R. (2004). Sobre cráneos, pelvis y otros huesos. Entierros humanos en el sitio El Guanaco. En G. Martínez, M. Gutiérrez, R. Curtoni, M. Beron, & P. Madrid (Eds.), *Aproximaciones Arqueológicas Pampeanas: Teorías, Métodos y Casos de Aplicación Contemporáneos* (pp. 293-304). INCUAPA-UNCPBA.

Medina Reguilón, N. M., Babot, P., & Hocsman, S. (2025). Understanding inhabited landscapes through distant foreign Leguminosae tree plant fruit used by agro-pastoralist societies in the high elevation deserts of southern South-Central Andes (Punta de la Peña 9, Argentina, sixth through eighth centuries AD). *Vegetation History & Archaeobotany*. En prensa.

Medina, M. E. (2015). Casas-pozo, agujeros de postes y movilidad residencial en el Período Prehispánico Tardío de las Sierras de Córdoba, Argentina. En J. Salazar (Ed.), *Condiciones de posibilidad de la reproducción social en sociedades prehispánicas y coloniales tempranas en las Sierras Pampeanas (República Argentina)* (pp. 267-301). Centro de Estudios Históricos “Carlos Segreti”.

Mendonça, O., Bordach, M., & Arrieta, M. (2012). Arqueología y bioarqueología: Interacciones y perspectivas para el registro fragmentado de una evidencia incompleta. *Revista Argentina de Antropología Biológica, 14*, número especial, 23-32.

Merlo, N., Mendonça, O., Bordach, M., & Ruiz, M. (2005). Vida y muerte en el Pucara de Yacoraite. Estudio de osteología humana. *Cuadernos de la Facultad de Humanidades y Ciencias Sociales, 29*, 113-142.

Merlotto, A., & Bértola, G. R. (2009). Coastline evolution at Balneario Parque Mar Chiquita, Argentina. *Ciencias Marinas, 35*(3), 271-286.

Messineo, P., Favier Dubois, C. M., Politis, G. G., & Vitale, P. (2021). Site formation process and megamammal bone radiocarbon dates in Campo Laborde (Pampas of Argentina): Contribution towards a research methodology. *Quaternary International, 586*, 53-65.

Messineo, P. G., Gonzalez, M. E., Álvarez, M. C., & Pal, N. (2018). Las ocupaciones humanas en la localidad arqueológica Laguna de los Pampas (Campo de Dunas del Centro Pampeano, Argentina) durante el Holoceno. *Latin American Antiquity, 29*(4), 736-753.

Messineo, P. G., Scheifler, N. A., Álvarez, M. C., Gonzalez, M. E., Pal, N., Barros, M. P., & Politis, G. G. (2019). Was the Central Pampean Dunefields of Argentina Occupied during the Late Pleistocene? A Reappraisal of the Evidence. *PaleoAmerica: A Journal of Early Human Migration and Dispersal, 5*(4), 378-391. https://doi.org/10.1080/20555563.2019.1697620

Miranda de Zela, P. (2013). Bioarqueología de la Puna de Jujuy, Argentina [Tesis de licenciatura]. Universidad de Buenos Aires.

Miranda de Zela, P. (2018). Salud y enfermedad de las poblaciones arqueológicas de la Puna Argentina durante el Periodo Tardío y Tardío-Inka (ca. 1000-1535 D.C.) [Tesis doctoral]. Universidad de Buenos Aires.

Miranda de Zela, P. (2021). Volver a Doncellas: Análisis paleopatológico de muestras osteológicas recuperadas en la década de 1970 en la Puna de Jujuy, República Argentina. *Mundo De Antes, 15*(1), 223-258.

Miranda de Zela, P., & Fuchs, M. L. (2021). Introducción al Dossier "Puna de Jujuy: Encuentros y desencuentros en la Arqueología y Bioarqueología de la región puneña". *Mundo De Antes, 15*(1), 99-102. https://doi.org/10.59516/mda.v15.223

Mondini, M., Martínez, J. G., Pintar, E., & Reigadas, M. C. (2013). Middle Holocene foraging, mobility and landscape use in the southern Argentinean Puna: Hunter-gatherers from Antofagasta de la Sierra, Catamarca, Argentina. *Quaternary International, 307*, 66-73.

Montes, A. (1943). Yacimiento arqueológico de Ongamira. *Congreso de Historia del Norte y Centro*. Córdoba: Editorial Litvack.

Motti, J. M., Pauro, M., Scabuzzo, C., García, A., Aldazábal, V., Vecchi, R., & Nores, R. (2023). Ancient mitogenomes from the Southern Pampas of Argentina reflect local differentiation and limited extra-regional linkages after rapid initial colonization. *American Journal of Biological Anthropology, 181*(2), 216-230.

Murgo, A. (2002). Contribución al estudio de los patrones de subsistencia a partir de análisis de isótopos estables y elementos traza. Primeros resultados para restos humanos del sector Centro Oriental de la Pampa Deprimida [Tesis de licenciatura]. Facultad de Filosofía y Letras, Universidad de Buenos Aires.

Murgo, A., & Aldazabal, V. (2001). Resultados de los análisis de isótopos estables de carbono y nitrógeno de los restos humanos provenientes de sitios del área Pampa Deprimida Centro

Oriental. *Libro de Resúmenes. XIV Congreso Nacional de Arqueología Argentina*. Facultad de Humanidades y Artes, Escuela de Antropología, Universidad Nacional de Rosario.

Nakatsuka, N., Luisi, P., Motti, J. M., Salemme, M., Santiago, F., D'Angelo del Campo, M. D., & Reich, D. (2020). Ancient genomes in South Patagonia reveal population movements associated with technological shifts and geography. *Nature Communications, 11*(1), 3868.

Naumann, M. (2006). *Atlas del Gran Chaco Sudamericano*. Sociedad Alemana de Cooperación Técnica (GZT).

Neme, G., & Gil, A. F. (2009). Human occupation and increasing mid-Holocene aridity. Southern Andean perspectives. *Current Anthropology, 50*, 149-163.

Nielsen, A. (1996). Demografía y cambio social en Quebrada de Humahuaca (Jujuy, Argentina) 700-1535 d.C. *Relaciones de la Sociedad Argentina de Antropología*, 21, 307-354. http://sedici.unlp.edu.ar/handle/10915/25024

Nielsen, A. (1997). Tendencias temporales en la cultura material de la Quebrada de Humahuaca (Jujuy, Argentina) ca. 700-1650 d.C. *Avances en Arqueología*, 3, 147-189.

Nielsen, A. (2006). Pobres jefes: Aspectos corporativos en las formaciones sociales preincaicas de los Andes Circumpuneños. En C. Gnecco & H. Langebaek (Eds.), *Contra la tiranía del pensamiento tipológico en arqueología: Una visión desde Suramérica* (pp. 120-150). Ediciones Uniandes.

Nielsen, A. (2007). Armas significantes: Tramas culturales, guerra y cambio social en el Sur Andino prehispánico. *Boletín del Museo Chileno de Arte Precolombino, 12*(1), 9-41.

Nielsen, A. E., & Walker, W. H. (1999). Conquista ritual y dominación política en el Tawantinsuyu: El caso de Los Amarillos (Jujuy, Argentina). In A. Zarankin & F. A. Acuto (Eds.), Sed non satiata. *Teoría social en la arqueología latinoamericana contemporánea* (pp. 153-169). Ediciones del Tridente.

Nóbile, J. C., Brizuela, C., Collo, G., Robledo, A., Perassi, I., Wunderlin, C., Mignino, J., Ribeiro Guevara, S., Germanier, A., Faudone, S., Rustán, M., Izeta, A., & Cattáneo, R. (2025). Multiproxy analysis of clay sources and pottery sherds to elucidate the provenance of archaeological pottery in the Characato region, Córdoba, Argentina. *Journal of Archaeological Science: Reports, 62*, 104993. https://doi.org/10.1016/j.jasrep.2025.104993

Noelli, S. F., Brochado, J. P., & Alves Corrêa, Â. (2018). A linguagem da cerâmica Guaraní: Sobre a persistência das práticas e materialidade (parte 1). *Revista Brasileira de Linguística Antropológica, 10*(2), 167-200.

Nores, B., & D'Andrea, U. (1997). Alpa Corral: Un reservorio arqueológico de cazadores superiores.

Nores, R., Fabra, M., & Demarchi, D. (2011). Variación temporal y espacial en la población humana del actual territorio de Córdoba. Análisis de ADN antiguo. *Revista del Museo de Antropología (F.F. y H., U.N.C.), 4*, 187-193.

Nores, R., Lamenza, G., García, A., Salceda, S., Boocok, J., & Demarchi, D. (2017). Análisis del genoma mitocondrial completo de una muestra arqueológica humana del sitio "El Quebracho" (Formosa, Argentina). Trabajo completo presentado con resumen publicado en la revista *Scientia Interfluvius - Suplementos Resúmenes VII Encuentro de discusión Arqueológica del Nordeste Argentino*, 52.

Núñez Regueiro, V., & Tartusi, M. (1990). Aproximación al estudio del área Pedemontana de Sudamérica. *Cuadernos, 12*, 125-160.

Oliszewski, N., Martínez, J. G., Arreguez, G. A., Gramajo Bühler, C. M., & Naharro, M. E. (2018). "La transición" vista desde los valles intermontanos del NOA: Nuevos datos de la Quebrada de los Corrales (El Infiernillo, Tucumán). *Chungará. Revista de Antropología Chilena, 50*(1), 71-86.

Ortner, D. (2008). Differential diagnosis of skeletal lesions in infectious disease. En S. Mays & R. Pinhasi (Eds.), *Advances on human paleopathology* (pp. 191-214). John Wiley & Sons Ltd.

Ottalagano, F. V. (2013). Aves simbólicas, estilo e identidad en la arqueología del gran río sudamericano: Un estudio contextual del arte cerámico de las sociedades prehispánicas de la cuenca del Paraná Medio. *Serie Monográfica, Vol. III, INAPL*.

Palavecino, E. (1948). Noticia preliminar sobre un viaje arqueológico a Goya. *Notas del Museo de la Plata, 13*, 253-258.

Palma, J. (1997-1998). Ceremonialismo mortuorio y registro arqueológico: Apuntes sobre complejidad social. *Relaciones de la Sociedad Argentina de Antropología, XXII/XXIII*, 179-202.

Palma, J. (2000). Urbanismo y complejidad social en la región Humahuaca. *Estudios Sociales del NOA*, 3, 31-57.

Pärsinnen, M. (2003). *Tawantinsuyu. El Estado Inca y su organización política*. Instituto Francés de Estudios Andinos.

Paucke, F. (1942-1944). *Hacia allá y para acá. Una estada entre los indios mocobíes (1749-1767)*. Universidad Nacional de Tucumán.

Pérez Gollán, J. A., & Heredia, O. R. (1987). Hacia un replanteo de la Cultura de la Aguada. *Cuadernos del Instituto Nacional de Antropología, 12*, 161-178.

Pérez Gollán, J. A. (1991). La Cultura de la Aguada vista desde el Valle de Ambato. *Publicaciones del CIFFYH, Arqueología, 46*, 157-174.

Pérez Jimeno, L. (2007). Investigaciones arqueológicas en el sector septentrional de la llanura aluvial del Paraná, margen santafesina. La variabilidad del registro arqueológico [Tesis doctoral inédita]. Universidad Nacional de La Plata.

Pérez, M., & Killian Galván, V. (2011). Doncellas (Puna Septentrional, Jujuy, Argentina): Nuevos enfoques a partir del estudio cerámico y del análisis paleodietario. *Estudios Atacameños, 42*, 79-100.

Pérez, S. I., & Monteiro, L. (2009). Nonrandom factors in modern human morphological diversification: A study of craniofacial variation in Southern South American populations. *Evolution, 63*(4), 978-993.

Píccoli, C. (2015). Estudios de los paisajes arqueológicos en el sector de islas y borde frontal de la terraza baja de la llanura aluvial del Paraná Medio. Depto. de Goya [Tesis doctoral inédita]. Universidad Nacional de Rosario.

Podestá, M. M., & Aschero, C. A. (2012). Evidencias tempranas del arte rupestre de los cazadores-recolectores de la Puna (NO de la Argentina). En J. Clottes (Dir.), *L'art pléistocène dans le monde / Pleistocene art of the world / Arte pleistoceno en el mundo* (pp. 773-791). Préhistoire, Art et Sociétés, Bulletin de la Société Préhistorique Ariège-Pyrénées.

Politis, G., & Borrero, L. (2024). *The archaeology of the Pampas and Patagonia*. Cambridge University Press.

Politis, G., & Tissera, L. (2023). Prehispanic macroregional networks between the Southern Andes and the Lower Paraná River of South America. *Journal of Anthropological Research, 79*(5), 307-351.

Politis, G., & Bonomo, M. (2018). Estado actual y perspectivas de Goya-Malabrigo. Una sociedad indígena del Noreste Argentino. En G. Politis & M. Bonomo (Eds.), *Goya-Malabrigo. Arqueología de una sociedad indígena del Noreste Argentino* (pp. 9-44). Editorial UNICEN.

Politis, G. G., Messineo, P. G., Gonzalez, M. E., Álvarez, M. C., & Favier Dubois, C. (2012). Primeros resultados de las investigaciones en el sitio Laguna de los Pampas (partido De Lincoln, provincia de Buenos Aires). *Relaciones de la Sociedad Argentina de Antropología, XXXVII*, 463-472.

Politis, G., & Bonomo, M. (2012). La entidad arqueológica Goya-Malabrigo (Ríos Paraná y Uruguay) y su filiación Arawak. *Revista de Arqueología, 25*(1), 10-46.

Politis, G., & Bonomo, M. (2015). Una revisión del Túmulo de Campana. *Relaciones de la Sociedad Argentina de Antropología, 60*(1), 149-181.

Politis, G., & Bonomo, M. (2023). Goya-Malabrigo y la expansión Arawak 10 años después. *Revista de Arqueología, 36*(1), 1-16.

Politis, G., Barrientos, G., & Stafford, T. (2011). Revisiting Ameghino: New 14C dates from ancient human skeletons from the Argentine Pampas. En D. Vilaou (Ed.), *Peuplements et préhistoire en Amériques* (pp. 43-54). Editorial du CTHS.

Politis, G., Bonomo, M., Castiñeira, C., & Blasi, A. (2011). Archaeology of the Upper Delta of the Paraná River (Argentina): Mound construction and anthropic landscapes in the Los Tres Cerros locality. *Quaternary International, 245*, 74-88.

Politis, G., Bonomo, M., Loperfido, M., Rodríguez, J. M., & Pedersen, O. (2021). Una estructura arquitectónica Goya-Malabrigo en el Delta Superior del río Paraná. *Libro de resúmenes del IX Encuentro de discusión arqueológica del Nordeste*. Resistencia, Argentina.

Politis, G., Martínez, G., & Bonomo, M. (2001). Alfarería temprana en sitios de cazadores-recolectores de la Región Pampeana (Argentina). *Latin American Antiquity, 12*(2), 167-181.

Politis, G., Gutiérrez, M. A., Rafuse, D., & Blasi, A. (2016). The arrival of Homo sapiens into the Southern Cone at 14,000 years ago. *PLoS ONE, 11*(9), 1-27.

Pommarés, N. N. (2017). Noroeste Argentino. En E. E. Fucks & M. F. Pisano (Comps.), *Cuaternario y geomorfología de Argentina: Distribución y características de los principales depósitos y rasgos geomorfológicos* (pp. 170–195). Editorial Facultad de Ciencias Naturales y Museo, Universidad Nacional de La Plata.

Posth, C., Nakatsuka, N., Lazaridis, I., Skoglund, P., Mallick, S., Lamnidis, T. C., Rohland, N., Nägele, K., Adamski, N., Bertolini, E., Broomandkhoshbacht, N., Cooper, A., Culleton, B. J., Ferraz, T., Ferry, M., Furtwängler, A., Haak, W., Harkins, K., Harper, T. K., Hünemeier, T., Lawson, A. M., Llamas, B., Michel, M., Nelson, E., Oppenheimer, J., Patterson, N., Schiffels, S., Sedig, J., Stewardson, K., Talamo, S., Wang, C.-C., Hublin, J.-J., Hubbe, M., Harvati, K., Nuevo Delaunay, A., Beier, J., Francken, M., Kaulicke, P., Reyes-Centeno, H., Rademaker, K., Trask, W. R., Robinson, M., Gutierrez, S. M., Prufer, K. M., Salazar-García, D. C., Chim, E. N., Müller, L., Gomes, P., Alves, M. L., Liryo, A., Inglez, M., Oliveira, R. E., Bernardo, D. V., Barioni, A., Wesolowski, V., Scheifler, N. A., Rivera, M. A., Plens, C. R., Messineo, P. G., Figuti, L., Corach, D., Scabuzzo, C., Eggers, S., DeBlasis, P., Reindel, M., Méndez, C., Politis, G., Tomasto-Cagigao, E., Kennett, D. J., Strauss, A., Fehren-Schmitz, L., Krause, J., & Reich, D. (2018). Reconstructing the deep population history of Central and South America. *Cell, 175*(5), 1185-1197.e22.

Powell, M., & Cook, D. (Eds.). (2005). *The myth of syphilis: The natural history of treponematosis in North America*. University Press of Florida/Florida Museum of Natural History.

Prates, L., & Pérez, I. (2021). Late Pleistocene South American megafaunal extinctions associated with rise of fishtail points and human population. *Nature Communications, 12*(1), 2175.

Raíces Montero, C., Miranda de Zela, P., & Olivera, D. (2015). Aporte al estudio del comportamiento mortuorio de las poblaciones del Periodo Tardío de Antofagasta de la Sierra (Catamarca, Puna Argentina) a través de la bioarqueología y arqueoentomología. En *Libro de resúmenes de la Primeras Jornadas sobre el Altiplano Sur: Miradas Disciplinares*. Facultad de Filosofía y Letras, Universidad Nacional de Buenos Aires (UBA), Centro Universitario Tilcara, Jujuy.

Raffino, R. A. (1983). *Los Inkas del Kollasuyu* (2.ª ed.). Ramos Americana Editora.

Raffino, R. (1999). Las tierras altas del Noroeste. En *Nueva historia de la Nación Argentina. I. La Argentina aborigen. Conquista y colonización*. Editorial Planeta.

Raffino, R. A. (2007). *Poblaciones indígenas en Argentina. Urbanismo y proceso social precolombino* (1.ª ed.). Emecé Editores.

Ramos van Raap, A., & Bonomo, M. (2016). Nuevos estudios de la colección bioarqueológica de los sitios Arroyo Malo, El Cerrillo y Arroyo Sarandí (Delta del Paraná). *Intersecciones en Antropología, vol. especial 3*, 71-82.

Recalde, A., & Rivero, D. (2018). Los primeros habitantes de la provincia de Córdoba. En M. Philp, A. Ceballos, & C. Navarro (Eds.), *Itinerarios: Recorridos por la historia de Córdoba*. Editorial de la UNC; Escuela de Historia FFyH-UNC y Ministerio de Educación, Gobierno de la Provincia de Córdoba.

Reichlen, H. (1940). Recherches archéologiques dans la province de Santiago del Estero (Rép. Argentine). *Journal de la Societé des Amèricanistes, 65*, 133-225.

Restifo, F., Carbonelli, J. P., & Agnolin, A. (2019). Puntas de proyectil de puna y valles mesotermales del noroeste argentino en perspectiva comparativa: Los casos de la cuenca de Pastos Grandes (departamento de Los Andes, Salta) y el valle de Santa María (Catamarca) como aporte para la arqueología de cazadores recolectores. *Andes, Antropología e Historia, 2*(30), 1-28.

Ribero, F. (2017). Sitios arqueológicos sudamericanos-Santa Rosa de Tastil. *Cultura en Red, 2*(2).

Richard, N. (2008). Les chiens, les hommes et les étrangers furieux. Archéologie des identités indiennes dans le Chaco boréal. *Nuevo Mundo Mundos Nuevos*. Thèse soutenue à l'EHESS.

Richard, N., & Combès, I. (2015). O complexo alto-paraguaiense: Do Chaco a Mato Grosso do Sul. En G. Chamorro & I. Combès (Orgs.), *Povos indígenas em Mato Grosso do Sul. História, cultura e transformações sociais* (pp. 231-248). UFGD Editora.

Rivero, D. E. (2012). La ocupación humana durante la transición Pleistoceno-Holoceno (11,000-9000 a.P.) en las Sierras Centrales de Argentina. *Latin American Antiquity, 23*(4), 551-564. https://doi.org/10.7183/1045-6635.23.4.551

Rivero, D. E., Balena, I., Costantino, F., Lallami, C., Álvarez, M., Medina, M. E., Pastor, S., & Sario, G. (2024). Radiocarbon dates, projectile points, and the spread of bow-and-arrow technology in Sierras of Córdoba (Argentina). *Bows and Arrows in South America: Advances and Debates, 704*, 62-68. https://doi.org/10.1016/j.quaint.2023.10.007

Rivero, D. E., Pastor, S., & Heider, G. (2018). The Tigre Projectile Point in Central Argentina: Implications for the Initial Peopling of the Region. *PaleoAmerica, 4*(1), 68-70. https://doi.org/10.1080/20555563.2017.1395257

Ruggeroni, D. (1975). Arqueología del Paraná. Yacimiento de la Isla del Indio. *Publicaciones del Museo Municipal de Reconquista, Nro. 2*.

Ruggeroni, D., & Gallagher, E. (2006). *Historia de Reconquista*. Municipalidad de Reconquista.

Schmitz, P. I., Ceruti, C., González, A. R., & Rizzo, A. (1972). Investigaciones arqueológicas en la zona de Goya (Corrientes, Argentina). *Revista Dédalo, 8*(15), 11-121.

Ruiz, M., & Albeck, M. (1997). El fenómeno pukara visto desde la puna jujeña. *Cuadernos de la Facultad de Humanidades y Ciencias Sociales, UNJu, 9*, 233-255.

Salceda, S. A., & Raffino, R. A. (2004). El hombre de El Shincal. En R. A. Raffino (Ed.), *El Shincal de Quimivíl* (pp. 165-177). Editorial Sarquis.

Salceda, S. A., Desántolo, B., & Plischuk, M. (2015). Espacio de reflexión: El por qué y para quién de la investigación bioantropológica. *Revista Argentina de Antropología Biológica, 17*(2), 1-6.

Salega, M. S., & Fabra, M. (2013). Niveles de actividad física en poblaciones de las sierras y las llanuras de la provincia de Córdoba (Argentina) durante el Holoceno tardío. *Revista Relaciones, XXXVIII*(2), 401-420.

Salega, S., & Fabra, M. (2018). First evidence of elongated styloid process in two female archaeological individuals from Córdoba hills, Argentina (late Holocene). *International Journal of Osteoarchaeology, 28*(4), 458-463. https://doi.org/10.1002/oa.2665

Sario, G., Zarate, P., Tavarone, A., Salega, S., Da Peña, G., & Fabra, M. (2023). Entierros humanos, prácticas de molienda, talla y consumo vegetal. El sitio Arroyo de la Palma 1 como caso de estudio (Holoceno tardío, sierras de Córdoba, Argentina). *InterSecciones En Antropología, 24*(2), 123-142. https://doi.org/10.37176/iea.24.2.2023.800

Sartori, J., & Galligani, P. (2014). Zooarqueología en el humedal de la cuenca fluvial del centro este santafesino. *Relaciones de la Sociedad Argentina de Antropología, 39*(2), 387-409.

Scabuzzo, C., & Ramos Van Raap, M. A. (2017). Nuevos resultados de los estudios osteológicos del sitio Los Tres Cerros 1 (Delta Superior del río Paraná). *Comechingonia. Revista de Arqueología, 21*(2), 201-228.

Scabuzzo, C., Frontini, R., Vecchi, R., & Bayón, C. (2016). Isótopos estables y dieta de los cazadores recolectores del sudoeste bonaerense (Argentina). *Chungara: Revista de Antropología Chilena, 48*(3), 383-396. https://doi.org/10.4067/S0717-73562016005000020

Scabuzzo, C., Ramos van Raap, M. A., Bonomo, M., & Politis, G. (2015). Estudios bioarqueológicos en el sitio Los Tres Cerros 1 (Delta Superior del río Paraná, Entre Ríos, Argentina). *Boletim do Museu Paraense Emílio Goeldi. Ciências Humanas, 10*(2), 509-535.

Scattolin, C. (2010). La organización del hábitat precalchaqui (500 A.C.-1000 D.C.). En M. E. Albeck et al. (Eds.), *El hábitat prehispánico. Arqueología de la arquitectura y de la construcción del espacio organizado* (pp. 13-51). Edunju.

Scheifler, N. A., Messineo, P. G., Boscheren, H., & Politis, G. G. (2024). Isotopic evidence of diet breadth hunter-gatherers changes during the Holocene in the Central Pampean Dunefields (Argentina, South America). *American Journal of Biological Anthropology, 185*(4), e25039. https://doi.org/10.1002/ajpa.25039

Schmitz, P., Ceruti, C., González, A. R., & Rizzo, A. (1972). Investigaciones arqueológicas en la zona de Goya (Corrientes) Argentina. *Dédalo, 15*, 11-121.

Seldes, V. (2006). Bioarqueología de poblaciones prehistóricas de la quebrada de Humahuaca (Jujuy, Argentina). *Estudios Atacameños, 31*, 47-61.

Serrano, A. (1945). Los comechingones. *Serie Aborígenes Argentinos, Vol. I*. Instituto de Arqueología, Lingüística y Folklore, Universidad Nacional de Córdoba.

Serrano, A. (1958). *Manual de Cerámica Indígena*. Assandri.

Serrano, A. (1950). Los primitivos habitantes de Entre Ríos. *Serie II*. Paraná: Ediciones de la Biblioteca Entrerriana "General Perón".

Silva, L. C. R., Giorgis, M. A., Anand, M., Enrico, L., Perez-Harguindeguy, N., Falczuk, V., Tieszen, L. L., & Cabido, M. (2011). Evidence of shift in C4 species range in central Argentina during the late Holocene. *Plant and Soil, 349*(1-2), 261-279. https://doi.org/10.1007/s11104-011-0868-x

Somonte, C., & Baied, C. A. (2017). Ocupaciones humanas de finales del Pleistoceno en valles intermontanos del Noroeste Argentino. *Materialidades. Perspectivas actuales en cultura material, 5*, 1-21.

Susnik, B. (1959). Material arqueológico del área alto-paraguayense. *Boletín de la Sociedad Científica del Paraguay y del Museo Etnográfico, Vol. III, Miscelánea 1*, 81-103.

Susnik, B. (1972). Dimensiones migratorias y pautas culturales de los pueblos del gran Chaco y de su periferia (enfoque etnológico). *Suplemento Antropológico, Vol. VII, N° 1-2*, 85-107.

Taboada, C. (2014). Sequía Vieja y los Bañados de Añatuya en Santiago del Estero. Nodo de desarrollo local e interacción macrorregional. *Comechingonia, Revista de Arqueología, 18*(1), 93-116. https://doi.org/10.37603/2250.7728.v18.n1.27628

Taboada, C. (2016). Montículos arqueológicos, actividades y modos de habitar. Vivienda y uso del espacio doméstico en Santiago del Estero (tierras bajas de Argentina). *Arqueología de la Arquitectura, 13*, e040. https://doi.org/10.3989/arq.arqt.2016.003

Taboada, C. (2017). Espacio, cultura material y procesos sociales tardíos en la llanura santiagueña. Modelo para pensar a las poblaciones de la región. En B. Ventura, G. Ortiz, & B. Cremonte (Eds.), *Arqueología de la vertiente oriental Surandina: Interacción macro-regional, materialidades, economía y ritualidad* (pp. 237-266). Sociedad Argentina de Antropología.

Taboada, C. (2019). Procesos sociales prehispánicos y pericoloniales en torno a los ríos Salado y Dulce (Santiago del Estero, Argentina). *Revista del Museo de La Plata, 4*(2), 511-540. https://doi.org/10.24215/25456377e087

Taboada, C., & Angiorama, C. (2010). Metales, textiles y cerámica. Tres líneas de análisis para pensar una vinculación entre los habitantes de la llanura santiagueña y el Tawantinsuyu. *Memoria Americana, 18*(2), 11-41.

Taboada, C., & Angiorama, C. (2021). Tras los sitios de los pioneros y algo más. Prospecciones en la llanura de Santiago del Estero (Argentina) y aportes a problemáticas de investigación regional. *Arqueología, 27*(1), 41-67. https://doi.org/10.34096/arqueologia.t27.n1.7625

Taboada, C., & Farberman, J. (2014). Asentamientos prehispánicos y pueblos de indios coloniales sobre el río Salado (Santiago del Estero, Argentina). *Revista Latinoamericana de Arqueología Histórica, 8*(1), 7-44.

Tarragó, M. N. (2000). Chacras y pukara. Desarrollos sociales tardíos. En M. N. Tarragó (Dir.), *Nueva Historia Argentina. Los pueblos originarios y la conquista* (Tomo 1, pp. 255-300). Sudamericana.

Tavarone, A., Ramírez, D., González, C. V., Colobig, M., Nores, R., & Fabra, M. (2020a). Multidisciplinary perspectives for the study of ancient diet and oral health. A case study from the Central Region of Argentina. *Archaeology and Anthropology Science, 12*, 47. https://doi.org/10.1007/s12520-019-00965-7

Tavarone, A., González, C. V., Salega, S., Bellis, J., Benedetti, F., Ramírez, D., Loupias, L., & Fabra, M. (2016). Análisis bioarqueológico de restos humanos recuperados en el sitio La Para 1387/15 (Departamento Río Primero, Córdoba). *Revista Memorias del Pueblo, del Museo Histórico Municipal de La Para, Año 3*(3), 5-21.

Tavarone, A. M., Colobig, M. de los M., & Fabra, M. (2020b). Estudio de dieta en poblaciones arqueológicas del centro de Argentina a través del análisis de microrrestos vegetales e isótopos estables. *InterSecciones en Antropología, 21*(2), 213-228. https://doi.org/10.37176/iea.21.2.2020.556

Tavarone, A., Dantas, M., & Fabra, M. (2017). Tafonomía de restos óseos humanos arqueológicos en ambientes lacustres. El caso del sitio El Diquecito (Laguna Mar Chiquita, Córdoba, Argentina). *Revista Cuadernos del Instituto de Antropología y Pensamiento Latinoamericano, 25*(2), 191-210.

Tavarone, A., Colobig, M. de los M., & Fabra, M. (2023). Primeros resultados de estudios arqueobotánicos sobre fragmentos cerámicos del sitio Isla Orihuela (Laguna Mar Chiquita, Córdoba). *Revista Folia Histórica del Nordeste, 46*, 269-282. https://doi.org/10.30972/fhn.4606503

Tavarone, A., Colobig, M. de los M., Dantas, M., & Figueroa, G. G. (2023). De la planta a la boca: Identificación de restos vegetales en los sitios El Polear 1 y Uturunco 1 a partir del análisis de cálculo dental humano. *Relaciones, 48*(2), 245-263.

Tibayrenc, M. (2007). Human genetic diversity and the spread of infectious diseases. En M. Tibayrenc (Ed.), *Encyclopedia of infectious diseases-modern methodologies* (pp. 321-35). John Wiley.

Togo, J. (2004). Arqueología Santiagueña: Estado actual del conocimiento y evaluación de un sector de la cuenca del Río Dulce [Tesis doctoral inédita]. Facultad de Ciencias Naturales y Museo, Universidad Nacional de La Plata.

Togo, J. (2007). Las Mercedes: Los primeros fechados radiocarbónicos. *Indoamérica. Nueva Serie Científica, 1*(1), 51-79.

Torres, L. M. (1907). Arqueología de la cuenca del río Paraná. *Revista del Museo de La Plata, 14*, 53-122.

Torres, L. M. (1911). Los primitivos habitantes del Delta del Paraná. *Biblioteca Centenaria, 4*. Universidad Nacional de La Plata.

Torres, L. M., & Ameghino, C. (1913a). Investigaciones antropológicas y geológicas en el litoral marítimo sur de la provincia de Buenos Aires. *Physis, 5*(1), 261-264.

Torres, L. M., & Ameghino, C. (1913b). Informe preliminar sobre las investigaciones geológicas y antropológicas en el litoral marítimo sur de la provincia de Buenos Aires. *Revista del Museo de La Plata, 20*(7), 153-167.

Torres-Rouff, C., & Costa-Junqueira, M. (2006). Interpersonal violence in prehistoric San Pedro de Atacama, Chile: Behavioral implications of environmental stress. *American Journal of Physical Anthropology, 130*, 60-70.

Urquiza, S. V., & Babot, M. P. (2018). Ofrendar y propiciar. Fauna y prácticas agropastoriles prehispánicas del segundo milenio AP en Antofagasta de la Sierra, Puna meridional Argentina. *Archaeofauna, 27*, 209-232.

Vignati, M. A. (1960). El indigenado de la Provincia de Buenos Aires. *Anales de la Comisión de Investigaciones Científicas de la Provincia de Buenos Aires, 1*, 95-182.

Violante, R. A., Parker, G., & Cavallotto, J. L. (2001). Evolución de las llanuras costeras del este bonaerense entre la bahía Samborombón y la laguna Mar Chiquita durante el Holoceno. *Revista de la Asociación Geológica Argentina, 56*(1), 51-66.

von Hauenschild, J. (1949). Ensayo de clasificación de la documentación arqueológica de Santiago del Estero. *Revista de la Universidad Nacional de Córdoba*, 36, 7-75.

Wagner, E., & Wagner, D. (1934). *La Civilización Chaco-Santiagueña y sus correlaciones con las del Viejo y Nuevo Mundo*. Tomo I. Compañía Editora Argentina S.A.

Williams, V., & D'Altroy, T. (1998). El sur del Tawantinsuyu: Un dominio selectivamente intensivo. *Tawantinsuyu*, 5, 170-178.

Williams, V., Villegas, M. P., Gheggi, M. S., & Chaparro, M. G. (2005). Hospitalidad e intercambio en los valles mesotermales del Noroeste argentino. *Boletín de Arqueología PUCP*, 9, 335-372.

Yacobaccio, H. D. (2004). Social dimensions of camelid domestication in the southern Andes. *Anthropozoologica, 39*(1), 237-247.

Yacobaccio, H. D. (2006). Intensificación económica y complejidad social en cazadores-recolectores surandinos. *Boletín De Arqueología PUCP, 10*, 305-320.

Yacobaccio, H. D. (2017). Peopling of the high Andes of northwestern Argentina. *Quaternary International, 461*, 34-40.

Yacobaccio, H. D. (2021). The domestication of South American camelids: A review. *Animal Frontiers, 11*(3), 43-51.

Yacobaccio, H. D., Cata, M. P., Solá, P., & Alonso, M. S. (2008). Estudio arqueológico y fisicoquímico de pinturas rupestres en Hornillos 2 (Puna de Jujuy). *Estudios Atacameños: Arqueología y Antropología Surandinas, 36*, 5-28.

Zabala, M. E., Salega, S., Tavarone, A., & Fabra, M. (2023). De la exhumación al proceso de devolución en Cerro Colorado (provincia de Córdoba): Un abordaje intercultural, transdisciplinar e interinstitucional. *Anuario TAREA, 10*(10), 46-74. https://revistasacademicas.unsam.edu.ar/index.php/tarea/article/view/1374

Zárate, M. A. (2016). Explorando la historia geológica del Alero Deodoro Roca. En R. Cattáneo & A. D. Izeta (Eds.), *Arqueología en el Valle de Ongamira, 2010-2015* (pp. 43-56). Universidad Nacional de Córdoba.

Zárate, P., Fabra, M., Tavarone, A., & González, C. (2021). Ser mujer en el norte del valle de Punilla a inicios del primer milenio (Sitio El Vado, Córdoba). Aportes desde la bioarqueología social. *Comechingonia. Revista De Arqueología, 26*(2), 5-28. https://doi.org/10.37603/2250.7728.v26.n2.34364

Zarza, M., del Papa, L., & Lamenza, G. (2019). Variación temporal en la estructura de los recursos en un sitio del Chaco húmedo argentino. *Comechingonia, 23*(2), 37-58.

Zarza, M., Panzeri, K., Larsen, T., del Papa, L., & Lamenza, G. (2024). Morphogeometric analysis of tooth plates of Lepidosiren paradoxa (Sarcopterygii, Dipnoi) to infer Late-Holocene paleoenvironmental variations in the South American Gran Chaco. *The Holocene*. https://doi.org/10.1177/09596836241285796

Zeballos, E., & Pico, P. (1878). Informe sobre el túmulo prehistórico de Campana. *Anales de la Sociedad Científica Argentina, 6*, 244-260.

SI Section 7. Bulleted summary of the main results shared with Indigenous communities (when present or identified), rural localities, regional Indigenous councils (such as the Consejo de Comunidades de Pueblos Indígenas de la Provincia de Córdoba), and other stakeholders.

Estimados/as,

Les escribimos estas líneas porque nos parece importante compartir con ustedes los principales resultados de un trabajo de investigación que estamos llevando a cabo junto con investigadores e investigadoras de distintas universidades de Argentina y de Estados Unidos.

Desde hace más de 7 años, nos hemos puesto en contacto con arqueólogos, arqueólogas y trabajadores de museos que estudian el pasado de los pueblos originarios americanos. Junto con ellos/as nos propusimos investigar los vínculos genéticos de los grupos humanos de distintas regiones de lo que hoy es Argentina. Hemos analizado información genética de 238 personas de tiempos pre-hispánicos de la Región Chaqueña, el Litoral, la Región Pampeana, el Noroeste y el Centro de Argentina, incluyendo muchas de Córdoba, y uno de Paraguay, en un período que va desde hace 150 a 10.000 años atrás; y la hemos comparado con datos disponibles de otras poblaciones de América.

Explicaremos brevemente qué es lo que analizamos y cómo los datos genéticos nos sirven para responder a algunas preguntas tales como: ¿cuál es la antigüedad de una población en una determinada región?; ¿cuál fue la ruta utilizada por las poblaciones humanas?; ¿en qué medida las poblaciones de distintas regiones se han mezclado? Una vez que las poblaciones se asentaron en una determinada región, ¿hubo continuidad?; ¿hubo llegada de personas de otras regiones?

A partir de dientes o huesos antiguos, es posible extraer el ADN y realizar una lectura de la secuencia de bases que lo componen (llamadas A, C, G y T). Cada individuo posee una secuencia única, es decir que el orden en que se disponen estas cuatro letras es diferente para cada ser humano, pero es más parecido entre aquellas personas que están más cercanamente emparentadas, o como nos gusta decir a los/as genetistas, que comparten un ancestro común más reciente. Analizando cientos de miles de estas bases presentes en el genoma, podemos estimar los procesos de diferenciación y los vínculos entre las poblaciones humanas, rastreando los caminos que la humanidad ha seguido desde su origen en África, la llegada a América desde Asia y el poblamiento de las distintas regiones de Sudamérica.

Este estudio de las similitudes y diferencias genéticas es un proceso que no está acabado y que constantemente se revisa a partir de nuevas investigaciones, que incorporan nuevos datos, o nuevos modos de analizar los datos. Por esta razón, el conocimiento científico no debe tomarse como una verdad indiscutible, sino justamente como una interpretación que constantemente es puesta a prueba. Lo que aquí presentamos es el conocimiento al que hemos llegado hoy, con los datos disponibles y que representa la mejor interpretación que hemos alcanzado desde nuestra perspectiva basada en el conocimiento científico. Con esto no pretendemos imponer nuestro punto de vista ni negar otros modos de conocimiento, sino que nos parece importante también compartir

el fruto de nuestro trabajo con ustedes para fomentar y potenciar el diálogo de saberes. Esperamos que, luego de la lectura de esta carta, puedan compartir con nosotros/as sus pareceres, dudas e inquietudes. Quedamos en contacto y desde ya, muchas gracias por el tiempo dedicado.

Título del trabajo: "*Genomas antiguos del Centro del Cono Sur documentan la continuidad de ocho milenios y las interacciones de un linaje sudamericano desconocido*".

Principales resultados:

- Analizamos la información genética de una persona que vivió hace 10.000 años en el territorio que actualmente corresponde al norte de la provincia de Buenos Aires y encontramos que la población a la que perteneció está genéticamente más vinculada a los grupos humanos que posteriormente habitaron el sur de Sudamérica (incluyendo la Región Pampeana, el Centro de Argentina y el sur de Patagonia). Los datos analizados muestran que hace 10.000 años los habitantes de estas regiones ya se habían diferenciado genéticamente de los habitantes de otras regiones de Sudamérica, como los Andes Centrales o el centro-este de Brasil, indicando así una continuidad poblacional, al menos parcial, de miles de años en la región.
- Por otra parte, la información genética de otra persona que vivió hace 8.800 años en la Puna de Catamarca, en el territorio que actualmente se denomina Antofagasta de la Sierra, nos indica que la población a la que perteneció estaba genéticamente más emparentada con las poblaciones que en ese mismo momento habitaban la región de los Andes Centrales (actuales Perú y Bolivia).
- Uno de los resultados más significativos de nuestro trabajo es que las poblaciones originarias del Centro del actual territorio argentino descienden de habitantes que ya vivían en esta región desde hace al menos 8.500 años. La evidencia de esto la constituye una persona que vivió en ese momento, cerca de lo que actualmente es la ciudad de Jesús María, en la provincia de Córdoba. Es decir, todos los individuos analizados que habitaron la región posteriormente son genéticamente más similares a este individuo de Jesús María que a representantes de otros linajes sudamericanos. Esta continuidad genética de más de 8 milenios llega hasta el presente, ya que pudimos determinar la presencia de este linaje desconocido, propio del Centro del Cono Sur, en poblaciones actuales de la misma región.
- También encontramos que las poblaciones que habitaron los territorios de las actuales provincias de Córdoba, Santiago del Estero, Santa Fe, Entre Ríos y la zona del Delta del Paraná en la provincia de Buenos Aires (territorios a los que en conjunto denominamos como Centro del Cono Sur) son muy semejantes desde el punto de vista genético, ya que comparten mayoritariamente este nuevo linaje, mientras que las poblaciones antiguas de la Puna de Salta y Jujuy son más parecidas genéticamente a las de los Andes Centrales.
- En el Noroeste Argentino, en la zona de los Valles Subandinos y en el sur de la Puna (territorios que actualmente corresponden a las provincias de Catamarca y Tucumán), los datos que analizamos nos permiten afirmar que, desde el punto de vista genético, las

poblaciones se pueden caracterizar como una mezcla entre el componente de los Andes Centrales y el del Centro del Cono Sur.

- No nos fue posible determinar cuál o cuáles fueron las rutas por las que los primeros habitantes llegaron al Centro del Cono Sur, ya que no pudimos identificar similitudes entre las poblaciones del Centro de Argentina y las poblaciones de los Andes Centrales (lo que indicaría un poblamiento desde el oeste siguiendo la costa del Pacífico), y tampoco con las de los bosques tropicales de Brasil (lo que sugeriría un ingreso bordeando el Atlántico). Lo que interpretamos a partir de esta dificultad para distinguir por dónde llegaron, es que el poblamiento de esta región fue muy temprano, hace más de 12.000 años, y muy rápido. Es decir, que se pobló al poco tiempo de la entrada de los primeros grupos humanos a Sudamérica, cuando las poblaciones, que posteriormente se asentaron en zonas más cercanas al Pacífico y al Atlántico, aún no se habían diferenciado genéticamente.

- También pudimos describir que las poblaciones que hace 7.000 años habitaban la Región Pampeana eran más parecidas genéticamente a las del centro de Chile, pero que hace al menos 3.300 años, estas poblaciones entraron en contacto con las poblaciones del Centro de Argentina (especialmente con las de las Sierras Pampeanas de Córdoba), generándose un proceso de mestizaje que resultó en un cambio en la composición genética de los habitantes de la Región Pampeana. Este cambio no consistió en un reemplazo de la población, sino que hubo una mezcla entre las poblaciones con más tiempo de permanencia en la región y las que llegaron posteriormente desde la región central de Argentina.

- Por otra parte, las antiguas poblaciones de las actuales provincias de Chaco y Formosa y del norte de Paraguay se distinguen de las mencionadas anteriormente, indicando que hubo un relativo aislamiento biológico de estos grupos, más vinculados genéticamente con poblaciones de los bosques tropicales y subtropicales de Sudamérica. Sin embargo, en un individuo de 200 años de antigüedad de Chaco, así como en Tobas y Wichís actuales, se observa un patrón de mestizaje entre dicho componente de los bosques tropicales y el de la región central, revelando continuidad genética de más de 2.000 años y movimientos poblacionales recientes.

Todas estas similitudes o diferencias señaladas no se asocian directa ni necesariamente con otras características culturales tales como la lengua, el tipo de asentamiento, las formas de subsistencia o las características de los objetos materiales, tales como el estilo cerámico o de las herramientas. Nuestros resultados pertenecen a la esfera de la biología, siendo los mecanismos de herencia y diferenciación genética distintos de los mecanismos mediante los cuales se transmiten las prácticas culturales y simbólicas.

SI Section 8 Preprocessing of genotype data

8.1 Assessment of contamination or post-colonial admixture

Apart from the contamination estimation using `contamMix` [3], `hapConX` [4] and `ANGSD` [5] (see Methods), we conducted an additional step to identify individuals with evidence of contamination or post-colonial admixture. We ran a direct `qpAdm` analysis in which we tested the presence of non-Native American ancestry on a per-individual basis. For each individual, we considered a single-source model of Native American ancestry (taking *Dominican_Ceramic*, a pre-European contact ancient American population with tens of well-covered individuals, as a well-powered representative), including European (represented by *French*), East Asian (represented by *Han*) and African (represented by *Mbuti* and *YRI*) populations on the right.

A model of 100% Native American ancestry was rejected for 11 individuals at $p < 0.01$ (Supplementary Data 1). These individuals were excluded from analysis. Tests were conducted with the `R` library `ADMIXTOOLS2` [6, 7] (version 2.0.0), setting `allsnps=TRUE`.

8.2 Exclusion of relatives up to 2nd degree

To avoid unsound inferences of population history coming from biased allele-frequency variance estimates, we removed relatives up to 2nd degree from all population-level analyses, while keeping them in individual-level analyses. We first chose the higher-coverage individual within the single pair of duplicates, and we then plotted the network of up-to-2nd degree relatedness in our dataset (Supplementary Figure 84), and visually inspected it to identify a minimal vertex cover (that is, a set of vertices of the graph that will cause, upon being removed, no two remaining vertices being adjacent to each other). In other words, we sought to remove the minimal number of possible individuals so that no pair of relatives up to 2nd degree remained in the dataset. We proceeded as follows, for the two unique cases we encountered:

(a) Case 1: A pair of isolated relatives: we kept the higher-coverage individual of the two.

(b) Case 2: A trio of pairwise-connected relatives linked to a pair: we kept the higher-coverage individual within the trio (`I12758`) and the higher-coverage individual within the pair (`ARG_22640`).

SI Section 9 Interrogation of deep lineages in the Early and Middle Holocene Central Southern Cone

To explore the genetic affinity of the oldest individuals in our dataset to previously-reported deep lineages relevant to the population history of South America, we combined semi-automatic and automatic approaches.

We started from the following set of previously-published individuals:

```
USA_Ancient_Beringian.SG (to serve as an outgroup)
USA_Anzick_realigned.SG
Chile_LosRieles_12000BP.SG
Chile_LosRieles_5100BP.AG
Brazil_MG_C_Sumidouro_EH_HG_10100BP.SG
Brazil_Capelinha_Sambaqui_10400BP.AG
Brazil_MG_C_LapaDoSanto_EH_HG_9600BP.AG
Brazil_LocaDoSuin_Sambaqui_9100BP.AG
Brazil_SP_S_Laranjal_MH_HG_6800BP.AG
Peru_Cuncaicha_9000BP.AG
Peru_Lauricocha_8600BP.AG
Chile_PuntaSantaAna_7300BP.SG
Chile_WesternArchipelago_Ayayema_5100BP.SG
Argentina_NorthTierradelFuego_LaArcillosa2_5800BP.AG
Argentina_ArroyoSeco2_7700BP.AG
Argentina_Pampas_6800BP.AG
```

which we combined with the following 3 newly-reported Early/Middle Holocene individuals from this study:

```
Argentina_Pampas_CentralPampeanDunefields_LagunadelosPampas_10000BP
Argentina_Northwest_SouthernPuna_Antofagasta_PeñasdelasTrampas1.1_8800BP
Argentina_Central_Hills_SierrasChicas_JesusMaria_8500BP
```

and we computed a neighbor-joining tree from a genetic distance matrix, constructed by inverting pairwise outgroup f_3 statistics, with *YRI.DG* (Yoruba population from the 1000G project [8]) as an outgroup (Supplementary Data 2). We used a custom script implemented in `R` [9] (version 4.2.1), making use of libraries `ape` [10] (version 5.8), to work with phylogenetic trees; and `spaa` [11] (version 0.2.2), for similarity table to similarity matrix conversion. We rooted trees at *USA_Ancient_Beringian.SG*.

To understand if this tree was well-supported by the data, we implemented a custom script, in `python` (version 3.9.5) [12], making use of libraries `ete3` [13] (version 3.1.3), to work with phylogenetic trees; `argparse` (version 1.1), to parse command-line arguments; `itertools`, for efficient looping and iterator functions; and `sys`, for system-specific parameters and functions.

This script, available at `https://github.com/javiermaravall/aDNA_CSC/`, takes as input a phylogenetic tree in Newick format [14] and a table of f_4 statistics of the form

$$f_4(\text{Outgroup}, \text{Pop}_1, \text{Pop}_2, \text{Pop}_3), \tag{2}$$

where Pop_i, for $i \in \{1, \ldots, 3\}$, represent all populations present in the input tree (Supplementary Data 2). It traverses the tree, going up one level at each step, looking for statistics from the table that indicate a violation of the cladal structure encoded in the tree at the current level.

Using this script, we found a great number of statistics that indicated violations of the outgroup f_3 tree, most of them involving *Brazil_MG_C_Sumidouro_EH_HG_10100BP.SG*. We reasoned that many of them could be driven by artifactual attraction due to the mixture of shotgun and capture data types. Thus, we removed this individual from the tree and re-examined the output of the script. Although the number of violations was greatly reduced, we still observed a large number of them, indicated by many statistics involving *USA_Anzick_realigned.SG* and *Chile_LosRieles_12000BP.SG*. We reasoned that this surely had to do with differential affinity to Anzick [15] among early South Americans, as shown by previous studies [16, 17]. This differential affinity has proven hard to incorporate into population-history models [16], and this is again further complicated by the mixture of data types. Therefore, we chose to reduce modeling complexity by excluding these two individuals from the tree.

When testing the remaining tree, clear patterns emerged, revealing a set of clades that were not rejected by f_4 statistics at a significance threshold of $|Z| = 3$ (Supplementary Data 2). In particular:

```
1. Chile_WesternArchipelago_Ayayema_5100BP.SG,
Chile_PuntaSantaAna_7300BP.SG and
Argentina_NorthTierradelFuego_LaArcillosa2_5800BP.AG
appeared to be a clade.

2. Peru_Cuncaicha_9000BP.AG appeared to be a clade with
Peru_Lauricocha_8600BP.AG.

3. Argentina_ArroyoSeco2_7700BP.AG appeared to be a clade with
Argentina_Pampas_6800BP.AG.

4. Brazil_Capelinha_Sambaqui_10400BP,
Brazil_Laranjal_6800BP,
Brazil_LapaDoSanto_9600BP and Brazil_LocaDoSuin_Sambaqui_9100BP
appeared to be a clade.
```

In the cases of Southern Patagonia (1) and Central East Brazil (4), a particular branching order was not unambiguosly suggested by the data, so we make no statement in that regard in those cases.

We note that these putative clades exhibit very good agreement with geography, and are also in line with previous studies on the genetic history of South America [18], [16], [19], [20], [21], [17].

For these reasons, to both increase power and reduce the burden of model exploration, we merged the putative clades above into the following common labels:

1. Brazil_CentralEast(6800-10400BP): containing
Brazil_Capelinha_Sambaqui_10400BP,
Brazil_Laranjal_6800BP,
Brazil_LocaDoSuin_Sambaqui_9100BP
and
Brazil_LapaDoSanto_9600BP.

2. Peru_CentralAndes(8600-9000BP): containing
Peru_Cuncaicha_9000BP
and
Peru_Lauricocha_8600BP.

3. Argentina_Pampas(6800-7700BP): containing
Argentina_Pampas_ArroyoSeco2_7700BP
and
Argentina_Pampas_LagunaChica_6800BP.

4. SouthernPatagonia(5100-7300BP): containing
Chile_PuntaSantaAna_7300BP,
Argentina_TierradelFuego_LaArcillosa2_5800BP
and
Chile_WesternArchipelago_Ayayema_5100BP.

We combined these putative clades with remaining individuals that were not identified as part of any clade, for automatic population-history model exploration. We used the R library ADMIXTOOLS2 [6, 7] (version 2.0.0). To extract data, using function extract_f2, we allowed maxmiss= 0.15, which resulted in keeping 329279 SNPs, 293834 of which polymorphic among the studied populations. Although the recommended value of this parameter is 0 for use with automatic population-history model exploration, lower values of allowed missingness resulted in a very small number of SNPs being retained (less than 30000), which we deemed inappropriate for robust inferences.

We launched 100 independent iterations of the function find_graphs, for each of $n = 0, 1$ admixture events, which departs from a given set of populations and explores admixture graphs until the resulting graph cannot be made to better fit the data by any manipulation. Because this search can get stuck in local optima, the execution of a large number of independent iterations, each departing from a randomly-initialized admixture graph, is motivated by an effort to better characterize the set of optimally-fitting graphs. For each n and each iteration, we recorded the hash (unique topology identifier), score (a measure of fit) and worst residual ($Z-$score for the largest deviation between observed f_4-statistics and the value predicted by the model).

For each n, we gathered all final models with a unique hash, and we aggregated these across values of n. This resulted in a set of 52 unique models (Supplementary Data 2; full results are available at https://github.com/javiermaravall/aDNA_CSC/). To understand if some elements of this set better fitted the data than others, we tested, for each

pair of models, whether the scores were significantly different. To this end, we used the functions `qpgraph_resample_multi` and `compare_fits`, which perform this test using a combination of holding out data and SNP block bootstrap resampling, to account for both differences in model complexity and potential differences in scores due to chance alone.

We found no pair producing a significant $p-$value, even before multiple-hypothesis-testing correction (Supplementary Data 2). In view of the lack of evidence for invoking a higher number of admixture events resulting in a better fit of models to the data, we chose not to explore models with a number of admixture events greater than 1.

Furthermore, we decided to restrict to $n = 0$ when inspecting shared features of models. We provide the 9 unique winning models for $n = 0$ in Supplementary Figures 2 to 10 (range of scores: $34.067 - 43.379$, range of worst residuals: $2.864 - 4.750$). Notably, for all of these models, all internal branches had a drift value of either 0 (indicating an inability to decide the order of splits) or 1 or 2 (indicating very thin evidence supporting that branch). These results are consistent with previous studies that have found it difficult to identify the timing and order of splits in South America, plausibly due to a rapid radiation in its peopling [16–18]. However, we note that most of the newly-reported Late Holocene individuals tended to show attraction to Southern Patagonia, the Central Andes, and the Middle Holocene Pampas individuals with respect to the Central East of Brazil (see Supplementary Information, Part II, Section 11), rendering it plausible that the Central East Brazil lineage represents the deepest split, a hypothesis that is in line with models proposed by previous work [21].

All 9 models shared the following new clade, not previously identified:

1. `Argentina_Northwest_Puna_Antofagasta_PeñasdelasTrampas1.1_8800BP with Peru_CentralAndes(8600-9000BP).`

Additionally, 8 of 9 models (with the only exception of model 7, the one with the largest score) shared the following clade:

2. `Chile_LosRieles_5100BP.AG with Argentina_Pampas(6800-7700BP).`

The first clade was not rejected by f_4 statistics ($|Z| < 1.6$ for all Pop_2 in Expression 2 when $\text{Pop}_3 =$ *Argentina_Northwest_PeñasdelasTrampas_8800BP* and $\text{Pop}_4 =$ *Peru_CentralAndes(8600-9000BP)*, Supplementary Data 2).

The second clade was not rejected by f_4 statistics ($|Z| < 2.27$ for all Pop_2 in Expression 2 when $\text{Pop}_3 =$ *Argentina_Pampas(6800-7700BP)* and $\text{Pop}_4 =$ *Chile_LosRieles_5100BP*, Supplementary Data 2).

Despite this agreement across well-fitting models, we regard these clades as potentially low-confidence, due to the fitted drift value on these branches being very low.

As for the individual *Argentina_LagunadelosPampas_10000BP*, 5 of the 9 trees positioned it as a clade with *Argentina_JesusMaria_8500BP*, 3 did not make it part of any clade, and the remaining one positioned it as a clade with *Argentina_Pampas(6800-7700BP)*. To better understand the patterns of affinity of this individual with other Early/Middle Holocene South Americans, we inspected statistics of the form in Expression 2, with the former in the second position (Supplementary Data 2, Extended Data Figure 2a). This

revealed significant genetic attraction between *Argentina_LagunadelosPampas_10000BP* and *Argentina_JesusMaria_8500BP*, *SouthernPatagonia(5100-7300BP)* and *Argentina_Pampas(6800-7700BP)*, both with respect to *Brazil_CentralEast(6800-10400BP)* and *Peru_CentralAndes(8600-9000BP)*. At the same time, however, each one of the pairs resulting from the former 3-group set was consistent with being symmetrically related to *Argentina_LagunadelosPampas_10000BP*, suggesting that this individual was genetically equidistant to all three of them, up to the limits of our resolution.

This highly interesting pattern suggests a scenario in which *Argentina_LagunadelosPampas_10000BP* has a shared history with these three context labels (like a more recent common ancestor) to the exclusion of *Brazil_CentralEast(6800-10400BP)* and *Peru_CentralAndes(8600-9000BP)*, nonetheless without contributing asymmetrically to any one of them. However, we found statistics indicating that both *Argentina_JesusMaria_8500BP* and *Argentina_Pampas(6800-7700BP)* show excess allele-sharing with *Brazil_CentralEast(6800-10400BP)* with respect to *SouthernPatagonia(5100-7300BP)* ($|Z| = -2.397, 3.44$, respectively). Because Southern Patagonia individuals were mostly sequenced using shotgun technology, unlike the rest, whose data type is 1240k in-solution enrichment, we regard them as likely statistical artifacts of similar data types being biased towards attracting each other.

We summarized these results in the conservative tree from Figure 2b, flagging low-confidence branches with ★. Because the position of *Argentina_LagunadelosPampas_10000BP* was ambiguous across well-fitting models, we excluded it from the tree.

Finally, we evaluated the affinities of our populations to Anzick. As previously reported, *LosRieles_12000BP* showed the strongest affinity [16] ($|Z| < 4.1$), followed by weaker attraction by *LagunadelosPampas_10000BP* ($|Z| < 2.6$) (Supplementary Data 2). Despite this weaker attraction on the part of *LagunadelosPampas_10000BP* as measured by f_4 statistics, these three individuals were positioned together as a clade in an outgroup f_3 neighbor-joining tree (Supplementary Figure 1), suggesting that *LagunadelosPampas_10000BP* did harbor a distinct Anzick-related genetic component.

9.1 Differential attraction to Mesoamerican and Northern South American populations

Because of previous reports of asymmetrical relatedness to Mesoamerican populations among ancient and present-day South Americans [16, 18], we tested for excess-allele sharing with ancient and modern populations among early Southern Cone individuals. In particular, we computed statistics of the form in Expression 2, with Pop_1 an ancient or modern American population not sampled in present-day Peru, Bolivia, Argentina, Chile, Brazil, Paraguay or Uruguay; and $\text{Pop}_{2,3}$ coming from the set

```
Chile_LosRieles_12000BP

Argentina_Pampas_LagunaPampas_10030BP

Brazil_LapaDoSanto_9600BP.AG
Brazil_Laranjal_6800BP.AG
```

```
Peru_Cuncaicha_9000BP.AG
Peru_Lauricocha_8600BP.AG
Peru_Lauricocha_5800BP.AG

Argentina_Central_JesusMaria_8500BP

Argentina_ArroyoSeco2_7700BP.AG
Argentina_LagunaChica_6800BP.AG

Chile_PuntaSantaAna_7300BP
Argentina_NorthTierradelFuego_LaArcillosa2_5800BP

Chile_LosRieles_5100BP.AG
```

We included the additional individual *Peru_Lauricocha_5800BP* because it was consistent with being cladal with *Peru_Cuncaicha_9000BP* with respect to Early/Middle Holocene South American individuals (largest $|Z| < 2.09$; Supplementary Data 2), and it represented a further early data point in the Southern Cone.

We found statistics indicating excess allele-sharing, mainly between *Chile_LosRieles_5100BP* and *Peru_Lauricocha_5800BP*, and a range of present-day ($2.5 < |Z| < 3.6$) and ancient ($2.8 < |Z| < 4.2$) Mesoamerican, Caribbean and Californian populations (the latter with evidence of Mesoamerican gene flow [22]), mostly with respect to *Brazil_LapaDoSanto_9600BP* and *Argentina_LagunadelosPampas_10000BP*, but also *Chile_PuntaSantaAna_7300BP* and Middle Holocene Pampas (Supplementary Data 2).

These results suggest the possibility of gene flow between Mesoamerican-related and Southern Cone populations at a late point during the Middle Holocene. Furthermore, the observation of similar patterns in at least two individuals from different parts of the Southern Cone that carry ancestries already differentiated from each other by the Middle Holocene suggests these findings are driven by gene flow into South America, and not in the reverse direction. This phenomenon appears to have mostly or exclusively impacted the West of the Southern Cone, with ancient populations from Central East Brazil being seemingly uninvolved in this trend, consistent with a model proposed by previous work [23].

We attempted to quantify this putative contribution from a Mesoamerican-related source. Because *Chile_LosRieles_5100BP* gave the strongest signals —a phenomenon not attributable to this population label being better-powered than others, since it consists of a single individual of intermediate coverage—, we reasoned that it should provide an upper bound of the extent of this event in the other individuals tested. We used a rotating protocol in `qpAdm` in which we tested models of the form

$$\textit{Chile\_LosRieles\_5100BP} = \textit{Brazil\_LapaDoSanto\_9600BP} + X,$$

where X comes from a set of Californian and Mexican populations identified from the f_4 results (Supplementary Data 2). We found 4 models with plausible fits ($p > 0.01$), 3 of them

having a Mexican population as a source and 1 of them having a Californian population as a source (Supplementary Data 3). Under the natural assumption that these models are all underlain by the same signal, an inverse-variance-weighted meta-analysis across these 4 models estimates the Mesoamerican contribution into *Chile_LosRieles_5100BP* at 16.2±3.3% against the *Brazil_LapaDoSanto_9600BP* ancestry.

The differential affinity to Anzick has been translated to a model of two distinct waves of ancestry (one of them Anzick-related) into South American by the Early Holocene [16], whereas asymmetrical relationships to Mesoamerican populations among ancient South Americans has been interpreted as demonstrating a third ancestry movement into the subcontinent [24]. We tested this interpretation using `qpWave` (Methods), taking

```
Chile_PuntaSantaAna_7300BP.SG
Chile_LosRieles_5100BP.AG
Peru_Cuncaicha_9000BP.AG
Chile_LosRieles_12000BP.SG
Argentina_ArroyoSeco2_7700BP.AG
Brazil_MG_C_LapaDoSanto_EH_HG_9600BP.AG
Argentina_Central_Hills_SierrasChicas_JesusMaria_8500BP
```

as the set of left population, chosen to contain a representative of inferred early South American lineages: Southern Patagonia, Central East Brazil, Central Argentina, Middle Holocene Pampas, Central Andes and Central Chile, as well as the Anzick-related *LosRieles_12000BP* individual.

We tested for homogeneity against the following set of right populations:

```
YRI.DG
USA_Anzick_realigned.SG
USA_Ancient_Beringian.SG
USA_Nevada_SpiritCave_11000BP.SG
Belize_9400BP.AG
USA_CA_Goleta_4800BP.AG
USA_CA_CalaverasCounty_600BP.AG
Mexico_CoyoteCave_950BP.AG
USA_CA_LHolocene_Barbareno_Chumash.AG
```

chosen to contain representatives of deep American lineages sampled outside South America as well as Mesoamerican-related populations.

While a single-wave model was rejected ($p < 3 \times 10^{-4}$), we found no evidence to reject a two-wave model ($p > 0.12$) (Supplementary Data 2). This could be compatible with a recent suggestion that asymmetrical relatedness to Anzick is better explained by a model of structure on a gradient than by two independent pulses [17], but we may also be lacking a full set of appropriate reference populations.

On the other hand, affinity between late Central Andes individuals and ancient Californians has been taken to evince yet a fourth movement into South America [24]. Nevertheless, we note that Late Central Andes individuals in fact show greater attraction to

ancient Caribbean individuals than to ancient Californian individuals (Supplementary Data 2) ($Z > 6$) with respect to early Central Andes individuals. Considering that recent work has shown south-to-north migration in Central America [25], and that a recent study has pointed out that the California attraction is only detectable when considering Californian populations with Mexican gene glow [22], it seems plausible that the late Central Andes signal is instead driven by interactions within South America and subsequent back-migration.

SI Section 10 Relationships to previously-reported relevant lineages in the Americas

10.1 NNA and SNA ancestries

It has been previously claimed that all Southern Native Americans stem from the so-called "Southern North American" lineage, which unlike the "Northern North American" is reported to have produced waves of migration into South America ([2], [18]).

Using the *Canada_ASO.SG* ancient population as a proxy for NNA ancestry [26], and the *Chile_LosRieles_12000BP* individual as a proxy for SNA ancestry, we computed the statistic

$$f(\text{YRI.DG, Canada\_ASO.SG; Chile\_LosRieles\_12000BP, Newly-reported group}),$$

which should be sensitive to the presence of NNA ancestry. As expected, none of these statistics deviates significantly from 0 (highest $|Z| < 1.7$, Supplementary Data 1), consistent with all sampled populations falling on the SNA branch.

10.2 Population Y

Analogously, we used the statistic

$$f(\text{Yoruba.DG, Onge.DG/Papuan.DG; Arg pop, Mixe.DG}),$$

to test for a signal of relatedness to Australasian populations, where Mixe is a Native American population previously established to minimize the Population Y signal ([27]).

Overall, we found no strong evidence for a Population Y signal in these populations, with a single context label producing a Z slightly above 3 (*Argentina_Central_Hills_SouthPunilla_600BP*, which could merely constitute a statistical fluctuation (Supplementary Data 1).

SI Section 11 Genetic affinities of newly-reported Late Holocene individuals

To obtain formal results on the genetic affinities of newly-reported Late Holocene individuals, we computed f_4-statistics of the form $f_4(\text{Outgroup}, P_1; P_2, P_3)$ for each of our study subregions, where P_2 are Early/early Middle Holocene South Americans, P_3 are newly-reported context labels from the study subregion (and also modern populations in the case of the Gran Chaco region), and P_1 are all other modern or ancient populations of any time period, including those previously-published and those newly-reported sampled at other study subregions (Supplementary Data 4).

These statistics produced the following set of observations. First, Northwest individuals showed highest attraction to other newly-reported individuals from the same region ($3 < Z_{BY} < 15.2$) (Supplementary Data 4). Most of the context labels showed strong attraction to ancient ($3 < Z_{BY} < 11$) as well as modern ($3 < Z_{BY} < 9.6$) populations from the Central Andes and Northwest Argentina, like Quechua, Aymara or Diaguita, with individuals from the Northern Puna heading this trend (Supplementary Data 4). At the same time, Northwest context labels exhibited strong affinity to Central Argentina groups with respect to Middle Holocene Central Andes ($3 < Z_{BY} < 12.31$) (Supplementary Data 4), strengthening the evidence for mixture between these two lineages having taken place in the region (Table 1).

The Late Holocene individuals from Central Argentina showed the strongest attraction to other individuals from the same region ($3 < Z_{BY} < 27.1$) (Supplementary Data 4). At a threshold of $Z_{BY} > 3$, they exhibited no affinity to populations outside of the region with respect to *JesusMaria_8500BP* (Supplementary Data 4). Below that threshold, we identified weak excess allele-sharing with some ancient and modern Mesoamerican-related populations, including in the case of the second-oldest Central Argentina context label (*Argentina_Central_Hills_Calamuchita_4200BP*) (Supplementary Data 4). However, we observed no such attraction when comparing later individuals to *Calamuchita_4200BP* (Supplementary Data 5), suggesting this affinity stems from an event pre-dating 4200BP, likely the same that induced the above-reported Mesoamerican affinity in *LosRieles_5100BP*. Paucity of data between 8500BP and 4200BP thus precludes better characterization of this hypothesized genetic interaction.

The Late Holocene individuals from the Paraná River region showed strong attraction to the other newly-reported individuals ($3 < Z_{BY} < 16.3$), and no other clear patterns of affinity beyond some excess allele-sharing with Mesoamerican-related populations, analogous to the Central Argentina case (Supplementary Data 4).

The individuals from the Gran Chaco showed strong attraction to modern populations from the same or neighboring regions like Chané, Wichí, Guaraní or Toba ($3 < Z_{BY} < 6.9$), as did the Pantanal individual, to a lesser extent, who was also attracted to the Gran Chaco individuals (Supplementary Data 4). These also showed affinity to newly-reported context labels from Central Argentina and the Paraná river region ($3 < Z_{BY} < 5.73$), and to modern populations of Tropical and Subtropical Forests (TSF) ancestry, like Karitiana or Piapoco ($3 < Z_{BY} < 5.43$) (Supplementary Data 4). Conversely, they were also attracted to Central Argentina with respect to these TSF peoples, providing evidence of a history of gene flow between these two lineages in the Gran Chaco region (Table 1).

Finally, the individuals from the Pampas showed general attraction to other individuals

from the same region ($3 < Z_{BY} < 14.84$), and also to Central Argentina with respect to the Middle Holocene Pampas individuals ($3 < Z_{BY} < 9.49$) (Supplementary Data 4), suggesting an ancestry transition. Some of the Late Holocene Pampas context labels could not be related to Middle Holocene Pampas and the *Argentina_Central_JesusMaria_8500BP* via a simple tree (Table 1), indicating a history of admixture.

SI Section 12 Formal modelling of ancestry components

To formally model the ancestry components of the newly-reported populations, we used `qpAdm` (Methods), using the `allSNPs=YES` mode to maximize information content. We followed a hierarchical approach in which we asked what populations were consistent with being simple clades or simple two-way mixtures of the set of sources, cyclically assessing models with respect to the other sources and more distant outgroups, and adding complexity to failing single-source models only if needed. To reduce the burden of hypothesis testing, we considered only sources from one of the lineages above identified as relevant to the newly-reported individuals: Central Andes, Central Argentina, Middle Holocene Pampas, and Tropical and Subtropical Forests. However, we included a wide set of reference populations on the right, chosen to represent broader South American diversity, so that we were in a position to detect further ancestry components if missed by previous analyses.

In particular, the following set of reference (right) populations was used:

- Distant outgroup:
  - *YRI.DG* (Yoruba)
- Central East Brazil:
  - *Brazil_LapaDoSanto_9600BP.AG*
  - *Brazil_LocaDoSuin_Sambaqui_9100BP.AG*
  - *Brazil_JabuticabeiraII_Sambaqui_2400BP.AG*
- Central Andes:
  - *Peru_Lauricocha_8600BP.AG*
  - *Bolivia_Miraflores_MH.AG*
- Early Holocene Southern Puna:
  - *Argentina_Northwest_SouthernPuna_Antofagasta_PeñasdelasTrampas1.1_8800BP*
- Central Chile:
  - *Chile_LosRieles_5100BP.AG*
  - *Chile_Conchali_700BP.AG*
- Southern Patagonia:
  - *Chile_WesternArchipelago_Ayayema_5100BP.SG*
  - *Chile_Yamana_BeagleChannel_800BP.SG*
- Early Holocene Pampas:
  - *Argentina_Pampas_CentralPampeanDunefields_LagunadelosPampas_10000BP*
- Middle Holocene Pampas:
  - *Argentina_ArroyoSeco2_7700BP.AG*

- *Argentina_LagunaChica_6800BP.AG*

- Central Argentina:
  - *Argentina_Central_Hills_SierrasChicas_JesusMaria_8500BP*
  - *Argentina_Central_Plains_MiddleSaladoRiver_SantiagodelEstero_400BP*

- Tropical and Subtropical Forests:
  - *Karitiana.DG*

We first assessed the fit of single-source models for each one of the newly-reported groupings (Supplementary Figures 77–82), Extended Data Tables 2.5.-2.10.). Only in cases of failure, or fitting single-source models but other lines of evidence suggesting more than one ancestry component, did we consider additional sources. We considered models to fit if $p > 0.01$.

To minimize the risk of there having been gene flow between a source (left) and a reference (right) population (a violation of the assumptions underlying the `qpAdm` methodology [28]), we used the most recent of the proxies for each ancestry type.

12.1 Northwest Argentina

In this section, when assessing two-way admixture models with Central Argentina-type ancestry (represented by *Argentina_Central_Plains_MiddleSaladoRiver_SantiagodelEstero_400BP*) and Central Andes-type ancestry (represented by *Bolivia_Miraflores_MH.AG*) we added an additional Central-Andes proxy to the set of reference (right) populations (*Bolivia_Akapana_Tiwanaku.SG*), since this produced qualitatively similar results as not including it, yet provided much tighter standard errors (Supplementary Data 12).

1. Population *Northwest_NorthernPuna_Cochinoca_700BP* was only consistent with being cladal with *Bolivia_Miraflores_MH.AG* (Supplementary Figure 79, Supplementary Data 8). There was no indication from f_4−statistics that this was not the case (Supplementary Data 5), and this grouping clustered with Central Andes population in an outgroup f_3 tree (Supplementary Figure 1). For these reasons, we accepted the single-source model.

2. Population *Northwest_PrePuna_600BP* was only consistent with being cladal with *Bolivia_Miraflores_MH.AG* (Supplementary Figure 79, Supplementary Data 8). There was no indication from f_4−statistics that this was not the case (Supplementary Data 5), and this grouping clustered with Central Andes population in an outgroup f_3 tree (Supplementary Figure 1). For these reasons, we accepted the single-source model.

3. Population *Northwest_SouthernPuna_Antofagasta_1200BP* was only consistent with being cladal with *Bolivia_Miraflores_MH.AG* (Supplementary Figure 79, Supplementary Data 8). In f_4−statistics, however, it showed weak excess allele-sharing with *Argentina_Central_Plains_MiddleSaladoRiver_ SantiagodelEstero_400BP* ($|Z| > 2.44$) (Supplementary Data 5) and it clustered with Central Argentina in an outgroup f_3 tree

(Supplementary Figure 1). We had previously already identified evidence of this grouping being admixed between Central Argentina and Central Andes (Table 1). For this reason, we favored a model of two-way admixture, which was successful ($p > 0.285$) (Supplementary Data 12).

4. Population *Northwest_SouthernPuna_Antofagasta_2100BP* was not consistent with being cladal with any of the sources considered (Supplementary Figure 79, Supplementary Data 8). Examination of f_4−statistics indicated it was attracted to Central Argentina with respect to *Bolivia_Miraflores_MH.AG* ($|Z| > 3.25$) and vice-versa ($|Z| > 4.59$) (Supplementary Data 5), as we had already detected (Table 1). A 2-way admixture model involving these two sources was successful ($p > 0.888$) (Supplementary Data 12).

5. Population *Northwest_SouthernPuna_Antofagasta_4600BP* was consistent with being cladal with *Bolivia_Miraflores_MH.AG*, *Argentina_Central_Plains_ MiddleSaladoRiver_SantiagodelEstero_400BP* and *Karitiana.DG* (Supplementary Figure 79, Supplementary Data 8), although the latter is surely a result of low power, since the individual *Northwest_SouthernPuna_ Antofagasta_4600BP* had previously given no signs of affinity with *Karitiana.DG*. In an outgroup f_3 tree (Supplementary Figure 1), it clustered with other Northwest individuals within a clade containing Central Argentina. Additionally, it showed strong affinity to other Northwest individuals ($|Z| < 11.1$) as well as ancient and modern populations from the Central Andes ($|Z| < 6.02$) (Supplementary Data 4). For these reasons, we favored a model of two-way admixture, which was successful ($p > 0.684$) (Supplementary Data 12).

6. Population *Northwest_SouthernPuna_Antofagasta_500BP* was only consistent with being cladal with *Argentina_Central_Plains_MiddleSaladoRiver_SantiagodelEstero_400BP* (Supplementary Figure 79, Supplementary Data 8). Additionally, it showed strong affinity to other Northwest individuals ($|Z| < 6.02$) as well as some ancient populations from the Central Andes ($|Z| < 4.6$), even though this was less consistent than in the case of the other Northwest groupings (Supplementary Data 4). For these reasons, we considered a model of two-way admixture, which was successful ($p > 0.168$), but whose Central-Andes ancestry estimate was not unambiguously non-null (Supplementary Data 12).

7. Population *Northwest_SubandeanValleys_Aconquija_1600BP* was not consistent with being cladal with any of the sources considered (Supplementary Figure 79, Supplementary Data 8). In an outgroup f_3 tree (Supplementary Figure 1), it clustered with other Northwest individuals within a clade containing Central Argentina. Additionally, it showed strong affinity to other Northwest individuals ($|Z| < 6.67$) as well as ancient and modern populations from the Central Andes (Supplementary Data 4). For these reasons, we favored a model of two-way admixture, which was not successful ($p < 0.0011$) (Supplementary Data 12). Because the reason for this failure was unclear (perhaps small amounts of undetected contamination), we excluded *Northwest_SubandeanValleys_Aconquija_1600BP* from Figure 4a.

8. Population *Northwest_SubandeanValleys_Ambato_1200BP* was only consistent with be-

ing cladal with *Argentina_Central_Plains_MiddleSaladoRiver_SantiagodelEstero_400BP* (Supplementary Figure 79, Supplementary Data 8). Additionally, it showed strong affinity to other Northwest individuals ($|Z| < 11.633$) as well as modern and ancient populations from the Central Andes ($|Z| < 6.31$) (Supplementary Data 4). For these reasons, we favored a model of two-way admixture, which was successful ($p > 0.298$) (Supplementary Data 12).

9. Population *Northwest_SubandeanValleys_Belen_700BP* was not consistent with being cladal with any of the sources considered (Supplementary Figure 79, Supplementary Data 8). In an outgroup f_3 tree (Supplementary Figure 1), it clustered with other Northwest individuals within a clade containing Central Argentina. Additionally, it showed strong affinity to other Northwest individuals ($|Z| < 11.8$) as well as ancient and modern populations from the Central Andes ($|Z| < 7.5$) (Supplementary Data 4). For these reasons, we favored a model of two-way admixture, which was not successful ($p < 0.0014$) (Supplementary Data 12). Because the reason for this failure was unclear (perhaps small amounts of undetected contamination), we excluded *Northwest_SubandeanValleys_Belen_700BP* from Figure 4a.

10. Population *Northwest_SubandeanValleys_Hualfin_2400BP* was only consistent with being cladal with *Argentina_Central_Plains_MiddleSaladoRiver_SantiagodelEstero_400BP* (Supplementary Figure 79, Supplementary Data 8). Additionally, it showed strong affinity to other Northwest individuals ($|Z| < 13.1$) as well as modern and ancient populations from the Central Andes ($|Z| < 7.3$) (Supplementary Data 4). For these reasons, we favored a model of two-way admixture, which was successful ($p > 0.38$) (Supplementary Data 12).

12.2 Pantanal

1. Individual *Paraguay_Pantanal_ParaguayRiver_1600BP* was consistent with being cladal with *Karitiana.DG* and *Argentina_Central_Plains_MiddleSaladoRiver_SantiagodelEstero_400BP* (Supplementary Figure 78, Supplementary Data 10). In $f4-$statistics, it showed no evidence of cladality breaking with either of these context labels (Supplementary Data 5), but its overall strongest affinity was with populations from the Gran Chaco region, the Caribbean, or the Southeastern Brazilian coast ($Z < 5$) (Supplementary Data 4). This is consistent with the position of *Paraguay_Pantanal_ParaguayRiver_1600BP* in an outgroup$-f_3$ tree (Supplementary Figure 1), within a cluster of populations from Brazil and the Caribbean. For this reason, we considered it to be of fully Tropical and Subtropical Forests-like ancestry, but we caution that we appear to be ill-powered to fully resolve the ancestry profile of this individual due to relatively low coverage and sample size.

12.3 Gran Chaco

In this section, when assessing two-way admixture models with Central Argentina-type ancestry (represented by *Argentina_Central_Plains_MiddleSaladoRiver_SantiagodelEstero_400BP*) and Tropical and Subtropical Forests-type ancestry (TSF) (represented by *Karitiana.DG*) we added an additional proxy of the latter ancestry to the set of reference (right) populations (*Piapoco.DG*), since it helped in reducing estimate uncertainty. We also modeled the ancestry of modern populations from the Gran Chaco region: Chané, Wichí and Toba [2].

1. Population *Argentina_GranChaco_DryChaco_1900BP* was consistent with being cladal with *Karitiana.DG* and *Argentina_Central_Plains_MiddleSaladoRiver_SantiagodelEstero_400BP* (Supplementary Figure 77, Supplementary Data 7). In $f4-$statistics, it showed no clear evidence of cladality breaking with either of these context labels (Supplementary Data 5). However, in an outgroup$-f_3$ tree (Supplementary Figure 1), it fell within a cluster of populations from Brazil and the Caribbean. For this reason, we favored it to be of fully Tropical and Subtropical Forests-like ancestry, but we caution that we appear to be ill-powered to fully resolve the ancestry profile of this grouping due to relatively low coverage and sample size.

2. Population *Argentina_GranChaco_HumidChaco_1400BP* was consistent with being cladal with *Argentina_Central_Plains_MiddleSaladoRiver_SantiagodelEstero_400BP* and *Bolivia_Miraflores_MH.AG* (Supplementary Figure 77, Supplementary Data 7), although the latter is surely a result of low power, since this grouping had previously given no signs of affinity with Central-Andes populations. In $f4-$statistics, it showed no evidence of cladality breaking with the Central Argentina proxy (Supplementary Data 5). However, in an outgroup$-f_3$ tree, it fell within a cluster of populations from Brazil and the Caribbean, like the approximately contemporaneous Gran Chaco and Pantanal individuals (Supplementary Figure 1). For this reason, we considered a two-way admixture model including a Tropical and Subtropical Forests source (*Karitiana.DG*), which was successful ($p > 0.058$). The ancestry estimate for *Karitiana.DG*, however, was not unambiguously non-null (Supplementary Data 12).

3. Individual *Argentina_GranChaco_HumidChaco_200BP* was not consistent with being cladal with any of the considered sources (Supplementary Figure 77, Supplementary Data 7). Examination of f_4-statistics indicated it exhibited the highest affinity to *Argentina_Central_Plains_MiddleSaladoRiver_ SantiagodelEstero_400BP* and *Karitiana.DG*. In light of this and previously-identified evidence of admixture between these two ancestry components in modern Gran Chaco individuals (Table 1), we considered a two-way admixture model with these sources, which was not rejected ($p > 0.207$) (Supplementary Data 12).

4. Modern population *Wichí* was not consistent with being cladal with any of the considered sources (Supplementary Figure 77, Supplementary Data 7). In light of previously-identified evidence of admixture between these two ancestry components in this population (Table 1), we considered a two-way admixture model with these sources, which was not rejected ($p > 0.436$) (Supplementary Data 12).

5. Modern population *Toba* was not consistent with being cladal with any of the considered sources (Supplementary Figure 77, Supplementary Data 7). In light of previously-identified evidence of admixture between these two ancestry components in this population (Table 1), we considered a two-way admixture model with these sources, which was not rejected ($p > 0.609$) (Supplementary Data 12).

6. Modern population *Chané* was only consistent with being cladal with *Karitiana.DG* (Supplementary Figure 77, Supplementary Data 7). In $f4-$statistics, it showed no evidence of carrying other ancestry components (Supplementary Data 5). For this reason, we accepted the single-source model.

12.4 Paraná River

1. Population *ParanaRiver_LowerDelta_1600BP* was only consistent with being cladal with Central Argentina (Supplementary Figure 82, Supplementary Data 11). In f_4-statistics, it showed strong attraction to *Argentina_Central_Plains_MiddleSaladoRiver_ SantiagodelEstero_400BP* with respect to the other sources considered ($|Z| < 5.73$), and no clear attraction to any other source with respect to the latter (Supplementary Data 5). Furthermore, it fell within the Central Argentina cluster in an outgroup f_3 tree (Supplementary Figure 1). For these reasons, we accepted the single-source model with Central Argentina.

2. Population *ParanaRiver_LowerDelta_900BP* was not consistent with being cladal with any of the sources considered (Supplementary Figure 82, Supplementary Data 11). In f_4-statistics, it showed strong attraction to *Argentina_Central_Plains_MiddleSaladoRiver_ SantiagodelEstero_400BP* with respect to the other sources considered ($|Z| < 6.17$) (Supplementary Data 5), and no clear attraction to any other source with respect to the latter. However, *Argentina_Central_Plains_MiddleSaladoRiver_ SantiagodelEstero_400BP* showed some weak, diffuse attraction ($|Z| < 2.2$) to other divergent groups (like *Brazil_LapaDoSanto_9600BP.AG*, *Bolivia_Miraflores_MH.AG* and *Chile_LosRieles_5100BP.AG*) (Supplementary Data 5) with respect to this grouping, the only apparent reason for the failure of the single-source model. This pattern is perhaps best explained by a small amount of contamination present in the *ParanaRiver_LowerDelta_900BP* individuals that went undetected and causes *Argentina_Central_Plains_MiddleSaladoRiver_ SantiagodelEstero_400BP* to be repelled. Less likely, there could be ghost admixture in this grouping. For these reasons, we regard the failure of this single-source model as likely artifactual, but we excluded *ParanaRiver_LowerDelta_900BP* from Figure 4a.

3. Population *ParanaRiver_UpperDelta_700BP* was only consistent with being cladal with Central Argentina (Supplementary Figure 82, Supplementary Data 11). In f_4-statistics, it showed strong attraction to *Argentina_Central_Plains_MiddleSaladoRiver_ SantiagodelEstero_400BP* with respect to the other sources considered ($|Z| < 6.8$), and no attraction to any other source with respect to the latter (Supplementary Data 5). Furthermore, it fell within the Central Argentina cluster in an outgroup f_3 tree (Supplemen-

tary Figure 1). For these reasons, we accepted the single-source model with Central Argentina.

4. Population *ParanaRiver_MiddleParanaSaladoRivers_1700BP* was not consistent with being cladal with any of the sources considered (Supplementary Figure 82, Supplementary Data 11). In f_4-statistics, it showed strong attraction to *Argentina_Central_Plains_MiddleSaladoRiver_SantiagodelEstero_400BP* with respect to the other sources considered ($|Z| < 3.5$), and no clear attraction to any other source with respect to the latter. However, *Argentina_Central_Plains_MiddleSaladoRiver_ SantiagodelEstero_400BP* showed diffuse attraction ($|Z| < 3.1$) to several other divergent groups (like *Bolivia_ Miraflores_MH.AG*, *Brazil_JabuticabeiraII_Sambaqui_2400BP.AG* and *Brazil_LapaDoSanto_9600BP.AG*) with respect to this grouping (Supplementary Data 5), the only apparent reason for the failure of the single-source model. This pattern is perhaps best explained by a small amount of contamination present in the *ParanaRiver_MiddleParanaSaladoRivers_1700BP* individuals that went undetected and causes *Argentina_Central_Plains_MiddleSaladoRiver_SantiagodelEstero_400BP* to be repelled. Less likely, there could be ghost admixture in this grouping. For these reasons, we regard the failure of this single-source model as likely artifactual, but we excluded *ParanaRiver_MiddleParanaSaladoRivers_1700BP* from Figure 4a.

5. Population *ParanaRiver_MiddleParanaSaladoRivers_800BP* was not consistent with being cladal with any of the sources considered (Supplementary Figure 82, Supplementary Data 11). In f_4-statistics, it showed strong attraction to *Argentina_Central_Plains_MiddleSaladoRiver_SantiagodelEstero_400BP* with respect to the other sources considered ($|Z| < 13.14$), and no clear attraction to any other source with respect to the latter. However, *Argentina_Central_Plains_MiddleSaladoRiver_ SantiagodelEstero_400BP* showed weak, diffuse attraction ($|Z| < 2.66$) to divergent groups *Brazil_JabuticabeiraII_Sambaqui_2400BP.AG* and *Brazil_LapaDoSanto_9600BP.AG*) with respect to this grouping (Supplementary Data 5), the only apparent reason for the failure of the single-source model. This pattern is perhaps best explained by a small amount of contamination present in the *ParanaRiver_MiddleParanaSaladoRivers_800BP* individuals that went undetected and causes *Argentina_Central_Plains_MiddleSaladoRiver_ SantiagodelEstero_400BP* to be repelled. Less likely, there could be ghost admixture in this grouping. For these reasons, we regard the failure of this single-source model as likely artifactual, but we excluded *ParanaRiver_ MiddleParanaSaladoRivers_800BP* from Figure 4a.

12.5 Central Argentina

1. Population *Argentina_Central_Hills_Calamuchita_4200BP* was only consistent with being cladal with *Argentina_Central_Plains_MiddleSaladoRiver_ SantiagodelEstero_400BP* (Supplementary Figure 81, Supplementary Data 6). Because there was no clear indication from f_4-statistics (Supplementary Data 5) or other lines of evidence that this was not the case, we accepted the single-source model.

2. Population *Argentina_Central_Hills_Calamuchita_2600BP* was not consistent with being cladal with any of the considered sources (Supplementary Figure 81, Supplementary Data 6). In f_4-statistics, it appeared homogeneous with *Argentina_Central_Plains_MiddleSaladoRiver_SantiagodelEstero_400BP* (Supplementary Data 5) and it exhibited strongest affinity with other Central Argentina populations ($|Z| < 10.122$) (Supplementary Data 4). It also fell within the main Central Argentina cluster in an outgroup f_3 tree (Supplementary Figure 1). Since we could not identify a reason for the failure of this single-source model, we excluded *Argentina_Central_Hills_Calamuchita_2600BP* from Figure 4a.

3. Population *Argentina_Central_Hills_Calamuchita_700BP* was not consistent with being cladal with any of the considered sources (Supplementary Figure 81, Supplementary Data 6). Examination of f_4-statistics revealed the likely cause of this failure was excess allele-sharing between this grouping and Middle Holocene Pampas to the exclusion of *Argentina_Central_Plains_MiddleSaladoRiver_ SantiagodelEstero_400BP* ($Z < 4.33$) (Supplementary Data 5). However, when we attempted to model this context label as a two-way mixture of Central Argentina and Middle Holocene Pampas, the model was rejected ($p < 4.27 \cdot 10^{-3}$) (Supplementary Data 12). Since we could not identify a clear reason for failing to find a fitting ancestry model for this individual, we excluded *Argentina_Central_Hills_Calamuchita_700BP* from Figure 4a.

4. Population *Argentina_Central_Hills_Comechingones_4100BP* was was only consistent with being cladal with *Argentina_Central_Plains_MiddleSaladoRiver_ SantiagodelEstero_400BP* and *Bolivia_Miraflores_MH.AG* (Supplementary Figure 81, Supplementary Data 6), although the latter is surely a result of low power, since this individual had previously given no signs of affinity with Central-Andes populations. Because there was no indication of cladality breaking with Central Argentina from f_4-statistics (Supplementary Data 5) or other lines of evidence that this was not the case, we accepted the first single-source model.

5. Population *Argentina_Central_Hills_Comechingones_1200BP* was only consistent with being cladal with *Argentina_Central_Plains_MiddleSaladoRiver_ SantiagodelEstero_400BP* (Supplementary Figure 81, Supplementary Data 6). Because there was no indication from f_4-statistics (Supplementary Data 5) or other lines of evidence that this was not the case, we accepted the single-source model.

6. Population *Argentina_Central_Hills_Comechingones_500BP* was only consistent with being cladal with *Argentina_Central_Plains_MiddleSaladoRiver_ SantiagodelEstero_400BP* (Supplementary Figure 81, Supplementary Data 6). Because there was no indication from f_4-statistics or other lines of evidence that this was not the case (Supplementary Data 5 and 5), we accepted the single-source model.

7. Population *Argentina_Central_Hills_NorthCordoba_900BP* was only consistent with being cladal with *Argentina_Central_Plains_MiddleSaladoRiver_ SantiagodelEstero_400BP* (Supplementary Figure 81, Supplementary Data 6). Because there was no indication

from f_4−statistics (Supplementary Data 5) or other lines of evidence that this was not the case, we accepted the single-source model.

8. Population *Argentina_Central_Hills_NorthPunilla_700BP* was only consistent with being cladal with *Argentina_Central_Plains_MiddleSaladoRiver_ SantiagodelEstero_400BP* (Supplementary Figure 81, Supplementary Data 6). Because there was no indication from f_4−statistics (Supplementary Data 5) or other lines of evidence that this was not the case, we accepted the single-source model.

9. Population *Argentina_Central_Hills_Occientales_1900BP* was only consistent with being cladal with *Argentina_Central_Plains_MiddleSaladoRiver_ SantiagodelEstero_400BP* and *Bolivia_Miraflores_MH.AG* (Supplementary Figure 81, Supplementary Data 6), although the latter is surely a result of low power, since this individual had previously given no signs of affinity with Central-Andes populations. Because there was no indication of cladality breaking with Central Argentina from f_4−statistics (Supplementary Data 5) or other lines of evidence that this was not the case, we accepted the first single-source model.

10. Population *Argentina_Central_Hills_SierrasChicas_4100BP* was only consistent with being cladal with *Argentina_Central_Plains_MiddleSaladoRiver_ SantiagodelEstero_400BP* (Supplementary Figure 81, Supplementary Data 6). Because there was no indication from f_4−statistics (Supplementary Data 5) or other lines of evidence that this was not the case, we accepted the single-source model.

11. Population *Argentina_Central_Hills_SierrasChicas_1700BP* was only consistent with being cladal with *Argentina_Central_Plains_MiddleSaladoRiver_ SantiagodelEstero_400BP* (Supplementary Figure 81, Extended Data Tablen 2.5.). Because there was no clear indication from f_4−statistics (Supplementary Data 5) or other lines of evidence that this was not the case, we accepted the single-source model.

12. Population *Argentina_Central_Hills_SierrasChicas_700BP* was only consistent with being cladal with *Argentina_Central_Plains_MiddleSaladoRiver_ SantiagodelEstero_400BP* (Supplementary Figure 81, Supplementary Data 6). Because there was no indication from f_4−statistics (Supplementary Data 5) or other lines of evidence that this was not the case, we accepted the single-source model.

13. Population *Argentina_Central_Hills_SouthPunilla_2800BP* was only consistent with being cladal with *Argentina_Central_Plains_MiddleSaladoRiver_ SantiagodelEstero_400BP* (Supplementary Figure 81, Supplementary Data 6). Because there was no indication from f_4−statistics (Supplementary Data 5) or other lines of evidence that this was not the case, we accepted the single-source model.

14. Population *Argentina_Central_Hills_SouthPunilla_1600BP* was only consistent with being cladal with *Argentina_Central_Plains_MiddleSaladoRiver_ SantiagodelEstero_400BP* (Supplementary Figure 81, Supplementary Data 6). There was a weak indication of affinity with Middle Holocene Pampas from f_4−statistics (Supplementary Data 5). However, when we considered a two-way admixture model with this lineage and the

Central Argentina source, the fitted ancestry coefficient for the Middle Holocene Pampas component was negative (Supplementary Data 12). For this reason, we accepted the single-source model.

15. Population *Argentina_Central_Hills_SouthPunilla_600BP* was not consistent with being cladal with any of the considered sources (Supplementary Figure 81, Supplementary Data 6). In f_4−statistics, it appeared homogeneous with *Argentina_Central_Plains_MiddleSaladoRiver_ SantiagodelEstero_400BP* (Supplementary Data 5) and it exhibited strongest affinity with other Central Argentina populations ($|Z| < 20.4$) (Supplementary Data 4). It also fell within the main Central Argentina cluster in an outgroup f_3 tree (Supplementary Figure 1). Since we could not identify a reason for the failure of this single-source model, we excluded *Argentina_Central_Hills_SouthPunilla_600BP* from Figure 4a.

16. Population *Argentina_Central_Hills_Traslasierra_2500BP* was not consistent with being cladal with any of the considered sources (Supplementary Figure 81, Supplementary Data 6). In f_4−statistics, it exhibited strongest affinity with other Central Argentina populations ($|Z| < 15.294$) (Supplementary Data 4). It also fell within the main Central Argentina cluster in an outgroup f_3 tree (Supplementary Figure 1). There was indication of cladality breaking with *Argentina_Central_Plains_MiddleSaladoRiver_SantiagodelEstero_400BP* (Supplementary Data 5), in the form of excess allele-sharing between the former and *Brazil_LapaDoSanto _9600BP.AG* ($|Z| < 3.249$), who otherwise appeared fairly genetically divergent in all our analyses, which could be caused by a small amount of undetected contamination in *Argentina_Central_Hills_Traslasierra_2500BP*. Since we could not identify a population-history reason for the failure of this single-source model, we excluded *Argentina_Central_Hills_Traslasierra_2500BP* from Figure 4a.

17. Population *Argentina_Central_Hills_Traslasierra_800BP* was only consistent with being cladal with *Argentina_Central_Plains_MiddleSaladoRiver_ SantiagodelEstero_400BP* (Supplementary Figure 81, Supplementary Data 6). There was an indication of affinity with Middle Holocene Pampas from f_4−statistics ($|Z| < 3.37$) (Supplementary Data 5). However, when we considered a two-way admixture model with this lineage and the Central Argentina source, the fitted ancestry coefficient for the Middle Holocene Pampas component was negligible, and a Central Argentina-only model still fit (Supplementary Data 12). For this reason, we accepted the single-source model.

18. Population *Argentina_Central_Plains_EastCordoba_3700BP* was not consistent with being cladal with any of the considered sources (Supplementary Figure 81, Supplementary Data 6). In f_4−statistics, it appeared homogeneous with *Argentina_Central_Plains_MiddleSaladoRiver_ SantiagodelEstero_400BP* (Supplementary Data 5) and it exhibited strongest affinity with other Central Argentina populations ($|Z| < 8.1$) (Supplementary Data 5). It also fell within the main Central Argentina cluster in an outgroup f_3 tree (Supplementary Figure 1). Since we could not identify a reason for the failure of this single-source model, we excluded *Argentina_Central_Plains_EastCordoba_3700BP* from Figure 4a.

19. Population *Argentina_Central_Plains_EastCordoba_600BP* was only consistent with being cladal with *Argentina_Central_Plains_MiddleSaladoRiver_ SantiagodelEstero_400BP* (Supplementary Figure 81, Supplementary Data 6). Because there was no indication from f_4−statistics (Supplementary Data 5) or other lines of evidence that this was not the case, we accepted the single-source model.

20. Population *Argentina_Central_Plains_MarChiquita_3900BP* was only consistent with being cladal with *Argentina_Central_Plains_MiddleSaladoRiver_ SantiagodelEstero_400BP* (Supplementary Figure 81, Supplementary Data 6). Because there was no indication from f_4−statistics (Supplementary Data 5) or other lines of evidence that this was not the case, we accepted the single-source model.

21. Population *Argentina_Central_Plains_MarChiquita_2500BP* was only consistent with being cladal with *Argentina_Central_Plains_MiddleSaladoRiver_ SantiagodelEstero_400BP* (Supplementary Figure 81, Supplementary Data 6). Because there was no clear indication from f_4−statistics (Supplementary Data 5) or other lines of evidence that this was not the case, we accepted the single-source model.

22. Population *Argentina_Central_Plains_MarChiquita_1800BP* was not consistent with being cladal with any of the considered sources (Supplementary Figure 81, Supplementary Data 6). In f_4−statistics, it exhibited strongest affinity with other Central Argentina populations ($|Z| < 26.142$) (Supplementary Data 4). It also fell within the main Central Argentina cluster in an outgroup f_3 tree (Supplementary Figure 1). It appeared homogeneous with *Argentina_Central_Plains_MiddleSaladoRiver_ SantiagodelEstero_400BP*, safe perhaps for some weak excess allele-sharing between the former and *Argentina_ArroyoSeco2_7700BP.AG* ($|Z| < 2.69$) (Supplementary Data 5), which could be caused by a small amount of undetected contamination in *Argentina_Central_Plains_MarChiquita_1800BP*. Since we could not identify a reason for the failure of this single-source model, we excluded *Argentina_Central_Plains_MarChiquita_1800BP* from Figure 4a.

23. Population *Argentina_Central_Plains_MarChiquita_700BP* was only consistent with being cladal with *Argentina_Central_Plains_MiddleSaladoRiver_ SantiagodelEstero_400BP* (Supplementary Figure 81, Supplementary Data 6). Because there was no indication from f_4−statistics (Supplementary Data 5) or other lines of evidence that this was not the case, we accepted the single-source model.

24. Population *Argentina_Central_Plains_MesopotamiaSantiagoDelEstero_1700BP* was only consistent with being cladal with *Argentina_Central_Plains_MiddleSaladoRiver_ SantiagodelEstero_400BP* (Supplementary Figure 81, Supplementary Data 6). Because there was no indication from f_4−statistics (Supplementary Data 5) or other lines of evidence that this was not the case, we accepted the single-source model.

25. Population *Argentina_Central_Plains_MesopotamiaSantiagoDelEstero_1000BP* was only consistent with being cladal with *Argentina_Central_Plains_MiddleSaladoRiver_ SantiagodelEstero_400BP* (Supplementary Figure 81, Supplementary Data 6). Because there

was no indication from f_4−statistics (Supplementary Data 5) or other lines of evidence that this was not the case, we accepted the single-source model.

26. Population *Argentina_Central_Plains_NorthDulceRiver_500BP* was only consistent with being cladal with *Argentina_Central_Plains_MiddleSaladoRiver_ SantiagodelEstero_400BP* (Supplementary Figure 81, Supplementary Data 6). Because there was no clear indication from f_4−statistics (Supplementary Data 5) or other lines of evidence that this was not the case, we accepted the single-source model.

27. Population *Argentina_Central_Plains_NorthSantiagodelEstero_500BP* was only consistent with being cladal with *Argentina_Central_Plains_MiddleSaladoRiver_ SantiagodelEstero_400BP* (Supplementary Figure 81, Supplementary Data 6). Because there was no indication from f_4−statistics (Supplementary Data 5) or other lines of evidence that this was not the case, we accepted the single-source model.

28. Population *Argentina_Central_Plains_SouthCordoba_150BP* was not consistent with being cladal with any of the considered sources (Supplementary Figure 81, Supplementary Data 6). In f_4−statistics, it exhibited strongest affinity with other Central Argentina populations ($|Z| < 12.710$) (Supplementary Data 4). It also fell within the main Central Argentina cluster in an outgroup f_3 tree (Supplementary Figure 1). It appeared homogeneous with *Argentina_Central_Plains_MiddleSaladoRiver_ SantiagodelEstero_400BP*, safe perhaps for some weak excess allele-sharing between *Argentina_Central_Plains_ SouthCordoba_150BP* and *Argentina_NorthTierradelFuego_ LaArcillosa2_5800BP.AG* ($|Z| < 2.72$) (Supplementary Data 5). However, when we attempted to move that Southern Patagonia individual to the left as a source, the resulting two-way admixture model was rejected ($p < 7.17 \cdot 10^{-3}$) (Supplementary Data 12). Since we could not identify a clear reason for failing to find a fitting ancestry model for this individual, we excluded *Argentina_Central_Plains_SouthCordoba_150BP* from Figure 4a.

29. Population *Argentina_Central_Plains_WestSantiagodelEstero_1400BP* was not consistent with being cladal with any of the considered sources (Supplementary Figure 81, Supplementary Data 6). In f_4−statistics, it appeared mostly homogeneous with *Argentina_Central_Plains_MiddleSaladoRiver_ SantiagodelEstero_400BP* (Supplementary Data 5) and it exhibited strongest affinity with other Central Argentina populations ($|Z| < 12.892$) (Supplementary Data 4). It also fell within the main Central Argentina cluster in an outgroup f_3 tree (Supplementary Figure 1). Since we could not identify a reason for the failure of this single-source model, we excluded *Argentina_Central_Plains_ WestSantiagodelEstero_1400BP* from Figure 4a.

12.6 Pampas

1. Population *Argentina_Pampas_Southern_2600BP* was not consistent with being cladal with any of the sources considered (Supplementary Figure 80, Supplementary Data 9). Examination of f_4−statistics indicated it was attracted to Central Argentina with

respect to Middle Holocene Pampas ($|Z| > 6$) and vice-versa ($|Z| > 10.95$) (Supplementary Data 5). A 2-way admixture model involving these two sources was successful ($p > 0.0195$) (Supplementary Data 12).

2. Population *Argentina_Pampas_West_LagunaChica_1600BP* was not consistent with being cladal with any of the sources considered (Supplementary Figure 80, Supplementary Data 9). Examination of f_4−statistics indicated it was attracted to Central Argentina with respect to Middle Holocene Pampas ($|Z| > 6.4$) and vice-versa ($|Z| > 5.76$) (Supplementary Data 5). A 2-way admixture model involving these two sources was successful ($p > 0.083$) (Supplementary Data 12).

3. Population *Argentina_Pampas_Southern_800BP* was not consistent with being cladal with any of the sources considered (Supplementary Figure 80, Supplementary Data 9). Examination of f_4−statistics indicated it was attracted to Central Argentina with respect to Middle Holocene Pampas ($|Z| > 5.6$) (Supplementary Data 5). A 2-way admixture model involving these two sources was successful ($p > 0.038$) (Supplementary Data 12).

4. Population *Argentina_Pampas_SouthSaladoRiver_3300BP* was not consistent with being cladal with any of the sources considered (Supplementary Figure 80, Supplementary Data 9). Even though examination of f_4−statistics did not indicate clear attractions, perhaps due to a lack of power, (Supplementary Data 5), it showed strong affinity to other Late Holocene Pampas populations ($|Z| > 5.3$) (Supplementary Data 4), and it clustered with them in an outgroup f_3 tree (Supplementary Figure 1). For this reason, we considered a 2-way admixture model involving a Central Argentina and a Middle Holocene Pampas source, which was successful ($p > 0.425$) (Supplementary Data 12).

SI Section 13 Relationship of `qpAdm` to `ADMIXTURE` results

We note that unsupervised ADMIXTURE clustering analysis considering modern and ancient South American individuals was scarcely informative, since the cross-validation error showed a strictly monotonically increasing pattern with the number of ancestral components K (Supplementary Figure 13), indicating an overall bad fit to the data. Inconsistencies were also observed for different random iterations of the same K. Still, at $K = 4$ (the number of relevant ancestry components highlighted by previous analyses) ADMIXTURE and qpAdm results were broadly consistent in that both inferred most of the newly-reported individuals to be homogeneous with a Central Argentina source, with Central Andes-type ancestry detected in the individuals from the Northwest −maximized by Northern Puna and PrePuna individuals− and a component represented by either Middle Holocene Pampas or Southern Patagonia individuals detected in the Late Holocene Pampas individuals (Extended Data Figures 14- 17 show results for 4 random runs of ADMIXTURE for $K = 4$, and full raw output for $K = 1, \ldots, 12$ is available at https://github.com/javiermaravall/aDNA_CSC/).

References

1. Luisi, P. *et al.* Fine-scale genomic analyses of admixed individuals reveal unrecognized genetic ancestry components in Argentina. *PLoS ONE* **15.** ISSN: 19326203 (7 2020).
2. Reich, D. *et al.* Reconstructing Native American population history. *Nature* **488,** 370–374. ISSN: 1476-4687. https://doi.org/10.1038/nature11258 (Aug. 2012).
3. Fu, Q. *et al.* A Revised Timescale for Human Evolution Based on Ancient Mitochondrial Genomes. *Current Biology* **23,** 553–559. ISSN: 0960-9822. https://www.sciencedirect.com/science/article/pii/S0960982213002157 (2013).
4. Huang, Y. & Ringbauer, H. hapCon: estimating contamination of ancient genomes by copying from reference haplotypes. *Bioinformatics* **38,** 3768–3777. ISSN: 1367-4803. eprint: https://academic.oup.com/bioinformatics/article-pdf/38/15/3768/49884336/btac390.pdf. https://doi.org/10.1093/bioinformatics/btac390 (June 2022).
5. Korneliussen, T. S., Albrechtsen, A. & Nielsen, R. ANGSD: Analysis of Next Generation Sequencing Data. *BMC Bioinformatics* **15,** 356. ISSN: 1471-2105. https://doi.org/10.1186/s12859-014-0356-4 (Nov. 2014).
6. Maier, R. & Patterson, N. *admixtools: Inferring demographic history from genetic data* R package version 2.0.0 (2023). https://github.com/uqrmaie1/admixtools.
7. Maier, R. *et al.* On the limits of fitting complex models of population history to *f*-statistics. *eLife* **12** (eds Nordborg, M., Przeworski, M., Balding, D. & Wiuf, C.) e85492. ISSN: 2050-084X. https://doi.org/10.7554/eLife.85492 (Apr. 2023).
8. Consortium, 1. G. P. *et al.* A global reference for human genetic variation. *Nature* **526,** 68 (2015).
9. R Core Team. *R: A Language and Environment for Statistical Computing* ISBN 3-900051-07-0. R Foundation for Statistical Computing (Vienna, Austria, 2013). http://www.R-project.org/.
10. Paradis, E. & Schliep, K. ape 5.0: an environment for modern phylogenetics and evolutionary analyses in R. *Bioinformatics* **35,** 526–528 (2019).
11. Zhang, J. *spaa: SPecies Association Analysis* R package version 0.2.2 (2016). https://CRAN.R-project.org/package=spaa.
12. Van Rossum, G. & Drake, F. L. *Python 3 Reference Manual* ISBN: 1441412697 (CreateSpace, Scotts Valley, CA, 2009).
13. Huerta-Cepas, J., Serra, F. & Bork, P. ETE 3: Reconstruction, Analysis, and Visualization of Phylogenomic Data. *Molecular Biology and Evolution* **33,** 1635–1638. ISSN: 0737-4038. eprint: https://academic.oup.com/mbe/article-pdf/33/6/1635/7953632/msw046.pdf. https://doi.org/10.1093/molbev/msw046 (Feb. 2016).
14. Felsenstein, J. PHYLIP : phylogeny inference package (version 3.2). *Cladistics* **5,** 164–166 (1989).
15. Rasmussen, M. *et al.* The genome of a Late Pleistocene human from a Clovis burial site in western Montana. *Nature* **506,** 225–229. ISSN: 1476-4687. https://doi.org/10.1038/nature13025 (Feb. 2014).
16. Posth, C. *et al.* Reconstructing the Deep Population History of Central and South America. *Cell* **175.** ISSN: 10974172 (5 2018).

17. Ferraz, T. *et al.* Genomic history of coastal societies from eastern South America. *Nature Ecology & Evolution* **7,** 1315–1330. ISSN: 2397-334X. https://doi.org/10.1038/s41559-023-02114-9 (Aug. 2023).

18. Moreno-Mayar, J. V. *et al.* Early human dispersals within the Americas. *Science* **362.** ISSN: 10959203 (6419 2018).

19. Nakatsuka, N. *et al.* A Paleogenomic Reconstruction of the Deep Population History of the Andes. *Cell* **181,** 1131–1145.e21. ISSN: 0092-8674. https://doi.org/10.1016/j.cell.2020.04.015 (May 2020).

20. Nakatsuka, N. *et al.* Ancient genomes in South Patagonia reveal population movements associated with technological shifts and geography. *Nature Communications* **11,** 3868. ISSN: 2041-1723. https://doi.org/10.1038/s41467-020-17656-w (Aug. 2020).

21. Arango-Isaza, E. *et al.* The genetic history of the Southern Andes from present-day Mapuche ancestry. *Current Biology* **33.** ISSN: 18790445 (13 2023).

22. Nakatsuka, N. *et al.* Genetic continuity and change among the Indigenous peoples of California. *Nature* **624,** 122–129. ISSN: 1476-4687. https://doi.org/10.1038/s41586-023-06771-5 (Dec. 2023).

23. Capodiferro, M. R. *et al.* Archaeogenomic distinctiveness of the Isthmo-Colombian area. *Cell* **184,** 1706–1723.e24. ISSN: 0092-8674. https://www.sciencedirect.com/science/article/pii/S0092867421002294 (2021).

24. Willerslev, E. & Meltzer, D. J. Peopling of the Americas as inferred from ancient genomics. *Nature* **594,** 356–364. ISSN: 1476-4687. https://doi.org/10.1038/s41586-021-03499-y (June 2021).

25. Kennett, D. J. *et al.* South-to-north migration preceded the advent of intensive farming in the Maya region. *Nature Communications* **13,** 1530. ISSN: 2041-1723. https://doi.org/10.1038/s41467-022-29158-y (Mar. 2022).

26. Scheib, C. L. *et al.* Ancient human parallel lineages within North America contributed to a coastal expansion. *Science* **360,** 1024–1027. eprint: https://www.science.org/doi/pdf/10.1126/science.aar6851. https://www.science.org/doi/abs/10.1126/science.aar6851 (2018).

27. Skoglund, P. *et al.* Genetic evidence for two founding populations of the Americas. *Nature* **525,** 104–108. ISSN: 1476-4687. https://doi.org/10.1038/nature14895 (Sept. 2015).

28. Haak, W. *et al.* Massive migration from the steppe was a source for Indo-European languages in Europe. *Nature* **522,** 207–211. ISSN: 1476-4687. https://doi.org/10.1038/nature14317 (June 2015).
